# Diatom Cell Size, Coloniality and Motility: Trade-Offs between Temperature, Salinity and Nutrient Supply with Climate Change

**DOI:** 10.1371/journal.pone.0109993

**Published:** 2014-10-03

**Authors:** Filip Svensson, Jon Norberg, Pauline Snoeijs

**Affiliations:** 1 Department of Ecology, Environment and Plant Sciences, Stockholm University, Stockholm, Sweden; 2 Stockholm Resilience Centre, Stockholm University, Stockholm, Sweden; University of Lincoln, United Kingdom

## Abstract

Reduction in body size has been proposed as a universal response of organisms, both to warming and to decreased salinity. However, it is still controversial if size reduction is caused by temperature or salinity on their own, or if other factors interfere as well. We used natural benthic diatom communities to explore how “body size” (cells and colonies) and motility change along temperature (2–26°C) and salinity (0.5–7.8) gradients in the brackish Baltic Sea. Fourth-corner analysis confirmed that small cell and colony sizes were associated with high temperature in summer. Average community cell volume decreased linearly with 2.2% per °C. However, cells were larger with artificial warming when nutrient concentrations were high in the cold season. Average community cell volume increased by 5.2% per °C of artificial warming from 0 to 8.5°C and simultaneously there was a selection for motility, which probably helped to optimize growth rates by trade-offs between nutrient supply and irradiation. Along the Baltic Sea salinity gradient cell size decreased with decreasing salinity, apparently mediated by nutrient stoichiometry. Altogether, our results suggest that climate change in this century may polarize seasonality by creating two new niches, with elevated temperature at high nutrient concentrations in the cold season (increasing cell size) and elevated temperature at low nutrient concentrations in the warm season (decreasing cell size). Higher temperature in summer and lower salinity by increased land-runoff are expected to decrease the average cell size of primary producers, which is likely to affect the transfer of energy to higher trophic levels.

## Introduction

Climate change over the next century is projected to alter the environmental conditions for primary producers. The Intergovernmental Panel on Climate Change (IPCC) predicts a global temperature increase of 2–4.5°C over the coming 100 years [1]. Regional climate scenarios for the brackish Baltic Sea area predict, besides an increase in the sea surface temperature, a decrease in salinity with increased land runoff due to precipitation [2], [3]. Signs of an on-going climate change have already been observed in the temperature record. Historical data on the maximum summer surface water temperature in the Baltic Sea show an increase of ∼1.3°C between 1861–1900 and 1985–2005 [4]. The two decades 1985–2005 also had an increased frequency of warm winters. Although decadal variations in the mean surface salinity in the Baltic Sea have been recorded, no overall long-term trend was detected in the 20th century [5].

The changing environment created by climate change will alter the composition of biological communities. Critical questions to be answered are how communities will reorganize and what the consequences of these community changes will be for ecosystem functioning. To predict and assess community shifts and their consequences, ecologists are increasingly investigating how the functional traits of primary producers determine their relative fitness along environmental gradients [6]. The trait-based approach in ecology offers the opportunity to tackle the complexity of species-rich communities by constructing simple taxon-independent models of community structure and dynamics in relation to the environment. By defining species according to their form and function, communities with different species can be compared through the “common currency” of their traits. The use of a trait-based approach has helped to elucidate ecological patterns in a wide range of research fields, e.g. biogeography in microbiology [7], leaf economic spectra in terrestrial plant ecology [8] and distributional changes of marine fish due to climate change in marine ecology [9].

For unicellular organisms, cell size is considered to be the “master trait”. Cell size determines to a large extent how they respond to changes in the environment [10], by affecting several crucial ecological processes such as light harvesting [11], nutrient uptake [12], growth rate [13], and predator avoidance [14]. It is generally assumed that global warming shifts the distribution of phytoplankton size towards smaller individuals with rapid turnover and low standing biomass, resulting in a reorganization of food web structure and functioning [15], [16]. However, it is still controversial if this size reduction is caused by temperature alone, or if other factors, notably nutrient supply, interfere as well. Under controlled laboratory conditions the intraspecific cell size of microalgae tends to decrease with increasing temperature [17], [18], [19]. However, for a natural community with hundreds of different species exposed to environmental change, it is more likely that community changes occur at the interspecific level by changes in the average community size composition [6], [20]. In open oceans and deep lakes, temperature, stratification and nutrient concentrations are often tightly coupled to each other, and together these factors shape the size spectra of the phytoplankton community [21], [22], [23]. Phytoplankton cell size is also known to decrease with decreasing salinity [12]. Thus, in areas where increased land runoff is expected to occur with climate change, like in the Baltic Sea [2], [3], size reduction may be caused by salinity as well.

Compared with phytoplankton communities, little is known about the trait-based ecology of microphytobenthic communities, which are important primary producers in the coastal zone. In temperate waters these communities are usually nearly completely dominated by diatoms. Benthic diatom communities are typically species-rich [24] and display large variation in community composition and growth forms in response to changes in substrate, mechanical disturbance, grazing pressure, light intensity and nutrient availability [25]. In this paper we focus on the cell and colony size and motility of the diatoms. Within benthic diatom communities these traits exhibit large interspecific variation while the basic physiological traits are similar among species.

Like many other organism groups, the diatoms display a much larger interspecific than intraspecific size variation. Measurements on the intraspecific length of four common diatom species in the Baltic Sea displayed changes in average length with a factor 1–3 in different populations along a 1400 km salinity gradient [26]. In *Diatoma moniliformis*, the average length varied a factor 1.7 between populations sampled at different times throughout the year, while single cells varied between 3 and 80 µm in length in 34,000 measured valves [27]. Interspecific size in diatoms, on the other hand, ranges from the smallest *Cyclotella* species with a minimum diameter of ∼3 µm to *Ethmodiscus* with a maximum diameter of ∼2 mm. This large difference in one dimension translates to enormous differences in surface area and cell volume between species. For example, the largest recorded difference in the average volume between the smallest and the largest diatom species in the Baltic Sea is a factor 678,000 [28].

The formation of single-species colonies may also be regarded as an increase in “body size” and is another ecologically relevant trait that may be affected by environmental factors. For example, the chain length of *Skeletonema costatum* increases with growth rate and was shown to be stimulated by higher temperature and nutrient availability [29]. Chain length affects the sinking rate of phytoplankton diatoms since longer chains remain suspended for longer times in the photic zone. Colonial growth allows benthic diatoms to extend three-dimensionally into the water column, which provides competitive advantages, e.g. in the competition for light and nutrients [30].

Diatoms are not able to move in water (except for flagellated male gametes), but species that possess a raphe can glide over a surface and reach speeds >10 µm s^−1^ [31]. Motile diatom species have a competitive advantage over their attached relatives as they can actively expose themselves to optimal light conditions, i.e. they can move towards the light for light harvesting and they can also move away from supersaturating irradiance to avoid oxidative damage [31], [32]. Another advantage of motility is that the cells can move to more nutrient-rich microhabitats when nutrients are limiting growth.

In this paper we apply a trait approach on two previously published comprehensive ecological data sets of brackish-water benthic diatom communities along environmental gradients, a combined seasonal and man-made temperature gradient and a natural stable salinity gradient. We explore how “body size” (of cells and colonies) and motility change along the gradients and how they are associated with other environmental and biotic variables. Specifically, we test the following hypotheses for the average community traits of the diatom taxa: (1) Cell size and coloniality decrease and motility increases with higher temperature (2) Cell size and coloniality decrease and motility increases with lower salinity, (3) Nutrient supply interferes with temperature and salinity effects on cell size, coloniality and motility.

## Materials and Methods

### Diatom data

Two diatom data sets, one along a temperature gradient and another along a salinity gradient, were used in this study (Tables S1–S4). These data sets have previously been used to assess changes in species composition in relation to environmental variables [33], [34]. The diatom communities were sampled from stone surfaces at 0.2–0.7 m of water depth in the brackish Baltic Sea. Each sample consists of 1000 diatom valves, which were identified to the species or variety level from permanent diatom slides under a light microscope at magnification ×1000.

**Figure 1:**
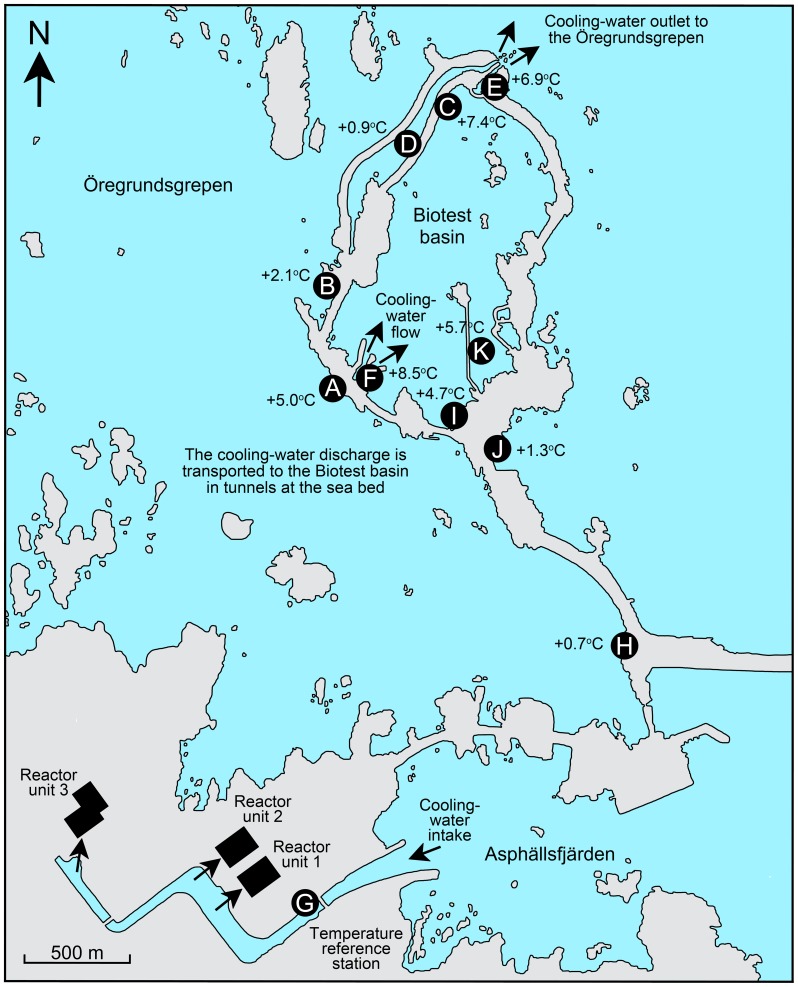
Map of the temperature gradient. The locations of the 11 sampling sites (Sites A–K) in the artificial temperature gradient outside the Forsmark nuclear power plant are indicated. The temperatures shown at the sampling sites indicate the average temperature anomaly (N = 11 sampling dates) compared to Site G in the cooling-water intake channel where the reference temperature was measured on each sampling date. Figure © Pauline Snoeijs.

**Figure 2:**
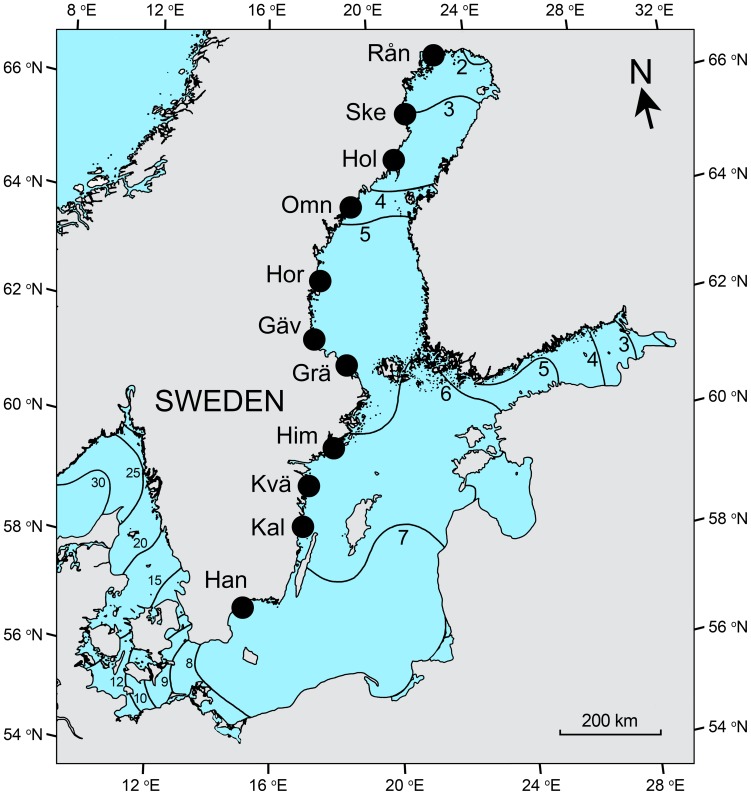
Map of the salinity gradient. The locations of the 11 sampling areas in the Baltic Sea along the Swedish coast are indicated. Forsmark (Figure 1) is located in the Gräsö area. Han  =  Hanösund, Kal  =  Kalmarsund, Kvä  =  Kvädöfjärden, Him  =  Himmerfjärden, Grä  =  Gräsö, Gäv  =  Gävlebukten, Hor  =  Hornslandet, Omn  =  Omnefjärden, Hol  =  Holmön, Ske  =  Skelleftehamn, Rån  =  Rånefjärden. Isohalines indicate the surface salinity of the Baltic Sea and adjacent waters in the transition zone to the North Sea to the left. Figure © Pauline Snoeijs.

The temperature data set included 121,000 valves belonging to 230 diatom taxa, sampled from 11 sites on 11 dates every three weeks during the ice-free season (early May – late November 1984). The sampling area comprised a few km^2^ in the vicinity of a large cooling-water discharge from the Forsmark nuclear power plant to the Bothnian Sea in the northern Baltic Sea (60°37′ N, 18°16′ E; Figure 1, in the Gräsö area in Figure 2). The salinity data set included 119,000 valves belonging to 355 diatom taxa, sampled from 119 sites during the benthic diatom spring bloom, a few weeks after ice-break (April – May 1990 and 1991). The sampling sites were located in 11 areas along the stable Baltic Sea salinity gradient and comprised ∼1400 km of the Swedish coast between 56°00′ N and 65°49′ N and 14°44′ E and 22°42′ E (Figure 2). Permissions for both studies were given by the Swedish Board of Fisheries.

### Environmental data

**Figure 3:**
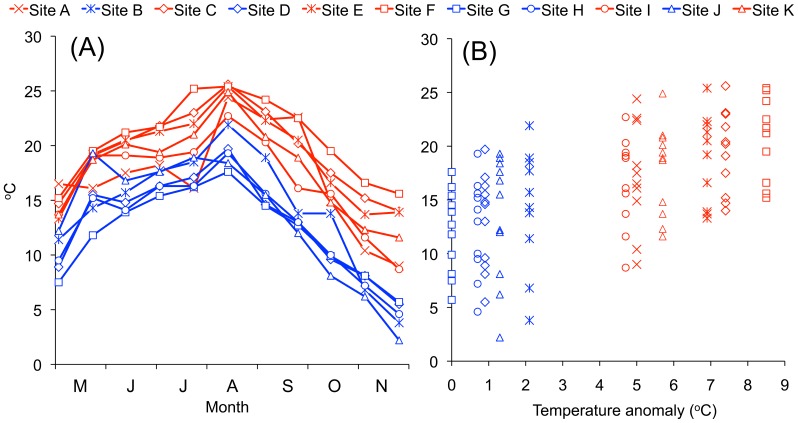
Summary of the temperature gradient with five unheated sites and six heated sites. (A) Water temperature measured during sampling for the 11 sites on the 11 dates. (B) Water temperature measured during sampling plotted against the average temperature anomaly per site (Spatanom).

The Forsmark area can be considered as an ecological temperature experiment at full natural scale [35]. The Biotest basin (Figure 1) is a ∼1 km^2^ artificial enclosure through which the cooling water from the Forsmark nuclear power plant is discharged. This enclosure was especially built for research purposes in the late 1970′s. Six of the 11 sampling sites used in this study were directly affected by the cooling water from the nuclear power plant and five sites were not (Figure 1) [33]. Overall, water temperature ranged between 2 and 26°C during the sampling period (Figure 3) [33]. The reference water temperature in the area (“Seastemp”), which we define as the temperature in the intake channel (Site G) of the cooling water for the power plant ranged between 6 and 18°C. Compared to Site G, the water temperature was a few °C lower in the cold season (lowest 2°C) and a few °C higher in July – August (highest 22°C) at the four other sampling sites outside the reach of the cooling water (Sites B, D, H, J; Figures 1 and 3). This was caused by a combination of factors: the cooling water that passed Site G was taken from 2–3 m of water depth, Site J was situated in a shallow bay and the water temperature was strongly influenced by solar irradiation, and Sites B and D could be slightly influenced by leakage of warm water from the Biotest basin through the artificial dams.

A spatial temperature anomaly (“Spatanom”) was calculated for each sampling site as the average temperature at the site minus the average temperature at Site G in the intake channel (N = 11 sampling dates). The Spatanom varied between 0 and 8.5°C among the sites (Figures 1 and 3). In this way it was possible to analyze the effects of the absolute temperature during sampling (the temperature gradient), the effects of seasonal variation in temperature in the area as recorded at Site G (Seastemp) and the effects of long-term artificial warming (Spatanom). Further environmental factors used in the present study are daily average photosynthetically active radiation (PAR) at the water surface calculated from 24 hourly measurements per day (range in the data set 47–523 µmol photons PAR m^−2^ s^−1^), dissolved inorganic nitrogen concentrations in the water (DIN: 0.1–6.4 µmol L^−1^), dissolved inorganic phosphorus in the water (DIP: 0.05–0.24 µmol L^−1^), dissolved inorganic silicate in the water (DSi: 4–23 µmol L^−1^), the DIN:DIP ratio (N:P: 2–33 on a molar basis) and a “flow factor”, which describes water movement according to an ordinal scale (FLOW, Table S5) for each site [33]. PAR and nutrient concentrations varied with season and exposure varied with sampling site. The salinity in the Forsmark area of 5 is stable year-round [33].

**Figure 4:**
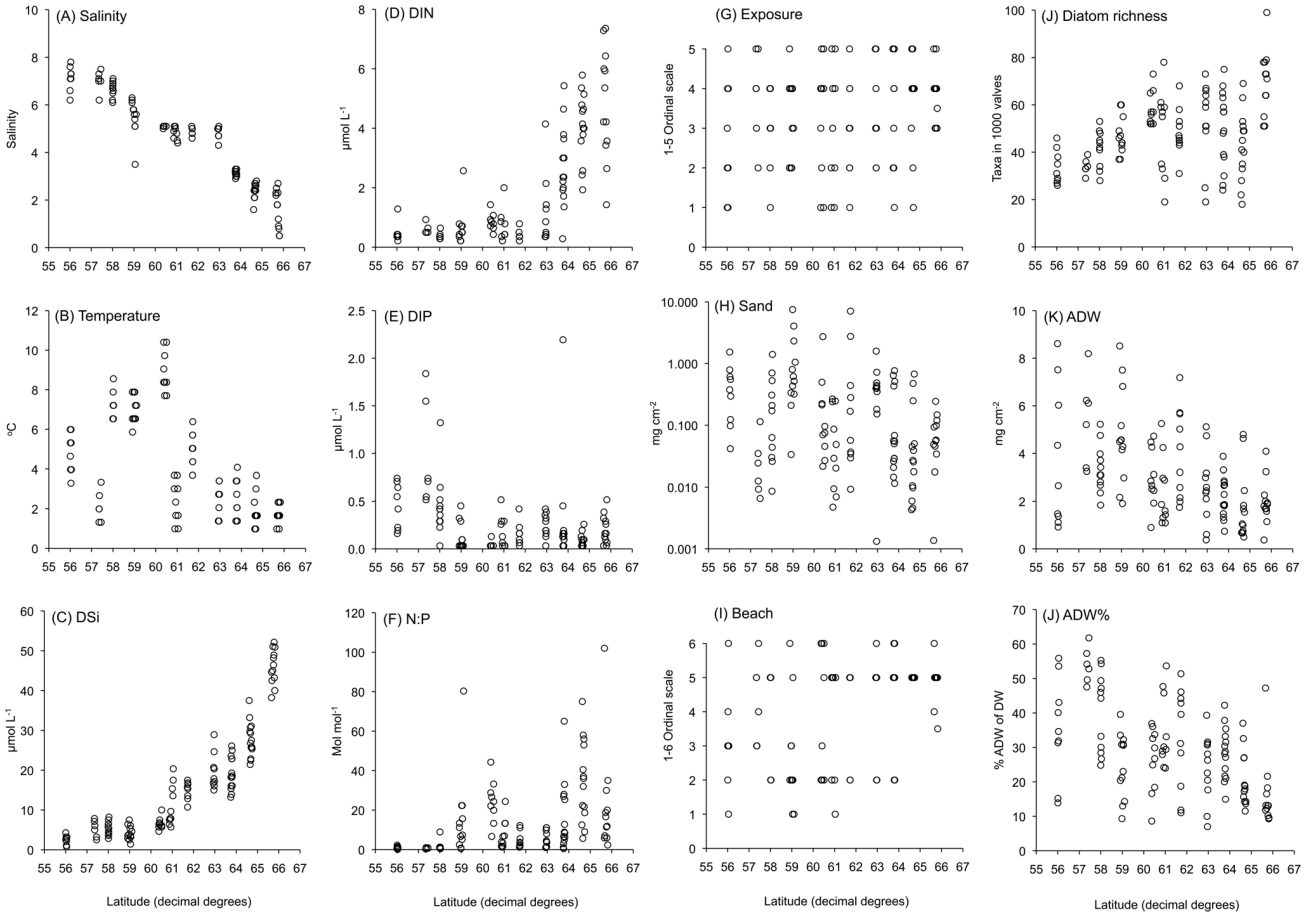
Summary of the variation of the environmental and biotic variables with latitude along the Baltic Sea salinity gradient. (A) Salinity. (B) Surface water temperature. (C) Dissolved inorganic silicate. (D) Dissolved inorganic nitrogen. (E) Dissolved inorganic phosphorus. (F) molar N:P ratio. (G) Exposure to waves. (H) Ash weight of sand grains on the stones. (I) Beach type. (J) Diatom richness. (K) Ash-free dry weight (a measure of biomass on the stones). (L) % Ash-free dry weight (a measure of the relative amount of macroalgae on the stones).

The salinity gradient ranged from 0.5 to 7.8 at the 119 sampling sites along the Swedish coast (Figures 2 and 4) [34]. Within most of the areas, the sites represented small salinity gradients from the mainland to the outer archipelago. The Baltic Sea is a brackish sea with large freshwater runoff and limited water exchange with the North Sea. Lunar tides are virtually absent and the water level is mainly determined by air pressure. The absence of tides, the inflow of freshwater from >200 rivers and the limited exchange of water together create a long and stable salinity gradient. The salinity gradient is correlated to a north-south climate gradient and the period during which the coastal zone is ice-covered in winter is longer with increasing latitude [36]. Further environmental factors used in the present study (Figure 4) are: Temperature (range in the data set 1.0–10.4°C), DIN (0.2–7.4 µmol L^−1^), DIP (0.03–2.2 µmol L^−1^, and one outlier value of 16 µmol L^−1^), DSi (1–52 µmol L^−1^), N:P (0.1–102 on a molar basis), the amount of sand on the stones (SAND: 0–7.5 mg cm^−2^) and exposure to wave action (EXP) and beach type (BEACH) estimated according to ordinal scales (Table S5) for each site [34].

At the coastal sites along the salinity gradient the water temperature measured in the field was strongly influenced by daily variation in solar irradiation in relation to exposure to waves. For example, at one of the sampling sites a difference of 6°C in water temperature was measured between early morning and late afternoon. To compensate for this discrepancy a model was developed to estimate the average daily temperature at each sampling site. The linear model *Temp_site_  =  β_1_·Temp_SMHI_ + β_2_·EXP +2* was used. *Temp_site_* is the measured water temperature at the site during sampling, *Temp_SMHI_* is the sea temperature recorded by the Swedish Meteorological and Hydrological Institute (SMHI; www.smhi.se) at a station close to the sampling site in time and space. *EXP* is the degree of exposure of each site on an ordinal scale (Table S5), *β_1_* and *β_2_* are coefficients and the 2 was added to avoid negative temperature values. The value of the coefficients was calculated by least square linear regression analysis for each of the 119 sites. The daily average *Temp_site_* was then calculated for each of the sites by using the average coefficients (N = 119), *Temp_smhi_* and *EXP* according to the linear model. Linear regression analysis between the daily average *Temp_site_* and the measured *Temp_site_* indicated a highly significant relationship (P<2.2·10^−16^, R^2^ = 0.42). This indicates that the calculated values are reasonable estimates of the daily average temperatures at the sampling sites.

### Biotic data

For each sample, diatom richness was calculated as the number of diatom taxa in 1000 valves as a measure of taxonomic diversity. This varied from 31 to 81 in the temperature gradient and from 18 to 99 in the salinity gradient. Further biotic variables used in the temperature data set are the microphytobenthic biomass measured as ash-free dry weight (MPB: range in the dataset 3–43 g m^−2^), the cover of macroalgae on an ordinal scale (Maccov, Table S5) and invertebrate macrofauna >1 mm (Fauna: 16–31,000 individuals m^−2^) [37], [38]. Further biotic variables used in the salinity data set (Figure 4) are total ash-free dry weight on the stones (ADW: 0.4–8.6 mg cm^−2^) and the percentage ADW of the DW (ADW%: 7–62%) [34]. The ADW% is assumed to be high for diatom-poor algal communities (containing more macroalgae) and low for diatom-rich communities (containing less macroalgae). The reason for this is that silicate, the main component of the diatom frustules, persists after combustion at 550°C.

### Assignment of traits to the diatom taxa

Altogether 405 diatom taxa are included in this study (Table S6). Four variables for cell size were defined for each taxon as (1) Length  =  the average length of the largest cell dimension, (2) Surface  =  the average cell surface area, (3) Volume  =  the average cell volume, and (4) S:V  =  the average surface to volume ratio. To describe major differences in cell shape between thin needle-like taxa and disk-shaped to nearly spheric-shaped taxa, we defined the parameter “Shape” as the aspect ratio of the largest cell dimension divided by the second-largest cell dimension. The cell size and shape data for 352 taxa were obtained from [27], and those for the additional 53 taxa were based on unpublished measurements (PS) from diatom slides. Life-form data for 290 of the taxa were obtained from [39]–[43] while those for the other 115 taxa were based on [24] or unpublished observations (PS) from living diatoms in the Baltic Sea.

Some diatom taxa typically form colonies, others do not [24]. In this study we distinguish between the coloniality traits “Colonial” or “Solitary” and how far the cells are able to extend away from the substratum. This latter category (“Growth height”) was subdivided into three groups: (1) “High”  =  large attached colonies visible to the naked eye in the field (up to ∼50 cm high) with cells in mucilage tubes or long chains, or in bushes on long branched mucilage stalks, (2) “Medium”  =  medium-sized colonies of ∼10 to 50 cells and/or cells elevated from the substratum on mucilage stalks, and (3) “Low”  =  solitary cells or small colonies (generally <10 cells), which are motile, adnate or attached with pads.

We subdivided the 405 diatom taxa into four mobility trait groups, which are not related to the possession of a raphe, e.g. some species with a raphe occur mainly attached to long stalks (*Cymbella* spp.). These four groups are: (1) Attached  =  non-motile species, attached to a substratum available on the stones, e.g. stone surface, sand grains, macroalgae, other diatoms and fauna, (2) Creeping  =  adnate species that are basically non-motile, but may move very slowly on their substratum, e.g. *Cocconeis* spp., (3) Motile  =  species that may move relatively fast over a substratum, e.g. *Navicula* spp., and (4) Floating  =  non-motile species that are basically pelagic, but may occur in the benthic community.

### Tests for spatial and temporal autocorrelation

Parts of the temperature gradient in Forsmark have a unidirectional water flow, which potentially can cause spatial autocorrelation in the diatom data. We therefore applied asymmetric eigenvector mapping (AEM) to check for directional spatial autocorrelation [44] between sites for all sampled dates. The AEM analyses were performed by first constructing a flow chart describing how the different sites in and outside the Biotest basin are connected to each other. All sites were included in this analysis except Site G, which was used as a zero-node, and Site D, which at the time of data collection had no direct connection to Site G. An edge matrix [45] was constructed, which contained information on the direction of the water flow and the connections between sites. Sites directly connected to each other were represented by values 1 and the others by 0. The distances between sites were transformed to similarities and used as weights to the columns in the edge-matrix. From the edge matrix we extracted the eigenvectors describing positive spatial autocorrelation by calculating Moran's I. The ability of these eigenvectors to significantly describe positive spatial autocorrelation was tested by performing a global test using all eigenvectors as predictor variables for species composition in a redundancy analysis (RDA).

AEM analysis was also used to check for temporal autocorrelation between sampled dates for each site in the Biotest basin. Temporal autocorrelation is similar to a unidirectional one-dimensional stream where sites downstream can only be affected by sites upstream and not the other way around [46]. As weights in the edge matrix we used days between sampling. The significance of temporal autocorrelation was tested in the same way as that for spatial autocorrelation.

In the salinity gradient we tested for spatial autocorrelation within each area. This was performed with Moran's eigenvector map (MEM) [47]. In the MEM analysis we identified the shortest water distance between all sampled sites. A minimum spanning tree was constructed based on the distances between the sites. From this, a connectivity matrix was constructed by connecting sites between which the distance was equal to or shorter than the longest distance that keeps all sites connected, the threshold value of the minimum spanning tree. Sites directly connected to each other were represented by values 1 and the others by 0. The connection matrix was combined with a distance matrix through the Hadamard product [48]. From the resulting matrix, eigenvectors describing positive spatial autocorrelation were extracted and tested for spatial autocorrelation in the same way as for the Forsmark area.

The AEM and PCNM packages in the statistical software R (Version 2.15.1, R Foundation for Statistical Computing) were used for the spatial and temporal autocorrelation analyses.

### Fourth-corner analysis and other statistics

Fourth-corner analysis [49], [50] was used to test the relationship between the environmental variables and the species traits. This method applies three different data matrix tables: an **L** (m*k) table with *k* species abundances at *m* sites, an **R** (m*p) table with *p* environmental variables at *m* sites and finally a **Q** (k*n) table with *n* traits for *k* species in table **L**. To estimate the correlation between traits and the environment a so-called fourth-corner statistic is calculated via matrix algebra on the three data matrices. This statistic is equivalent of Pearson product-moment correlation coefficient (r_P_). The significance of the fourth-corner statistic was tested by randomly permuting rows and columns of the **L** table and after each permutation a new fourth-corner statistic was calculated. This operation was repeated 9999 times to obtain a distribution for the randomly permuted statistic.

Finally, the fourth-corner statistic was compared to the randomly permuted distribution of statistics, in order to obtain the p-value. The row and columns of the **L** table can be permuted in different ways depending on the hypothesis tested. In this study we used a combination by first permuting the entire rows (the site vectors) of table **L** and then permuting the entire columns (the species vector) of table **L**. The reason for this approach is two-fold; it controls the type I error and the combination of the two permutation tests is the only way to properly test the relationship between species traits and environmental variables [50], [51]. In our analyses we use the fourth-corner analysis to explore association between different environmental variables and different traits. We do not use it to reject a global null hypothesis that there is no environment-trait correlation at all, as this would require some type of multiple test correction.

Linear and factorial multiple regression analyses (GLM) were used to explore responses of traits to environmental variables. The covariation between environmental and biotic variables was summarized in a principal components analysis (PCA). Spearman rank correlations (r_s_) were used to evaluate the significance of covariation between environmental variables and between quantitative size traits. When r_s_ values are given in the text, they are significant at P<0.05 using Holm's multiple test correction.

The statistical software R (Version 2.15.1) was used for the fourth-corner analyses (ade4 package) and the calculations of r_s_, PCA and GLM were performed with the statistical software Statistica (Version 12).

## Results

### Spatial and temporal autocorrelation

There was no spatial autocorrelation between sites in the temperature gradient for any of the sampled dates (Table S7). However, temporal autocorrelation within sites was recorded for all sites except two (Sites B and H; Table S7). Temporal autocorrelation was expected to occur because the samples were taken at regular intervals during the season and all sites were strongly influenced by seasonal variation in temperature, solar irradiation and inorganic nutrient concentrations. We did not correct for this temporal autocorrelation, which may lead to an increased type I error in the fourth-corner analysis. Therefore significant correlations close to the P = 0.05 significance limit should be interpreted with some caution.

In the salinity gradient, all areas except one (Rånefjärden) showed no spatial autocorrelation within the areas (Table S7). Rånefjärden was still included in the analysis because our main hypothesis for the salinity gradient concerns a process of which the influence is larger than that of any one of the sampled areas separately.

### Correlations between cell size and shape traits

The relationships between the cell size and shape traits were basically the same in both gradients (Tables S8 and S9). Cell length, surface and volume were positively correlated to each other (r_s_  = 0.83 to 0.99) and negatively to S:V (r_s_  =  −0.62 to −0.95). The shape parameter was correlated to the length (r_s_  = 0.51), weakly to surface and S:V (r_s_  = 0.15 to 0.17) and not to volume (P>0.05).

### Coloniality and mobility traits

Solitary diatom taxa made up 71–72% of the taxa in the two data sets (Table 1). However, valves belonging to colonial taxa were more abundant and dominated the data sets in terms of cell numbers (58–66%). This was mainly due to the 14–15% of the taxa that were able to form macroscopic colonies. Motile diatom taxa made up 56–58% of the taxa in the two data sets, but in terms of cell numbers attached taxa were about equally abundant (41%) as motile taxa (44%) in the temperature gradient. In the salinity gradient the abundance of attached taxa (60%) was almost twice that of motile taxa (32%).

**Table 1:** Percentages of the number of taxa and of the total abundance (of 1000 valves) assigned to the different categorical diatom traits in the two data sets.

		Temperature gradient		Salinity gradient	
Trait group	Trait	% Taxa	% Abundance	% Taxa	% Abundance
Coloniality	Solitary	72	42	71	34
	Colony	28	58	29	66
Growth height	Low	70	50	72	34
	Medium	16	16	14	24
	High	14	34	15	43
Mobility	Attached	19	41	21	60
	Motile	58	44	56	32
	Creeping	10	4	12	4
	Floating	13	11	11	4

### Correlations between variables in the temperature gradient

**Figure 5:**
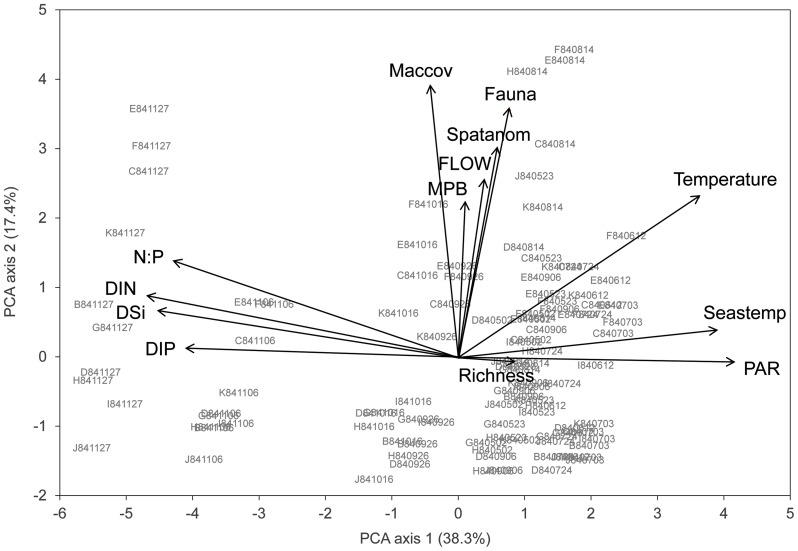
Results of the principal component analysis (PCA) of environmental and biotic variables in the temperature gradient. The ordination plot summarizes the variation of the environmental and biotic variables across the 121 samples (indicated by site and date) included in the fourth-corner analysis of the temperature gradient. Temperature  =  surface water temperature at each sampling site, Seastemp  =  surface water temperature at Site G, Spatanom  =  spatial surface water temperature anomaly, PAR  =  daily average solar irradiation (photosynthetically active radiation) at the water surface, DIN  =  dissolved inorganic nitrogen, DIP  =  dissolved inorganic phosphorus, DSi  =  dissolved inorganic silicate, N:P  =  molar DIN:DIP ratio, FLOW  =  water movement flow factor cf. Table S5, MPB  =  microphytobenthic biomass, Maccov  =  macroalgal cover cf. Table S5, Fauna  =  invertebrate macrofauna density, Richness  =  number of diatom taxa in 1000 valves.

In the PCA of the environmental variables in the temperature gradient, Seastemp, PAR, DIN, DIP, DSi and N:P were mainly associated with the first component (Figure 5). The nutrient concentrations were all strongly positively correlated with each other (r_s_  = 0.80 to 0.83) and N:P was more related to DIN (r_s_  = 0.95) than to DIP (r_s_  = 0.57) (Table S10). Solar irradiation (PAR) was negatively correlated to the four nutrient variables (r_s_  =  −0.71 to −0.82), following the normal seasonal variation in the Baltic Sea with high nutrient concentrations in the cold season and low ones in summer. The variables Spatanom and FLOW were in the PCA mainly associated with the second component. These two spatial variables were positively correlated to each other (r_s_  = 0.45) as the site-specific temperature anomaly was higher with increased influence of the cooling water flow. The water temperature measured during sampling was correlated to Seastemp, Spatanom and PAR (r_s_  = 0.74, 0.56 and 0.43, respectively) and also weakly to the nutrient variables (r_s_  =  −0.21 to −0.31). In the PCA, water temperature was about equally associated with the first (seasonal variation) and second (spatial variation) component as it is a combination of Seastemp and Spatanom.

Although the macroalgal cover, microphytobenthic biomass and invertebrate density were associated with Spatanom and FLOW in the PCA (Figure 5), correlations between biotic and environmental variables in the temperature gradient were not significant except for a positive correlation between water temperature and invertebrate density (Table S10). The microphytobenthic biomass and the density of invertebrates increased with increasing macroalgal cover (r_s_  = 0.36 and 0.65, respectively), while diatom richness decreased with increasing macroalgal cover (r_s_  =  −0.26). Richness was positively correlated to Seastemp (r_s_  = 0.34), but not to Spatanom, indicating that the highest diatom richness occurred in summer.

### Correlations between variables in the salinity gradient

**Figure 6:**
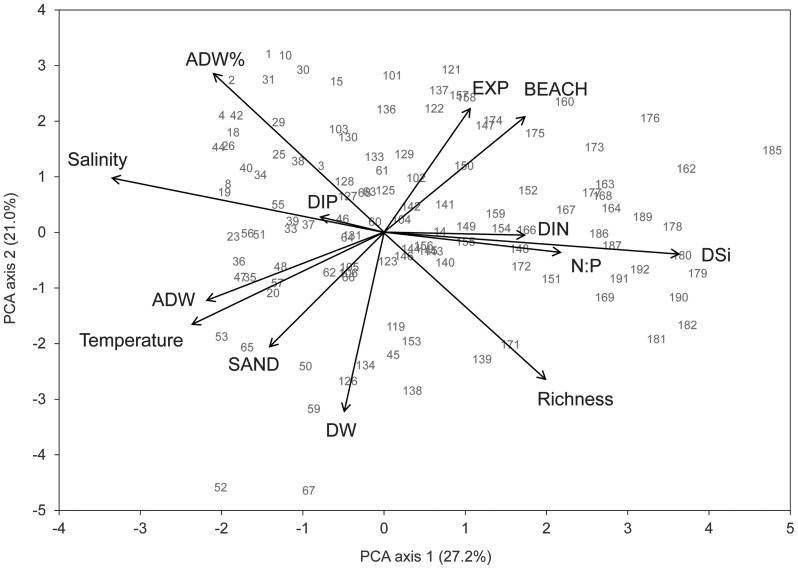
Results of the principal component analysis (PCA) of environmental and biotic variables in the salinity gradient. The ordination plot summarizes the variation of the environmental and biotic variables across the 119 samples (indicated by site number) included in the fourth-corner analysis of the salinity gradient. Salinity  =  surface water salinity at each sampling site, Temperature  =  surface water temperature at each sampling site, DIN  =  dissolved inorganic nitrogen, DIP  =  dissolved inorganic phosphorus, DSi  =  dissolved inorganic silicate, N:P  =  molar DIN:DIP ratio, EXP  =  exposure to waves cf. Table S5, BEACH  =  beach type cf. Table S5, SAND  =  ash weight of sand grains on the stones, ADW  =  total ash-free dry weight on the stones, ADW%  =  % ash-free dry weight (a measure of the relative amount of macroalgae on the stones), Richness  =  number of diatom taxa in 1000 valves.

In the PCA of the environmental variables, salinity, nutrient variables and biomass (ADW) were associated with the first component while the other variables were associated with both the first and second components (Figure 6). Salinity was positively correlated to water temperature (r_s_  = 0.59) and DIP (r_s_  = 0.33), but negatively to DIN, DSi and N:P (r_s_  =  −0.73, −0.89 and −0.61, respectively) (Table S11). The N:P ratio was positively correlated to DIN (r_s_  = 0.63) and negatively to DIP (r_s_  =  −0.83). With exposure to wave action, rocky-shore beaches increased (r_s_  = 0.53) and consequently sandy beaches decreased and less sand occurred on the stones (r_s_  =  −0.46).

In the salinity gradient, the abundance of macroalgae (ADW%) was positively correlated with salinity (r_s_  =  0.49) and negatively with diatom richness (r_s_  =  −0.60). This indicates that there were more macroalgae on the stones in the south than in the north of the Baltic Sea and that diatom richness decreased when epiphytes and other diatoms associated with macroalgae were abundant.

### Trait-environment associations in the temperature gradient

**Figure 7:**
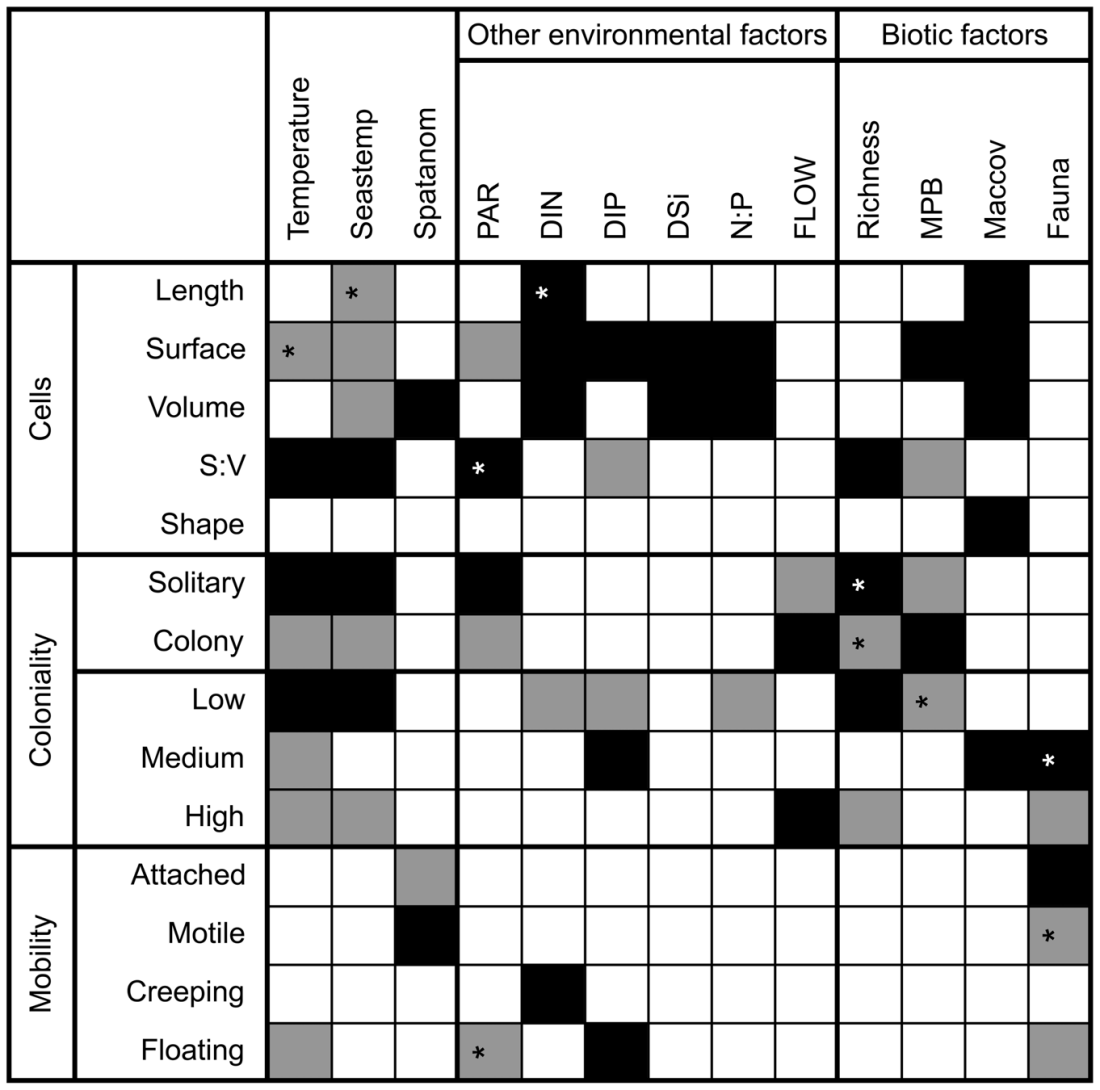
Result of the fourth-corner analysis for the temperature gradient. Black fields represent significant (P<0.05) positive correlations, grey fields represent significant negative correlations. Stars indicate P-values between 0.04 and 0.05. Abbreviations cf. Figure 5.

The fourth-corner analysis of the temperature gradient data displayed 62 significant correlations between 14 traits and 13 environmental or biotic variables (Figure 7). This is more than would be statistically expected from 182 random correlations (14 traits * 13 environmental variables or biotic variables), which should result in 9 significant correlations under the 5% error level when assuming random data.

Among the cell-size variables, positive associations were found between S:V and temperature, Seastemp, PAR and diatom richness, suggesting that smaller diatoms in high-diversity communities prevailed in the warm season, including at the heated sites in the warm season. Positive associations were also found between cell size (volume) and Spatanom, DIN, DSi, N:P and Maccov, indicating that diatom cell size was larger with artificial warming, higher nutrient concentrations and higher overall biomass on the stones. Shape was only correlated with Maccov, which confirms that diatom epiphytes in general are more needle-like in shape than other epilithic diatoms.

A colonial life form was negatively associated with temperature, Seastemp, PAR and richness and positively with FLOW and MPB. This shows that colonial diatoms were more abundant in the cold season when they formed high-biomass low-diversity communities at stations with strong water movement. The same trend was observed in the environmental associations with the traits related to three-dimensional growth.

The warming variable (Spatanom) was not associated with any coloniality trait, but it was positively associated with motility. This indicates that motile diatoms were selected by warming. The opposite was found for grazing pressure (macrofaunal density); with high grazing pressure attached diatoms increased and possibly motile diatoms were preferred as food items. The occurrence of pelagic diatom valves in the benthic communities was highest in the cold season, which may indicate that these valves originated from dead or decaying cells that had been sinking to the bottom even at the shallow depth at which the samples were taken (0.2–0.7 m).

### Trait-environment associations in the salinity gradient

**Figure 8:**
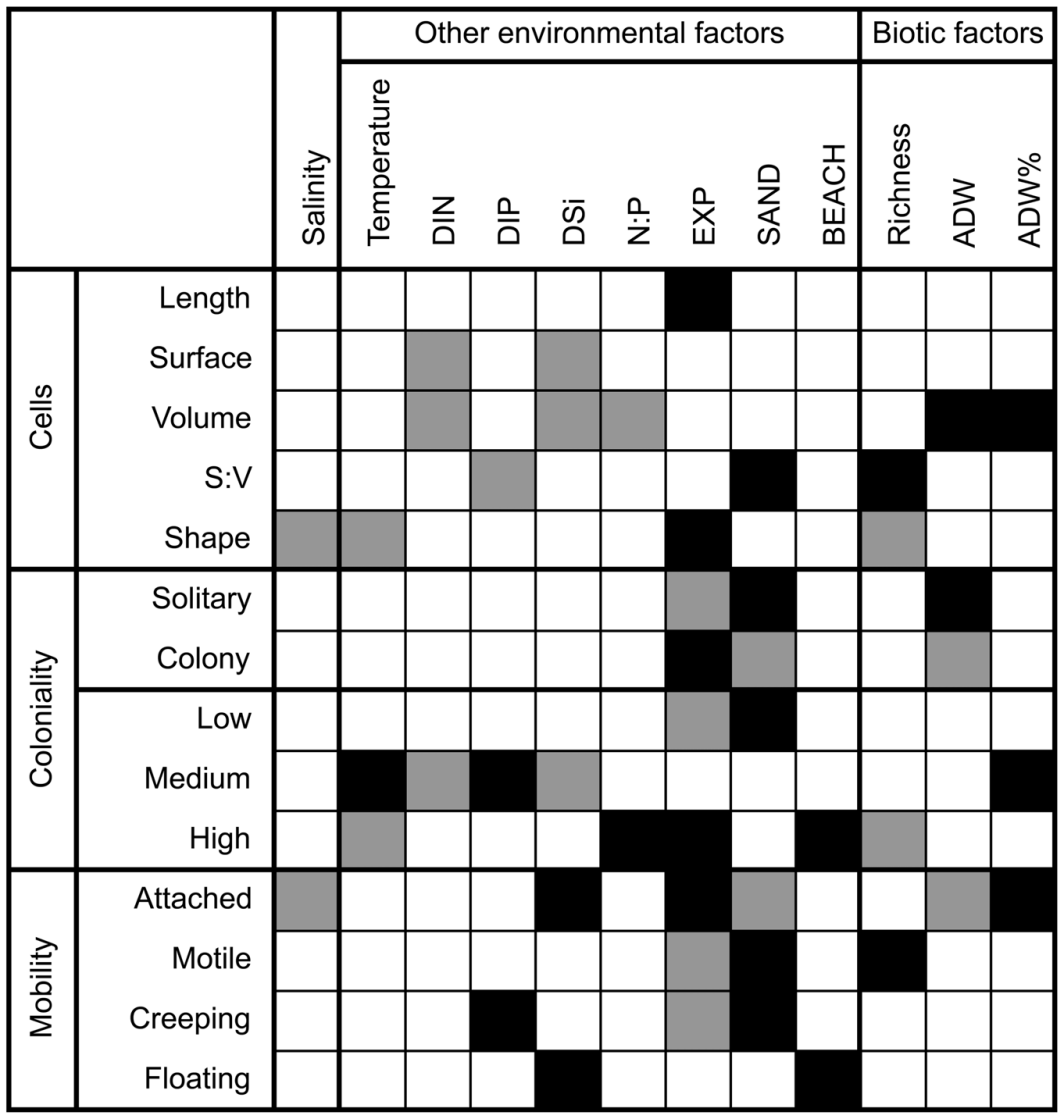
Result of the fourth-corner analysis for the salinity gradient. Black fields represent significant positive correlations, grey fields represent significant negative correlations. Abbreviations cf. Figure 6.

The fourth-corner analysis of the salinity gradient data displayed 48 significant correlations between 14 traits and 12 environmental or biotic variables (Figure 8). This is more than would be statistically expected from 168 random correlations (14 traits * 12 environmental variables or biotic variables), which should result in 8 significant correlations under the 5% error level when assuming random data.

None of the cell size-related diatom traits was associated with salinity, nor did temperature select for cell size in this data set. Like in the temperature data set, DIP was negatively correlated to S:V. However, in contrast with the temperature data set, DIN and DSi were negatively correlated to cell surface and volume (Figure 8). Salinity was negatively associated with attached and needle-shaped cells and temperature was not associated with any of the other three mobility life forms. In this data set the mobility life forms were strongly related to water movement, with positive selection for attached taxa at sites that with strong exposure to wave action where more macroalgae and less sand grains occurred on the stones.

Macroscopic diatom colonies were more abundant at low temperature when they formed low-diversity communities at stations with strong water movement. This is the same result as in the temperature data set.

### Quantification of cell size decrease

**Figure 9:**
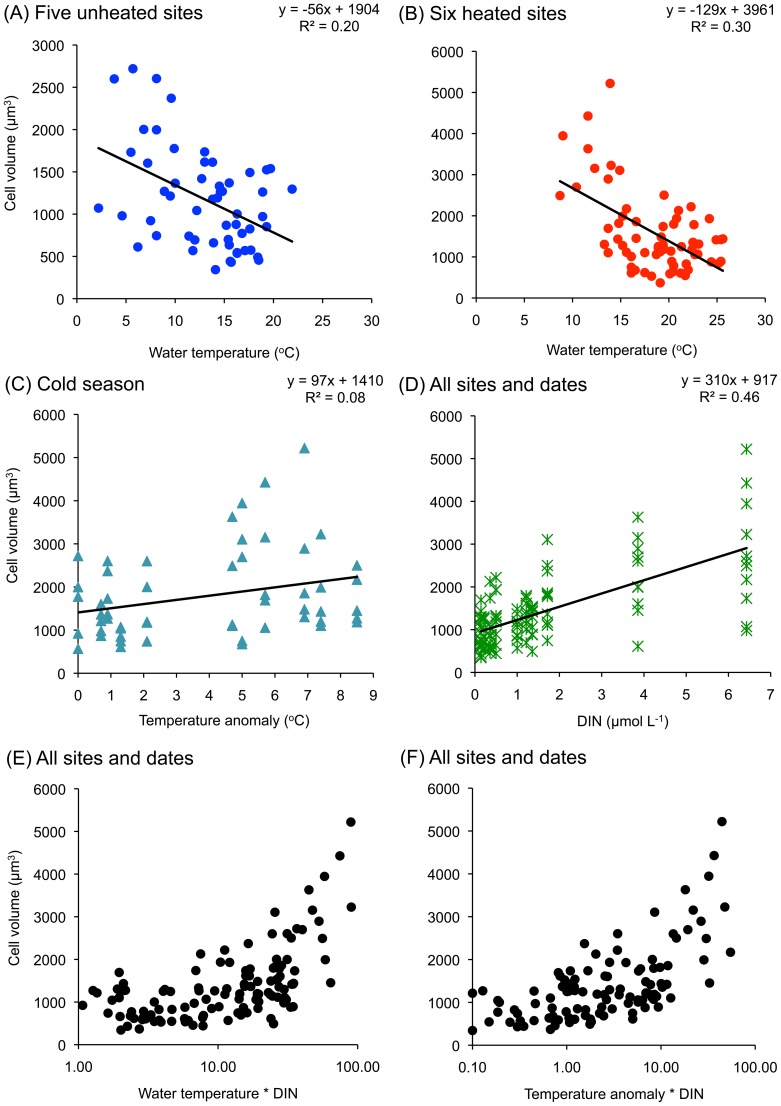
Average community cell volume plotted against the variables used in linear and multiple regression analyses for the temperature gradient (a) Water temperature at the five unheated sites. (B) Water temperature at the six heated sites. (C) The temperature anomaly during the cold season (when the reference temperature at Site G was below 12°C). (D) Dissolved inorganic nitrogen (DIN). (E) Water temperature * DIN. (F) Temperature anomaly * DIN. Data extrapolated from Tables S1 and S6.

To quantify the inverse relationship between water temperature and cell size obtained in the fourth-corner analysis (Figure 7), we performed linear regression analyses (Table 2, Figure 9). In a regression model for the whole data set, average community cell volume (sum of the cell volumes of the 1000 taxa counted divided by 1000) decreased linearly with 2.2% per °C. This relationship was similar when the five unheated sites and the six heated sites were analyzed separately, yielding 2.9% and 3.3% average community cell volume decreases per °C, respectively. The intercept, which represents the average community cell volume at the lowest temperature, was at the heated sites 3961 µm^3^ but at the unheated sites this was less than half of this (1904 µm^3^). The regression coefficient, representing the average rate of cell volume decrease, was also much larger at the heated sites (−129 µm^3^ per °C) than at the unheated sites (−56 µm^3^ per °C). However, the R^2^ values were quite low, suggesting that other environmental and biotic variation interfered as well.

**Table 2:** Results of linear regression analyses with average community cell volume as response variable.

Data set	Predictor variable	N	Range of predictor variable	Range of average cell volume (µm^3^)	P-value	R^2^	Intercept ± SE	Coefficient ± SE	% change per unit predictor variable
Temperature	Temp	121	2–26°C	344–5219	0.0018	0.08	2133±243	−46±14	−2.2
Temperature	Temp (unheated sites)	55	2–22°C	344–2720	0.0006	0.20	1904±213	−56±15	−2.9
Temperature	Temp (heated sites)	66	9–26°C	368–5219	<0.0001	0.30	3961±462	−129±24	−3.3
Temperature	Spatanom	121	0–8.5°C	344–5219	0.0240	0.04	1159±128	60±26	5.2
Temperature	Spatanom (warm season)	66	0–8.5°C	344–2219	0.1535	-	-	-	-
Temperature	Spatanom (cold season)	55	0–8.5°C	569–5219	0.0392	0.08	1410±224	97±46	6.9
Temperature	DIN	121	0.1–6.4 µmol L^−1^	344–5219	<0.001	0.46	917±73	310±30	34
Salinity	Salinity	119	0.5–7.8	186–4859	0.0003	0.11	331±137	105±28	32
Salinity	Temperature	119	1–10°C	186–4859	0.3254	-	-	-	-
Salinity	DIN	119	0.2–7.4 µmol L^−1^	186–4859	0.0103	0.05	937±70	−73±28	−7.8

Warm season  =  Reference temperature at Site G above 12°C in June, July, August and September, Cold season  =  Reference temperature at Site G below 12°C in May, October and November, N =  number of samples.

The concentrations of DIN, DIP and DSi were strongly correlated to each other (Table S10), but in the fourth-corner analysis DIN showed most associations with the cell size variables (Figure 7). A factorial multiple regression analysis showed that the effects of temperature and DIN on average community cell volume per sample were not significant, but that there was a strong combination effect of temperature and DIN (Table 3, Figure 9).

**Table 3:** Results of two factorial multiple regression analyses with average community cell volume per sample as response variable.

N	R^2^	Effect	Coefficient ± SE	Sum of squares	F-value	P-value
121	0.55	Intercept	1119±284	5125035	15.6	0.0001
		Temperature	−20±16	508711	1.5	0.2162
		DIN	54±76	161618	0.5	0.4848
		Temperature * DIN	27±6	6171106	18.8	<0.0001
121	0.55	Intercept	872±115	19155683	57.4	<0.0001
		Spatanom	11±24	78974	0.2	0.6274
		DIN	186±48	5075928	15.2	0.0002
		Spatanom * DIN	31±10	3433983	10.3	0.0017

N =  number of samples. Temperature  =  water temperature at each sampling site, DIN  =  concentration of dissolved inorganic nitrogen in the water. (Data from Table S1).

Linear regression analysis showed that the average community cell volume increased by 5.2% per °C of artificial warming of the seawater (Table 2). This effect was restricted to the cold season, defined as a reference temperature at Site G of <12°C in May, October and November 1984 (Figure 9C). This finding is opposite to what one would expect according to the relationship between the average community cell volume and increasing temperature described above. A factorial multiple regression showed that DIN (which co-varied with DIP and DSi) was involved also here. While the effect of Spatanom was not significant, both DIN and Spatanom*DIN influenced the average cell size per sample (Table 3, Figure 9).

**Figure 10:**
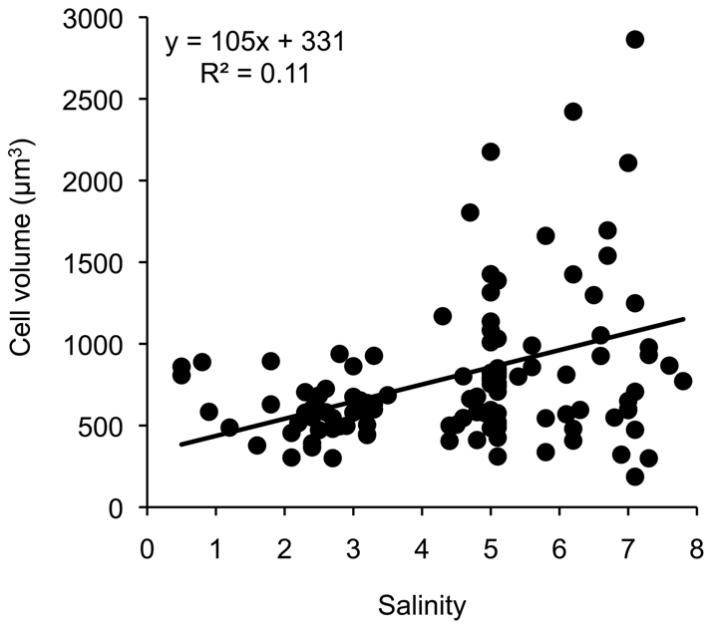
The relationship between average community cell volume and salinity along the salinity gradient. Data extrapolated from Tables S3 and S6.

Despite that no significant associations were found between cell size and salinity in the fourth-corner analysis (Figure 8), there was a significant linear increase in the average community cell volume along the salinity gradient, with an increase of 32% per unit salinity increase (Table 2, Figure 10). Temperature had no effect on the average community cell volume in the salinity gradient (Table 2).

## Discussion

### Cell size: a combined result of temperature and nutrients

The linear cell-size decrease of 2.2% per °C we recorded in the natural diatom communities is similar to the linear cell-size decrease of 2.5% per °C found in a meta-analysis of single-species cultures of protists [52]. These results are in agreement with the “temperature-size rule”, a phenotypically plastic response of body size to rearing temperature, which is a ubiquitous intraspecific phenomenon in most ectotherms [53], [54]. Selection for smaller-sized species with increased temperature may be related to a faster turnover time, which favors r-selected species [55]. Decreases in average community cell size with increased temperature have also been recorded for phytoplankton, and similar to our findings such a decrease is usually coupled to nutrient dynamics [56]. In phytoplankton, cell size depends on trade-offs with respect to growth rates and sinking rates, involving processes like nutrient uptake, light harvesting and self-shading in relation to the chemical and physical environment. Thus, the effect of climate change on phytoplankton community size structure may vary across aquatic ecosystems. Increased stratification and reduced nitrogen to phosphorus ratios in Lake Tahoe (USA) between 1982 and 2006 selected for small-celled diatoms with low sinking rates [23]. In contrast, in shallow Canadian lakes pelagic diatoms decreased in size with higher nutrient availability [57]. Obviously, in deep lakes with strong stratification and low nutrient fluxes, it is beneficial for a diatom to be small to maximize its surface to volume ratio for nutrient uptake and to minimize sinking rates [58]. On the contrary, in shallow lakes with high nutrient availability and low transparency it is beneficial for a diatom to be small to maximize its surface area to reach a high photosynthetic performance while at the same time the effect of self-shading is reduced [59].

In our case, 55% of the variation in the average community cell volume along the whole temperature gradient (2–26°C) was explained by the multiple regression model, in which a significant combination effect of temperature and DIN was found. This translates to the simple model that cells grow larger at low temperature/high nutrient supply while small cells are found at high temperature/low nutrient supply. However, it cannot be excluded that other processes like light harvesting, self-shading and selective grazing also play a role in the trade-offs that determine cell size in benthic diatom communities.

### Cell size: a combined result of salinity and nutrients

Freshwater diatoms are generally significantly smaller than marine diatoms, which has been explained by trade-offs between size and DIP vs. DIN limitation in fresh- and marine waters, respectively [12]. Since there is an abrupt shift from dominance of marine species to dominance of freshwater species in the Baltic Sea benthic diatom communities between salinity 5 and 6 [60], we expected cell size to be smaller in the north than in the south of our sampling area (Figure 2). While there was a significant linear decrease in the average community cell volume with decreasing salinity in the regression analysis as expected [12], we found no correlation between cell volume and salinity in the fourth-corner analysis. However, in our data set DIP was positively correlated to salinity while DIN and N:P were negatively related to salinity. This change in the N:P ratio with salinity indicates DIP as the limiting nutrient for primary production in the northern, more freshwater-influenced part of the Baltic Sea and DIN as the limiting nutrient in the southern, more marine-influenced part [61]. The fourth corner analysis showed that cell volume was smaller at higher N:P ratios (DIP-limitation) in the north of the Baltic Sea than with DIN-limitation in the south. All these results point in the same direction: diatom size decreases with decreasing salinity, apparently mediated by nutrient stoichiometry. This suggests that a future decrease in the surface salinity of the Baltic Sea by increased land-runoff would lead to a decrease in the average cell size of the benthic diatom community.

### Warming polarizes seasonality

In our large temperature experiment at full natural scale it was possible to separate the warming effect (Spatanom) from a general temperature effect since part of the long temperature gradient (2–26°C) in Forsmark was artificially heated by cooling water. This implies that the normally covarying seasonal temperature, irradiation and nutrient cycles are disentangled. The Spatanom variable was not correlated to PAR or any of the nutrient variables in our dataset. This set-up created two new niches, the same niches that are being established by the ongoing climate change. The first niche is higher temperature than normal at high irradiation and low nutrient concentrations in the warm season. The second niche is higher temperature than normal at low irradiation and high nutrient concentrations in the cold season.

Our results show that warming in the warm season (June – September) has no effect on cell size other than the “normal” cell size decrease with temperature, here quantified to 3.3% per °C. Since summer temperature is predicted to rise with climate change [1], it is thus expected that the benthic diatom communities at the end of this century will consist of smaller cells with faster turnover rates and lower biomass, similar to predictions for marine phytoplankton [62].

Our study also shows that warming in the cold season (May, October and November) causes a considerable increase in cell size with increasing temperature anomaly. This second new niche extends into the normally ice-covered period in the northern Baltic Sea. A comparison of benthic spring bloom dynamics over six years in Forsmark in the 1980′s showed that large blooms of colonial diatoms occupy this niche when the ice cover decreases or disappears [63]. This latter study identified the absence of an ice cover as the crucial factor for increased biomass in winter. However, to be able to compare the diatom traits from all sampling sites at all sampling dates in our study we excluded the ice-covered period December – April (no data were available for the ice-covered sites B,D,H and J during this time). Nevertheless, we observed a large increase in cell size with warming in the cold season. This may at first sight seem contradictory to the previously described linear size decrease with temperature, but they are in agreement when it is considered that we also showed that nutrient supply is a major regulating factor, in combination with Spatanom.

While studies on phytoplankton have suggested that nutrient limitation decreases cell size with warming [23], [56], we here show that warming also can increase cell size when nutrients are in excess and temperature is not too high. Seemingly contradictory results to our observations in the cold season have also been published [16], [64], although these publications refer to mesocosm studies, which were seeded with natural communities and then manipulated, and are not fully comparable with our study at full natural scale. For example, the temperature ranges were narrower than in our study and nutrients were not in focus.

Since winter temperature is predicted to rise with climate change [1], it is thus expected that the winter protist communities at the end of this century will consist of larger cells with slower turnover rates and higher biomass. Altogether, our results suggest that climate change in this century may polarize seasonality by elevated temperature at high nutrient concentrations (larger cells and higher community biomass) in the cold season and elevated temperature at low nutrient concentrations (smaller cells and lower community biomass) in the warm season.

### The importance of nutrients

Nutrient concentrations and nutrient limitation played key roles in all changes in the diatom cell size spectra we documented in this paper. This brings us back to the question if size reduction in benthic diatom communities (and protist communities in general) is caused by temperature or salinity on their own, or if other factors interfere as well. Our answer to this question is that the size reductions in nature probably are combination effects between temperature or salinity and other factors, presumably in many cases nutrient variables. In a study on natural benthic diatom communities in geothermally heated freshwater streams on Iceland, no evidence was found that temperature would have any effect on diatom cell size [65]. It was concluded that diatoms may be an important exception to temperature-size rules at species and community levels of organization. This is truly contradictory to what we found and, based on our results presented here, we do not agree with their general conclusion. A possible explanation for the disagreement with our result is that in the Icelandic freshwater streams algal production was (co)limited by nitrogen and phosphorus and there was very little variation in nutrients across the catchment, i.e. nutrient concentrations were not strongly related to temperature [65]. This supports the hypothesis that size reduction in protist communities is strongly mediated by nutrients. However, in other systems this can be light regimes [57], [66] or selective grazing [14].

### Motility and coloniality

We found that warming and not nutrient availability promoted a motile life form while no selection for motility occurred with the seasonal temperature variation. Previous studies, both in mesocosms and in field studies, have tied a motile life form to an increase in nutrient supply, arguing that a motile life form would be more competitive in a resource-rich environment [25], [67]. Our results suggest that warming may have similar effects on motility as increased nutrient availability. The reason why warming would promote mobility in diatoms may be traced to changes in the mechanism of locomotion in motile diatoms with changes in temperature. The viscosity of the cytoplasm in the raphe has been shown to decrease with increased temperature, thereby making the motile diatoms able to move faster at higher temperature [68]. Warming may therefore further increase the competitive advantage of motile diatoms over attached diatoms.

We recorded strong effects of temperature and light, but not of nutrient supply, on coloniality. Colony-forming diatoms dominated in our temperature dataset during the colder and darker period while solitary diatoms dominated the warmer and lighter periods of the season. In general, light availability has a strong influence of diatom community structure [69]. Colonial diatoms have been associated with high-light and nutrient-rich conditions [25], [67]. Our result is therefore unexpected given that we found colonial life forms to dominate under low light conditions and not under high light conditions. This suggests that temperature in combination with light may influence the dominance of solitary vs. colony-forming species in the diatom community. In the salinity gradient there were few signs that salinity or nutrients correlated to salinity selected for any particular life form. Instead, as expected, the local physical variables exposure and sand selected for contrasting life forms both in coloniality and motility, in the salinity gradient.

### Implications with climate change

Based on the results presented in this paper, the cell size and biomass of the epilithic diatom communities are expected to decrease in summer and increase during the cold season with the increasing sea-surface temperatures and decreasing sea-surface salinity predicted by the regional climate scenarios for the brackish Baltic Sea area until the end of this century [2], [3]. Both in summer and winter, nutrient supply is an additional key factor that adds to this polarization of seasonality. Aquatic food-webs are to a large extent size-structured; any changes in size of the primary producers may therefore have an impact on the flow of energy to higher trophic levels [70]. Food-webs composed of larger primary producers have a more efficient transfer of energy to higher trophic levels than a food-web dominated by smaller primary producers [71], [72]. The food web in the shallow benthic zone of aquatic ecosystems is to a lesser degree size-structured than in the pelagic zone. Still, parts of the grazer community in the shallow benthic zone are known to display size-elective feeding [14]. Warming may displace part of the annual total rocky-shore biomass production towards the colder, more nutrient-rich, part of the year (eutrophication), while the warmest part of the year will see a decline in biomass production.

## Supporting Information

Table S1 **Dataset “Temperature” with 121 site/dates: Environmental and biotic variables.** Abbreviations cf. Figure 5.
(PDF)

Table S2 **Dataset “Temperature” with 121 site/dates and 230 diatom taxa: 1000 valves/sample.**
(PDF)

Table S3 **Dataset “Salinity” with 119 sites: Environmental and biotic variables.** Abbreviations cf. Figure 6.
(PDF)

Table S4 **Dataset “Salinity” with 119 sites and 355 diatom taxa: 1000 valves/sample.**
(PDF)

Table S5 **Ordinal scales used to estimate the magnitude of some environmental and biotic variables in the temperature and salinity gradients.**
(PDF)

Table S6 **List of the 405 diatom taxa included in the analyses.** BMB nr  =  number under which photomicrographs and ecological information can be found in publications 39–43. Temp  =  temperature gradient data set, Salin  =  Salinity gradient data set, Freq  =  average frequency in 1000 valves, Abund  =  average abundance in 1000 valves, Salinity group  =  distribution in the Baltic Sea area (M =  marine, B =  brackish, F =  freshwater), Euryhal group  =  Euryhalinity according to the species' salinity range in the Baltic Sea area after [39]–[43] (M =  medium, L =  large, XL  =  very large, XXL  =  extremely large). For further definitions of traits, see “Assignment of traits to the diatom taxa” in the “Materials and Methods” section.
(PDF)

Table S7 **Result from the autocorrelation analyses in the temperature and salinity gradients, showing the P-values from the RDA analysis using eigenvectors describing positive spatial and temporal autocorrelation.**
(PDF)

Table S8 **Correlations between cell size and shape traits in the temperature gradient.** The lower triangle shows Spearman rank correlation coefficients, the upper triangle shows the adjusted P-values using Holm's method.
(PDF)

Table S9 **Correlations between cell size and shape traits in the salinity gradient.** The lower triangle shows Spearman rank correlation coefficients, the upper triangle shows the adjusted P-values using Holm's method.
(PDF)

Table S10 **Correlations between the environmental and biotic variables in the temperature gradient.** The lower triangle shows Spearman rank correlation coefficients, the upper triangle shows the adjusted P-values using Holm's method. Abbreviations cf. Figure 5.
(PDF)

Table S11 **Correlations between the environmental and biotic variables in the salinity gradient.** The lower triangle shows Spearman rank correlation coefficients, the upper triangle shows the adjusted P-values using Holm's method. Abbreviations cf. Figure 6.
(PDF)
