# Supplementary material for: Diatom Cell Size, Coloniality and Motility: Trade-Offs between Temperature, Salinity and Nutrient Supply with Climate Change
Source: PLoS One. 2014 Oct 3;9(10):e109993. doi: 10.1371/journal.pone.0109993 (PMC4184900; doi:10.1371/journal.pone.0109993)
Supplement: Table S1 — Dataset “Temperature” with 121 site/dates: Environmental and biotic variables. Abbreviations cf. Figure 5. (PDF) [file pone.0109993.s001.pdf]

Table S1. Dataset “Temperature” with 121 site/dates: Environmental and biotic variables. Abbreviations cf. Figure 5.

SITE	DATE	°C TEMP	°C TEMP G	°C ANOM	µmol/m^{2}s PAR	µmol/L DIN	µmol/L DIP	µmol/L DSi	mol/mol N:P	1-6 cale FLOW	of 1000 RICHN	g/m^2 MPB	1-9 scale MACCOV	Indiv/m^2 FAUNA
A	840502	16.5	7.5	5.0	455	0.14	0.06	6.00	2.21	5	47	43	3.5	620
A	840523	16.1	11.8	5.0	474	1.00	0.09	4.00	11.07	5	53	43	3.3	3277
A	840612	17.5	13.9	5.0	499	0.14	0.05	7.14	3.16	5	57	8	5.0	12128
A	840703	18.2	15.4	5.0	523	0.21	0.05	7.29	4.15	5	73	7	1.5	497
A	840724	16.1	16.2	5.0	477	0.36	0.13	8.43	2.77	5	73	15	5.0	1210
A	840814	24.4	17.6	5.0	432	1.36	0.10	10.21	14.02	5	69	36	8.0	20318
A	840906	22.4	14.5	5.0	222	0.50	0.09	8.43	5.74	5	68	30	8.3	7142
A	840926	22.6	12.7	5.0	70	1.21	0.17	9.07	6.97	5	75	16	3.8	1733
A	841016	14.9	9.9	5.0	117	1.71	0.09	8.50	18.98	5	58	31	9.0	8511
A	841106	10.4	8.1	5.0	82	3.86	0.24	19.43	16.16	5	55	15	3.0	28
A	841127	9.0	5.7	5.0	47	6.43	0.19	22.64	33.21	5	60	12	6.0	5549
B	840502	11.4	7.5	2.1	455	0.14	0.06	6.00	2.21	3	50	8	1.0	485
B	840523	14.3	11.8	2.1	474	1.00	0.09	4.00	11.07	3	44	10	0.5	478
B	840612	15.7	13.9	2.1	499	0.14	0.05	7.14	3.16	3	52	5	1.0	1325
B	840703	17.7	15.4	2.1	523	0.21	0.05	7.29	4.15	3	62	6	0.7	879
B	840724	18.5	16.2	2.1	477	0.36	0.13	8.43	2.77	3	58	3	0.3	332
B	840814	21.9	17.6	2.1	432	1.36	0.10	10.21	14.02	3	58	13	1.7	1955
B	840906	18.9	14.5	2.1	222	0.50	0.09	8.43	5.74	3	68	10	2.3	1644
B	840926	13.8	12.7	2.1	70	1.21	0.17	9.07	6.97	3	65	10	0.8	252
B	841016	13.8	9.9	2.1	117	1.71	0.09	8.50	18.98	3	66	9	0.3	36
B	841106	6.8	8.1	2.1	82	3.86	0.24	19.43	16.16	3	57	14	1.0	112
B	841127	3.8	5.7	2.1	47	6.43	0.19	22.64	33.21	3	56	15	6.5	2212
C	840502	14.7	7.5	7.4	455	0.14	0.06	6.00	2.21	4	56	23	2.3	1157
C	840523	19.1	11.8	7.4	474	1.00	0.09	4.00	11.07	4	56	23	3.8	4278
C	840612	20.4	13.9	7.4	499	0.14	0.05	7.14	3.16	4	69	5	5.5	1070
C	840703	21.8	15.4	7.4	523	0.21	0.05	7.29	4.15	4	62	13	1.8	802
C	840724	23.0	16.2	7.4	477	0.36	0.13	8.43	2.77	4	61	11	5.5	2051
C	840814	25.6	17.6	7.4	432	1.36	0.10	10.21	14.02	4	62	18	7.5	7852
C	840906	23.1	14.5	7.4	222	0.50	0.09	8.43	5.74	4	62	18	0.8	258
C	840926	20.2	12.7	7.4	70	1.21	0.17	9.07	6.97	4	60	27	1.3	603
C	841016	17.5	9.9	7.4	117	1.71	0.09	8.50	18.98	4	52	29	2.8	349
C	841106	15.2	8.1	7.4	82	3.86	0.24	19.43	16.16	4	54	11	0.8	50
C	841127	14.0	5.7	7.4	47	6.43	0.19	22.64	33.21	4	57	23	5.8	4920
D	840502	8.9	7.5	0.9	455	0.14	0.06	6.00	2.21	2	50	42	7.0	3224
D	840523	15.5	11.8	0.9	474	1.00	0.09	4.00	11.07	2	59	30	3.6	1557
D	840612	14.8	13.9	0.9	499	0.14	0.05	7.14	3.16	2	68	32	0.3	1111
D	840703	16.3	15.4	0.9	523	0.21	0.05	7.29	4.15	2	75	6	0.3	459
D	840724	17.1	16.2	0.9	477	0.36	0.13	8.43	2.77	2	69	9	0.3	746
D	840814	19.7	17.6	0.9	432	1.36	0.10	10.21	14.02	2	60	16	6.5	11585

D	840906	14.6	14.5	0.9	222	0.50	0.09	8.43	5.74	2	54	14	1.0	405
D	840926	13.0	12.7	0.9	70	1.21	0.17	9.07	6.97	2	62	11	1.0	484
D	841016	9.6	9.9	0.9	117	1.71	0.09	8.50	18.98	2	54	26	2.0	483
D	841106	8.1	8.1	0.9	82	3.86	0.24	19.43	16.16	2	63	22	1.5	1070
D	841127	5.5	5.7	0.9	47	6.43	0.19	22.64	33.21	2	59	17	3.8	179
E	840502	13.3	7.5	6.9	455	0.14	0.06	6.00	2.21	6	58	26	1.5	2586
E	840523	19.2	11.8	6.9	474	1.00	0.09	4.00	11.07	6	60	9	2.5	3690
E	840612	20.5	13.9	6.9	499	0.14	0.05	7.14	3.16	6	67	14	2.5	4653
E	840703	21.3	15.4	6.9	523	0.21	0.05	7.29	4.15	6	71	17	1.8	852
E	840724	22.0	16.2	6.9	477	0.36	0.13	8.43	2.77	6	60	11	2.0	327
E	840814	25.4	17.6	6.9	432	1.36	0.10	10.21	14.02	6	67	26	7.8	13367
E	840906	22.3	14.5	6.9	222	0.50	0.09	8.43	5.74	6	53	20	2.0	1809
E	840926	20.5	12.7	6.9	70	1.21	0.17	9.07	6.97	6	67	28	2.0	1105
E	841016	16.6	9.9	6.9	117	1.71	0.09	8.50	18.98	6	57	28	3.8	539
E	841106	13.7	8.1	6.9	82	3.86	0.24	19.43	16.16	6	59	19	1.0	344
E	841127	13.9	5.7	6.9	47	6.43	0.19	22.64	33.21	6	54	29	7.0	6240
F	840502	15.2	7.5	8.5	455	0.14	0.06	6.00	2.21	6	49	32	0.3	600
F	840523	19.5	11.8	8.5	474	1.00	0.09	4.00	11.07	6	74	19	0.3	1828
F	840612	21.2	13.9	8.5	499	0.14	0.05	7.14	3.16	6	70	13	0.8	12039
F	840703	21.7	15.4	8.5	523	0.21	0.05	7.29	4.15	6	73	11	0.8	261
F	840724	25.2	16.2	8.5	477	0.36	0.13	8.43	2.77	6	57	7	0.8	188
F	840814	25.4	17.6	8.5	432	1.36	0.10	10.21	14.02	6	73	23	6.5	16395
F	840906	24.2	14.5	8.5	222	0.50	0.09	8.43	5.74	6	73	9	1.0	115
F	840926	22.5	12.7	8.5	70	1.21	0.17	9.07	6.97	6	65	13	2.5	283
F	841016	19.5	9.9	8.5	117	1.71	0.09	8.50	18.98	6	56	15	6.3	1475
F	841106	16.6	8.1	8.5	82	3.86	0.24	19.43	16.16	6	73	8	1.0	49
F	841127	15.6	5.7	8.5	47	6.43	0.19	22.64	33.21	6	45	13	6.3	4656
G	840502	7.5	7.5	0.0	455	0.14	0.06	6.00	2.21	5	43	21	1.8	504
G	840523	11.8	11.8	0.0	474	1.00	0.09	4.00	11.07	5	59	22	0.5	583
G	840612	13.9	13.9	0.0	499	0.14	0.05	7.14	3.16	5	69	20	0.3	773
G	840703	15.4	15.4	0.0	523	0.21	0.05	7.29	4.15	5	70	14	1.0	152
G	840724	16.2	16.2	0.0	477	0.36	0.13	8.43	2.77	5	81	14	0.3	266
G	840814	17.6	17.6	0.0	432	1.36	0.10	10.21	14.02	5	74	19	1.5	1340
G	840906	14.5	14.5	0.0	222	0.50	0.09	8.43	5.74	5	81	27	1.0	1389
G	840926	12.7	12.7	0.0	70	1.21	0.17	9.07	6.97	5	75	19	1.0	227
G	841016	9.9	9.9	0.0	117	1.71	0.09	8.50	18.98	5	61	20	1.0	321
G	841106	8.1	8.1	0.0	82	3.86	0.24	19.43	16.16	5	55	14	1.0	212
G	841127	5.7	5.7	0.0	47	6.43	0.19	22.64	33.21	5	72	22	3.5	1180
H	840502	9.5	7.5	0.7	455	0.14	0.06	6.00	2.21	2	46	19	3.0	939
H	840523	15.2	11.8	0.7	474	1.00	0.09	4.00	11.07	2	48	4	3.3	1205
H	840612	14.1	13.9	0.7	499	0.14	0.05	7.14	3.16	2	31	7	5.7	1581
H	840703	16.3	15.4	0.7	523	0.21	0.05	7.29	4.15	2	62	9	1.0	2010
H	840724	16.3	16.2	0.7	477	0.36	0.13	8.43	2.77	2	57	10	5.7	6273
H	840814	19.3	17.6	0.7	432	1.36	0.10	10.21	14.02	2	36	14	9.0	31280
H	840906	15.6	14.5	0.7	222	0.50	0.09	8.43	5.74	2	42	7	1.0	268
H	840926	13.0	12.7	0.7	70	1.21	0.17	9.07	6.97	2	61	10	1.8	564

H	841016	10.0	9.9	0.7	117	1.71	0.09	8.50	18.98	2	52	15	3.0	176
H	841106	7.2	8.1	0.7	82	3.86	0.24	19.43	16.16	2	55	16	2.3	226
H	841127	4.6	5.7	0.7	47	6.43	0.19	22.64	33.21	2	53	11	4.3	681
I	840502	13.7	7.5	4.7	455	0.14	0.06	6.00	2.21	1	65	36	4.3	1966
I	840523	19.1	11.8	4.7	474	1.00	0.09	4.00	11.07	1	62	11	1.0	1613
I	840612	19.1	13.9	4.7	499	0.14	0.05	7.14	3.16	1	63	11	2.3	6738
I	840703	18.9	15.4	4.7	523	0.21	0.05	7.29	4.15	1	66	12	0.3	171
I	840724	19.4	16.2	4.7	477	0.36	0.13	8.43	2.77	1	66	25	1.5	479
I	840814	22.7	17.6	4.7	432	1.36	0.10	10.21	14.02	1	66	32	2.0	1152
I	840906	20.3	14.5	4.7	222	0.50	0.09	8.43	5.74	1	68	22	1.0	1117
I	840926	16.1	12.7	4.7	70	1.21	0.17	9.07	6.97	1	80	12	1.0	1143
I	841016	15.6	9.9	4.7	117	1.71	0.09	8.50	18.98	1	70	16	1.0	943
I	841106	11.6	8.1	4.7	82	3.86	0.24	19.43	16.16	1	59	11	0.5	142
I	841127	8.7	5.7	4.7	47	6.43	0.19	22.64	33.21	1	68	11	0.3	16
J	840502	12.2	7.5	1.3	455	0.14	0.06	6.00	2.21	1	46	26	4.0	2336
J	840523	19.3	11.8	1.3	474	1.00	0.09	4.00	11.07	1	49	31	9.0	15208
J	840612	16.8	13.9	1.3	499	0.14	0.05	7.14	3.16	1	52	13	1.8	196
J	840703	17.6	15.4	1.3	523	0.21	0.05	7.29	4.15	1	60	18	0.3	391
J	840724	18.9	16.2	1.3	477	0.36	0.13	8.43	2.77	1	64	9	1.8	1806
J	840814	18.4	17.6	1.3	432	1.36	0.10	10.21	14.02	1	52	16	3.0	5039
J	840906	15.5	14.5	1.3	222	0.50	0.09	8.43	5.74	1	61	10	0.7	1193
J	840926	12.0	12.7	1.3	70	1.21	0.17	9.07	6.97	1	53	9	0.3	603
J	841016	8.1	9.9	1.3	117	1.71	0.09	8.50	18.98	1	63	11	0.8	869
J	841106	6.2	8.1	1.3	82	3.86	0.24	19.43	16.16	1	46	10	1.0	1833
J	841127	2.2	5.7	1.3	47	6.43	0.19	22.64	33.21	1	64	10	1.0	911
K	840502	13.7	7.5	5.7	455	0.14	0.06	6.00	2.21	2	50	14	0.3	637
K	840523	18.7	11.8	5.7	474	1.00	0.09	4.00	11.07	2	56	8	1.0	1333
K	840612	20.1	13.9	5.7	499	0.14	0.05	7.14	3.16	2	52	10	7.0	3403
K	840703	19.4	15.4	5.7	523	0.21	0.05	7.29	4.15	2	52	8	0.3	86
K	840724	21.0	16.2	5.7	477	0.36	0.13	8.43	2.77	2	51	16	7.0	4064
K	840814	24.9	17.6	5.7	432	1.36	0.10	10.21	14.02	2	49	24	5.5	7307
K	840906	20.8	14.5	5.7	222	0.50	0.09	8.43	5.74	2	52	13	0.8	1467
K	840926	18.9	12.7	5.7	70	1.21	0.17	9.07	6.97	2	55	14	3.3	2655
K	841016	14.8	9.9	5.7	117	1.71	0.09	8.50	18.98	2	48	20	4.6	1608
K	841106	12.3	8.1	5.7	82	3.86	0.24	19.43	16.16	2	55	10	1.0	171
K	841127	11.6	5.7	5.7	47	6.43	0.19	22.64	33.21	2	45	15	6.8	3984
