# Supplementary material for: Diatom Cell Size, Coloniality and Motility: Trade-Offs between Temperature, Salinity and Nutrient Supply with Climate Change
Source: PLoS One. 2014 Oct 3;9(10):e109993. doi: 10.1371/journal.pone.0109993 (PMC4184900; doi:10.1371/journal.pone.0109993)
Supplement: Table S2 — Dataset “Temperature” with 121 site/dates and 230 diatom taxa: 1000 valves/sample. (PDF) [file pone.0109993.s002.pdf]

Table S2. Dataset "Temperature" with 121 site/dates and 230 diatom taxa: 1000 valves/sample

TAXON	SITE	DATE 840502	DATE 840523	DATE 840612	DATE 840703	DATE 840724	DATE 840814	DATE 840906	DATE 840926	DATE 841016	DATE 841106	DATE 841127
Acd_minu	A	0	0	2	0	0	4	2	1	0	0	3
Acd_pyre	A	0	0	0	0	0	0	0	2	0	0	0
Acd_suba	A	0	0	0	1	2	0	0	0	0	0	0
Ach_bica	A	0	0	0	0	0	0	0	0	0	0	0
Ach_brev	A	0	0	0	2	0	2	0	0	3	0	0
Ach_brin	A	0	0	0	0	0	0	0	0	12	7	10
Ach_long	A	0	0	0	0	0	0	0	0	0	0	0
Ach_misc	A	0	0	0	9	7	4	6	8	2	2	1
Ach_vist	A	0	0	0	2	0	2	0	1	0	0	0
Act_occr	A	0	0	0	0	0	0	0	0	0	0	0
Amp_comm	A	0	0	0	0	0	0	0	0	0	0	0
Amp_copu	A	0	0	0	0	0	0	0	2	1	0	0
Amp_fleb	A	0	5	10	8	10	2	0	3	0	0	0
Amp_inar	A	0	0	0	0	0	0	0	0	0	0	0
Amp_line	A	0	0	0	0	0	0	0	0	0	0	0
Amp_micr	A	0	0	0	2	0	0	0	0	0	0	0
Amp_oval	A	0	0	0	0	0	0	0	0	0	0	0
Amp_pedi	A	5	5	13	9	7	8	10	11	9	1	14
Amp_stau	A	0	0	0	0	0	0	0	0	0	0	0
Ane_tusc	A	0	0	0	0	0	0	0	0	0	0	0
Asa_bahu	A	0	2	0	0	0	0	0	0	0	0	0
Ast_form	A	0	0	0	0	0	0	0	0	0	0	0
Aul_spp	A	0	1	0	0	0	0	1	0	0	0	0
Bac_paxi	A	0	0	0	0	3	7	36	3	10	26	31
Ber_ruti	A	230	300	11	14	29	23	93	131	18	77	45
Bra_apon	A	0	0	0	0	0	0	0	0	0	0	0
Bre_lanc	A	0	0	0	0	0	0	0	2	0	4	4
Cal_baci	A	0	0	0	0	1	2	0	0	0	0	6
Cer_clos	A	4	0	0	5	2	8	0	1	0	0	0
Cha_spp.	A	24	45	13	14	13	11	9	6	11	5	3
Chp_marg	A	0	0	0	0	2	4	2	12	6	0	9
Coa_guil	A	0	0	0	0	0	0	0	1	0	0	0
Coc_neod	A	2	2	0	9	1	1	1	3	4	4	3
Coc_neot	A	0	0	1	0	0	2	7	0	4	0	7
Coc_pedi	A	0	4	26	6	2	17	19	0	14	5	40
Coc_pelt	A	0	0	0	0	0	0	0	0	0	0	0
Coc_plac	A	5	1	4	4	1	4	3	4	4	1	4
Coc_psth	A	0	0	1	0	0	0	0	0	0	0	2
Coc_scut	A	0	0	0	1	0	0	0	4	0	0	1
Con_weis	A	0	0	0	0	0	0	0	0	0	0	0
Cra_cusp	A	0	0	0	0	0	0	0	0	0	0	0
Cte_pulc	A	3	5	1	0	0	8	9	4	10	1	4
Cyb_amph	A	0	0	0	0	0	0	0	0	0	0	0
Cyc_atom	A	0	0	0	0	0	0	2	2	0	0	0
Cyc_choc	A	2	0	8	4	6	3	3	11	19	6	9
Cyc_mene	A	0	0	0	0	0	0	0	0	0	0	0
Cyc_radi	A	0	0	0	0	0	0	0	0	0	0	0
Cyc_stel	A	0	0	0	0	0	0	0	0	0	0	0
Cyc_stri	A	0	0	0	0	0	0	0	0	0	0	0
Cyl_grac	A	0	0	0	0	0	0	0	0	0	0	0
Cym_affi	A	0	0	0	0	0	0	0	0	0	0	0
Cym_cist	A	0	0	0	0	0	1	2	0	0	0	0
Cym_lanc	A	0	0	0	0	0	0	2	0	2	0	0
Den_cret	A	0	0	0	12	2	0	0	0	0	0	0
Den_sund	A	0	0	0	0	0	0	0	0	0	0	0
Dia_moni	A	273	195	290	58	76	36	14	18	6	18	7
Dia_tenu	A	0	0	0	0	1	2	2	0	0	0	0
Dia_vulg	A	64	50	1	0	9	0	0	0	3	1	2
Dip_domb	A	0	0	0	0	0	0	0	0	0	0	0
Dip_smit	A	0	0	2	0	0	0	0	0	0	0	0
Dip_smpu	A	0	0	0	0	0	0	0	0	0	0	0
Dip_smrh	A	0	0	0	0	0	0	0	0	0	0	0
Enc_caes	A	0	0	0	0	0	0	6	0	0	0	0
Enc_lacu	A	0	0	0	0	0	0	0	0	0	0	0
Enc_sile	A	0	0	0	0	0	0	0	0	0	0	0
Ent_cost	A	0	0	0	0	0	0	0	0	0	0	0
Ent_giga	A	0	0	0	0	0	0	0	0	0	0	0
Ent_palu	A	0	1	2	7	3	4	1	1	0	2	0
Ent_pseu	A	0	0	0	1	0	0	0	0	0	0	0
Ent_suri	A	1	1	0	0	0	0	0	0	0	0	0
Epi_adna	A	0	0	0	0	0	0	0	0	0	0	2

Epi_sore	A	0	3	0	3	1	33	24	1	3	2	2
Epi_tuwe	A	0	1	0	1	1	0	5	0	1	0	8
Fal_cryp	A	0	0	1	0	0	0	0	3	0	0	5
Fal_psli	A	0	0	0	0	0	0	0	0	0	0	0
Fal_pygm	A	0	0	0	0	0	0	0	0	0	0	0
Fra_amic	A	3	2	16	18	23	13	14	14	7	16	3
Fra_cava	A	0	0	0	0	0	0	0	0	0	0	0
Fra_cons	A	0	0	0	0	4	3	2	0	0	3	3
Fra_hydu	A	0	0	0	0	0	0	0	0	0	0	0
Fra_stri	A	1	0	7	4	1	3	0	0	0	0	0
Frp_cyli	A	0	0	0	0	0	0	0	0	0	0	0
Fru_creu	A	0	0	0	0	0	0	0	0	0	0	0
Gom_oliv	A	54	2	3	5	0	2	14	13	0	0	2
Gom_parv	A	0	0	0	0	0	0	0	0	0	0	0
Gon_exig	A	0	0	0	0	0	2	0	0	0	0	0
Gos_pseu	A	9	0	6	2	4	0	0	0	4	0	2
Gra_ocea	A	0	0	0	2	0	0	0	0	0	0	0
Gyr_exim	A	0	0	0	0	0	0	0	0	0	0	0
Gyr_fasc	A	0	1	0	0	0	0	0	0	0	0	0
Hal_acut	A	0	2	5	2	4	0	2	0	0	1	0
Hal_coff	A	0	0	3	10	16	4	10	16	0	10	0
Hal_exig	A	0	0	0	0	1	1	0	0	0	0	0
Hal_hols	A	0	0	0	0	0	0	0	0	0	0	0
Hal_hybr	A	0	0	0	0	0	0	0	0	0	0	0
Hal_luci	A	0	0	0	0	0	0	0	0	0	0	0
Hal_subh	A	0	0	0	0	0	0	0	0	0	0	0
Hal_tene	A	1	0	3	2	2	0	0	0	0	0	0
Hal_vene	A	0	0	0	0	0	0	0	0	0	0	0
Has_spic	A	0	0	3	4	2	9	3	0	0	0	0
Hip_hung	A	0	0	0	2	2	2	0	0	0	0	0
Hip_lesm	A	0	0	0	0	2	0	0	1	0	0	0
Hya_scot	A	0	0	0	2	0	0	0	3	0	0	2
Kar_amoe	A	0	0	0	0	0	0	1	0	2	0	8
Kar_clev	A	0	0	1	3	0	1	4	1	4	0	2
Lic_comm	A	0	0	0	0	0	0	0	0	0	0	0
Lic_debi	A	3	1	2	0	0	1	1	0	0	0	0
Lic_gran	A	0	0	0	0	0	0	0	0	0	1	0
Lic_rhom	A	0	0	0	2	0	0	0	0	0	5	0
Lun_bise	A	2	9	8	11	2	0	0	1	6	2	6
Lut_muti	A	0	0	0	0	0	0	0	0	0	0	0
Mar_atom	A	7	10	38	67	37	33	19	25	32	27	21
Mar_schu	A	0	0	0	0	0	0	0	0	0	0	0
Mas_balt	A	0	0	0	0	0	0	0	0	0	0	0
Mas_brau	A	0	0	0	0	0	0	0	0	0	0	0
Mas_elli	A	0	0	0	0	0	0	0	0	0	0	0
Mas_exig	A	0	0	0	0	0	0	0	0	0	0	0
Mas_pumi	A	2	0	0	0	0	0	6	0	0	0	0
Mas_smit	A	0	0	8	2	2	23	5	0	0	0	0
May_atom	A	0	0	0	0	0	0	1	0	0	0	0
Mel_arct	A	1	2	0	0	0	0	0	0	0	0	0
Mel_line	A	0	5	2	3	0	1	4	6	12	2	30
Mel_moni	A	0	0	1	1	1	1	2	0	19	10	67
Mel_numm	A	0	0	0	0	0	0	0	0	0	2	0
Mel_spec	A	4	32	8	2	11	13	45	197	18	217	23
Nav_aren	A	0	0	0	0	0	0	0	0	0	0	0
Nav_brem	A	0	2	0	0	2	0	0	0	2	0	0
Nav_cinc	A	0	0	0	0	0	0	0	0	0	0	0
Nav_clem	A	0	0	0	0	0	0	0	0	0	0	0
Nav_cryp	A	0	0	0	0	0	0	0	0	0	0	0
Nav_digi	A	0	0	0	0	0	0	0	0	0	0	0
Nav_duer	A	0	0	0	5	2	8	2	4	0	0	0
Nav_flan	A	0	0	0	0	1	0	0	0	0	0	0
Nav_greg	A	3	25	6	3	8	1	4	1	0	6	1
Nav_infi	A	0	0	0	0	2	14	7	32	6	2	0
Nav_lanc	A	2	2	1	0	0	0	0	0	0	0	0
Nav_meni	A	0	0	0	0	0	0	0	0	0	0	0
Nav_nole	A	0	0	0	0	0	0	0	0	0	0	0
Nav_pere	A	0	0	0	0	0	0	0	0	0	0	0
Nav_perm	A	52	41	34	60	42	11	82	20	95	84	145
Nav_phyl	A	2	5	2	2	2	1	0	1	0	1	0
Nav_rato	A	0	0	0	4	2	0	3	4	2	6	6
Nav_rhyn	A	0	0	0	0	0	0	0	0	0	0	0
Nav_saco	A	2	0	2	2	0	0	2	0	0	0	0
Nav_sana	A	0	0	0	0	0	0	0	0	0	0	0
Nav_sjoe	A	0	0	1	0	0	0	0	0	2	0	0

Nav_supr	A	0	0	0	0	1	0	0	0	0	0	0
Nav_trip	A	0	2	0	2	0	0	0	1	0	0	0
Nav_vene	A	0	0	0	1	6	0	0	2	0	0	0
Ncy_pusi	A	0	0	0	0	0	0	0	0	0	0	0
Nit_amph	A	0	0	0	0	0	4	0	0	0	0	1
Nit_anla	A	0	0	0	2	0	0	0	0	0	0	0
Nit_aura	A	2	0	0	0	0	0	0	0	0	0	0
Nit_brev	A	0	0	0	0	0	0	0	0	0	1	0
Nit_comm	A	0	0	0	0	0	0	0	0	0	0	0
Nit_diss	A	0	0	0	0	0	0	0	1	0	0	0
Nit_dubi	A	0	0	0	0	0	0	0	0	0	0	0
Nit_eleg	A	0	0	2	0	0	0	0	0	0	0	0
Nit_fili	A	0	0	0	0	22	0	1	8	28	109	101
Nit_flex	A	0	2	0	0	0	0	0	0	0	0	0
Nit_frus	A	64	43	120	99	111	46	139	23	53	21	160
Nit_gand	A	0	0	0	0	0	0	0	0	0	0	0
Nit_heuf	A	2	0	0	0	0	0	0	0	0	0	0
Nit_inco	A	0	1	0	0	2	0	1	1	0	0	2
Nit_lieb	A	0	0	0	3	7	10	4	2	0	0	0
Nit_lisu	A	0	0	0	0	0	0	0	0	0	2	0
Nit_lore	A	0	0	0	0	0	0	0	0	0	0	0
Nit_micr	A	2	0	4	7	19	8	1	2	4	8	6
Nit_pale	A	0	0	0	0	4	0	0	2	0	1	0
Nit_pate	A	0	0	0	0	1	0	0	0	0	0	0
Nit_pcea	A	1	4	45	86	30	173	27	8	0	9	0
Nit_pell	A	0	1	0	4	22	2	0	2	0	22	0
Nit_perm	A	0	0	0	2	0	0	0	1	0	0	0
Nit_pusi	A	63	41	5	3	7	0	7	4	0	2	0
Nit_reve	A	0	0	2	1	0	2	0	1	0	0	0
Nit_rose	A	7	4	14	23	22	73	50	39	5	25	0
Nit_sdea	A	0	0	0	0	0	0	0	0	0	0	0
Nit_sigm	A	0	0	0	0	0	0	0	0	0	0	1
Nit_soci	A	0	0	0	1	3	0	0	0	0	2	0
Nit_subc	A	0	0	0	0	0	0	0	0	0	0	0
Nit_supr	A	0	0	0	0	2	2	0	1	2	0	0
Nit_ther	A	0	4	0	10	71	2	0	3	0	19	2
Nit_vald	A	2	0	0	0	0	2	4	5	3	1	4
Ope_muta	A	4	13	31	52	42	26	22	27	50	13	8
Pau_taen	A	6	2	0	0	1	0	0	0	1	0	0
Pin_eleg	A	0	0	0	0	0	0	0	0	0	0	0
Pin_kroc	A	0	0	0	0	2	2	0	0	0	0	0
Pin_micr	A	0	0	0	0	0	0	0	0	0	0	0
Ple_elon	A	0	0	0	0	0	2	0	0	0	0	0
Ple_sali	A	0	0	0	0	0	0	0	2	0	0	1
Plt_deli	A	4	4	0	1	3	0	2	1	0	0	0
Plt_disp	A	0	0	0	0	0	0	2	0	0	0	0
Plt_freq	A	0	0	0	0	0	0	0	0	0	0	1
Plt_hauc	A	1	2	4	0	6	11	4	5	6	2	2
Plt_lemm	A	0	0	5	2	2	4	2	2	6	0	2
Plt_sept	A	0	0	2	0	0	0	0	1	0	0	0
Pro_buln	A	0	4	0	1	0	0	0	0	0	0	0
Pse_west	A	0	0	0	0	0	0	0	1	2	0	0
Psf_tene	A	0	0	0	0	0	0	0	0	0	0	0
Pss_brev	A	0	0	3	8	5	0	1	0	6	0	1
Pss_elli	A	35	47	124	250	226	192	83	188	122	102	50
Pss_zeil	A	4	12	26	20	14	26	14	28	26	19	19
Pst_punc	A	4	0	0	4	0	0	0	0	0	0	0
Ptd_gemm	A	0	0	0	0	0	0	0	0	0	0	0
Rei_sinu	A	0	1	0	0	0	0	0	0	0	0	0
Rho_abbr	A	13	21	47	13	10	38	61	7	97	20	35
Rhp_gibb	A	0	0	0	0	0	2	0	0	0	0	0
Rhp_gipa	A	0	0	0	0	0	0	0	0	0	0	0
Sel_pupu	A	0	0	0	0	0	0	0	0	0	0	0
Ske_cost	A	2	1	0	2	0	0	1	0	0	0	0
Std_hant	A	0	0	0	0	0	0	0	0	0	0	0
Std_minu	A	0	0	0	0	0	0	0	0	0	0	0
Stn_simu	A	0	0	0	0	0	0	0	0	0	0	0
Sur_breb	A	0	4	1	0	0	0	0	2	0	5	0
Syn_acus	A	0	0	0	0	0	0	0	0	0	0	0
Tab_fasc	A	16	23	15	6	4	29	72	25	76	21	17
Tab_tabu	A	3	0	0	0	0	2	7	10	129	37	36
Tab_waer	A	0	0	0	0	0	0	0	0	0	0	0
Tal_fene	A	0	0	0	0	0	0	2	0	0	0	0
Tal_floc	A	0	0	0	0	0	0	0	0	1	0	0
Tha_balt	A	0	0	2	0	1	1	0	5	2	2	0

Tha_leva	A	4	2	0	4	0	0	0	1	3	0	0
Tha_pros	A	0	0	0	0	0	0	0	12	20	0	2
Tha_pseu	A	0	0	2	1	0	4	1	3	2	0	6
Tro_dann	A	0	0	0	0	0	0	0	0	0	0	0
Try_angu	A	0	0	0	0	0	0	0	0	0	0	0
Try_apic	A	0	0	0	0	0	0	0	0	0	0	0
Try_ardu	A	0	0	0	0	0	2	0	0	0	0	0
Try_hung	A	0	0	0	0	0	0	0	0	1	0	0
Try_levi	A	0	0	0	0	0	0	0	0	2	0	0
Acd_minu	B	13	0	10	6	4	2	2	0	4	4	5
Acd_pyre	B	0	0	0	0	0	0	0	0	0	0	0
Acd_suba	B	0	0	0	0	0	0	0	0	0	0	0
Ach_bica	B	0	0	0	0	0	0	0	0	0	0	0
Ach_brev	B	0	0	0	0	1	0	0	0	0	0	0
Ach_brin	B	0	0	0	0	0	0	1	0	0	0	0
Ach_long	B	0	0	0	0	0	0	0	0	0	0	0
Ach_misc	B	0	0	2	3	0	0	0	6	7	0	2
Ach_vist	B	0	0	0	0	0	0	0	0	0	0	0
Act_occr	B	0	0	0	0	0	0	0	0	0	0	0
Amp_comm	B	0	0	0	0	0	0	0	0	0	0	0
Amp_copu	B	0	0	5	0	0	2	0	0	2	0	2
Amp_fleb	B	8	22	2	2	13	2	8	10	3	10	1
Amp_inar	B	2	0	0	0	0	0	0	0	0	0	2
Amp_line	B	1	0	0	0	0	0	0	0	0	0	0
Amp_micr	B	0	0	0	0	0	0	0	0	0	0	0
Amp_oval	B	0	0	0	0	0	0	0	0	0	1	0
Amp_pedi	B	16	4	6	11	5	23	4	5	6	7	0
Amp_stau	B	0	0	0	0	0	0	0	0	0	0	0
Ane_tusc	B	0	0	0	0	1	0	0	0	0	0	0
Asa_bahu	B	1	0	0	0	2	0	0	0	0	0	0
Ast_form	B	0	0	0	0	0	0	0	0	0	0	0
Aul_spp	B	0	0	0	0	0	0	0	0	0	2	0
Bac_paxi	B	0	0	0	0	0	0	2	8	0	3	1
Ber_ruti	B	28	15	19	7	2	23	26	16	10	90	31
Bra_apon	B	0	0	2	0	0	0	0	0	0	0	0
Bre_lanc	B	0	0	0	0	0	0	0	5	0	5	3
Cal_baci	B	0	0	2	0	0	0	0	0	0	0	2
Cer_clos	B	2	0	0	0	6	4	6	2	3	2	0
Cha_spp.	B	24	21	3	8	4	1	2	5	3	3	0
Chp_marg	B	0	0	0	2	0	0	0	0	1	1	0
Coa_guil	B	0	0	0	1	0	0	0	0	0	4	0
Coc_neod	B	1	0	0	3	9	1	0	3	1	0	8
Coc_neot	B	0	0	0	0	0	0	0	0	0	2	0
Coc_pedi	B	0	0	20	7	12	21	29	10	4	5	19
Coc_pelt	B	0	0	0	0	0	0	0	0	0	0	0
Coc_plac	B	4	0	8	2	5	6	12	3	5	2	14
Coc_psth	B	0	0	0	1	0	1	0	0	1	0	0
Coc_scut	B	0	0	0	0	0	0	0	0	0	0	0
Con_weis	B	0	0	0	0	0	0	0	0	0	0	0
Cra_cusp	B	0	0	0	0	0	0	0	0	0	0	0
Cte_pulc	B	4	0	2	5	0	0	12	2	5	6	2
Cyb_amph	B	0	0	0	0	0	0	0	0	0	0	0
Cyc_atom	B	0	0	1	0	0	0	0	0	0	0	0
Cyc_choc	B	0	0	0	2	0	0	4	9	14	4	0
Cyc_mene	B	0	0	0	0	0	0	0	0	0	0	0
Cyc_radi	B	0	0	0	0	0	0	0	0	0	0	0
Cyc_stel	B	0	0	0	0	0	0	0	0	0	0	0
Cyc_stri	B	0	0	0	0	0	0	0	0	0	0	0
Cyl_grac	B	0	0	0	0	0	0	0	0	0	0	0
Cym_affi	B	0	0	0	0	0	0	0	0	0	0	0
Cym_cist	B	0	2	0	0	0	0	0	0	0	5	1
Cym_lanc	B	0	0	0	0	0	0	0	0	0	0	6
Den_cret	B	4	2	8	38	6	3	0	0	4	4	2
Den_sund	B	0	0	0	0	0	0	0	0	0	0	0
Dia_moni	B	263	289	290	50	162	22	48	119	90	79	14
Dia_tenu	B	9	6	2	4	1	0	5	3	1	1	0
Dia_vulg	B	28	18	2	3	0	0	0	5	0	35	7
Dip_domb	B	0	0	0	0	0	0	0	0	0	0	0
Dip_smit	B	0	0	0	0	0	0	0	0	1	0	0
Dip_smpu	B	0	0	0	0	0	0	0	0	0	0	0
Dip_smrh	B	0	0	0	0	0	0	0	0	0	0	0
Enc_caes	B	0	0	0	4	0	0	4	2	0	0	0
Enc_lacu	B	4	0	0	0	0	0	0	0	0	0	0
Enc_sile	B	0	0	0	0	0	0	0	0	0	0	0
Ent_cost	B	0	0	0	0	0	0	0	0	0	0	0

Ent_giga	B	0	0	0	0	0	0	0	0	0	0	0
Ent_palu	B	0	5	0	7	1	4	2	11	4	18	2
Ent_pseu	B	0	0	0	0	0	0	0	0	0	0	0
Ent_suri	B	1	0	1	0	0	0	0	1	0	0	0
Epi_adna	B	0	0	0	0	0	2	0	0	0	0	0
Epi_sore	B	0	0	3	11	7	38	13	8	12	2	14
Epi_tuwe	B	0	0	2	1	0	4	0	0	0	0	2
Fal_cryp	B	0	0	0	0	2	0	0	0	0	0	0
Fal_psli	B	0	0	0	0	0	0	0	0	0	0	0
Fal_pygm	B	0	0	0	0	0	0	0	0	0	0	0
Fra_amic	B	0	13	2	7	2	6	4	4	4	6	1
Fra_cava	B	0	0	0	1	0	0	1	1	0	0	2
Fra_cons	B	2	0	15	4	25	36	54	123	106	47	3
Fra_hydu	B	0	0	0	0	0	0	0	0	0	0	0
Fra_stri	B	28	14	13	13	24	2	2	21	24	12	8
Frp_cyli	B	0	0	0	0	0	0	0	0	0	0	0
Fru_creu	B	0	0	0	0	0	0	0	0	0	0	0
Gom_oliv	B	128	256	11	15	2	0	16	131	92	153	43
Gom_parv	B	0	0	0	0	0	0	0	0	0	0	0
Gon_exig	B	0	0	0	0	0	0	0	0	0	0	0
Gos_pseu	B	6	4	0	6	0	5	9	4	0	0	13
Gra_ocea	B	0	0	0	0	0	0	0	0	0	0	0
Gyr_exim	B	0	0	0	0	0	0	0	0	0	0	0
Gyr_fasc	B	0	0	0	0	0	0	0	0	0	0	0
Hal_acut	B	2	0	0	0	0	0	4	2	0	0	3
Hal_coff	B	0	4	2	7	11	2	4	7	5	4	4
Hal_exig	B	0	0	0	0	0	0	0	0	0	0	0
Hal_hols	B	0	0	0	0	0	0	1	0	0	0	0
Hal_hybr	B	0	0	0	0	0	0	0	0	0	0	0
Hal_luci	B	0	0	0	0	0	0	0	0	0	0	0
Hal_subh	B	0	0	0	0	0	0	0	0	0	0	0
Hal_tene	B	0	1	4	8	8	1	1	0	2	0	1
Hal_vene	B	0	0	0	1	0	0	0	0	0	0	0
Has_spic	B	2	2	3	7	2	6	2	25	6	7	0
Hip_hung	B	0	0	0	2	0	0	0	0	0	0	0
Hip_lesm	B	0	0	0	1	2	0	0	0	2	1	0
Hya_scot	B	0	0	0	0	0	0	0	0	0	0	0
Kar_amoe	B	0	0	0	0	0	0	0	0	0	0	0
Kar_clev	B	0	0	0	1	1	0	0	0	2	1	0
Lic_comm	B	0	0	0	0	0	1	0	5	0	0	0
Lic_debi	B	1	0	6	0	0	4	5	9	0	1	0
Lic_gran	B	0	0	0	0	0	0	0	0	0	0	0
Lic_rhom	B	0	0	0	0	2	8	11	16	2	2	0
Lun_bise	B	39	11	11	0	6	0	3	1	2	0	2
Lut_muti	B	0	0	0	0	0	2	0	0	0	0	0
Mar_atom	B	6	17	11	15	20	14	4	9	9	5	7
Mar_schu	B	0	0	0	0	0	0	2	0	0	0	0
Mas_balt	B	0	0	0	0	0	8	0	0	0	0	0
Mas_brau	B	0	0	0	0	0	0	0	0	0	0	0
Mas_elli	B	0	0	0	0	0	2	0	0	0	0	4
Mas_exig	B	0	0	0	0	0	0	0	0	0	0	0
Mas_pumi	B	0	0	1	0	0	23	28	2	0	0	4
Mas_smit	B	0	4	20	19	8	230	95	6	3	0	47
May_atom	B	0	0	0	0	0	0	0	0	0	0	0
Mel_arct	B	0	0	0	0	0	0	0	0	0	0	0
Mel_line	B	0	0	0	2	3	2	0	0	0	0	0
Mel_moni	B	0	0	0	0	0	0	1	0	0	0	37
Mel_numm	B	0	0	0	0	0	0	0	0	0	0	0
Mel_spec	B	0	0	0	0	0	3	35	6	12	65	4
Nav_aren	B	0	0	0	0	0	0	0	0	0	0	0
Nav_brem	B	0	0	0	0	0	0	0	2	0	0	0
Nav_cinc	B	0	0	0	0	0	0	0	0	0	0	0
Nav_clem	B	0	0	0	0	0	0	0	0	0	0	0
Nav_cryp	B	0	0	0	0	0	0	0	0	0	0	0
Nav_digi	B	0	0	0	0	2	0	0	0	1	0	0
Nav_duer	B	0	4	0	0	2	4	5	11	0	0	0
Nav_flan	B	0	0	0	0	0	0	1	0	1	0	0
Nav_greg	B	3	5	2	6	19	0	2	3	6	4	0
Nav_infi	B	0	0	0	0	0	0	4	4	5	28	0
Nav_lanc	B	0	0	0	0	0	0	0	0	0	0	0
Nav_meni	B	0	0	0	0	0	0	0	0	0	0	0
Nav_nole	B	0	0	0	0	0	0	0	0	2	0	0
Nav_pere	B	0	0	0	0	0	0	0	0	0	0	0
Nav_perm	B	21	46	21	62	117	20	105	25	66	52	61
Nav_phyl	B	0	2	0	4	4	0	2	0	0	0	0

Nav_rato	B	0	2	0	0	0	0	0	0	0	0	12
Nav_rhyn	B	0	0	0	0	0	0	0	0	0	0	0
Nav_saco	B	0	0	0	0	0	4	0	0	2	0	0
Nav_sana	B	0	0	0	0	0	0	2	0	0	0	0
Nav_sjoe	B	8	2	1	0	0	0	0	0	0	0	0
Nav_supr	B	4	0	0	0	0	0	0	0	0	0	0
Nav_trip	B	0	0	0	2	0	0	0	0	0	0	0
Nav_vene	B	0	0	0	0	0	0	2	0	0	0	0
Ncy_pusi	B	0	0	0	2	0	0	2	0	0	0	2
Nit_amph	B	0	0	0	0	0	0	0	0	0	0	0
Nit_anla	B	0	0	0	2	0	0	0	0	1	0	0
Nit_aura	B	0	0	0	0	0	0	0	0	0	0	0
Nit_brev	B	0	0	0	0	0	0	0	0	0	0	0
Nit_comm	B	0	0	0	0	0	0	0	0	0	0	0
Nit_diss	B	0	0	0	0	0	0	0	0	0	0	0
Nit_dubi	B	0	0	0	0	0	0	0	0	0	0	0
Nit_eleg	B	0	0	0	0	0	0	0	0	0	0	0
Nit_fili	B	0	2	0	0	2	0	0	0	0	0	0
Nit_flex	B	0	0	0	0	0	0	0	2	3	4	0
Nit_frus	B	218	59	330	399	129	168	73	53	98	25	257
Nit_gand	B	0	0	0	0	0	0	0	0	0	0	0
Nit_heuf	B	0	0	0	0	0	0	0	0	0	0	0
Nit_inco	B	0	0	0	0	0	0	0	0	0	0	0
Nit_lieb	B	4	0	2	4	2	8	5	2	0	0	10
Nit_lisu	B	0	0	0	0	0	0	0	0	0	0	0
Nit_lore	B	0	0	0	0	0	0	0	0	0	0	0
Nit_micr	B	0	4	6	3	8	2	3	5	5	0	3
Nit_pale	B	8	9	2	1	2	6	2	2	0	0	19
Nit_pate	B	4	0	0	0	0	1	2	2	2	0	0
Nit_pcea	B	9	24	20	14	118	21	18	47	14	34	11
Nit_pell	B	0	1	0	0	2	2	0	0	8	40	1
Nit_perm	B	0	0	0	0	0	0	0	0	0	0	0
Nit_pusi	B	8	0	0	0	2	8	2	3	0	0	0
Nit_reve	B	0	0	0	0	0	0	0	0	0	0	0
Nit_rose	B	18	20	10	12	32	17	15	18	21	30	16
Nit_sdea	B	0	0	0	0	0	0	0	0	0	0	0
Nit_sigm	B	0	0	0	1	0	0	0	0	0	0	0
Nit_soci	B	0	0	0	0	0	0	0	0	0	0	0
Nit_subc	B	0	0	0	0	0	0	0	0	1	4	0
Nit_supr	B	0	0	1	1	4	0	0	2	3	2	0
Nit_ther	B	1	1	1	0	0	0	6	11	38	48	4
Nit_vald	B	3	0	4	6	0	7	1	0	0	0	0
Ope_muta	B	0	4	3	26	16	20	11	4	13	7	0
Pau_taen	B	1	0	0	0	2	0	0	0	0	0	1
Pin_eleg	B	0	0	0	0	0	0	0	0	0	0	0
Pin_kroc	B	0	0	0	0	0	0	0	0	0	0	0
Pin_micr	B	0	0	0	0	0	0	0	0	0	0	0
Ple_elon	B	0	0	0	0	0	0	0	0	0	0	0
Ple_sali	B	0	0	0	0	0	0	0	0	0	0	0
Plt_deli	B	0	0	0	3	0	1	2	0	1	0	0
Plt_disp	B	0	0	0	0	0	0	0	0	0	0	0
Plt_freq	B	0	0	0	0	0	0	0	0	0	0	0
Plt_hauc	B	0	5	2	0	7	4	3	3	1	1	1
Plt_lemm	B	0	0	2	0	1	0	0	3	1	0	0
Plt_sept	B	0	0	0	0	0	0	0	0	0	0	0
Pro_buln	B	0	4	0	0	0	0	7	0	0	0	0
Pse_west	B	0	0	0	0	0	0	0	0	0	0	0
Psf_tene	B	0	0	0	0	0	0	0	0	0	0	0
Pss_brev	B	0	2	0	5	3	6	0	4	5	3	0
Pss_elli	B	17	57	46	100	77	110	74	53	55	39	24
Pss_zeil	B	4	10	8	7	14	9	13	14	14	17	3
Pst_punc	B	0	0	0	1	0	0	0	0	0	0	0
Ptd_gemm	B	0	0	0	0	0	0	0	0	0	0	0
Rei_sinu	B	0	0	0	0	0	0	0	0	0	0	0
Rho_abbr	B	15	13	43	42	57	52	121	64	108	19	227
Rhp_gibb	B	0	0	0	0	0	0	2	2	0	0	0
Rhp_gipa	B	0	0	0	0	0	0	0	0	0	0	0
Sel_pupu	B	0	0	0	0	0	0	0	0	0	0	0
Ske_cost	B	4	3	0	0	0	0	0	0	0	0	0
Std_hant	B	0	0	0	0	0	0	0	0	0	0	0
Std_minu	B	0	0	0	0	0	0	0	0	0	0	0
Stn_simu	B	0	0	0	0	0	0	0	0	0	0	0
Sur_breb	B	2	0	0	0	0	0	1	0	2	1	2
Syn_acus	B	0	0	0	0	0	0	0	0	0	0	0
Tab_fasc	B	17	10	6	9	15	13	34	42	45	38	12

Tab_tabu	B	0	0	0	1	0	2	12	5	9	0	11
Tab_waer	B	0	0	0	0	0	0	0	0	0	0	0
Tal_fene	B	0	0	0	0	0	0	0	0	0	0	0
Tal_floc	B	0	0	0	0	0	0	0	0	0	0	0
Tha_balt	B	0	0	0	0	1	1	0	0	2	1	0
Tha_leva	B	2	0	1	0	0	0	0	0	0	0	0
Tha_pros	B	0	0	0	0	0	0	0	8	15	4	0
Tha_pseu	B	1	1	0	0	0	0	6	1	0	0	0
Tro_dann	B	0	0	0	0	0	0	0	0	0	0	0
Try_angu	B	0	0	0	0	0	0	0	0	0	0	0
Try_apic	B	1	0	0	0	3	0	0	0	0	0	0
Try_ardu	B	0	0	0	0	0	0	0	0	0	0	0
Try_hung	B	0	0	0	0	0	0	0	0	0	0	0
Try_levi	B	0	0	0	0	0	0	0	0	0	0	0
Acd_minu	C	0	0	2	2	2	1	0	0	0	0	0
Acd_pyre	C	2	0	0	0	0	0	0	0	0	0	0
Acd_suba	C	0	0	0	0	0	0	0	0	0	0	0
Ach_bica	C	0	0	0	0	0	0	0	0	0	0	0
Ach_brev	C	0	0	0	0	0	0	0	0	4	0	0
Ach_brin	C	0	0	0	0	2	4	0	0	3	2	47
Ach_long	C	0	0	0	0	0	0	0	0	0	0	0
Ach_misc	C	0	2	7	2	4	7	8	4	0	2	0
Ach_vist	C	0	0	0	0	2	0	0	0	0	0	0
Act_occr	C	0	0	0	0	0	0	0	0	0	0	1
Amp_comm	C	0	0	0	0	0	0	0	0	0	0	0
Amp_copu	C	0	0	0	0	0	0	0	0	0	0	0
Amp_fleb	C	12	12	12	5	13	1	2	2	0	2	0
Amp_inar	C	0	0	0	0	0	0	0	2	0	0	0
Amp_line	C	0	0	0	0	0	0	0	0	0	0	0
Amp_micr	C	0	0	0	0	0	0	0	0	0	0	0
Amp_oval	C	0	0	0	0	0	0	0	0	0	0	0
Amp_pedi	C	2	5	1	3	11	5	6	8	0	2	1
Amp_stau	C	0	0	0	0	0	0	0	0	0	0	0
Ane_tusc	C	0	0	0	0	0	0	0	0	0	0	0
Asa_bahu	C	0	0	5	2	0	0	0	0	0	0	0
Ast_form	C	0	0	0	0	0	0	0	0	0	0	0
Aul_spp	C	0	0	0	0	0	1	0	0	0	0	0
Bac_paxi	C	1	3	13	0	2	6	35	29	12	88	9
Ber_ruti	C	131	372	32	6	6	14	31	75	46	7	28
Bra_apon	C	0	2	0	0	0	0	0	0	0	0	0
Bre_lanc	C	0	0	0	0	0	0	0	4	0	1	3
Cal_baci	C	2	2	0	0	0	0	0	2	0	0	0
Cer_clos	C	0	0	0	26	32	8	4	0	0	2	0
Cha_spp.	C	16	30	13	7	3	1	5	6	3	0	3
Chp_marg	C	0	0	2	3	2	1	8	0	0	0	0
Coa_guil	C	2	0	0	0	0	0	0	0	0	0	0
Coc_neod	C	2	3	2	2	6	4	1	1	3	0	8
Coc_neot	C	0	0	2	0	0	2	1	1	0	0	1
Coc_pedi	C	4	0	0	0	6	18	6	5	0	0	40
Coc_pelt	C	0	0	0	0	0	0	0	0	0	0	0
Coc_plac	C	2	1	9	5	9	11	21	5	3	2	12
Coc_psth	C	1	0	0	0	0	1	0	0	0	0	1
Coc_scut	C	0	0	0	0	0	0	0	0	0	0	0
Con_weis	C	0	0	0	0	0	0	0	0	0	0	0
Cra_cusp	C	0	0	0	0	0	0	0	0	0	0	0
Cte_pulc	C	4	0	2	1	0	4	0	0	0	0	2
Cyb_amph	C	0	0	0	0	0	0	0	0	0	0	0
Cyc_atom	C	0	0	0	0	3	0	0	4	0	0	0
Cyc_choc	C	0	0	0	2	0	4	1	8	4	2	3
Cyc_mene	C	0	0	0	0	0	0	0	0	1	1	0
Cyc_radi	C	0	0	0	0	0	0	0	0	0	1	0
Cyc_stel	C	0	0	0	0	0	0	0	1	0	0	0
Cyc_stri	C	0	0	0	0	0	0	0	0	0	0	0
Cyl_grac	C	0	0	0	0	0	0	0	0	0	0	0
Cym_affi	C	0	0	0	0	0	0	0	0	0	0	0
Cym_cist	C	0	2	0	0	0	0	0	2	0	0	0
Cym_lanc	C	0	0	0	0	0	0	0	0	0	0	0
Den_cret	C	0	0	0	0	0	0	0	0	2	2	2
Den_sund	C	0	0	0	0	0	0	0	0	0	0	0
Dia_moni	C	187	117	51	154	70	6	3	17	4	8	1
Dia_tenu	C	11	6	7	4	0	1	0	4	0	0	0
Dia_vulg	C	151	28	2	0	0	0	0	0	0	0	1
Dip_domb	C	0	0	0	0	0	0	0	0	0	0	0
Dip_smit	C	0	0	0	0	0	0	0	0	0	0	0
Dip_smpu	C	0	0	0	0	0	0	0	0	0	0	0

Dip_smrh	C	0	0	0	0	0	0	0	0	0	0	0
Enc_caes	C	0	0	0	0	0	0	4	2	3	0	0
Enc_lacu	C	0	0	0	0	0	0	0	0	0	0	0
Enc_sile	C	0	0	0	1	0	0	0	0	0	0	0
Ent_cost	C	0	0	0	0	0	0	0	0	0	0	0
Ent_giga	C	0	0	0	0	0	0	0	0	0	0	0
Ent_palu	C	3	4	4	5	8	8	2	0	0	3	0
Ent_pseu	C	0	0	1	0	0	0	0	0	0	0	0
Ent_suri	C	1	5	0	0	1	0	0	0	0	0	0
Epi_adna	C	0	0	0	0	0	0	2	0	0	0	2
Epi_sore	C	0	2	1	2	20	83	33	16	0	0	11
Epi_tuwe	C	0	0	0	0	1	0	0	0	0	0	1
Fal_cryp	C	0	0	0	0	0	0	3	2	0	0	0
Fal_psli	C	0	0	0	0	0	0	0	0	0	0	0
Fal_pygm	C	0	0	0	0	0	0	0	0	0	0	0
Fra_amic	C	0	4	9	15	5	10	9	13	1	3	2
Fra_cava	C	0	2	2	0	0	0	0	0	0	0	0
Fra_cons	C	0	0	1	2	3	1	0	1	0	0	0
Fra_hydu	C	0	0	0	0	0	0	0	0	0	0	0
Fra_stri	C	2	9	6	0	4	0	0	0	0	0	0
Frp_cyli	C	0	0	0	0	0	0	0	0	0	0	0
Fru_creu	C	0	0	0	0	0	0	0	0	0	0	0
Gom_oliv	C	7	4	7	1	1	2	3	1	4	0	0
Gom_parv	C	0	0	0	0	0	0	0	0	0	0	0
Gon_exig	C	0	0	0	0	0	0	0	0	0	0	0
Gos_pseu	C	0	0	2	0	5	4	5	0	1	0	0
Gra_ocea	C	0	0	0	0	0	0	0	0	0	0	0
Gyr_exim	C	0	0	0	0	0	0	0	0	0	0	0
Gyr_fasc	C	0	0	0	0	0	0	0	0	0	0	0
Hal_acut	C	0	0	2	7	3	7	11	3	2	1	0
Hal_coff	C	1	3	16	9	16	2	5	28	8	7	5
Hal_exig	C	0	0	0	0	0	0	0	0	1	0	0
Hal_hols	C	0	0	0	0	1	0	0	0	0	0	0
Hal_hybr	C	0	0	0	0	0	0	0	0	0	0	0
Hal_luci	C	0	0	0	0	0	0	0	0	0	0	0
Hal_subh	C	1	0	3	0	0	0	0	0	1	0	8
Hal_tene	C	0	0	2	0	1	0	0	0	0	0	0
Hal_vene	C	0	0	0	0	0	0	0	0	0	0	0
Has_spic	C	0	0	8	6	11	0	4	4	2	0	0
Hip_hung	C	0	0	4	2	0	0	0	0	0	0	0
Hip_lesm	C	0	0	1	1	0	0	2	0	1	10	0
Hya_scot	C	0	0	0	1	0	1	0	0	0	0	0
Kar_amoe	C	0	0	0	0	0	0	0	0	0	0	2
Kar_clev	C	0	0	0	0	0	0	2	2	0	0	1
Lic_comm	C	0	0	0	0	0	0	0	0	0	0	0
Lic_debi	C	0	0	0	0	0	0	1	0	0	0	0
Lic_gran	C	0	0	0	0	0	0	0	0	0	0	0
Lic_rhom	C	0	0	0	0	0	0	0	0	0	0	0
Lun_bise	C	0	5	2	1	0	0	0	0	0	0	0
Lut_muti	C	0	0	0	0	0	0	0	0	0	0	0
Mar_atom	C	6	15	7	39	29	19	26	29	11	26	16
Mar_schu	C	0	0	0	0	0	0	0	0	0	0	0
Mas_balt	C	0	0	0	0	0	0	0	0	0	0	0
Mas_brau	C	0	0	0	0	0	0	0	0	0	0	0
Mas_elli	C	0	0	0	0	1	2	0	0	0	0	0
Mas_exig	C	0	0	0	0	0	0	0	0	0	0	0
Mas_pumi	C	0	0	0	0	0	2	7	0	2	0	0
Mas_smit	C	0	0	5	2	32	14	15	12	2	0	5
May_atom	C	0	0	0	0	0	0	0	0	0	0	0
Mel_arct	C	0	0	0	0	0	0	0	0	0	0	0
Mel_line	C	13	13	0	1	2	0	1	3	6	0	15
Mel_moni	C	2	4	5	6	0	6	0	0	4	5	54
Mel_numm	C	6	4	0	0	0	0	0	0	0	0	4
Mel_spec	C	107	29	8	28	59	99	152	108	42	221	12
Nav_aren	C	0	0	0	0	0	0	0	1	0	0	0
Nav_brem	C	0	0	0	0	0	0	0	0	0	0	0
Nav_cinc	C	0	0	0	0	0	0	0	0	0	0	0
Nav_clem	C	0	0	0	0	0	0	0	0	0	0	0
Nav_cryp	C	0	0	0	0	0	0	0	0	0	0	0
Nav_digi	C	1	0	0	0	0	1	0	0	0	0	0
Nav_duer	C	0	2	18	2	6	1	0	0	2	0	3
Nav_flan	C	0	0	0	0	0	0	0	0	0	0	0
Nav_greg	C	1	2	4	4	1	0	2	0	0	12	0
Nav_infi	C	6	3	3	8	12	13	5	8	2	2	2
Nav_lanc	C	9	0	1	0	0	0	0	0	0	0	0

Nav_meni	C	0	0	0	0	0	0	0	0	0	0	0
Nav_nole	C	0	0	0	0	0	0	0	0	0	0	0
Nav_pere	C	0	0	0	0	0	0	0	0	0	0	0
Nav_perm	C	87	47	103	76	81	42	141	228	251	180	135
Nav_phyl	C	0	4	1	1	0	0	0	0	2	2	0
Nav_rato	C	0	6	12	0	0	0	2	0	0	25	5
Nav_rhyn	C	0	0	0	0	0	0	0	0	0	0	0
Nav_saco	C	2	0	2	0	0	0	0	0	4	0	0
Nav_sana	C	0	0	0	0	0	0	0	0	0	0	0
Nav_sjoe	C	0	0	0	0	0	0	0	0	0	0	0
Nav_supr	C	0	0	0	0	0	1	0	1	0	0	0
Nav_trip	C	0	0	0	0	0	0	0	0	0	0	2
Nav_vene	C	0	0	0	0	0	0	0	0	0	0	0
Ncy_pusi	C	0	0	0	0	0	0	2	4	0	0	4
Nit_amph	C	0	0	1	0	0	0	0	0	0	0	0
Nit_anla	C	0	0	2	0	0	0	2	1	0	2	0
Nit_aura	C	0	0	0	0	0	0	0	0	0	0	0
Nit_brev	C	0	0	0	0	0	0	0	0	0	0	0
Nit_comm	C	0	0	0	0	0	0	0	0	0	1	0
Nit_diss	C	0	0	0	0	2	0	0	0	0	2	0
Nit_dubi	C	0	0	0	0	0	0	0	0	0	0	0
Nit_eleg	C	0	0	0	0	0	0	0	0	0	0	0
Nit_fili	C	15	7	0	9	0	0	4	3	24	94	136
Nit_flex	C	0	0	0	0	0	0	0	0	0	0	0
Nit_frus	C	32	28	130	33	123	142	110	77	189	36	135
Nit_gand	C	0	0	0	0	0	0	0	0	0	0	0
Nit_heuf	C	1	0	0	0	0	0	0	0	0	0	0
Nit_inco	C	2	0	1	1	0	0	0	0	0	2	0
Nit_lieb	C	0	0	1	6	3	0	7	4	16	4	14
Nit_lisu	C	0	0	0	0	0	0	0	0	0	0	0
Nit_lore	C	0	0	0	0	0	0	0	0	0	0	0
Nit_micr	C	3	7	21	12	10	20	32	9	7	9	6
Nit_pale	C	1	1	5	0	5	0	0	0	0	3	0
Nit_pate	C	0	0	0	4	2	0	0	0	0	0	0
Nit_pcea	C	8	16	153	133	16	5	9	3	5	5	3
Nit_pell	C	0	1	4	5	1	2	1	0	0	22	0
Nit_perm	C	0	0	1	0	0	1	1	0	0	0	0
Nit_pusi	C	6	5	0	5	1	0	0	0	0	0	0
Nit_reve	C	0	0	0	0	0	0	1	0	0	0	0
Nit_rose	C	5	20	137	133	115	49	35	32	19	68	14
Nit_sdea	C	0	0	0	0	0	0	0	0	0	0	0
Nit_sigm	C	0	0	0	0	0	0	0	0	0	0	0
Nit_soci	C	0	0	0	0	0	0	0	0	0	0	0
Nit_subc	C	1	0	0	0	0	0	0	0	0	0	0
Nit_supr	C	0	0	0	0	0	4	6	2	0	4	0
Nit_ther	C	1	2	2	1	1	0	0	0	2	7	0
Nit_vald	C	0	0	0	0	0	2	1	2	0	2	0
Ope_muta	C	0	13	8	27	16	25	20	16	4	3	13
Pau_taen	C	0	2	2	1	0	0	0	0	0	0	0
Pin_eleg	C	0	0	0	0	0	1	0	0	0	0	0
Pin_kroc	C	0	0	0	0	0	0	0	0	0	0	0
Pin_micr	C	0	0	0	0	0	0	0	0	0	0	0
Ple_elon	C	0	0	0	0	0	0	0	1	0	0	0
Ple_sali	C	0	0	0	0	0	0	0	1	2	0	0
Plt_deli	C	3	0	0	1	2	1	1	0	0	4	6
Plt_disp	C	0	0	0	0	0	0	0	0	0	0	0
Plt_freq	C	0	0	1	2	1	1	0	1	0	0	0
Plt_hauc	C	1	3	2	0	8	2	6	2	1	4	0
Plt_lemm	C	0	0	0	0	0	1	1	2	0	0	0
Plt_sept	C	2	0	0	0	0	0	0	0	0	1	0
Pro_buln	C	1	0	0	2	2	24	41	2	2	0	0
Pse_west	C	0	0	0	0	0	0	0	0	1	0	0
Psf_tene	C	0	0	0	0	0	0	0	0	0	0	0
Pss_brev	C	0	0	4	1	2	0	2	0	8	2	1
Pss_elli	C	37	66	86	126	133	159	92	108	42	52	38
Pss_zeil	C	17	15	10	25	19	29	3	14	6	10	25
Pst_punc	C	0	0	0	0	0	0	0	0	0	0	0
Ptd_gemm	C	0	0	0	0	0	0	0	0	0	0	0
Rei_sinu	C	0	0	0	0	0	0	0	0	0	0	0
Rho_abbr	C	4	8	12	13	26	82	25	35	167	13	114
Rhp_gibb	C	0	2	0	0	0	3	2	2	2	0	4
Rhp_gipa	C	0	0	0	0	0	0	0	0	0	0	0
Sel_pupu	C	0	0	0	0	0	0	0	0	0	0	0
Ske_cost	C	7	1	0	0	0	0	0	0	0	0	0
Std_hant	C	0	0	0	0	0	0	0	0	0	0	0

Std_minu	C	0	0	0	0	0	0	0	0	0	0	0
Stn_simu	C	0	0	0	0	0	0	0	0	0	0	0
Sur_breb	C	5	2	0	0	0	1	0	0	0	7	0
Syn_acus	C	0	0	0	0	0	0	0	0	0	0	0
Tab_fasc	C	57	43	12	11	20	27	26	33	41	14	15
Tab_tabu	C	2	2	1	0	16	1	0	4	24	8	13
Tab_waer	C	0	0	0	0	0	0	0	0	0	0	0
Tal_fene	C	0	0	0	0	0	0	0	0	0	0	0
Tal_floc	C	0	0	1	0	0	0	0	0	0	0	0
Tha_balt	C	0	2	0	2	0	0	1	0	0	0	1
Tha_leva	C	4	2	0	0	0	0	0	0	0	0	0
Tha_pros	C	0	0	0	0	0	0	0	0	1	0	5
Tha_pseu	C	0	0	1	1	0	0	0	0	0	0	1
Tro_dann	C	0	0	0	0	0	0	0	0	0	0	0
Try_angu	C	0	0	0	0	0	0	0	0	0	0	0
Try_apic	C	0	0	0	2	0	0	0	0	0	0	2
Try_ardu	C	0	0	0	0	0	0	0	0	0	2	0
Try_hung	C	0	0	0	0	0	0	0	0	0	0	0
Try_levi	C	0	0	0	0	0	0	0	0	0	0	2
Acd_minu	D	2	5	8	9	12	62	15	20	0	0	7
Acd_pyre	D	0	0	0	0	0	2	0	0	0	0	0
Acd_suba	D	0	0	0	2	0	0	0	0	0	0	0
Ach_bica	D	0	0	0	0	0	0	0	0	0	0	0
Ach_brev	D	0	0	0	0	0	0	0	0	0	0	0
Ach_brin	D	0	0	0	0	0	0	0	1	0	0	0
Ach_long	D	1	0	0	0	0	0	0	0	0	0	0
Ach_misc	D	2	0	0	6	6	0	0	4	12	0	2
Ach_vist	D	0	0	0	0	0	2	0	0	0	0	0
Act_occr	D	0	0	0	0	0	0	0	0	0	0	0
Amp_comm	D	0	0	0	0	0	0	0	0	0	0	0
Amp_copu	D	0	0	0	0	0	0	0	2	0	0	0
Amp_fleb	D	9	27	30	19	24	9	3	6	3	4	0
Amp_inar	D	0	0	0	0	0	0	0	0	0	0	0
Amp_line	D	0	0	0	0	0	0	0	0	0	0	0
Amp_micr	D	0	0	0	0	0	0	0	0	0	0	0
Amp_oval	D	0	0	0	0	0	0	0	0	0	0	0
Amp_pedi	D	0	6	3	18	8	1	4	11	5	3	15
Amp_stau	D	0	0	0	2	0	0	0	0	0	0	0
Ane_tusc	D	0	0	0	0	0	0	0	0	0	1	0
Asa_bahu	D	0	0	0	0	0	0	0	0	1	0	0
Ast_form	D	0	0	1	0	0	0	0	0	0	0	0
Aul_spp	D	0	0	0	5	2	0	0	0	0	2	4
Bac_paxi	D	1	0	5	0	3	1	10	46	89	61	70
Ber_ruti	D	345	221	158	11	15	21	1	35	63	40	60
Bra_apon	D	0	2	1	0	0	0	0	0	0	0	0
Bre_lanc	D	2	0	0	1	0	0	0	0	10	7	15
Cal_baci	D	0	0	0	0	0	0	0	0	0	0	0
Cer_clos	D	0	1	0	8	3	5	16	0	2	0	0
Cha_spp.	D	25	28	35	14	12	1	5	4	2	4	1
Chp_marg	D	0	0	1	1	1	0	0	0	1	0	3
Coa_guil	D	0	0	0	0	0	0	0	0	0	0	0
Coc_neod	D	0	0	5	10	4	0	4	0	2	1	0
Coc_neot	D	0	0	0	0	0	2	1	0	0	1	2
Coc_pedi	D	0	0	0	3	7	40	11	5	0	2	4
Coc_pelt	D	0	0	0	0	0	0	0	0	0	0	2
Coc_plac	D	0	1	1	5	1	11	6	6	6	4	6
Coc_psth	D	1	0	0	2	2	0	0	0	0	0	0
Coc_scut	D	0	0	0	0	0	0	0	0	0	0	0
Con_weis	D	0	0	0	0	0	0	0	0	0	0	0
Cra_cusp	D	0	0	0	0	0	0	0	0	0	0	0
Cte_pulc	D	11	14	2	0	2	0	6	11	0	2	4
Cyb_amph	D	0	0	0	0	0	0	0	0	0	0	0
Cyc_atom	D	0	0	1	0	0	0	0	0	0	0	0
Cyc_choc	D	0	0	5	5	7	0	3	10	8	7	5
Cyc_mene	D	0	0	0	0	0	0	0	0	0	0	0
Cyc_radi	D	0	0	0	0	0	0	0	0	0	0	0
Cyc_stel	D	0	0	0	0	0	0	0	2	0	0	0
Cyc_stri	D	0	0	0	0	0	0	0	0	0	0	0
Cyl_grac	D	0	0	0	0	0	0	0	0	0	0	0
Cym_affi	D	0	0	0	0	0	0	0	0	0	0	0
Cym_cist	D	6	2	0	0	0	0	0	1	0	0	0
Cym_lanc	D	0	0	0	0	0	0	0	0	0	0	0
Den_cret	D	0	0	6	0	0	4	34	0	0	2	0
Den_sund	D	0	0	0	0	0	0	0	0	0	0	0
Dia_moni	D	73	81	79	42	68	2	281	49	14	104	2

Dia_tenu	D	5	10	8	0	0	5	2	3	10	1	2
Dia_vulg	D	42	42	16	1	0	2	0	0	2	2	2
Dip_domb	D	0	0	0	0	0	0	0	0	0	0	0
Dip_smit	D	0	0	0	0	0	0	1	0	0	0	0
Dip_smpu	D	0	0	0	0	0	0	0	0	0	0	0
Dip_smrh	D	0	0	0	0	0	0	0	0	0	0	0
Enc_caes	D	0	0	6	0	0	0	2	0	0	0	1
Enc_lacu	D	20	0	0	0	0	3	0	0	0	0	0
Enc_sile	D	0	0	0	0	0	0	0	0	0	0	0
Ent_cost	D	0	0	0	3	0	0	0	0	0	0	0
Ent_giga	D	0	0	1	0	0	0	1	0	0	0	0
Ent_palu	D	2	9	17	8	5	16	6	3	1	2	1
Ent_pseu	D	0	0	0	0	0	0	0	0	0	0	0
Ent_suri	D	1	0	0	0	0	1	0	0	0	0	0
Epi_adna	D	0	0	0	0	0	0	0	1	0	0	0
Epi_sore	D	2	1	1	7	2	13	2	4	5	0	6
Epi_tuwe	D	0	0	0	3	0	0	0	0	0	2	1
Fal_cryp	D	0	0	0	2	1	0	0	0	0	2	0
Fal_psli	D	0	0	0	0	0	0	0	0	0	0	0
Fal_pygm	D	0	0	0	0	0	0	0	0	0	0	0
Fra_amic	D	0	0	4	14	19	19	0	12	6	7	12
Fra_cava	D	0	0	0	0	2	0	0	0	0	0	0
Fra_cons	D	0	0	0	0	3	0	0	2	0	0	0
Fra_hydu	D	0	2	0	0	0	0	0	0	0	0	0
Fra_stri	D	5	18	29	4	7	4	0	0	0	2	0
Frp_cyli	D	0	0	0	0	0	0	0	0	0	0	0
Fru_creu	D	0	0	0	0	0	0	0	0	0	0	0
Gom_oliv	D	0	3	11	9	6	0	2	7	5	11	6
Gom_parv	D	0	0	0	0	0	0	0	0	0	0	0
Gon_exig	D	0	0	0	0	0	0	0	0	0	0	0
Gos_pseu	D	2	4	0	1	0	2	2	2	2	2	0
Gra_ocea	D	0	0	0	0	2	0	0	0	0	0	0
Gyr_exim	D	0	0	0	0	0	0	0	0	0	0	0
Gyr_fasc	D	0	0	0	0	0	0	0	0	0	0	0
Hal_acut	D	0	1	3	0	2	6	0	6	5	2	2
Hal_coff	D	0	11	6	2	7	7	0	15	10	8	5
Hal_exig	D	0	0	0	0	0	0	1	1	0	0	0
Hal_hols	D	0	0	0	2	0	0	0	0	0	0	0
Hal_hybr	D	0	0	0	0	0	0	0	0	0	0	0
Hal_luci	D	0	0	0	0	0	0	0	0	0	0	0
Hal_subh	D	0	0	2	0	0	1	0	0	0	0	1
Hal_tene	D	3	1	4	2	1	0	3	0	2	0	0
Hal_vene	D	0	0	0	0	0	0	0	0	0	0	0
Has_spic	D	0	0	0	5	4	9	4	1	0	0	1
Hip_hung	D	0	0	0	0	0	0	0	0	0	0	0
Hip_lesm	D	0	0	2	0	2	0	0	0	0	0	0
Hya_scot	D	0	0	0	1	2	0	0	0	0	0	0
Kar_amoe	D	0	0	0	0	0	0	0	0	0	0	0
Kar_clev	D	0	0	0	1	0	0	1	0	0	0	1
Lic_comm	D	0	0	0	0	0	0	0	0	0	0	0
Lic_debi	D	0	0	0	0	0	0	2	2	4	1	0
Lic_gran	D	0	0	0	0	0	0	0	0	0	0	0
Lic_rhom	D	0	0	0	0	0	0	0	0	0	0	0
Lun_bise	D	1	3	9	15	13	8	0	7	9	6	2
Lut_muti	D	0	0	0	0	0	0	0	0	0	0	0
Mar_atom	D	3	13	13	46	32	6	18	10	20	23	14
Mar_schu	D	0	0	0	0	0	0	0	0	0	0	0
Mas_balt	D	0	0	0	0	0	0	0	0	0	0	0
Mas_brau	D	0	0	0	0	0	0	0	0	0	0	0
Mas_elli	D	0	0	0	0	0	0	0	0	0	0	0
Mas_exig	D	0	0	0	0	0	0	0	0	0	0	0
Mas_pumi	D	2	0	0	0	0	2	0	0	0	0	0
Mas_smit	D	3	2	0	0	2	6	5	7	2	0	3
May_atom	D	0	0	0	0	0	0	0	0	0	0	0
Mel_arct	D	0	0	0	0	0	0	0	0	0	0	0
Mel_line	D	10	21	15	13	6	2	1	5	4	3	4
Mel_moni	D	0	2	3	2	1	5	0	0	2	4	4
Mel_numm	D	2	4	0	0	0	0	0	0	0	4	0
Mel_spec	D	87	98	48	7	25	114	128	221	326	292	146
Nav_aren	D	0	0	0	0	0	0	0	0	0	0	0
Nav_brem	D	0	0	0	1	0	0	0	0	0	0	0
Nav_cinc	D	0	0	0	0	0	0	0	0	0	0	0
Nav_clem	D	0	0	0	0	0	0	0	0	0	0	0
Nav_cryp	D	0	0	0	0	0	0	0	0	0	0	0
Nav_digi	D	0	0	0	0	0	0	0	0	0	0	0

Nav_duer	D	0	0	4	8	14	11	8	10	0	2	0
Nav_flan	D	0	0	0	0	0	0	0	0	0	0	0
Nav_greg	D	4	3	7	2	6	1	0	4	2	5	10
Nav_infi	D	0	0	1	0	3	4	0	9	10	4	5
Nav_lanc	D	0	2	1	1	0	0	0	0	0	2	0
Nav_meni	D	0	0	0	0	2	0	0	0	0	0	0
Nav_nole	D	0	0	0	0	0	0	0	0	0	0	0
Nav_pere	D	0	0	0	0	0	0	0	0	0	0	0
Nav_perm	D	60	66	39	48	50	47	41	43	31	23	69
Nav_phyl	D	4	2	2	2	4	1	2	0	1	0	1
Nav_rato	D	0	0	0	0	0	0	0	0	0	0	0
Nav_rhyn	D	0	0	0	0	0	0	0	0	0	0	0
Nav_saco	D	0	0	0	1	0	0	2	0	1	0	0
Nav_sana	D	0	0	0	0	0	0	0	0	0	0	0
Nav_sjoe	D	0	0	0	0	0	0	0	0	0	0	0
Nav_supr	D	0	2	0	2	0	0	0	0	0	0	0
Nav_trip	D	0	0	0	0	0	0	0	2	0	4	0
Nav_vene	D	0	0	0	0	0	0	0	0	0	0	0
Ncy_pusi	D	0	2	0	0	0	0	0	0	0	0	0
Nit_amph	D	0	0	0	0	0	0	0	0	0	0	0
Nit_anla	D	0	0	1	0	0	0	0	0	2	0	0
Nit_aura	D	0	0	0	0	0	0	0	0	0	0	0
Nit_brev	D	0	0	0	0	0	0	0	0	0	0	0
Nit_comm	D	0	0	0	0	0	0	0	0	0	0	0
Nit_diss	D	1	0	0	0	0	0	0	0	0	2	0
Nit_dubi	D	0	0	0	0	0	0	0	0	0	0	0
Nit_eleg	D	0	0	0	0	2	0	0	0	0	0	0
Nit_fili	D	0	0	2	0	2	0	0	0	0	0	0
Nit_flex	D	0	1	0	1	0	0	0	0	0	2	0
Nit_frus	D	103	70	73	84	85	183	148	106	76	62	242
Nit_gand	D	2	0	0	0	0	0	0	0	0	0	0
Nit_heuf	D	0	2	1	0	0	0	0	0	0	0	0
Nit_inco	D	0	1	1	5	0	2	0	0	0	0	0
Nit_lieb	D	0	0	9	2	15	8	8	7	6	10	2
Nit_lisu	D	0	2	0	0	0	0	0	0	0	0	0
Nit_lore	D	0	0	0	0	0	0	0	0	0	0	0
Nit_micr	D	8	23	22	20	37	46	13	15	24	5	22
Nit_pale	D	0	2	5	6	1	2	0	8	1	1	0
Nit_pate	D	0	0	0	0	1	2	0	5	0	0	0
Nit_pcea	D	14	16	23	58	136	22	4	31	14	7	1
Nit_pell	D	6	2	2	6	5	0	4	3	0	0	0
Nit_perm	D	0	0	0	0	0	0	0	0	0	0	0
Nit_pusi	D	10	32	19	4	1	0	0	2	6	0	2
Nit_reve	D	0	0	0	0	0	0	0	1	0	0	0
Nit_rose	D	2	13	14	36	60	57	31	52	48	26	21
Nit_sdea	D	0	0	0	0	0	0	0	0	0	0	0
Nit_sigm	D	0	0	0	3	0	0	0	0	0	0	0
Nit_soci	D	0	0	0	0	0	0	0	0	0	0	0
Nit_subc	D	0	0	0	0	0	0	0	0	0	0	0
Nit_supr	D	0	0	0	2	4	0	0	0	0	2	0
Nit_ther	D	2	8	11	3	2	8	0	8	1	7	2
Nit_vald	D	0	0	0	0	3	0	0	0	4	0	0
Ope_muta	D	5	8	16	26	42	5	7	11	13	31	15
Pau_taen	D	0	2	1	0	4	0	0	0	0	0	1
Pin_eleg	D	0	0	0	0	0	0	0	0	0	0	0
Pin_kroc	D	0	0	0	2	0	0	0	0	0	0	0
Pin_micr	D	0	0	0	0	0	0	0	0	0	0	0
Ple_elon	D	0	0	0	0	0	0	0	0	0	0	0
Ple_sali	D	0	0	0	0	0	0	0	0	0	0	0
Plt_deli	D	0	0	0	8	0	0	0	0	0	0	2
Plt_disp	D	0	0	0	0	0	0	0	0	0	0	0
Plt_freq	D	0	0	0	0	0	0	0	0	0	0	0
Plt_hauc	D	0	2	3	10	2	5	3	2	2	4	2
Plt_lemm	D	0	0	1	2	2	2	4	2	0	0	2
Plt_sept	D	0	0	0	0	0	0	0	0	0	0	0
Pro_buln	D	2	2	4	0	0	0	4	0	0	0	0
Pse_west	D	0	0	0	0	0	0	0	0	0	0	0
Psf_tene	D	0	0	0	0	0	0	0	0	0	2	0
Pss_brev	D	0	0	2	2	3	1	4	2	0	3	0
Pss_elli	D	12	30	127	229	146	55	52	84	75	71	48
Pss_zeil	D	4	4	25	48	22	12	14	14	6	10	27
Pst_punc	D	0	1	0	0	0	0	0	0	0	0	2
Ptd_gemm	D	0	0	0	0	0	0	0	0	0	0	0
Rei_sinu	D	0	0	0	0	0	0	0	0	0	0	0
Rho_abbr	D	29	28	17	43	8	121	30	17	15	27	79

Rhp_gibb	D	0	0	0	0	0	1	0	0	0	0	0
Rhp_gipa	D	0	0	0	0	0	0	0	0	0	0	0
Sel_pupu	D	0	0	0	0	2	0	0	0	0	0	0
Ske_cost	D	3	2	4	0	0	0	0	0	0	0	0
Std_hant	D	0	0	0	0	0	0	0	0	0	0	0
Std_minu	D	0	0	0	0	0	0	0	0	0	0	0
Stn_simu	D	0	0	0	0	0	0	0	0	0	0	0
Sur_breb	D	11	4	7	1	0	2	5	0	0	5	2
Syn_acus	D	0	0	0	0	0	0	0	0	0	0	0
Tab_fasc	D	44	33	15	16	13	3	23	22	26	36	23
Tab_tabu	D	4	0	0	0	0	0	11	6	2	22	2
Tab_waer	D	0	0	0	0	0	0	0	0	0	0	0
Tal_fene	D	0	0	0	0	0	0	0	0	0	0	0
Tal_floc	D	0	0	0	0	0	0	0	0	0	0	0
Tha_balt	D	0	0	0	3	0	0	0	1	0	1	0
Tha_leva	D	2	2	0	1	2	0	0	0	0	0	0
Tha_pros	D	0	0	0	0	0	0	0	10	0	3	2
Tha_pseu	D	0	0	1	0	0	2	1	1	1	0	0
Tro_dann	D	0	0	0	0	0	0	0	0	0	0	0
Try_angu	D	0	0	0	0	0	0	0	0	0	0	0
Try_apic	D	0	0	1	1	0	3	0	0	0	2	0
Try_ardu	D	0	0	0	2	0	0	0	0	0	0	0
Try_hung	D	0	0	0	0	0	0	0	0	0	0	0
Try_levi	D	0	0	0	0	0	0	0	0	0	0	0
Acd_minu	E	1	4	2	0	0	6	2	0	0	0	0
Acd_pyre	E	0	0	1	0	0	0	0	0	0	0	0
Acd_suba	E	0	0	0	4	0	0	0	0	0	0	0
Ach_bica	E	0	0	0	0	0	0	0	0	0	0	0
Ach_brev	E	0	0	0	0	0	0	0	0	1	0	2
Ach_brin	E	2	2	0	0	2	0	2	0	24	16	31
Ach_long	E	0	0	0	0	0	0	0	0	0	0	0
Ach_misc	E	2	2	4	10	2	0	6	6	0	2	6
Ach_vist	E	0	0	0	0	0	0	0	0	0	0	0
Act_occr	E	0	0	0	0	0	0	0	0	0	0	0
Amp_comm	E	0	1	0	0	0	0	0	0	0	0	0
Amp_copu	E	0	0	2	0	0	2	0	0	0	0	0
Amp_fleb	E	13	20	12	4	2	0	0	6	5	0	0
Amp_inar	E	0	0	0	0	0	0	0	0	0	0	0
Amp_line	E	0	2	0	0	0	0	0	0	0	0	0
Amp_micr	E	0	0	0	0	0	0	0	0	0	4	0
Amp_oval	E	0	0	0	0	0	0	0	0	0	0	0
Amp_pedi	E	9	22	5	2	6	8	1	4	2	1	10
Amp_stau	E	0	0	0	0	0	0	0	0	0	0	0
Ane_tusc	E	0	0	0	0	0	0	0	0	0	0	0
Asa_bahu	E	3	1	0	0	0	0	0	0	0	0	0
Ast_form	E	0	0	0	0	0	0	0	0	0	0	0
Aul_spp	E	0	0	0	0	0	1	0	0	0	0	2
Bac_paxi	E	0	5	4	0	4	7	21	16	15	122	62
Ber_ruti	E	30	32	15	10	5	3	18	13	4	39	30
Bra_apon	E	0	0	0	0	0	0	0	0	0	0	0
Bre_lanc	E	0	0	0	0	0	0	2	7	2	7	2
Cal_baci	E	0	7	2	1	1	4	0	2	0	0	2
Cer_clos	E	0	0	13	39	10	52	10	0	0	2	0
Cha_spp.	E	38	36	21	12	2	9	4	5	4	6	4
Chp_marg	E	0	0	0	1	2	2	0	7	0	0	2
Coa_guil	E	0	0	0	0	0	0	0	0	0	0	0
Coc_neod	E	10	0	3	2	2	10	4	2	3	0	2
Coc_neot	E	0	4	4	3	0	0	4	0	0	2	5
Coc_pedi	E	0	4	16	0	1	16	3	2	2	0	13
Coc_pelt	E	0	1	0	0	0	0	0	0	0	0	0
Coc_plac	E	5	31	8	5	7	10	4	4	3	4	8
Coc_psth	E	0	0	0	0	0	0	0	0	0	0	0
Coc_scut	E	0	0	0	0	1	0	0	0	0	2	0
Con_weis	E	0	0	0	0	0	0	0	0	0	0	0
Cra_cusp	E	0	0	0	0	0	0	0	0	0	0	0
Cte_pulc	E	2	7	0	0	0	0	6	0	0	4	0
Cyb_amph	E	0	0	0	0	0	0	0	0	0	0	0
Cyc_atom	E	0	0	0	1	0	2	0	0	0	0	0
Cyc_choc	E	0	0	2	1	0	7	2	2	4	4	4
Cyc_mene	E	0	0	0	0	0	0	0	0	0	0	0
Cyc_radi	E	0	0	0	0	0	0	0	0	0	0	0
Cyc_stel	E	0	0	0	2	0	0	0	1	0	1	0
Cyc_stri	E	0	0	0	0	0	0	0	0	0	0	0
Cyl_grac	E	0	0	0	0	0	0	0	0	0	0	0
Cym_affi	E	0	2	0	0	0	0	0	0	0	0	0

Cym_cist	E	0	0	0	0	0	0	0	0	0	2	0
Cym_lanc	E	2	0	0	0	0	0	0	0	0	2	0
Den_cret	E	0	0	0	11	0	2	0	0	8	0	0
Den_sund	E	0	0	0	0	0	0	0	0	0	0	0
Dia_moni	E	290	133	121	37	247	140	15	20	6	10	6
Dia_tenu	E	13	9	5	1	0	12	0	2	0	0	0
Dia_vulg	E	197	23	7	2	0	0	0	0	2	0	2
Dip_domb	E	0	0	0	0	0	0	0	0	0	0	0
Dip_smit	E	0	0	0	0	0	0	2	0	0	0	0
Dip_smpu	E	0	0	0	0	0	0	0	0	0	0	0
Dip_smrh	E	0	0	0	0	0	0	0	0	0	0	0
Enc_caes	E	0	0	0	0	0	0	0	0	0	0	0
Enc_lacu	E	0	0	0	0	0	0	0	0	0	0	0
Enc_sile	E	0	0	0	0	0	0	0	0	0	0	0
Ent_cost	E	0	0	0	0	0	0	0	0	0	0	0
Ent_giga	E	0	0	0	0	0	0	0	0	0	0	0
Ent_palu	E	0	0	6	10	2	5	1	3	0	4	0
Ent_pseu	E	0	0	0	0	0	0	0	0	0	0	0
Ent_suri	E	0	1	0	0	0	0	0	0	0	0	0
Epi_adna	E	0	0	0	0	0	1	0	2	0	0	0
Epi_sore	E	0	0	6	4	19	11	8	4	0	0	1
Epi_tuwe	E	0	0	1	2	2	0	0	4	0	0	6
Fal_cryp	E	0	7	2	1	0	2	0	4	1	0	2
Fal_psli	E	0	0	0	0	0	0	0	0	0	0	0
Fal_pygm	E	0	0	0	0	0	0	0	0	0	0	0
Fra_amic	E	2	8	16	13	6	20	1	13	5	2	0
Fra_cava	E	0	0	0	0	0	2	0	0	0	0	0
Fra_cons	E	0	2	0	0	1	0	0	1	1	4	0
Fra_hydu	E	0	0	0	0	0	0	0	0	0	0	0
Fra_stri	E	3	0	0	5	2	0	0	2	1	0	0
Frp_cyli	E	0	0	0	0	0	0	0	0	0	0	0
Fru_creu	E	0	0	0	0	0	0	0	0	0	0	0
Gom_oliv	E	15	14	4	14	0	0	0	1	0	2	4
Gom_parv	E	0	0	0	0	0	0	0	0	0	0	0
Gon_exig	E	0	0	0	0	0	0	0	0	0	0	0
Gos_pseu	E	2	5	0	0	0	0	0	1	2	0	0
Gra_ocea	E	0	0	0	0	0	0	0	0	0	0	0
Gyr_exim	E	0	0	0	0	0	0	0	0	0	0	0
Gyr_fasc	E	0	0	0	0	0	0	0	0	0	0	0
Hal_acut	E	0	0	2	2	0	1	1	2	1	0	1
Hal_coff	E	0	13	6	19	24	3	11	17	18	7	11
Hal_exig	E	0	2	0	2	0	0	0	0	2	0	1
Hal_hols	E	0	0	0	0	0	2	3	0	0	0	0
Hal_hybr	E	0	0	0	0	0	0	0	0	0	0	0
Hal_luci	E	0	0	0	0	0	0	0	0	0	0	0
Hal_subh	E	1	0	0	0	0	0	0	0	0	2	0
Hal_tene	E	0	0	0	0	23	0	12	0	8	0	4
Hal_vene	E	0	0	0	0	0	0	0	0	0	0	0
Has_spic	E	0	0	13	9	9	14	2	1	2	0	0
Hip_hung	E	2	0	0	0	0	0	0	0	0	0	0
Hip_lesm	E	0	0	2	0	6	0	3	7	2	0	0
Hya_scot	E	0	0	0	0	0	0	0	0	0	0	0
Kar_amoe	E	2	0	0	0	0	0	0	0	0	0	3
Kar_clev	E	0	0	0	0	2	0	1	4	1	4	2
Lic_comm	E	0	0	0	0	0	0	0	0	0	0	0
Lic_debi	E	0	0	0	0	12	2	0	0	0	0	0
Lic_gran	E	1	0	0	0	0	0	0	0	0	2	0
Lic_rhom	E	0	0	0	0	0	0	0	0	0	0	0
Lun_bise	E	2	0	4	6	12	4	1	2	0	0	2
Lut_muti	E	0	0	0	0	0	0	0	0	0	0	0
Mar_atom	E	14	11	38	24	22	31	21	32	19	21	4
Mar_schu	E	0	0	0	0	0	0	0	0	0	0	0
Mas_balt	E	0	0	0	0	0	0	0	0	0	0	0
Mas_brau	E	0	0	0	0	0	0	0	0	0	0	0
Mas_elli	E	0	0	0	0	1	4	0	0	0	0	0
Mas_exig	E	0	0	0	0	0	0	0	0	0	0	0
Mas_pumi	E	0	0	0	0	0	0	0	0	0	0	0
Mas_smit	E	0	0	6	0	2	5	2	0	0	0	0
May_atom	E	0	0	0	0	0	0	0	0	0	0	0
Mel_arct	E	0	0	0	0	0	0	0	0	0	0	0
Mel_line	E	4	16	2	4	0	2	2	1	0	4	12
Mel_moni	E	4	21	5	10	2	6	3	8	1	9	95
Mel_numm	E	2	2	0	9	25	0	0	9	111	46	191
Mel_spec	E	28	30	0	29	29	17	325	140	180	160	55
Nav_aren	E	0	0	0	0	0	0	0	0	0	0	0

Nav_brem	E	0	0	0	0	0	2	0	0	0	0	0
Nav_cinc	E	0	0	0	0	6	0	0	0	0	0	0
Nav_clem	E	0	0	0	0	0	0	0	0	0	0	0
Nav_cryp	E	0	0	0	0	0	0	0	0	0	0	0
Nav_digi	E	0	0	0	0	1	0	0	0	0	0	0
Nav_duer	E	0	4	8	0	0	2	0	4	0	0	0
Nav_flan	E	0	0	0	0	0	0	0	1	0	0	0
Nav_greg	E	6	8	4	6	9	1	2	2	2	2	0
Nav_infi	E	6	2	17	32	6	33	10	16	4	10	0
Nav_lanc	E	2	0	0	0	0	0	0	4	1	4	0
Nav_meni	E	0	0	0	0	0	0	0	0	0	0	0
Nav_nole	E	0	0	0	0	0	0	0	0	0	0	0
Nav_pere	E	0	0	0	0	0	0	0	0	0	0	0
Nav_perm	E	89	151	68	68	81	30	77	124	112	109	73
Nav_phyl	E	0	0	2	4	0	3	0	4	2	2	0
Nav_rato	E	0	4	5	7	0	2	0	0	0	2	0
Nav_rhyn	E	0	0	0	0	0	0	0	0	0	0	0
Nav_saco	E	0	0	0	0	0	0	0	0	0	0	0
Nav_sana	E	0	0	0	1	2	0	0	2	0	0	0
Nav_sjoe	E	2	0	0	0	0	0	0	0	0	0	0
Nav_supr	E	0	0	0	1	0	0	0	0	0	0	0
Nav_trip	E	2	0	0	0	0	0	0	0	0	0	0
Nav_vene	E	0	0	0	4	0	0	0	0	3	2	0
Ncy_pusi	E	0	0	0	0	0	0	0	0	0	0	0
Nit_amph	E	0	0	0	0	0	0	0	1	0	0	0
Nit_anla	E	2	0	0	0	0	1	0	0	0	0	0
Nit_aura	E	0	0	0	0	0	0	0	0	0	0	0
Nit_brev	E	0	0	0	0	0	0	0	0	0	0	0
Nit_comm	E	0	0	0	0	0	0	0	0	0	0	0
Nit_diss	E	0	0	0	0	0	0	0	0	0	0	0
Nit_dubi	E	0	0	0	0	0	0	0	0	2	0	0
Nit_eleg	E	0	0	0	0	0	0	0	0	0	0	0
Nit_fili	E	5	3	7	57	12	0	4	31	159	117	124
Nit_flex	E	0	0	0	0	0	0	0	0	0	0	0
Nit_frus	E	30	128	57	87	58	41	80	54	78	27	68
Nit_gand	E	0	0	0	0	0	0	0	0	0	0	0
Nit_heuf	E	0	0	0	0	0	0	0	0	0	0	0
Nit_inco	E	0	0	0	0	0	0	0	0	0	0	0
Nit_lieb	E	2	0	0	2	2	2	0	0	0	0	1
Nit_lisu	E	2	0	0	0	0	0	0	0	0	0	0
Nit_lore	E	0	0	0	0	0	0	0	0	0	0	0
Nit_micr	E	4	4	31	20	12	6	26	22	15	11	12
Nit_pale	E	0	4	0	4	0	1	0	2	4	4	0
Nit_pate	E	0	0	0	0	0	0	0	0	0	0	0
Nit_pcea	E	3	30	71	47	35	8	0	7	0	2	0
Nit_pell	E	0	0	0	13	0	2	0	0	2	34	0
Nit_perm	E	0	0	0	0	0	0	0	0	0	0	0
Nit_pusi	E	0	2	7	76	8	0	2	0	0	0	0
Nit_reve	E	0	0	0	0	0	0	0	2	0	0	0
Nit_rose	E	9	29	81	53	97	38	17	40	32	33	2
Nit_sdea	E	0	0	0	0	0	0	0	0	0	0	0
Nit_sigm	E	0	0	0	0	0	0	0	0	0	0	2
Nit_soci	E	0	0	0	0	0	0	0	0	0	0	0
Nit_subc	E	0	0	0	0	0	0	0	0	0	0	0
Nit_supr	E	0	0	2	0	6	0	10	0	0	2	2
Nit_ther	E	1	3	3	2	2	2	0	0	0	20	0
Nit_vald	E	0	5	0	2	3	6	0	0	0	2	0
Ope_muta	E	3	21	17	27	5	19	28	49	11	10	13
Pau_taen	E	0	1	0	1	0	0	0	0	0	0	0
Pin_eleg	E	0	0	0	0	0	0	0	0	0	0	0
Pin_kroc	E	0	0	0	0	0	0	0	0	0	0	0
Pin_micr	E	0	0	0	0	0	0	0	0	0	0	0
Ple_elon	E	0	0	0	0	0	0	0	1	0	0	0
Ple_sali	E	0	0	0	0	0	2	10	0	2	0	2
Plt_deli	E	1	3	2	4	1	0	4	0	0	3	0
Plt_disp	E	0	0	0	0	0	0	0	0	0	0	0
Plt_freq	E	0	0	0	0	0	0	0	0	0	0	0
Plt_hauc	E	0	4	3	2	2	6	4	5	4	0	4
Plt_lemm	E	0	0	0	0	2	0	0	2	0	0	3
Plt_sept	E	1	1	2	2	0	9	0	0	4	0	2
Pro_buln	E	2	0	2	2	0	23	8	7	4	5	0
Pse_west	E	0	0	0	0	0	0	0	0	0	0	0
Psf_tene	E	0	0	0	0	0	0	0	0	0	0	0
Pss_brev	E	0	2	2	1	2	5	5	4	0	2	0
Pss_elli	E	45	51	142	95	103	183	125	158	64	25	69

Pss_zeil	E	8	0	39	27	7	19	16	28	23	5	5
Pst_punc	E	1	0	2	1	0	0	0	0	1	0	0
Ptd_gemm	E	0	0	0	0	0	0	0	0	0	0	0
Rei_sinu	E	0	0	0	0	0	0	0	0	0	0	0
Rho_abbr	E	22	39	22	25	16	32	7	15	6	10	14
Rhp_gibb	E	0	0	4	1	0	0	0	0	0	0	1
Rhp_gipa	E	0	0	0	0	0	0	0	0	0	0	0
Sel_pupu	E	0	0	0	0	0	0	0	0	0	0	0
Ske_cost	E	7	3	2	4	0	0	0	0	0	0	0
Std_hant	E	0	0	1	0	0	0	0	0	0	0	0
Std_minu	E	0	0	0	0	0	0	0	0	0	0	0
Stn_simu	E	0	0	0	0	0	0	0	0	0	0	0
Sur_breb	E	1	2	6	1	0	2	0	2	0	13	0
Syn_acus	E	0	0	0	0	0	0	0	0	0	0	0
Tab_fasc	E	38	10	24	9	25	88	59	31	16	9	5
Tab_tabu	E	3	6	0	0	0	2	3	0	5	37	9
Tab_waer	E	0	0	0	0	0	0	0	0	0	0	0
Tal_fene	E	0	0	0	0	0	0	0	0	0	0	0
Tal_floc	E	0	0	0	0	0	0	0	0	0	0	0
Tha_balt	E	2	0	1	1	0	0	0	2	2	0	0
Tha_leva	E	2	0	3	0	2	1	0	0	0	0	0
Tha_pros	E	0	0	0	0	0	0	0	20	2	1	0
Tha_pseu	E	0	0	0	0	0	4	0	3	0	0	2
Tro_dann	E	0	0	0	0	0	0	0	0	0	0	0
Try_angu	E	0	0	0	0	0	0	0	0	0	0	0
Try_apic	E	0	0	1	0	0	0	0	0	0	0	0
Try_ardu	E	0	0	2	0	0	0	0	0	0	0	0
Try_hung	E	0	0	0	0	0	0	0	0	0	0	0
Try_levi	E	0	0	0	0	0	0	0	0	0	0	0
Acd_minu	F	0	4	5	2	2	0	2	0	0	2	2
Acd_pyre	F	0	0	1	0	0	0	0	0	0	0	0
Acd_suba	F	0	0	0	0	0	0	0	0	0	0	0
Ach_bica	F	0	2	0	0	0	0	0	0	0	0	0
Ach_brev	F	0	2	0	0	2	0	3	0	0	0	0
Ach_brin	F	0	0	0	0	1	12	34	7	105	3	155
Ach_long	F	0	0	0	0	0	0	0	0	0	0	0
Ach_misc	F	2	2	6	4	7	6	18	0	2	6	0
Ach_vist	F	0	1	0	0	0	1	0	0	0	0	0
Act_occr	F	0	0	0	0	0	0	0	0	0	0	0
Amp_comm	F	0	0	0	0	1	0	0	0	0	0	0
Amp_copu	F	0	1	0	0	0	0	0	0	0	0	2
Amp_fleb	F	7	18	11	13	1	0	3	2	1	24	2
Amp_inar	F	0	0	0	2	0	0	0	0	0	0	0
Amp_line	F	0	0	0	0	0	0	0	0	0	0	0
Amp_micr	F	0	0	0	0	0	0	0	0	0	0	0
Amp_oval	F	0	0	0	0	0	0	0	0	0	0	5
Amp_pedi	F	6	10	11	5	9	13	16	6	5	2	6
Amp_stau	F	0	0	0	0	0	0	0	0	0	0	0
Ane_tusc	F	0	0	0	0	0	0	0	0	0	0	0
Asa_bahu	F	0	0	0	0	0	0	0	0	0	0	0
Ast_form	F	0	0	1	0	0	0	0	0	0	0	0
Aul_spp	F	0	0	0	0	0	0	0	0	0	1	0
Bac_paxi	F	0	0	0	2	0	35	96	42	4	26	0
Ber_ruti	F	244	41	16	16	6	33	62	136	10	12	0
Bra_apon	F	0	0	0	0	0	0	0	0	0	0	0
Bre_lanc	F	2	0	0	0	0	2	0	3	2	0	0
Cal_baci	F	0	1	2	0	2	0	0	0	0	0	8
Cer_clos	F	4	7	8	6	33	10	8	0	0	11	0
Cha_spp.	F	46	60	52	17	5	3	8	3	0	5	3
Chp_marg	F	2	12	4	4	4	2	4	4	0	0	0
Coa_guil	F	0	4	0	0	0	0	0	2	0	0	0
Coc_neod	F	0	0	7	2	3	3	5	5	7	0	0
Coc_neot	F	0	2	0	0	0	4	0	0	18	0	11
Coc_pedi	F	0	2	23	7	0	12	5	2	6	4	26
Coc_pelt	F	0	0	0	1	2	0	0	0	0	0	0
Coc_plac	F	5	6	9	6	3	16	10	4	13	4	13
Coc_psth	F	0	0	0	1	0	0	1	0	0	0	0
Coc_scut	F	1	0	0	0	0	2	0	1	0	1	2
Con_weis	F	0	0	0	0	0	0	0	0	0	0	0
Cra_cusp	F	0	0	0	0	0	0	0	0	0	0	0
Cte_pulc	F	7	0	1	3	0	10	0	0	2	0	2
Cyb_amph	F	0	0	0	0	0	0	0	0	0	0	0
Cyc_atom	F	0	0	0	0	2	0	0	3	2	0	0
Cyc_choc	F	1	2	1	8	5	3	2	11	3	2	6
Cyc_mene	F	0	0	0	0	0	0	0	0	0	0	0

Cyc_radi	F	0	1	0	0	0	0	0	0	0	2	0
Cyc_stel	F	0	0	0	0	0	0	0	0	0	0	0
Cyc_stri	F	0	0	0	0	0	0	0	0	0	0	0
Cyl_grac	F	0	0	0	2	0	0	0	0	0	0	0
Cym_affi	F	0	0	0	0	0	0	0	0	0	0	0
Cym_cist	F	0	0	0	0	0	0	0	0	0	0	0
Cym_lanc	F	0	0	0	0	0	0	0	0	1	4	0
Den_cret	F	3	13	2	17	0	0	0	0	0	0	0
Den_sund	F	0	0	0	0	0	0	0	0	0	0	0
Dia_moni	F	283	129	66	95	128	10	4	1	2	22	2
Dia_tenu	F	6	6	18	1	2	2	0	2	0	2	0
Dia_vulg	F	105	53	2	0	2	0	0	0	0	4	0
Dip_domb	F	0	0	0	0	0	0	0	0	0	0	0
Dip_smit	F	0	0	0	0	0	0	0	0	0	0	0
Dip_smpu	F	0	0	0	0	0	0	0	0	0	0	0
Dip_smrh	F	0	0	0	0	0	0	0	0	0	0	0
Enc_caes	F	0	0	0	0	0	0	0	0	0	0	0
Enc_lacu	F	0	0	0	0	0	0	0	0	0	0	0
Enc_sile	F	0	0	0	0	0	0	0	0	0	0	0
Ent_cost	F	0	0	0	0	0	0	0	0	0	0	0
Ent_giga	F	0	0	0	0	0	0	0	0	0	0	0
Ent_palu	F	0	3	3	4	4	1	10	0	1	7	0
Ent_pseu	F	0	0	0	0	0	0	0	0	0	0	0
Ent_suri	F	0	0	1	0	0	0	0	0	0	0	0
Epi_adna	F	0	0	0	0	0	2	0	0	0	0	0
Epi_sore	F	0	1	5	2	1	70	8	5	2	0	5
Epi_tuwe	F	0	0	0	0	0	0	2	0	0	0	2
Fal_cryp	F	0	1	1	0	0	0	0	0	2	0	0
Fal_psli	F	0	0	0	0	0	0	2	0	0	0	0
Fal_pygm	F	0	0	0	0	0	0	0	0	0	0	0
Fra_amic	F	0	2	12	25	12	14	10	25	0	10	3
Fra_cava	F	0	2	0	0	0	0	0	2	0	0	0
Fra_cons	F	0	2	0	0	0	0	2	0	1	2	0
Fra_hydu	F	0	0	0	0	0	0	0	0	0	0	0
Fra_stri	F	4	2	5	1	0	0	0	1	0	0	0
Frp_cyli	F	0	0	0	0	0	0	0	0	0	0	0
Fru_creu	F	0	0	0	0	0	0	0	0	0	0	0
Gom_oliv	F	7	15	3	7	16	8	5	2	2	6	0
Gom_parv	F	0	0	0	0	0	0	0	0	0	0	0
Gon_exig	F	0	0	0	0	0	2	0	0	0	0	0
Gos_pseu	F	0	4	4	0	0	0	2	2	2	0	12
Gra_ocea	F	0	0	0	0	0	0	0	0	0	0	0
Gyr_exim	F	0	0	0	0	0	0	0	0	0	0	0
Gyr_fasc	F	0	0	0	0	0	0	0	0	0	0	0
Hal_acut	F	0	5	0	3	0	2	1	4	0	2	0
Hal_coff	F	0	0	0	29	2	12	25	33	4	3	1
Hal_exig	F	0	0	0	0	1	2	21	4	5	3	4
Hal_hols	F	0	0	0	0	0	0	0	0	0	1	0
Hal_hybr	F	0	0	0	0	0	0	0	0	0	0	0
Hal_luci	F	0	0	0	0	0	0	0	0	0	0	0
Hal_subh	F	2	0	0	1	0	6	0	0	2	0	4
Hal_tene	F	0	0	3	0	0	2	1	1	2	0	0
Hal_vene	F	0	0	0	0	0	0	0	0	0	0	0
Has_spic	F	0	0	3	2	4	8	2	0	0	1	0
Hip_hung	F	0	0	2	0	0	0	0	0	0	0	0
Hip_lesm	F	0	4	1	0	2	0	0	0	0	0	0
Hya_scot	F	0	2	0	2	3	1	3	0	0	0	0
Kar_amoe	F	0	0	2	0	1	9	0	0	15	0	79
Kar_clev	F	0	0	2	4	3	1	5	4	1	0	0
Lic_comm	F	0	0	0	0	0	0	0	0	0	0	0
Lic_debi	F	0	0	6	0	0	0	1	1	0	2	0
Lic_gran	F	0	0	0	0	0	0	0	0	0	0	0
Lic_rhom	F	0	0	0	0	0	0	0	0	0	0	0
Lun_bise	F	8	8	2	15	2	3	6	2	1	2	0
Lut_muti	F	0	0	0	0	0	0	0	0	0	0	0
Mar_atom	F	5	33	39	38	51	28	33	39	12	29	8
Mar_schu	F	0	0	0	0	0	0	0	0	0	0	0
Mas_balt	F	0	0	0	0	0	0	0	0	0	0	0
Mas_brau	F	0	0	0	0	0	0	0	0	0	0	0
Mas_elli	F	0	0	0	0	0	0	0	0	0	0	0
Mas_exig	F	0	0	0	0	0	0	0	0	0	0	0
Mas_pumi	F	0	0	0	0	0	2	1	2	0	0	0
Mas_smit	F	0	0	7	0	0	8	2	0	2	0	0
May_atom	F	0	0	0	0	0	0	0	0	0	0	0
Mel_arct	F	0	0	0	0	0	0	0	0	0	0	0

Mel_line	F	0	9	10	2	0	15	4	0	9	1	20
Mel_moni	F	13	17	3	1	1	2	1	0	29	0	21
Mel_numm	F	0	0	0	0	0	0	0	0	0	0	0
Mel_spec	F	1	0	2	4	95	50	129	125	62	62	13
Nav_aren	F	0	0	0	0	0	0	0	0	0	0	0
Nav_brem	F	0	0	0	0	0	0	0	0	0	2	0
Nav_cinc	F	0	0	0	0	0	0	0	0	0	0	0
Nav_clem	F	0	0	0	0	0	0	0	0	0	0	0
Nav_cryp	F	0	0	0	0	0	2	0	0	0	0	2
Nav_digi	F	0	0	0	0	0	0	0	0	0	0	0
Nav_duer	F	0	0	0	6	4	2	3	2	0	6	0
Nav_flan	F	0	0	0	1	0	0	0	0	0	0	0
Nav_greg	F	4	1	3	5	0	2	0	2	2	11	0
Nav_infi	F	1	4	6	6	7	14	4	4	0	17	0
Nav_lanc	F	0	0	0	0	0	0	1	0	0	7	0
Nav_meni	F	0	0	0	0	0	0	0	0	0	0	0
Nav_nole	F	0	0	0	0	0	0	2	0	0	0	0
Nav_pere	F	0	0	0	0	0	0	0	0	0	0	0
Nav_perm	F	48	59	45	43	19	2	16	23	74	132	79
Nav_phyl	F	0	4	0	4	0	2	5	2	0	2	0
Nav_rato	F	2	2	0	2	0	0	6	2	0	10	0
Nav_rhyn	F	0	0	0	0	0	0	0	0	0	0	0
Nav_saco	F	0	0	0	0	0	0	0	0	0	0	0
Nav_sana	F	0	0	0	0	0	0	0	0	0	0	0
Nav_sjoe	F	0	0	0	0	0	0	0	2	0	0	0
Nav_supr	F	0	0	1	1	0	0	0	0	0	0	0
Nav_trip	F	0	0	4	0	0	0	0	0	0	0	0
Nav_vene	F	0	0	0	0	0	0	0	0	0	0	0
Ncy_pusi	F	0	0	0	0	0	0	0	0	0	0	0
Nit_amph	F	0	0	0	0	0	0	0	0	0	0	2
Nit_anla	F	0	2	2	2	0	2	0	0	0	3	0
Nit_aura	F	0	0	0	0	0	0	0	0	0	0	0
Nit_brev	F	0	0	0	0	0	0	0	0	0	0	0
Nit_comm	F	0	0	0	0	0	0	0	0	0	0	0
Nit_diss	F	0	0	0	0	0	0	0	0	0	0	0
Nit_dubi	F	0	0	0	0	0	0	0	0	0	0	0
Nit_eleg	F	0	0	0	0	0	0	0	0	0	0	0
Nit_fili	F	0	2	0	0	0	0	9	55	25	24	34
Nit_flex	F	0	0	0	0	0	0	1	0	0	6	0
Nit_frus	F	11	69	82	52	125	61	58	68	320	46	262
Nit_gand	F	0	0	0	0	0	0	0	2	0	2	0
Nit_heuf	F	0	0	0	0	0	0	0	0	0	0	0
Nit_inco	F	0	2	0	0	2	0	0	0	2	0	0
Nit_lieb	F	0	0	4	2	0	2	5	4	15	2	0
Nit_lisu	F	0	0	0	0	0	0	0	0	0	0	0
Nit_lore	F	0	0	0	0	0	0	0	0	0	0	0
Nit_micr	F	0	4	3	7	16	3	11	9	13	4	5
Nit_pale	F	0	5	6	0	0	0	2	0	0	3	0
Nit_pate	F	0	0	0	0	0	0	0	0	0	0	0
Nit_pcea	F	15	45	99	57	138	36	15	3	0	11	0
Nit_pell	F	0	10	0	7	4	4	6	1	0	107	0
Nit_perm	F	0	4	0	5	0	0	1	0	0	2	0
Nit_pusi	F	3	2	0	0	0	0	0	0	0	0	2
Nit_reve	F	0	0	0	0	0	0	3	0	0	2	0
Nit_rose	F	9	13	38	43	79	34	46	16	9	101	5
Nit_sdea	F	0	0	0	0	0	0	0	0	0	0	0
Nit_sigm	F	0	0	0	2	0	0	0	0	0	0	0
Nit_soci	F	0	0	0	0	0	0	0	2	0	0	0
Nit_subc	F	0	0	0	0	0	0	0	0	0	0	0
Nit_supr	F	0	0	0	0	4	2	4	0	0	5	0
Nit_ther	F	11	11	0	3	3	1	0	0	0	37	0
Nit_vald	F	2	0	3	2	2	4	4	4	5	2	6
Ope_muta	F	11	22	21	56	19	20	30	32	3	22	6
Pau_taen	F	2	8	2	2	0	0	0	0	0	2	0
Pin_eleg	F	0	0	0	0	0	0	0	0	0	0	0
Pin_kroc	F	0	0	0	0	0	2	0	0	0	0	0
Pin_micr	F	0	0	0	0	0	0	0	0	0	0	0
Ple_elon	F	0	0	0	0	0	0	0	2	0	0	0
Ple_sali	F	0	0	0	0	0	0	0	0	0	0	0
Plt_deli	F	2	3	2	0	0	1	0	0	1	1	3
Plt_disp	F	0	0	0	0	0	0	0	0	0	0	0
Plt_freq	F	0	0	0	2	0	0	2	0	0	0	0
Plt_hauc	F	0	4	6	2	4	7	12	2	6	7	5
Plt_lemm	F	1	8	3	7	0	2	1	1	0	0	0
Plt_sept	F	0	0	0	0	0	0	0	0	0	2	2

Pro_buln	F	6	5	0	2	0	0	0	2	0	2	0
Pse_west	F	0	0	0	0	0	0	0	0	0	0	0
Psf_tene	F	0	0	0	0	2	0	0	0	0	0	0
Pss_brev	F	2	2	2	4	1	6	1	5	2	4	0
Pss_elli	F	26	125	183	264	96	156	131	187	22	97	21
Pss_zeil	F	17	39	18	36	22	16	18	18	3	4	0
Pst_punc	F	0	2	0	3	0	4	0	0	0	0	0
Ptd_gemm	F	0	0	0	0	0	0	0	0	0	0	0
Rei_sinu	F	0	0	0	0	0	0	0	0	0	0	0
Rho_abbr	F	28	26	64	7	27	98	14	17	117	10	137
Rhp_gibb	F	0	1	0	0	0	17	1	1	0	0	0
Rhp_gipa	F	0	0	0	0	0	0	0	0	0	0	0
Sel_pupu	F	0	0	0	0	0	0	0	0	0	0	0
Ske_cost	F	4	5	6	3	0	0	0	0	0	0	0
Std_hant	F	0	0	0	0	0	0	0	0	0	0	0
Std_minu	F	0	0	0	0	0	0	0	0	0	0	0
Stn_simu	F	0	0	0	0	0	0	0	0	0	0	0
Sur_breb	F	2	2	2	0	0	0	0	0	0	3	0
Syn_acus	F	0	0	0	0	0	0	0	0	0	0	0
Tab_fasc	F	16	13	28	3	4	58	25	34	24	26	8
Tab_tabu	F	4	0	1	1	0	5	0	3	12	0	3
Tab_waer	F	0	0	0	0	0	0	0	0	0	0	0
Tal_fene	F	0	0	0	0	0	0	0	0	1	0	0
Tal_floc	F	0	0	0	0	0	0	0	0	0	0	0
Tha_balt	F	2	0	0	1	0	2	3	1	0	3	0
Tha_leva	F	5	2	1	1	2	2	0	0	0	1	0
Tha_pros	F	0	0	0	0	0	0	1	5	3	0	1
Tha_pseu	F	2	3	1	0	2	0	0	3	0	2	0
Tro_dann	F	0	0	0	0	0	0	0	0	0	0	0
Try_angu	F	0	0	0	0	0	0	0	0	0	0	0
Try_apic	F	0	2	2	0	0	4	2	0	2	2	0
Try_ardu	F	0	0	0	0	0	0	0	0	0	0	0
Try_hung	F	0	0	0	0	0	0	0	0	0	0	0
Try_levi	F	0	0	0	2	0	0	0	0	2	0	0
Acd_minu	G	2	2	8	3	5	8	2	3	0	2	2
Acd_pyre	G	0	0	0	0	0	3	1	0	0	0	0
Acd_suba	G	0	0	0	0	0	0	0	0	0	0	0
Ach_bica	G	0	0	0	0	0	0	0	0	0	0	0
Ach_brev	G	0	3	2	1	1	2	1	0	0	0	0
Ach_brin	G	0	0	0	0	0	0	0	0	0	0	0
Ach_long	G	0	0	0	0	0	0	0	0	0	0	0
Ach_misc	G	1	4	8	8	7	32	12	6	5	4	4
Ach_vist	G	0	0	0	0	2	0	0	0	0	0	0
Act_occr	G	0	0	0	0	0	0	0	0	0	0	0
Amp_comm	G	0	0	0	0	0	0	0	0	0	0	0
Amp_copu	G	0	0	0	0	2	0	0	0	2	0	2
Amp_fleb	G	0	3	11	7	33	4	12	0	6	0	4
Amp_inar	G	0	0	0	0	0	0	0	0	0	0	1
Amp_line	G	0	0	0	0	0	1	0	0	0	0	0
Amp_micr	G	0	0	0	0	0	0	0	0	0	0	0
Amp_oval	G	0	0	0	0	0	0	0	0	0	0	0
Amp_pedi	G	4	7	13	6	8	16	20	15	7	12	3
Amp_stau	G	0	0	0	0	0	0	0	0	0	0	0
Ane_tusc	G	0	0	0	0	0	0	0	0	0	0	0
Asa_bahu	G	0	0	0	0	2	3	0	0	0	0	0
Ast_form	G	0	0	0	0	0	0	0	0	0	0	0
Aul_spp	G	0	0	0	0	0	0	7	1	3	0	2
Bac_paxi	G	0	0	0	0	5	29	33	48	86	89	171
Ber_ruti	G	237	219	40	11	40	4	27	22	188	67	62
Bra_apon	G	0	0	0	0	0	0	0	0	0	0	0
Bre_lanc	G	0	2	0	0	0	0	0	2	0	0	0
Cal_baci	G	2	2	0	2	2	0	1	7	2	0	4
Cer_clos	G	0	3	3	9	7	6	12	22	0	6	1
Cha_spp.	G	25	70	31	27	14	10	10	6	7	4	9
Chp_marg	G	0	3	0	2	8	1	7	0	1	7	0
Coa_guil	G	0	0	0	0	0	0	0	0	0	0	2
Coc_neod	G	2	3	5	2	8	12	9	3	2	6	5
Coc_neot	G	0	0	0	2	0	0	0	0	0	1	0
Coc_pedi	G	0	0	2	1	3	7	2	3	1	1	1
Coc_pelt	G	0	0	0	0	1	0	0	0	0	0	0
Coc_plac	G	0	2	4	0	3	5	2	5	1	5	4
Coc_psth	G	0	0	0	1	0	0	0	0	0	0	1
Coc_scut	G	0	0	1	0	0	0	2	0	1	0	1
Con_weis	G	0	0	0	0	0	0	0	0	0	0	0
Cra_cusp	G	0	0	0	0	0	0	0	0	0	0	0

Cte_pulc	G	10	2	0	3	2	0	0	0	0	0	0
Cyb_amph	G	0	0	0	0	0	1	0	0	0	0	0
Cyc_atom	G	0	0	0	1	0	0	0	0	0	0	0
Cyc_choc	G	0	1	3	5	9	2	4	13	8	8	16
Cyc_mene	G	0	0	0	0	0	0	0	0	0	1	0
Cyc_radi	G	0	0	0	0	0	0	0	0	0	0	0
Cyc_stel	G	0	0	0	0	0	0	0	0	0	0	0
Cyc_stri	G	0	0	0	0	0	0	2	0	0	0	0
Cyl_grac	G	0	0	0	0	0	0	0	0	0	0	0
Cym_affi	G	0	0	0	0	0	0	2	0	0	0	0
Cym_cist	G	0	0	0	0	0	0	0	0	0	0	0
Cym_lanc	G	0	0	0	0	0	0	0	2	0	0	0
Den_cret	G	0	0	0	0	0	0	0	0	0	0	0
Den_sund	G	0	0	0	0	0	0	0	0	0	0	0
Dia_moni	G	206	170	57	33	40	5	30	47	13	81	8
Dia_tenu	G	0	4	2	0	1	4	6	8	26	88	53
Dia_vulg	G	45	17	9	0	2	0	0	2	6	15	15
Dip_domb	G	0	0	0	0	0	0	0	0	0	0	0
Dip_smit	G	0	0	0	0	0	0	0	0	0	0	0
Dip_smpu	G	0	0	0	0	0	0	0	0	0	0	0
Dip_smrh	G	0	0	0	0	0	0	0	0	0	0	0
Enc_caes	G	0	0	0	0	0	0	0	0	0	0	0
Enc_lacu	G	0	0	0	0	0	0	0	0	0	0	0
Enc_sile	G	0	0	0	0	0	0	0	0	0	0	0
Ent_cost	G	0	0	0	0	0	2	0	0	0	0	0
Ent_giga	G	0	0	0	0	0	0	0	0	1	0	0
Ent_palu	G	2	5	10	6	10	11	12	9	2	3	1
Ent_pseu	G	0	0	0	0	2	1	0	2	0	0	0
Ent_suri	G	0	0	0	0	0	0	0	0	0	0	0
Epi_adna	G	0	0	0	0	1	2	2	0	0	0	0
Epi_sore	G	0	0	0	4	3	10	9	1	2	4	2
Epi_tuwe	G	0	0	0	0	0	0	4	0	2	0	0
Fal_cryp	G	0	2	0	2	4	1	0	1	0	2	3
Fal_psli	G	0	0	0	0	0	0	0	0	0	0	0
Fal_pygm	G	0	0	0	0	0	0	0	0	0	0	0
Fra_amic	G	5	12	14	26	11	20	8	16	11	5	13
Fra_cava	G	0	0	0	0	0	0	0	0	0	0	0
Fra_cons	G	0	2	0	0	3	0	0	2	0	0	2
Fra_hydu	G	0	0	0	0	0	0	0	0	0	0	0
Fra_stri	G	0	0	0	4	1	2	0	2	0	0	0
Frp_cyli	G	0	0	0	0	0	0	0	0	0	0	2
Fru_creu	G	0	0	0	0	0	0	0	0	0	0	0
Gom_oliv	G	102	2	6	3	1	0	0	2	3	0	2
Gom_parv	G	0	0	0	0	0	0	0	0	0	0	0
Gon_exig	G	0	0	0	0	0	0	0	0	0	0	0
Gos_pseu	G	0	1	1	2	0	0	1	0	4	0	0
Gra_ocea	G	0	0	0	0	0	0	0	0	0	0	0
Gyr_exim	G	0	0	0	0	0	0	0	0	0	0	0
Gyr_fasc	G	0	0	0	0	0	0	0	0	0	0	0
Hal_acut	G	0	0	2	3	0	10	1	0	0	0	0
Hal_coff	G	2	0	3	5	13	11	4	3	2	0	0
Hal_exig	G	0	0	0	0	3	0	0	1	0	0	0
Hal_hols	G	0	0	0	0	0	0	0	0	0	0	0
Hal_hybr	G	0	0	0	0	0	0	0	0	0	0	0
Hal_luci	G	0	0	0	0	0	0	0	0	0	0	0
Hal_subh	G	0	0	0	0	0	0	1	2	0	0	2
Hal_tene	G	0	0	1	2	7	0	5	0	0	0	0
Hal_vene	G	0	0	0	0	0	0	0	0	0	0	0
Has_spic	G	0	2	2	0	2	3	5	8	0	2	2
Hip_hung	G	0	0	0	2	0	0	0	0	0	0	0
Hip_lesm	G	0	0	0	0	0	0	0	0	0	0	0
Hya_scot	G	0	0	0	0	0	1	0	0	0	0	1
Kar_amoe	G	0	0	0	0	0	0	0	0	0	0	0
Kar_clev	G	0	0	1	4	3	4	2	2	0	0	0
Lic_comm	G	0	0	0	0	0	0	0	0	0	0	0
Lic_debi	G	0	0	0	0	0	2	17	1	4	15	21
Lic_gran	G	2	0	0	0	0	0	0	0	0	0	2
Lic_rhom	G	0	0	0	0	0	0	0	0	0	0	1
Lun_bise	G	38	16	39	25	24	32	13	12	2	4	19
Lut_muti	G	0	0	0	0	1	0	0	1	0	4	0
Mar_atom	G	10	33	32	61	49	72	54	42	25	19	38
Mar_schu	G	0	0	0	0	0	0	0	0	0	0	0
Mas_balt	G	0	0	0	0	0	0	0	0	0	0	0
Mas_brau	G	0	0	0	0	0	0	0	0	0	0	0
Mas_elli	G	0	0	0	0	0	0	2	0	0	0	0

Mas_exig	G	0	0	0	0	0	0	0	0	0	0	0
Mas_pumi	G	0	0	0	0	0	0	0	0	0	0	0
Mas_smit	G	0	0	6	2	2	21	18	5	2	2	25
May_atom	G	0	0	0	0	0	0	0	0	0	0	0
Mel_arct	G	4	1	0	0	0	0	0	0	0	0	0
Mel_line	G	0	8	14	45	7	12	29	2	4	3	16
Mel_moni	G	0	0	0	1	2	9	1	1	0	0	21
Mel_numm	G	0	0	0	0	0	0	0	0	0	0	0
Mel_spec	G	4	3	11	0	20	12	41	119	202	265	117
Nav_aren	G	0	0	0	0	0	0	0	0	0	0	0
Nav_brem	G	0	1	2	0	1	0	2	0	0	0	0
Nav_cinc	G	0	0	0	0	0	0	0	0	0	0	0
Nav_clem	G	0	0	0	0	0	0	0	1	0	0	0
Nav_cryp	G	0	0	0	0	0	2	0	0	0	0	0
Nav_digi	G	0	0	0	0	0	0	0	0	0	0	0
Nav_duer	G	0	0	2	0	9	11	5	5	2	1	2
Nav_flan	G	0	0	0	0	0	0	0	0	0	0	0
Nav_greg	G	6	6	5	7	3	7	5	3	10	2	1
Nav_infi	G	0	1	2	3	0	20	38	18	4	6	9
Nav_lanc	G	0	0	2	2	0	0	1	0	6	2	0
Nav_meni	G	0	0	0	0	0	0	0	0	0	0	0
Nav_nole	G	0	0	0	0	0	0	0	0	0	0	0
Nav_pere	G	0	0	0	0	0	0	0	0	0	0	0
Nav_perm	G	39	67	41	22	44	20	27	28	49	37	9
Nav_phyl	G	0	3	6	10	2	4	6	2	7	0	4
Nav_rato	G	0	0	0	0	4	0	0	4	1	1	0
Nav_rhyn	G	0	2	1	0	0	0	0	0	0	0	0
Nav_saco	G	0	0	0	1	0	2	0	1	0	0	0
Nav_sana	G	0	0	0	0	0	0	0	0	0	0	0
Nav_sjoe	G	10	1	2	0	1	0	0	0	1	0	0
Nav_supr	G	0	0	0	0	0	3	2	2	0	0	0
Nav_trip	G	0	0	0	0	0	0	1	0	0	0	0
Nav_vene	G	0	0	0	0	0	0	0	0	0	0	3
Ncy_pusi	G	0	0	0	0	0	0	0	0	0	1	0
Nit_amph	G	0	2	0	0	0	0	2	0	0	0	0
Nit_anla	G	1	0	0	0	2	0	0	0	2	2	0
Nit_aura	G	0	0	0	0	2	0	0	2	0	0	0
Nit_brev	G	0	0	0	0	0	0	0	0	0	0	0
Nit_comm	G	0	0	0	0	0	0	0	0	0	0	0
Nit_diss	G	1	0	0	0	2	3	0	0	0	0	2
Nit_dubi	G	0	0	0	0	0	0	0	0	0	0	0
Nit_eleg	G	0	0	0	0	0	0	0	0	0	0	0
Nit_fili	G	0	0	0	0	4	2	0	6	0	0	2
Nit_flex	G	0	0	2	0	0	0	0	0	0	0	0
Nit_frus	G	142	60	151	46	78	47	55	75	47	25	54
Nit_gand	G	4	0	0	0	0	0	0	0	0	0	0
Nit_heuf	G	0	0	0	0	0	0	0	0	0	0	0
Nit_inco	G	0	0	0	0	6	0	0	0	0	0	0
Nit_lieb	G	0	6	2	0	0	2	11	4	0	0	2
Nit_lisu	G	0	0	0	0	0	0	2	0	0	0	0
Nit_lore	G	0	0	0	0	0	0	0	0	0	0	0
Nit_micr	G	0	6	7	5	17	9	6	5	0	0	2
Nit_pale	G	0	0	3	0	5	1	2	2	0	0	4
Nit_pate	G	0	0	0	0	2	0	0	0	0	0	0
Nit_pcea	G	1	29	52	34	24	2	11	16	11	4	6
Nit_pell	G	0	0	5	8	12	0	4	8	11	29	1
Nit_perm	G	0	0	2	2	0	0	0	0	0	0	0
Nit_pusi	G	0	0	0	11	1	0	4	0	0	0	2
Nit_reve	G	0	0	0	0	0	0	2	4	0	0	0
Nit_rose	G	8	21	19	56	27	31	41	46	22	21	15
Nit_sdea	G	0	0	0	0	0	0	0	0	0	0	0
Nit_sigm	G	0	0	0	0	0	0	0	0	2	0	2
Nit_soci	G	0	0	0	0	0	0	1	0	0	0	0
Nit_subc	G	0	0	0	0	0	0	0	0	0	0	0
Nit_supr	G	0	0	4	7	1	2	3	0	0	0	0
Nit_ther	G	5	2	6	7	18	8	10	7	4	2	2
Nit_vald	G	0	2	3	2	2	5	4	2	0	2	4
Ope_muta	G	5	4	30	70	44	64	27	34	13	19	31
Pau_taen	G	4	2	5	0	1	0	2	3	6	3	0
Pin_eleg	G	0	0	0	0	0	0	0	0	0	0	0
Pin_kroc	G	0	0	0	0	0	0	0	0	0	0	0
Pin_micr	G	0	0	0	0	0	0	0	0	0	0	0
Ple_elon	G	0	0	0	0	0	0	0	0	0	0	0
Ple_sali	G	0	0	0	0	0	0	0	0	0	0	0
Plt_deli	G	1	0	3	2	2	0	3	0	1	1	1

Plt_disp	G	0	0	0	0	0	0	0	0	0	0	0
Plt_freq	G	0	0	2	0	0	0	0	0	0	0	0
Plt_hauc	G	2	6	11	3	17	4	13	4	6	0	7
Plt_lemm	G	0	2	5	8	2	6	8	0	1	0	0
Plt_sept	G	0	0	0	0	0	0	0	0	0	0	0
Pro_buln	G	4	2	3	8	0	4	0	0	0	0	0
Pse_west	G	0	0	0	0	0	0	0	0	0	0	0
Psf_tene	G	0	0	0	0	0	0	0	0	0	0	0
Pss_brev	G	0	2	1	3	3	1	2	2	2	0	0
Pss_elli	G	18	113	211	274	240	272	213	196	110	81	97
Pss_zeil	G	4	27	18	50	24	39	21	23	11	4	22
Pst_punc	G	0	0	0	0	0	0	0	0	0	0	0
Ptd_gemm	G	0	0	0	0	0	0	0	0	0	0	0
Rei_sinu	G	0	0	0	0	0	0	0	0	0	0	0
Rho_abbr	G	17	8	21	9	13	5	10	4	5	5	9
Rhp_gibb	G	0	0	1	2	1	6	2	0	0	0	0
Rhp_gipa	G	0	0	0	0	0	0	0	0	0	0	0
Sel_pupu	G	0	0	0	0	0	0	0	2	0	0	0
Ske_cost	G	2	8	6	9	0	0	0	0	0	0	0
Std_hant	G	0	0	0	0	0	0	0	0	0	0	0
Std_minu	G	0	1	0	0	0	0	0	0	0	0	0
Stn_simu	G	0	0	0	2	0	0	0	0	0	0	0
Sur_breb	G	7	6	5	2	0	1	1	3	0	1	0
Syn_acus	G	0	0	1	0	0	0	0	0	0	0	0
Tab_fasc	G	12	3	14	1	7	11	12	14	20	17	27
Tab_tabu	G	0	0	0	0	0	0	0	0	0	4	4
Tab_waer	G	0	0	0	0	0	0	0	0	0	0	0
Tal_fene	G	0	0	0	0	0	0	0	0	0	0	0
Tal_floc	G	0	0	0	0	0	0	0	0	0	0	0
Tha_balt	G	1	0	0	1	2	5	2	1	1	0	4
Tha_leva	G	2	3	0	2	0	0	0	2	2	2	0
Tha_pros	G	0	0	0	0	0	0	0	18	9	2	6
Tha_pseu	G	1	0	1	2	1	2	0	0	3	1	4
Tro_dann	G	0	0	0	0	0	0	0	0	0	0	0
Try_angu	G	0	0	0	2	0	0	0	0	0	0	0
Try_apic	G	0	0	2	6	0	3	2	2	0	0	1
Try_ardu	G	0	0	0	0	0	0	4	2	0	0	0
Try_hung	G	0	0	0	0	0	0	0	0	0	0	0
Try_levi	G	0	0	0	0	2	3	0	0	0	0	0
Acd_minu	H	1	4	0	12	8	2	4	2	0	0	5
Acd_pyre	H	0	0	0	0	0	0	0	0	0	0	0
Acd_suba	H	0	0	0	0	0	0	0	0	0	0	0
Ach_bica	H	0	0	0	0	0	0	0	0	0	0	0
Ach_brev	H	1	0	0	0	2	0	0	0	0	0	0
Ach_brin	H	0	0	0	0	1	1	1	0	0	0	2
Ach_long	H	0	0	0	0	0	0	0	0	0	0	0
Ach_misc	H	5	0	0	6	0	0	0	0	2	2	2
Ach_vist	H	0	0	0	0	0	0	0	0	0	0	0
Act_occr	H	0	0	0	0	0	0	0	0	0	0	0
Amp_comm	H	0	0	0	0	0	0	0	0	0	0	0
Amp_copu	H	0	0	1	0	0	0	0	0	0	0	0
Amp_fleb	H	6	2	1	23	7	0	1	7	6	8	3
Amp_inar	H	0	0	0	0	0	0	0	0	0	0	0
Amp_line	H	0	0	0	0	0	0	0	0	0	0	0
Amp_micr	H	0	0	0	0	0	0	0	0	0	0	0
Amp_oval	H	0	0	0	0	0	0	0	0	0	0	0
Amp_pedi	H	1	4	0	4	5	0	2	0	0	2	2
Amp_stau	H	0	0	0	0	0	0	0	0	0	0	0
Ane_tusc	H	0	0	0	0	0	0	0	0	0	0	0
Asa_bahu	H	0	0	0	0	0	0	0	0	0	0	0
Ast_form	H	0	0	0	0	0	0	0	0	0	0	0
Aul_spp	H	0	0	0	0	0	0	0	0	0	0	0
Bac_paxi	H	0	0	0	0	0	0	2	32	17	3	7
Ber_ruti	H	10	10	20	5	4	0	4	35	73	46	67
Bra_apon	H	0	0	0	0	0	0	0	0	0	0	0
Bre_lanc	H	0	0	0	0	0	0	0	0	10	29	10
Cal_baci	H	0	0	0	2	0	0	0	2	0	0	2
Cer_clos	H	2	0	0	8	6	0	10	3	12	4	0
Cha_spp.	H	7	1	1	6	0	0	1	0	0	0	1
Chp_marg	H	2	2	0	2	0	0	0	4	4	0	0
Coa_guil	H	0	0	0	0	0	0	0	0	0	0	0
Coc_neod	H	2	0	0	2	3	0	0	1	0	1	1
Coc_neot	H	0	0	0	3	6	0	2	0	0	1	1
Coc_pedi	H	2	0	5	12	1	73	14	7	5	4	10
Coc_pelt	H	0	0	0	0	0	0	0	0	0	0	0

Coc_plac	H	5	5	9	6	8	2	9	6	2	0	5
Coc_psth	H	0	0	0	2	0	0	0	0	0	0	0
Coc_scut	H	0	0	0	1	0	0	0	0	0	0	1
Con_weis	H	0	0	0	0	0	0	0	0	0	0	0
Cra_cusp	H	0	0	0	0	0	0	0	0	0	0	0
Cte_pulc	H	4	0	3	0	2	93	0	2	0	14	10
Cyb_amph	H	0	0	0	0	0	0	0	0	0	0	0
Cyc_atom	H	0	0	0	0	0	0	0	0	0	0	0
Cyc_choc	H	1	0	0	0	6	0	3	5	8	1	1
Cyc_mene	H	0	0	0	0	0	0	0	0	0	0	0
Cyc_radi	H	0	0	0	0	0	0	0	0	0	0	0
Cyc_stel	H	0	0	0	0	0	0	0	0	0	0	0
Cyc_stri	H	0	0	0	0	0	0	0	0	0	0	0
Cyl_grac	H	0	0	0	0	0	0	0	0	0	0	0
Cym_affi	H	0	0	0	0	0	0	0	0	0	0	0
Cym_cist	H	0	0	0	0	0	0	0	0	0	2	0
Cym_lanc	H	0	0	0	0	0	0	0	2	0	0	0
Den_cret	H	2	4	8	0	0	0	0	0	4	4	0
Den_sund	H	0	0	0	2	0	0	0	0	0	0	0
Dia_moni	H	202	292	430	114	201	121	115	137	50	157	17
Dia_tenu	H	4	5	0	1	0	0	2	0	6	8	4
Dia_vulg	H	6	21	2	0	0	0	2	2	0	9	1
Dip_domb	H	0	0	0	0	0	0	0	0	0	0	0
Dip_smit	H	0	0	0	0	0	0	0	0	0	0	0
Dip_smpu	H	0	0	0	0	0	0	0	0	0	0	0
Dip_smrh	H	0	2	0	0	0	0	0	0	0	0	0
Enc_caes	H	0	0	0	0	0	0	0	0	6	1	0
Enc_lacu	H	0	0	0	0	0	0	0	0	0	0	0
Enc_sile	H	0	0	0	0	0	0	0	0	0	0	0
Ent_cost	H	0	0	0	0	0	0	0	0	0	0	0
Ent_giga	H	0	0	0	0	0	0	0	0	0	0	0
Ent_palu	H	1	1	0	3	2	1	0	19	5	2	1
Ent_pseu	H	0	0	0	0	0	0	0	0	0	0	0
Ent_suri	H	0	0	0	0	0	0	0	0	0	0	1
Epi_adna	H	0	0	0	0	0	0	0	2	2	0	0
Epi_sore	H	0	0	0	8	1	12	0	3	0	1	0
Epi_tuwe	H	0	1	0	2	0	0	0	0	0	0	0
Fal_cryp	H	0	0	0	0	0	0	0	2	0	0	0
Fal_psli	H	0	0	0	0	0	0	0	0	0	0	0
Fal_pygm	H	0	0	0	0	0	0	0	0	0	0	0
Fra_amic	H	2	8	0	7	10	2	0	10	4	2	0
Fra_cava	H	0	0	0	0	0	0	0	0	0	0	0
Fra_cons	H	1	0	13	2	7	113	38	102	37	42	14
Fra_hydu	H	0	0	0	0	0	0	0	0	0	0	0
Fra_stri	H	16	8	0	4	8	4	6	4	6	9	1
Frp_cyli	H	0	0	0	0	0	0	0	0	0	0	0
Fru_creu	H	0	0	0	0	0	0	0	0	0	0	0
Gom_oliv	H	241	171	20	11	19	20	23	26	37	71	28
Gom_parv	H	0	0	0	0	0	0	0	0	0	0	0
Gon_exig	H	0	0	0	0	0	0	0	0	0	0	0
Gos_pseu	H	2	2	2	2	6	0	0	0	0	2	6
Gra_ocea	H	0	0	0	0	0	0	0	0	0	0	0
Gyr_exim	H	0	0	0	0	0	0	0	0	0	0	0
Gyr_fasc	H	0	0	0	0	0	0	0	0	0	0	0
Hal_acut	H	0	1	0	0	2	0	0	0	0	0	0
Hal_coff	H	0	2	0	2	7	1	0	11	18	23	2
Hal_exig	H	0	0	0	0	5	0	0	0	0	0	6
Hal_hols	H	0	0	0	0	0	0	0	0	0	0	0
Hal_hybr	H	0	0	0	0	0	0	0	0	0	0	0
Hal_luci	H	0	0	0	0	0	0	0	0	0	0	0
Hal_subh	H	0	0	0	0	2	0	0	0	0	0	0
Hal_tene	H	5	5	3	16	37	0	28	8	18	9	15
Hal_vene	H	0	0	0	0	0	0	0	0	0	0	0
Has_spic	H	0	0	0	2	3	3	2	14	0	6	0
Hip_hung	H	0	2	0	14	2	0	0	4	4	0	0
Hip_lesm	H	0	0	0	0	0	0	0	0	0	0	0
Hya_scot	H	0	0	0	2	0	2	0	0	0	0	0
Kar_amoe	H	0	0	0	0	0	0	0	0	0	0	0
Kar_clev	H	0	0	0	0	0	0	0	0	0	0	0
Lic_comm	H	0	0	0	0	0	0	0	0	0	0	0
Lic_debi	H	0	0	0	0	2	1	0	0	1	0	0
Lic_gran	H	0	0	0	0	0	0	0	0	0	0	0
Lic_rhom	H	0	0	0	0	0	3	0	2	2	3	3
Lun_bise	H	5	6	0	8	2	0	1	2	2	1	0
Lut_muti	H	0	0	0	0	0	0	0	0	0	0	0

Mar_atom	H	12	19	4	42	10	1	3	21	4	0	4
Mar_schu	H	0	0	0	0	0	0	0	0	0	0	0
Mas_balt	H	0	2	0	0	0	0	0	0	0	0	0
Mas_brau	H	0	0	0	0	0	0	0	0	0	0	0
Mas_elli	H	2	0	0	0	0	0	0	0	0	0	0
Mas_exig	H	0	0	0	0	0	0	0	0	0	0	0
Mas_pumi	H	0	0	0	0	2	0	0	2	0	0	2
Mas_smit	H	0	2	2	2	55	4	0	0	0	0	2
May_atom	H	0	0	0	0	0	0	0	0	0	0	0
Mel_arct	H	0	0	0	0	0	0	0	0	0	0	0
Mel_line	H	0	6	0	10	2	4	1	5	0	1	0
Mel_moni	H	0	0	0	0	0	2	0	0	0	0	2
Mel_numm	H	0	0	0	0	0	0	0	0	0	0	0
Mel_spec	H	0	0	0	0	0	0	0	77	94	33	7
Nav_aren	H	0	0	0	0	0	0	0	0	0	0	0
Nav_brem	H	0	2	0	4	0	1	0	0	0	0	0
Nav_cinc	H	0	0	0	0	0	0	0	0	0	0	0
Nav_clem	H	0	0	0	0	0	0	0	0	0	0	0
Nav_cryp	H	0	0	0	0	0	0	0	0	0	0	0
Nav_digi	H	0	0	0	0	0	0	0	0	0	0	0
Nav_duer	H	0	0	0	2	12	8	0	29	3	0	0
Nav_flan	H	0	0	0	0	0	0	0	0	0	0	0
Nav_greg	H	4	10	2	18	1	0	0	4	3	0	0
Nav_infi	H	0	0	0	0	0	0	0	0	0	0	0
Nav_lanc	H	0	0	1	1	0	0	0	0	0	0	0
Nav_meni	H	0	0	0	0	0	0	0	0	0	0	0
Nav_nole	H	0	0	0	0	0	0	0	0	0	0	0
Nav_pere	H	0	0	0	0	0	0	0	0	0	0	0
Nav_perm	H	20	39	16	50	54	18	48	51	76	46	41
Nav_phyl	H	2	1	0	0	0	0	0	2	0	0	0
Nav_rato	H	0	0	1	0	0	0	1	0	4	4	3
Nav_rhyn	H	0	0	0	0	0	0	0	0	0	0	0
Nav_saco	H	0	0	0	0	0	0	4	0	2	0	0
Nav_sana	H	0	0	0	0	0	0	0	0	0	0	0
Nav_sjoe	H	2	0	0	0	0	0	0	0	0	0	0
Nav_supr	H	0	0	0	0	0	0	0	0	0	0	0
Nav_trip	H	0	0	0	0	0	0	0	0	0	0	0
Nav_vene	H	1	3	4	2	0	0	0	0	2	1	3
Ncy_pusi	H	0	0	0	2	0	0	0	0	0	0	0
Nit_amph	H	0	0	0	0	0	0	0	0	0	0	0
Nit_anla	H	0	2	0	0	0	0	0	0	0	2	0
Nit_aura	H	0	0	0	0	0	0	0	0	0	0	0
Nit_brev	H	0	0	0	0	0	0	0	0	2	0	0
Nit_comm	H	0	0	0	0	0	0	0	0	0	0	0
Nit_diss	H	0	0	0	0	0	0	0	0	0	0	0
Nit_dubi	H	0	0	0	0	0	0	0	0	0	0	0
Nit_eleg	H	0	0	0	0	0	0	0	0	0	0	0
Nit_fili	H	0	2	0	0	0	0	0	0	0	0	0
Nit_flex	H	0	0	0	0	0	0	0	0	0	0	0
Nit_frus	H	235	178	290	91	142	26	368	36	149	79	387
Nit_gand	H	0	0	0	0	0	0	0	0	0	0	0
Nit_heuf	H	0	0	0	0	0	0	0	0	0	0	0
Nit_inco	H	0	0	0	2	0	0	0	0	0	0	0
Nit_lieb	H	0	0	0	4	2	0	0	8	0	2	4
Nit_lisu	H	0	0	0	0	0	0	0	0	0	0	0
Nit_lore	H	0	0	0	0	0	0	0	0	0	0	0
Nit_micr	H	0	0	0	2	8	0	4	5	12	8	6
Nit_pale	H	0	2	0	4	0	0	0	0	0	0	1
Nit_pate	H	0	0	0	0	0	0	0	0	0	2	0
Nit_pcea	H	44	43	12	99	115	12	45	26	41	65	12
Nit_pell	H	2	4	0	3	0	0	2	10	12	11	0
Nit_perm	H	0	0	0	0	0	0	0	0	0	0	0
Nit_pusi	H	0	2	0	2	0	0	2	0	6	2	0
Nit_reve	H	0	0	0	0	0	0	0	0	0	0	0
Nit_rose	H	5	12	0	16	27	4	30	41	36	48	16
Nit_sdea	H	0	0	0	0	0	0	0	0	0	0	0
Nit_sigm	H	0	0	0	0	0	0	0	0	0	0	0
Nit_soci	H	0	0	0	0	0	0	0	0	0	0	0
Nit_subc	H	0	0	0	0	0	0	0	0	0	0	0
Nit_supr	H	0	0	0	4	2	0	0	2	0	2	0
Nit_ther	H	5	3	0	0	0	0	3	7	16	44	0
Nit_vald	H	0	0	0	4	0	0	2	4	0	2	5
Ope_muta	H	5	7	13	19	16	8	18	27	2	9	25
Pau_taen	H	0	0	0	0	0	0	0	0	0	0	0
Pin_eleg	H	0	0	0	0	0	0	0	0	0	0	0

Pin_kroc	H	0	0	0	0	0	0	0	0	0	0	0
Pin_micr	H	0	0	0	0	0	0	0	0	0	0	0
Ple_elon	H	0	0	0	0	0	0	0	0	0	0	0
Ple_sali	H	0	0	0	0	0	0	0	0	0	0	0
Plt_deli	H	0	1	0	0	0	0	0	2	0	0	0
Plt_disp	H	0	0	0	0	0	0	0	0	0	0	0
Plt_freq	H	0	0	0	0	0	0	0	0	0	0	0
Plt_hauc	H	0	0	1	4	2	0	4	1	4	0	4
Plt_lemm	H	0	0	0	0	1	0	0	0	0	2	0
Plt_sept	H	0	0	0	0	0	0	0	0	0	0	0
Pro_buln	H	0	0	0	0	0	0	0	0	0	0	0
Pse_west	H	0	0	0	0	0	0	0	0	0	0	0
Psf_tene	H	0	0	0	0	0	0	0	0	0	0	0
Pss_brev	H	0	0	0	5	6	0	1	3	3	0	0
Pss_elli	H	38	70	35	177	85	19	42	84	34	39	35
Pss_zeil	H	13	7	1	43	15	4	6	12	0	0	13
Pst_punc	H	0	0	2	0	1	0	0	0	2	0	0
Ptd_gemm	H	0	0	0	0	0	0	0	0	0	0	0
Rei_sinu	H	0	0	0	0	0	0	0	0	0	0	0
Rho_abbr	H	53	19	88	71	38	321	98	49	116	70	130
Rhp_gibb	H	0	0	2	4	0	6	0	1	0	1	0
Rhp_gipa	H	0	0	0	0	0	0	0	0	0	0	0
Sel_pupu	H	0	0	0	0	0	0	0	0	0	0	0
Ske_cost	H	10	2	0	0	1	0	0	0	0	0	0
Std_hant	H	0	0	0	0	0	0	0	0	0	0	0
Std_minu	H	0	0	0	0	0	0	0	0	0	0	0
Stn_simu	H	0	0	0	0	0	0	0	0	0	0	0
Sur_breb	H	0	3	0	0	1	0	0	0	1	0	0
Syn_acus	H	0	0	0	0	0	0	0	0	0	0	0
Tab_fasc	H	7	0	8	18	21	76	40	24	23	57	56
Tab_tabu	H	0	0	0	0	3	29	8	2	5	2	11
Tab_waer	H	0	0	0	0	0	0	0	0	0	1	0
Tal_fene	H	0	0	0	0	0	0	0	0	0	0	0
Tal_floc	H	0	0	0	0	0	0	0	0	0	0	0
Tha_balt	H	3	0	0	1	0	0	0	1	0	0	0
Tha_leva	H	1	0	0	0	1	0	0	0	0	0	0
Tha_pros	H	0	0	0	0	0	0	0	2	7	0	2
Tha_pseu	H	0	0	0	0	2	2	0	2	0	0	0
Tro_dann	H	0	0	0	0	0	0	0	0	0	0	0
Try_angu	H	0	0	0	0	0	0	0	0	0	0	0
Try_apic	H	0	0	0	0	0	0	0	2	0	0	0
Try_ardu	H	0	0	0	0	0	0	0	0	0	0	0
Try_hung	H	0	0	0	0	0	0	0	0	0	0	0
Try_levi	H	0	0	0	0	0	1	0	2	0	0	0
Acd_minu	I	9	10	32	29	0	4	6	2	4	2	3
Acd_pyre	I	2	0	0	0	0	0	0	1	0	0	0
Acd_suba	I	0	0	0	0	0	0	0	0	0	1	0
Ach_bica	I	0	0	0	0	0	0	0	0	0	0	0
Ach_brev	I	0	0	0	0	0	0	0	0	0	0	0
Ach_brin	I	0	2	0	0	0	4	2	2	18	0	3
Ach_long	I	0	0	0	0	0	0	0	0	0	0	0
Ach_misc	I	0	4	4	1	2	0	0	6	14	10	3
Ach_vist	I	0	0	2	0	0	0	0	1	0	1	0
Act_occr	I	0	0	0	0	0	0	1	0	2	0	0
Amp_comm	I	0	0	0	0	0	0	0	0	0	0	0
Amp_copu	I	0	0	0	0	0	0	0	1	0	0	0
Amp_fleb	I	10	7	14	9	53	41	8	9	9	19	14
Amp_inar	I	0	0	0	0	0	0	0	0	0	0	0
Amp_line	I	0	2	0	3	2	2	0	0	0	0	0
Amp_micr	I	0	0	0	0	0	0	0	0	0	0	0
Amp_oval	I	0	0	0	0	0	0	0	0	0	0	0
Amp_pedi	I	6	6	1	3	4	9	1	4	6	2	6
Amp_stau	I	0	0	0	0	0	0	0	0	0	0	0
Ane_tusc	I	0	0	0	0	0	0	0	0	0	0	0
Asa_bahu	I	0	0	0	0	0	0	0	0	0	0	0
Ast_form	I	0	0	0	0	0	0	0	0	0	0	0
Aul_spp	I	0	0	0	0	0	0	0	0	0	0	2
Bac_paxi	I	3	0	0	4	20	10	9	42	53	43	23
Ber_ruti	I	93	12	4	15	6	54	13	39	13	10	3
Bra_apon	I	0	0	0	0	0	0	0	0	0	0	0
Bre_lanc	I	0	0	0	0	0	0	2	0	0	0	0
Cal_baci	I	0	0	2	0	0	0	0	0	2	0	0
Cer_clos	I	2	0	0	12	0	0	1	2	12	4	0
Cha_spp.	I	4	1	1	1	10	0	2	2	2	2	4
Chp_marg	I	0	1	0	2	0	0	0	0	0	0	0

Coa_guil	I	0	0	0	0	0	0	0	0	0	0	0
Coc_neod	I	4	1	4	0	0	2	2	6	7	6	2
Coc_neot	I	0	0	0	0	0	0	0	0	1	0	0
Coc_pedi	I	0	2	0	7	5	3	1	0	2	2	6
Coc_pelt	I	0	0	0	0	0	0	0	0	0	0	0
Coc_plac	I	11	15	3	27	6	2	5	3	10	3	14
Coc_psth	I	1	0	1	0	0	0	0	0	0	0	0
Coc_scut	I	0	0	0	0	0	0	0	0	0	0	2
Con_weis	I	0	0	0	1	0	0	0	0	0	0	0
Cra_cusp	I	0	0	0	0	2	1	0	0	0	0	0
Cte_pulc	I	4	1	0	2	1	0	0	6	1	2	0
Cyb_amph	I	0	0	0	0	0	0	0	0	0	0	0
Cyc_atom	I	0	0	0	0	0	0	0	0	2	0	0
Cyc_choc	I	2	2	0	0	1	0	2	8	12	2	1
Cyc_mene	I	0	0	0	0	0	0	0	0	0	0	0
Cyc_radi	I	0	0	0	0	0	0	0	0	0	0	0
Cyc_stel	I	0	0	0	0	0	0	0	0	0	0	0
Cyc_stri	I	0	0	0	0	0	0	0	0	0	0	0
Cyl_grac	I	0	0	0	0	0	0	0	0	0	0	0
Cym_affi	I	0	0	0	0	0	0	0	0	0	0	0
Cym_cist	I	0	0	0	0	0	0	0	0	0	2	0
Cym_lanc	I	0	0	0	0	0	0	0	0	0	4	0
Den_cret	I	0	0	1	0	0	4	0	0	0	0	0
Den_sund	I	0	6	4	0	6	0	0	2	3	0	0
Dia_moni	I	46	7	4	5	1	3	16	1	1	2	3
Dia_tenu	I	17	4	2	11	27	9	10	6	3	0	1
Dia_vulg	I	33	6	0	0	0	0	0	0	0	0	2
Dip_domb	I	0	0	0	0	0	0	0	0	0	0	0
Dip_smit	I	0	0	0	0	0	0	0	1	0	0	2
Dip_smpu	I	0	0	0	0	0	0	0	0	0	0	0
Dip_smrh	I	0	0	0	0	0	0	0	0	0	0	0
Enc_caes	I	0	0	0	0	0	0	0	0	0	0	0
Enc_lacu	I	0	0	0	0	0	0	0	0	0	0	0
Enc_sile	I	0	0	0	0	0	0	0	0	0	0	0
Ent_cost	I	0	0	0	0	0	0	0	0	0	0	0
Ent_giga	I	0	0	0	0	1	0	0	0	0	0	0
Ent_palu	I	9	7	1	20	14	3	5	3	1	12	0
Ent_pseu	I	0	0	0	0	0	0	0	0	0	0	0
Ent_suri	I	0	0	0	1	0	0	0	1	0	0	0
Epi_adna	I	0	2	0	0	0	0	0	2	0	0	2
Epi_sore	I	9	9	14	16	4	20	12	13	5	2	3
Epi_tuwe	I	0	0	0	0	0	0	0	4	0	0	0
Fal_cryp	I	0	0	0	0	0	1	0	0	0	0	0
Fal_psli	I	0	0	0	0	0	0	0	0	0	0	0
Fal_pygm	I	0	0	1	0	0	4	0	2	0	0	0
Fra_amic	I	35	34	43	18	17	23	57	23	4	8	18
Fra_cava	I	3	0	2	2	2	2	3	1	0	0	1
Fra_cons	I	0	0	0	0	0	0	0	0	0	0	0
Fra_hydu	I	0	0	0	0	0	0	0	0	0	0	0
Fra_stri	I	12	4	4	5	4	1	6	5	9	9	0
Frp_cyli	I	0	0	0	0	0	0	0	0	1	0	0
Fru_creu	I	0	0	0	1	0	0	0	0	0	0	3
Gom_oliv	I	2	14	0	46	2	1	0	2	0	0	0
Gom_parv	I	0	0	0	0	0	0	0	0	0	0	0
Gon_exig	I	0	0	0	0	0	0	0	0	0	0	0
Gos_pseu	I	0	2	0	2	2	0	0	0	0	0	4
Gra_ocea	I	0	0	0	0	0	0	0	0	0	0	0
Gyr_exim	I	0	0	0	0	0	2	0	0	0	0	0
Gyr_fasc	I	0	0	0	0	0	0	0	0	0	0	0
Hal_acut	I	0	0	0	0	0	5	1	0	0	9	2
Hal_coff	I	4	7	17	14	57	34	39	38	43	6	8
Hal_exig	I	2	2	0	0	0	0	0	0	0	2	1
Hal_hols	I	0	0	1	0	3	2	2	2	0	0	0
Hal_hybr	I	0	0	0	0	2	1	2	0	2	0	0
Hal_luci	I	0	0	0	0	0	0	0	0	0	0	0
Hal_subh	I	0	0	1	0	0	0	0	0	2	0	0
Hal_tene	I	0	3	1	0	3	10	1	3	4	1	2
Hal_vene	I	0	0	0	0	0	0	0	0	0	0	0
Has_spic	I	0	0	0	2	8	0	5	2	2	0	0
Hip_hung	I	0	0	0	0	0	0	0	0	0	0	0
Hip_lesm	I	0	2	4	3	0	0	1	0	2	0	2
Hya_scot	I	0	0	0	0	0	2	2	0	0	0	0
Kar_amoe	I	0	0	0	0	0	0	0	0	0	0	0
Kar_clev	I	0	0	0	0	0	0	0	0	0	1	0
Lic_comm	I	0	0	0	0	0	0	0	0	0	0	0

Lic_debi	I	0	0	0	0	2	0	1	2	2	2	0
Lic_gran	I	0	0	0	0	0	0	0	0	0	0	0
Lic_rhom	I	0	0	0	0	0	0	0	0	0	0	0
Lun_bise	I	6	6	0	0	0	13	7	0	2	10	4
Lut_muti	I	0	0	0	0	0	0	0	0	0	0	0
Mar_atom	I	44	23	51	32	21	35	25	32	19	23	15
Mar_schu	I	2	0	1	0	2	2	0	1	0	0	0
Mas_balt	I	0	0	0	0	0	0	0	0	0	0	0
Mas_brau	I	0	0	0	0	2	4	4	6	2	0	0
Mas_elli	I	0	1	2	0	5	4	9	3	3	0	2
Mas_exig	I	0	0	0	0	2	9	0	0	0	0	0
Mas_pumi	I	0	0	4	4	69	89	44	29	20	0	2
Mas_smit	I	8	1	13	3	48	50	38	40	23	0	2
May_atom	I	0	0	0	0	0	0	0	0	0	0	0
Mel_arct	I	0	0	0	0	0	0	0	0	0	0	0
Mel_line	I	0	0	0	0	0	0	0	0	1	0	4
Mel_moni	I	6	11	1	0	0	0	0	0	1	1	11
Mel_numm	I	2	1	0	2	0	0	0	0	0	0	0
Mel_spec	I	53	23	4	51	122	5	25	34	65	496	312
Nav_aren	I	0	0	0	0	0	0	0	0	0	0	0
Nav_brem	I	0	2	0	0	0	0	1	1	2	0	0
Nav_cinc	I	0	0	0	0	0	0	0	0	0	0	0
Nav_clem	I	0	0	0	0	0	0	0	0	0	0	0
Nav_cryp	I	0	4	0	0	0	0	0	1	1	0	0
Nav_digi	I	2	0	0	0	0	0	0	0	0	0	0
Nav_duer	I	2	1	1	2	4	0	9	13	31	8	5
Nav_flan	I	2	0	0	0	0	1	0	0	0	0	0
Nav_greg	I	9	26	4	14	9	8	5	1	0	2	5
Nav_infi	I	0	0	2	0	0	0	0	0	0	0	0
Nav_lanc	I	0	0	0	0	0	0	0	1	1	0	0
Nav_meni	I	0	0	0	0	0	0	0	0	0	0	0
Nav_nole	I	0	0	0	0	0	0	0	0	0	0	0
Nav_pere	I	0	0	3	0	0	2	1	0	0	0	0
Nav_perm	I	29	39	26	20	14	0	18	64	153	42	97
Nav_phyl	I	10	12	5	2	3	15	7	2	0	0	0
Nav_rato	I	0	0	2	2	0	0	0	0	2	0	0
Nav_rhyn	I	1	0	0	0	0	0	0	0	0	0	0
Nav_saco	I	0	0	0	0	0	0	0	0	0	0	0
Nav_sana	I	0	2	0	0	2	2	0	0	0	0	0
Nav_sjoe	I	0	0	0	0	0	0	0	0	0	0	0
Nav_supr	I	0	0	0	1	0	0	0	0	0	0	0
Nav_trip	I	0	0	0	0	0	0	0	0	0	0	0
Nav_vene	I	0	0	0	0	0	2	2	0	0	0	0
Ncy_pusi	I	2	0	6	2	4	0	2	3	4	0	2
Nit_amph	I	0	0	2	0	0	0	0	1	0	0	0
Nit_anla	I	4	3	2	1	0	0	0	0	0	0	0
Nit_aura	I	0	0	0	2	0	0	0	0	0	0	0
Nit_brev	I	0	0	0	2	3	5	3	2	0	0	1
Nit_comm	I	0	0	0	0	0	0	0	0	0	0	0
Nit_diss	I	2	0	0	0	0	0	0	0	0	0	0
Nit_dubi	I	2	0	0	0	0	0	0	0	0	0	0
Nit_eleg	I	0	0	2	0	0	1	3	2	0	0	1
Nit_fili	I	0	0	0	3	1	0	0	2	0	4	0
Nit_flex	I	0	0	0	0	0	0	0	0	0	0	0
Nit_frus	I	28	260	51	252	32	55	27	30	57	15	93
Nit_gand	I	0	0	0	0	0	0	0	0	0	0	0
Nit_heuf	I	0	0	0	0	0	0	0	0	0	0	0
Nit_inco	I	0	0	0	0	0	0	0	0	0	0	1
Nit_lieb	I	2	0	0	0	2	0	4	19	23	2	2
Nit_lisu	I	0	0	0	0	0	0	0	2	0	0	0
Nit_lore	I	0	0	0	0	0	0	0	0	0	0	0
Nit_micr	I	34	19	50	29	95	26	77	56	74	22	60
Nit_pale	I	2	0	1	2	6	0	0	1	0	2	0
Nit_pate	I	0	0	6	2	17	15	6	3	5	1	2
Nit_pcea	I	7	4	8	46	22	8	31	8	2	5	2
Nit_pell	I	2	0	0	0	0	0	0	0	2	19	2
Nit_perm	I	0	0	0	0	0	0	0	0	0	0	0
Nit_pusi	I	8	4	9	13	49	24	22	43	27	8	0
Nit_reve	I	0	0	0	0	0	0	0	0	0	0	0
Nit_rose	I	20	0	6	6	16	7	32	9	42	59	31
Nit_sdea	I	0	0	0	0	1	0	0	0	0	0	0
Nit_sigm	I	0	0	0	0	1	2	0	0	0	0	0
Nit_soci	I	0	0	0	0	0	0	0	0	0	0	0
Nit_subc	I	0	0	0	0	0	0	0	0	0	0	0
Nit_supr	I	1	2	4	4	2	6	3	0	7	3	0

Nit_ther	I	0	2	0	4	4	0	0	0	0	0	1
Nit_vald	I	0	10	17	1	4	10	3	4	4	2	4
Ope_muta	I	61	45	108	25	19	74	82	45	22	3	18
Pau_taen	I	0	0	0	0	0	0	0	0	0	0	0
Pin_eleg	I	0	0	0	0	0	0	0	0	0	0	0
Pin_kroc	I	0	0	0	2	0	0	0	0	0	0	0
Pin_micr	I	2	0	0	0	0	0	0	0	0	0	0
Ple_elon	I	0	0	0	0	0	2	1	0	0	2	0
Ple_sali	I	0	0	0	0	0	0	0	0	1	0	0
Plt_deli	I	4	2	0	0	0	2	1	0	1	0	0
Plt_disp	I	0	0	0	0	0	0	0	0	0	0	0
Plt_freq	I	0	0	0	0	0	0	0	0	0	0	0
Plt_hauc	I	0	4	2	1	0	2	0	8	4	1	1
Plt_lemm	I	0	0	1	0	0	0	0	1	0	2	0
Plt_sept	I	0	0	0	0	0	0	0	0	0	0	0
Pro_buln	I	2	0	0	0	0	0	6	9	0	0	4
Pse_west	I	0	0	0	0	0	0	0	0	0	0	0
Psf_tene	I	0	0	0	0	0	0	0	0	0	0	0
Pss_brev	I	6	2	6	0	0	7	2	8	2	0	2
Pss_elli	I	160	144	237	38	64	121	138	106	68	41	67
Pss_zeil	I	105	104	177	33	38	110	112	120	33	8	43
Pst_punc	I	0	3	0	1	1	0	0	2	0	0	0
Ptd_gemm	I	0	0	0	0	0	0	0	0	0	0	0
Rei_sinu	I	0	0	0	0	0	0	0	1	0	0	0
Rho_abbr	I	9	47	5	122	30	7	2	3	5	2	32
Rhp_gibb	I	1	0	5	1	0	6	4	3	0	0	0
Rhp_gipa	I	0	0	0	0	1	3	1	2	0	0	3
Sel_pupu	I	0	0	0	4	0	0	0	0	0	0	0
Ske_cost	I	7	0	0	0	0	0	0	0	0	0	1
Std_hant	I	0	0	0	0	0	0	0	0	0	0	0
Std_minu	I	0	0	0	0	0	0	0	0	0	0	0
Stn_simu	I	0	0	0	0	0	0	0	0	0	0	0
Sur_breb	I	1	4	0	0	1	0	0	0	0	4	2
Syn_acus	I	0	0	0	0	0	0	0	0	0	0	0
Tab_fasc	I	21	12	6	6	17	7	23	27	33	35	19
Tab_tabu	I	2	0	0	0	0	0	0	0	0	1	2
Tab_waer	I	0	0	0	0	0	0	0	0	0	0	0
Tal_fene	I	0	0	0	0	0	0	0	0	0	0	0
Tal_floc	I	0	0	0	0	0	0	0	0	0	0	0
Tha_balt	I	0	2	0	0	0	0	0	0	2	3	2
Tha_leva	I	6	0	0	2	0	0	0	0	0	0	0
Tha_pros	I	0	0	0	0	0	0	0	2	2	5	2
Tha_pseu	I	0	0	0	1	0	0	2	1	0	0	0
Tro_dann	I	0	0	0	0	0	0	0	0	0	0	0
Try_angu	I	0	0	0	0	0	0	0	0	0	0	0
Try_apic	I	0	0	2	0	0	0	0	2	0	0	1
Try_ardu	I	0	0	0	0	0	0	0	0	0	0	0
Try_hung	I	0	0	0	0	0	0	0	0	0	0	0
Try_levi	I	0	0	0	0	0	0	0	0	0	0	0
Acd_minu	J	4	9	0	3	8	8	10	26	8	10	5
Acd_pyre	J	0	0	0	0	0	0	0	0	2	0	0
Acd_suba	J	0	0	0	0	0	0	0	0	0	0	0
Ach_bica	J	0	0	0	0	0	0	0	0	0	0	0
Ach_brev	J	0	0	0	0	0	0	0	0	0	0	0
Ach_brin	J	0	0	0	2	2	1	15	0	0	0	1
Ach_long	J	0	0	0	0	0	0	0	0	0	0	0
Ach_misc	J	6	2	0	0	0	2	0	0	2	0	0
Ach_vist	J	0	0	0	0	0	0	0	0	0	0	0
Act_occr	J	0	0	0	0	0	0	0	0	0	0	0
Amp_comm	J	0	0	0	0	0	0	0	0	0	0	0
Amp_copu	J	0	0	0	0	0	0	0	0	0	0	2
Amp_fleb	J	12	6	4	61	7	8	2	5	1	2	1
Amp_inar	J	0	0	0	0	1	0	0	0	0	0	0
Amp_line	J	0	0	0	2	0	2	0	0	0	0	0
Amp_micr	J	0	0	0	0	0	0	0	0	0	0	0
Amp_oval	J	0	0	0	0	0	0	0	0	0	0	0
Amp_pedi	J	4	3	6	6	8	1	9	16	13	4	14
Amp_stau	J	0	0	0	0	0	0	0	0	0	0	0
Ane_tusc	J	0	0	0	0	0	0	0	1	0	0	0
Asa_bahu	J	0	0	0	0	0	0	0	0	1	0	0
Ast_form	J	0	0	0	0	0	0	0	0	0	0	0
Aul_spp	J	0	0	0	0	0	0	0	0	0	0	0
Bac_paxi	J	0	0	0	0	0	0	0	0	0	0	0
Ber_ruti	J	0	1	5	13	12	10	4	11	1	0	0
Bra_apon	J	2	0	0	2	20	14	24	31	34	44	8

Bre_lanc	J	0	0	0	0	0	0	0	0	0	0	0
Cal_baci	J	2	0	0	1	2	0	2	2	0	4	0
Cer_clos	J	0	0	0	15	4	0	0	0	2	2	2
Cha_spp.	J	2	0	0	3	0	0	0	0	1	0	0
Chp_marg	J	0	0	0	0	0	1	0	2	0	0	2
Coa_guil	J	0	0	0	2	0	0	0	0	0	0	0
Coc_neod	J	5	2	2	3	2	6	0	7	6	2	12
Coc_neot	J	0	0	0	0	2	0	2	0	0	6	6
Coc_pedi	J	1	1	16	5	2	17	16	6	8	30	19
Coc_pelt	J	0	0	2	0	0	0	0	0	0	0	0
Coc_plac	J	6	4	11	16	2	18	26	35	74	121	56
Coc_psth	J	0	3	0	0	0	0	4	0	0	0	2
Coc_scut	J	2	0	1	0	1	3	2	0	4	4	6
Con_weis	J	0	0	0	0	0	0	0	0	0	0	0
Cra_cusp	J	0	0	0	0	0	0	0	0	0	0	0
Cte_pulc	J	10	2	11	1	37	0	4	14	3	2	7
Cyb_amph	J	0	0	0	0	0	0	0	0	0	0	0
Cyc_atom	J	0	0	0	0	0	0	0	0	0	0	0
Cyc_choc	J	0	0	0	0	0	0	4	0	5	0	0
Cyc_mene	J	0	0	0	2	1	0	0	0	1	0	0
Cyc_radi	J	0	0	0	0	0	0	0	0	0	0	0
Cyc_stel	J	0	0	0	0	0	0	0	0	0	0	0
Cyc_stri	J	0	0	0	0	0	0	0	0	0	0	0
Cyl_grac	J	0	0	0	0	2	0	0	0	0	0	0
Cym_affi	J	0	0	0	0	0	0	0	0	0	0	0
Cym_cist	J	0	0	0	0	0	0	0	0	0	0	0
Cym_lanc	J	0	0	0	0	0	0	0	0	0	0	0
Den_cret	J	0	0	0	2	0	0	0	0	0	0	0
Den_sund	J	0	0	0	0	0	0	0	0	0	0	2
Dia_moni	J	45	26	6	14	26	3	12	69	13	3	6
Dia_tenu	J	1	5	0	36	114	21	8	13	1	0	0
Dia_vulg	J	0	0	0	0	0	0	0	0	0	0	0
Dip_domb	J	0	0	0	0	0	0	0	0	0	0	2
Dip_smit	J	0	0	0	0	0	0	0	0	0	0	3
Dip_smpu	J	0	0	0	1	0	0	0	0	0	0	0
Dip_smrh	J	0	0	0	0	0	0	0	0	0	0	0
Enc_caes	J	0	0	0	0	0	0	0	0	0	0	0
Enc_lacu	J	0	0	0	0	0	0	0	0	0	0	0
Enc_sile	J	0	0	0	0	0	0	0	0	0	0	0
Ent_cost	J	0	0	0	0	0	0	0	0	0	0	0
Ent_giga	J	0	0	0	0	0	0	0	0	0	0	0
Ent_palu	J	1	1	0	8	7	0	0	3	2	1	0
Ent_pseu	J	0	0	0	0	0	0	0	0	0	0	0
Ent_suri	J	0	1	0	0	0	0	0	0	0	0	0
Epi_adna	J	0	0	0	0	1	1	0	2	2	0	1
Epi_sore	J	4	3	1	5	4	8	11	5	2	8	6
Epi_tuwe	J	0	0	2	0	0	0	0	0	0	2	0
Fal_cryp	J	2	0	0	0	0	0	0	1	0	0	1
Fal_psli	J	0	0	0	0	0	0	0	0	0	0	0
Fal_pygm	J	0	0	0	0	0	0	0	0	0	0	0
Fra_amic	J	7	4	4	5	1	24	7	2	12	0	4
Fra_cava	J	0	0	3	0	0	1	2	0	0	0	3
Fra_cons	J	0	0	0	1	4	0	3	23	7	2	4
Fra_hydu	J	0	0	0	0	0	0	0	0	0	0	0
Fra_stri	J	63	19	17	37	89	11	53	158	60	28	37
Frp_cyli	J	0	0	0	0	0	0	0	0	0	0	0
Fru_creu	J	0	0	2	0	0	0	0	0	0	0	0
Gom_oliv	J	252	201	68	16	3	0	0	2	4	2	0
Gom_parv	J	0	0	0	0	0	0	1	0	0	0	0
Gon_exig	J	0	0	0	0	0	0	0	0	0	0	0
Gos_pseu	J	2	0	0	0	2	2	4	0	0	0	0
Gra_ocea	J	0	0	0	0	0	0	0	0	0	0	0
Gyr_exim	J	0	0	0	0	0	0	0	0	0	0	0
Gyr_fasc	J	0	0	0	0	0	0	0	0	0	0	0
Hal_acut	J	0	0	0	0	0	0	0	0	0	0	0
Hal_coff	J	0	1	2	78	53	26	18	15	7	2	13
Hal_exig	J	0	0	0	0	0	0	0	0	0	0	0
Hal_hols	J	0	0	0	0	0	0	0	0	1	1	0
Hal_hybr	J	0	0	0	0	0	0	0	0	0	0	0
Hal_luci	J	0	0	0	0	0	0	0	0	0	0	0
Hal_subh	J	0	0	0	0	0	0	0	0	0	0	7
Hal_tene	J	0	1	0	0	1	0	2	1	0	2	5
Hal_vene	J	0	0	0	0	0	0	0	0	0	0	1
Has_spic	J	0	0	0	2	3	0	1	0	1	0	0
Hip_hung	J	0	4	2	0	0	0	0	0	0	0	0

Hip_lesm	J	0	0	0	0	2	4	3	6	2	4	11
Hya_scot	J	0	0	0	0	0	0	0	0	0	0	0
Kar_amoe	J	0	0	0	0	0	0	0	0	0	0	0
Kar_clev	J	0	0	0	0	0	0	0	0	1	0	0
Lic_comm	J	0	0	0	0	0	0	0	0	3	0	3
Lic_debi	J	0	0	0	1	30	4	3	2	1	0	0
Lic_gran	J	0	0	0	0	0	0	0	0	0	0	2
Lic_rhom	J	0	0	0	0	0	0	0	0	0	0	0
Lun_bise	J	24	2	5	2	0	1	2	0	0	0	6
Lut_muti	J	0	0	0	0	0	0	0	0	0	0	0
Mar_atom	J	53	40	54	13	26	47	52	20	12	6	37
Mar_schu	J	0	0	2	0	0	0	0	0	0	0	0
Mas_balt	J	0	0	0	0	0	0	0	0	0	0	0
Mas_brau	J	0	0	0	0	0	0	0	0	0	0	2
Mas_elli	J	0	0	0	0	2	0	0	0	0	0	0
Mas_exig	J	0	0	0	0	0	0	0	0	0	0	0
Mas_pumi	J	1	0	0	2	2	13	5	3	0	0	7
Mas_smit	J	0	3	1	5	8	103	7	7	11	2	7
May_atom	J	0	0	0	0	0	0	0	0	0	0	0
Mel_arct	J	0	0	0	0	0	0	0	0	0	0	0
Mel_line	J	0	0	0	0	0	1	1	2	2	0	0
Mel_moni	J	0	0	0	0	0	0	0	0	0	0	0
Mel_numm	J	0	0	0	0	0	0	0	0	0	0	0
Mel_spec	J	0	0	0	0	0	0	0	0	0	0	2
Nav_aren	J	0	0	0	0	0	0	0	0	0	0	0
Nav_brem	J	0	0	0	0	0	0	0	0	0	0	0
Nav_cinc	J	0	0	0	0	0	0	0	0	0	0	0
Nav_clem	J	0	0	0	0	0	0	0	0	0	0	0
Nav_cryp	J	0	0	2	8	0	0	2	0	0	0	0
Nav_digi	J	0	0	0	0	0	0	0	0	0	0	0
Nav_duer	J	0	0	0	0	3	0	0	0	0	2	0
Nav_flan	J	0	0	0	0	0	0	0	0	0	0	0
Nav_greg	J	2	6	41	12	2	2	8	7	4	8	45
Nav_infi	J	0	0	0	0	0	0	0	0	0	0	0
Nav_lanc	J	0	0	4	0	0	0	0	0	0	0	0
Nav_meni	J	0	0	0	0	0	0	0	0	0	0	1
Nav_nole	J	0	0	0	0	1	0	0	0	0	0	0
Nav_pere	J	0	0	0	1	0	0	0	0	0	0	0
Nav_perm	J	14	22	49	43	52	18	33	62	84	185	25
Nav_phyl	J	4	5	21	8	4	4	22	13	2	5	11
Nav_rato	J	0	0	0	0	0	0	0	0	0	0	0
Nav_rhyn	J	0	0	0	0	4	0	3	0	0	0	4
Nav_saco	J	0	0	0	0	0	2	0	0	2	2	0
Nav_sana	J	0	0	0	0	0	0	0	0	4	0	0
Nav_sjoe	J	0	0	0	0	2	0	0	0	0	0	0
Nav_supr	J	0	0	0	0	0	0	0	0	0	0	0
Nav_trip	J	0	1	0	0	1	0	0	0	0	2	0
Nav_vene	J	0	0	2	0	0	1	0	2	0	2	0
Ncy_pusi	J	10	0	1	2	2	12	5	0	2	4	0
Nit_amph	J	0	1	0	0	0	0	0	0	0	0	0
Nit_anla	J	0	0	0	0	0	0	0	0	0	0	0
Nit_aura	J	0	0	0	0	0	0	0	0	0	0	0
Nit_brev	J	0	0	0	0	6	0	0	0	0	0	0
Nit_comm	J	0	0	2	0	0	0	0	0	0	0	0
Nit_diss	J	0	0	0	0	0	0	0	0	0	0	0
Nit_dubi	J	0	0	0	0	0	0	0	0	0	0	0
Nit_eleg	J	0	0	0	0	0	3	4	6	0	0	0
Nit_fili	J	0	1	0	0	0	0	0	0	2	0	0
Nit_flex	J	0	0	0	0	0	0	0	0	0	0	0
Nit_frus	J	51	92	74	49	57	62	58	69	76	101	107
Nit_gand	J	1	0	0	0	0	0	0	0	0	0	0
Nit_heuf	J	0	0	0	0	0	0	0	0	0	0	0
Nit_inco	J	0	1	0	0	2	2	0	0	2	0	0
Nit_lieb	J	4	0	1	6	7	6	4	8	7	4	2
Nit_lisu	J	0	2	0	1	0	0	0	0	0	0	0
Nit_lore	J	0	0	0	0	0	0	0	0	0	0	0
Nit_micr	J	40	64	50	61	83	64	38	55	35	9	14
Nit_pale	J	0	2	0	3	2	0	0	0	2	0	0
Nit_pate	J	0	1	0	5	0	0	0	0	0	0	0
Nit_pcea	J	13	5	0	58	41	4	4	0	6	0	0
Nit_pell	J	0	0	0	0	0	0	1	0	0	0	0
Nit_perm	J	0	0	0	0	0	0	0	0	0	0	0
Nit_pusi	J	1	0	14	16	18	9	2	6	0	0	0
Nit_reve	J	0	0	0	0	0	0	0	0	0	0	0
Nit_rose	J	39	16	11	36	10	7	9	20	8	4	3

Nit_sdea	J	0	0	0	0	0	0	0	0	0	0	0
Nit_sigm	J	0	0	2	4	0	0	1	2	0	0	1
Nit_soci	J	0	0	0	0	0	0	0	0	0	0	0
Nit_subc	J	0	0	0	0	0	0	0	0	0	0	0
Nit_supr	J	0	0	0	0	2	0	0	2	0	0	0
Nit_ther	J	2	3	1	4	0	0	0	1	0	0	0
Nit_vald	J	1	4	1	1	0	0	4	4	8	4	5
Ope_muta	J	32	25	41	16	11	52	38	21	47	10	65
Pau_taen	J	0	0	1	2	0	0	0	0	0	0	0
Pin_eleg	J	0	0	0	0	0	0	0	0	0	0	0
Pin_kroc	J	0	0	0	0	0	0	2	0	0	0	0
Pin_micr	J	0	0	0	0	0	0	0	0	0	0	0
Ple_elon	J	0	0	0	0	0	0	2	0	0	0	3
Ple_sali	J	0	0	0	0	0	0	0	0	0	0	0
Plt_deli	J	0	1	3	0	1	0	2	0	0	0	3
Plt_disp	J	0	0	0	0	0	0	0	0	0	0	0
Plt_freq	J	0	0	0	0	0	0	0	1	0	0	1
Plt_hauc	J	1	0	0	4	0	1	4	2	1	0	3
Plt_lemm	J	0	0	1	0	0	2	5	0	1	0	0
Plt_sept	J	0	0	1	0	0	0	0	0	0	0	0
Pro_buln	J	0	0	0	0	0	0	0	0	0	0	0
Pse_west	J	0	0	0	0	0	0	0	0	0	0	0
Psf_tene	J	0	0	0	0	0	0	0	0	0	0	0
Pss_brev	J	2	1	0	0	0	4	2	0	1	1	0
Pss_elli	J	162	290	126	58	47	162	156	51	96	21	151
Pss_zeil	J	67	70	104	36	62	196	213	77	56	58	167
Pst_punc	J	0	0	0	0	0	0	0	0	0	0	2
Ptd_gemm	J	0	0	0	0	0	0	0	0	0	0	1
Rei_sinu	J	0	0	0	0	0	0	0	0	0	0	0
Rho_abbr	J	29	36	198	190	32	11	29	72	206	271	59
Rhp_gibb	J	0	2	0	0	6	1	0	0	0	0	0
Rhp_gipa	J	0	0	0	0	0	0	0	0	0	0	0
Sel_pupu	J	0	0	0	0	0	0	0	0	0	0	0
Ske_cost	J	0	0	0	2	0	0	0	0	0	0	0
Std_hant	J	0	0	2	0	0	0	0	0	0	0	0
Std_minu	J	0	0	0	0	0	0	0	0	0	0	0
Stn_simu	J	0	0	0	0	0	0	0	0	0	0	0
Sur_breb	J	1	2	1	0	0	0	0	0	0	0	3
Syn_acus	J	2	0	0	0	0	0	0	0	0	0	0
Tab_fasc	J	11	3	13	8	48	14	27	25	16	10	5
Tab_tabu	J	0	0	4	1	2	0	3	2	8	2	3
Tab_waer	J	0	0	0	0	0	0	0	0	0	0	0
Tal_fene	J	0	0	0	0	0	0	0	0	0	0	0
Tal_floc	J	0	0	0	0	0	0	0	2	0	0	0
Tha_balt	J	0	0	0	0	1	0	0	0	2	1	1
Tha_leva	J	0	0	0	0	0	0	0	0	0	0	0
Tha_pros	J	0	0	0	0	0	0	0	0	10	0	0
Tha_pseu	J	0	0	0	0	0	0	0	0	2	0	0
Tro_dann	J	0	0	0	0	0	0	0	0	0	0	0
Try_angu	J	0	0	0	0	0	0	0	0	0	0	0
Try_apic	J	0	0	0	0	0	0	0	0	0	0	0
Try_ardu	J	0	0	0	0	0	0	0	0	0	0	0
Try_hung	J	0	0	0	0	0	0	0	0	0	0	0
Try_levi	J	0	0	2	0	0	0	0	0	0	0	0
Acd_minu	K	1	0	22	0	6	4	4	2	0	0	0
Acd_pyre	K	0	0	0	0	0	0	0	0	0	0	0
Acd_suba	K	0	0	0	0	0	0	0	0	0	0	0
Ach_bica	K	0	0	0	0	0	0	0	0	0	0	0
Ach_brev	K	0	1	0	0	2	0	1	0	0	0	0
Ach_brin	K	2	0	0	0	1	2	0	14	3	1	34
Ach_long	K	0	0	0	0	0	0	0	0	0	0	0
Ach_misc	K	3	0	0	0	2	0	0	2	0	2	0
Ach_vist	K	0	0	0	0	0	0	0	0	0	0	0
Act_occr	K	0	0	0	0	0	0	0	0	0	0	0
Amp_comm	K	0	0	0	0	0	0	0	0	0	0	0
Amp_copu	K	0	0	0	0	0	0	1	0	0	0	0
Amp_fleb	K	9	14	31	4	3	5	3	2	0	10	2
Amp_inar	K	0	0	0	0	0	0	0	0	0	0	0
Amp_line	K	0	0	0	0	0	0	0	0	0	0	0
Amp_micr	K	0	0	0	0	0	0	0	0	0	0	0
Amp_oval	K	0	0	0	0	0	0	0	0	1	0	0
Amp_pedi	K	0	0	2	0	3	3	2	3	0	5	5
Amp_stau	K	0	0	0	0	0	0	0	0	0	0	0
Ane_tusc	K	0	0	0	0	0	0	0	0	0	0	0
Asa_bahu	K	0	0	0	0	0	0	0	0	0	0	0

Ast_form	K	0	0	0	0	0	0	0	0	0	0	0
Aul_spp	K	2	0	0	0	0	0	0	0	0	0	0
Bac_paxi	K	10	1	0	0	2	19	10	7	14	253	31
Ber_ruti	K	38	59	5	1	1	92	12	42	23	9	62
Bra_apon	K	0	0	0	0	0	0	0	0	0	0	0
Bre_lanc	K	0	2	0	0	0	0	0	0	10	0	1
Cal_baci	K	0	0	2	0	0	1	0	0	0	0	0
Cer_clos	K	6	2	2	14	0	12	11	2	2	0	0
Cha_spp.	K	45	32	4	4	3	0	0	0	1	4	0
Chp_marg	K	0	2	1	2	0	0	0	0	0	4	2
Coa_guil	K	0	0	0	0	0	0	0	0	0	0	0
Coc_neod	K	2	1	0	0	0	1	2	7	1	1	2
Coc_neot	K	0	0	0	0	0	0	0	0	0	0	2
Coc_pedi	K	3	3	8	1	16	9	1	18	6	9	42
Coc_pelt	K	0	0	0	1	0	0	0	0	0	1	0
Coc_plac	K	0	0	2	2	3	0	2	3	4	4	12
Coc_psth	K	0	0	0	0	0	0	0	0	0	0	0
Coc_scut	K	0	0	0	0	0	0	0	0	0	0	0
Con_weis	K	0	0	0	0	0	0	0	0	0	0	0
Cra_cusp	K	0	0	0	0	0	0	0	0	0	0	0
Cte_pulc	K	1	4	2	0	10	12	2	2	3	6	0
Cyb_amph	K	0	0	0	0	0	0	0	0	0	0	0
Cyc_atom	K	0	0	0	1	0	0	0	0	0	0	0
Cyc_choc	K	1	1	0	0	0	0	0	6	3	16	3
Cyc_mene	K	0	0	0	0	1	0	2	0	0	0	0
Cyc_radi	K	0	0	0	0	0	0	0	0	0	0	0
Cyc_stel	K	0	0	0	0	0	0	0	0	0	0	0
Cyc_stri	K	0	0	0	0	0	0	0	0	0	0	0
Cyl_grac	K	0	0	0	0	0	0	0	0	0	0	0
Cym_affi	K	0	0	0	0	0	0	0	0	0	0	0
Cym_cist	K	0	0	0	0	0	0	0	0	0	0	0
Cym_lanc	K	0	0	0	0	0	0	0	0	0	0	0
Den_cret	K	0	0	0	0	0	0	0	0	1	2	0
Den_sund	K	0	0	0	0	0	0	0	0	0	0	0
Dia_moni	K	193	206	182	54	69	15	52	2	1	0	2
Dia_tenu	K	36	31	11	2	1	3	2	0	0	0	0
Dia_vulg	K	203	34	2	1	0	0	0	2	0	0	0
Dip_domb	K	0	0	0	0	0	0	0	0	0	0	0
Dip_smit	K	0	0	0	0	0	0	0	0	0	0	0
Dip_smpu	K	0	0	0	0	0	0	0	0	0	0	0
Dip_smrh	K	0	0	0	0	0	0	0	0	0	0	0
Enc_caes	K	0	0	0	0	0	0	2	1	0	0	0
Enc_lacu	K	0	0	0	0	0	0	0	0	0	0	0
Enc_sile	K	0	0	0	0	0	0	0	0	0	0	0
Ent_cost	K	0	0	0	0	0	0	0	0	0	0	0
Ent_giga	K	0	0	0	0	0	0	0	0	0	0	0
Ent_palu	K	7	0	2	3	0	0	6	0	0	2	1
Ent_pseu	K	0	0	0	0	0	0	0	0	0	0	0
Ent_suri	K	1	1	0	0	0	0	0	0	0	0	0
Epi_adna	K	0	0	0	0	4	0	0	0	0	1	0
Epi_sore	K	2	2	4	4	55	69	16	21	2	7	11
Epi_tuwe	K	2	1	0	0	3	0	5	2	0	1	2
Fal_cryp	K	0	0	0	0	0	0	0	0	0	0	0
Fal_psli	K	0	0	0	0	0	0	0	0	0	0	0
Fal_pygm	K	0	0	0	0	0	0	0	0	0	0	0
Fra_amic	K	0	4	0	0	2	0	0	7	1	12	4
Fra_cava	K	0	0	0	0	0	0	0	0	0	0	0
Fra_cons	K	0	3	0	0	66	17	0	0	2	0	0
Fra_hydu	K	0	0	0	0	0	0	0	0	0	0	0
Fra_stri	K	7	10	9	5	9	10	1	0	1	0	0
Frp_cyli	K	0	0	0	0	0	0	0	0	0	0	0
Fru_creu	K	0	0	0	0	0	0	0	0	0	0	0
Gom_oliv	K	2	11	11	3	0	10	2	0	0	0	0
Gom_parv	K	0	0	0	0	0	0	0	0	0	0	0
Gon_exig	K	0	0	0	0	0	0	0	0	0	0	0
Gos_pseu	K	0	0	0	0	0	2	0	0	0	0	0
Gra_ocea	K	0	0	0	0	0	0	0	0	0	0	0
Gyr_exim	K	0	0	0	0	0	0	0	0	0	0	0
Gyr_fasc	K	0	0	0	0	0	0	0	2	0	0	0
Hal_acut	K	0	0	2	2	1	2	0	4	2	5	0
Hal_coff	K	0	45	12	52	3	24	5	10	7	3	12
Hal_exig	K	0	0	15	2	0	0	0	1	0	0	0
Hal_hols	K	0	0	0	0	0	0	0	0	0	0	0
Hal_hybr	K	0	0	0	0	0	0	0	0	0	0	0
Hal_luci	K	0	1	0	0	0	0	0	0	0	0	0

Hal_subh	K	0	0	1	1	0	2	0	0	0	1	1
Hal_tene	K	1	1	12	3	6	4	1	2	6	0	0
Hal_vene	K	0	0	0	0	0	0	0	0	0	0	0
Has_spic	K	0	0	8	4	2	22	1	2	0	0	0
Hip_hung	K	0	0	0	2	0	0	0	0	0	0	0
Hip_lesm	K	0	2	0	0	0	1	0	0	2	0	0
Hya_scot	K	0	0	0	0	0	0	0	0	0	0	0
Kar_amoe	K	0	0	0	0	0	0	0	0	0	0	0
Kar_clev	K	0	2	0	0	0	0	0	0	0	0	2
Lic_comm	K	0	0	0	0	0	0	0	0	2	0	0
Lic_debi	K	0	0	7	1	45	12	0	0	0	2	0
Lic_gran	K	0	0	0	0	0	0	0	0	0	0	0
Lic_rhom	K	0	0	0	0	0	0	0	0	0	0	0
Lun_bise	K	4	0	0	0	1	0	0	0	0	2	1
Lut_muti	K	0	0	0	0	0	0	0	0	0	0	0
Mar_atom	K	15	2	6	8	14	5	4	6	1	13	0
Mar_schu	K	0	0	0	0	0	0	0	0	0	0	0
Mas_balt	K	0	0	0	0	0	0	0	0	0	0	0
Mas_brau	K	0	0	0	0	0	0	0	0	0	0	0
Mas_elli	K	0	0	0	2	2	5	2	0	0	0	0
Mas_exig	K	0	0	0	0	0	0	0	0	0	0	0
Mas_pumi	K	0	0	0	4	7	28	5	18	0	3	4
Mas_smit	K	0	0	8	3	80	82	29	27	0	2	3
May_atom	K	0	0	0	0	0	0	0	0	0	0	0
Mel_arct	K	0	0	0	0	0	0	0	0	0	0	0
Mel_line	K	7	11	0	0	1	0	0	0	2	16	4
Mel_moni	K	4	8	2	0	0	0	0	2	4	20	82
Mel_numm	K	10	4	1	0	1	0	0	0	0	0	0
Mel_spec	K	100	35	22	199	156	85	266	127	160	256	27
Nav_aren	K	0	0	0	0	0	0	0	0	0	0	0
Nav_brem	K	0	0	0	0	0	0	0	0	0	0	0
Nav_cinc	K	0	0	0	0	0	0	0	0	0	0	0
Nav_clem	K	0	0	0	0	0	0	0	0	0	0	0
Nav_cryp	K	0	0	0	0	0	0	0	0	0	0	0
Nav_digi	K	0	0	0	0	0	0	0	0	0	0	0
Nav_duer	K	0	6	19	31	4	1	3	6	0	3	0
Nav_flan	K	0	0	0	0	0	0	0	0	0	0	0
Nav_greg	K	8	16	2	0	0	0	2	0	2	2	0
Nav_infi	K	4	2	0	0	0	0	0	2	2	2	0
Nav_lanc	K	0	0	0	2	0	0	0	0	0	0	0
Nav_meni	K	0	0	0	0	0	0	0	0	0	0	0
Nav_nole	K	0	0	0	0	0	0	0	0	0	0	0
Nav_pere	K	0	0	0	0	0	2	0	0	0	0	0
Nav_perm	K	80	147	78	30	58	122	208	344	382	48	237
Nav_phyl	K	1	1	0	0	0	0	0	1	2	0	0
Nav_rato	K	0	6	0	1	0	0	0	0	0	1	0
Nav_rhyn	K	0	0	0	0	0	0	0	0	0	0	0
Nav_saco	K	2	0	0	1	0	0	0	0	0	0	0
Nav_sana	K	0	0	0	0	0	0	0	0	0	0	0
Nav_sjoe	K	0	0	0	0	0	0	0	0	0	0	0
Nav_supr	K	0	0	0	0	0	0	0	0	0	0	0
Nav_trip	K	0	0	0	0	0	0	0	0	0	0	0
Nav_vene	K	0	0	0	0	0	0	0	0	0	0	0
Ncy_pusi	K	0	0	0	2	0	2	7	4	0	0	0
Nit_amph	K	0	0	0	0	0	0	1	0	0	0	0
Nit_anla	K	0	0	0	0	0	0	0	0	0	0	0
Nit_aura	K	0	0	0	0	0	0	0	0	0	0	0
Nit_brev	K	0	0	0	0	0	0	0	0	0	0	0
Nit_comm	K	0	0	0	0	0	0	0	0	0	0	0
Nit_diss	K	0	0	0	0	0	0	0	0	0	0	0
Nit_dubi	K	0	0	0	0	0	0	0	0	0	0	0
Nit_eleg	K	0	0	0	0	0	0	0	0	0	0	0
Nit_fili	K	2	1	2	0	0	0	0	0	0	0	6
Nit_flex	K	0	0	0	0	0	0	0	0	0	0	0
Nit_frus	K	25	56	91	29	33	47	53	73	96	40	129
Nit_gand	K	2	0	0	0	0	0	0	0	0	0	0
Nit_heuf	K	0	0	0	0	0	0	0	0	0	0	0
Nit_inco	K	0	0	0	0	0	0	0	0	0	0	0
Nit_lieb	K	0	17	14	8	0	0	4	7	16	0	5
Nit_lisu	K	0	0	0	0	0	0	0	0	0	0	0
Nit_lore	K	0	0	0	0	0	0	0	0	0	2	0
Nit_micr	K	8	14	71	42	10	18	32	33	27	13	22
Nit_pale	K	0	0	0	0	0	2	3	0	0	0	0
Nit_pate	K	0	0	0	9	4	0	8	0	0	0	0
Nit_pcea	K	14	51	147	278	54	51	25	5	2	0	2

Nit_pell	K	8	2	2	0	0	0	0	0	0	4	0
Nit_perm	K	0	0	2	0	0	0	0	0	0	0	0
Nit_pusi	K	0	6	1	2	0	0	10	4	0	0	6
Nit_reve	K	0	0	0	0	0	0	0	0	0	0	0
Nit_rose	K	16	28	70	104	61	39	51	10	27	24	20
Nit_sdea	K	0	0	0	0	0	0	0	0	0	0	0
Nit_sigm	K	0	0	0	1	0	0	0	0	0	0	0
Nit_soci	K	0	0	0	0	0	0	0	0	0	0	0
Nit_subc	K	0	0	0	0	0	0	0	0	0	0	0
Nit_supr	K	0	0	0	2	0	0	0	2	0	4	6
Nit_ther	K	0	1	2	1	1	8	0	0	0	0	0
Nit_vald	K	0	0	0	3	0	0	0	0	2	2	0
Ope_muta	K	7	5	9	3	12	1	4	12	12	25	5
Pau_taen	K	0	0	0	0	0	0	0	0	0	0	0
Pin_eleg	K	0	0	0	0	0	0	0	0	0	0	0
Pin_kroc	K	0	0	0	0	0	0	0	0	0	0	0
Pin_micr	K	0	0	0	0	0	0	0	0	0	0	0
Ple_elon	K	0	0	0	0	0	0	0	0	0	1	0
Ple_sali	K	0	0	0	0	0	0	0	0	0	0	0
Plt_deli	K	0	2	0	0	0	2	0	1	1	0	1
Plt_disp	K	0	0	0	0	0	0	0	0	0	0	0
Plt_freq	K	0	0	0	0	0	0	0	0	0	0	0
Plt_hauc	K	0	6	1	0	0	2	2	1	5	2	0
Plt_lemm	K	0	0	2	1	0	0	2	2	0	0	0
Plt_sept	K	0	0	0	0	0	0	0	0	0	0	0
Pro_buln	K	0	0	0	0	0	0	9	6	10	0	0
Pse_west	K	0	0	0	0	0	0	0	0	0	0	0
Psf_tene	K	0	0	0	0	0	0	0	0	0	0	0
Pss_brev	K	0	0	0	0	0	0	2	2	0	0	0
Pss_elli	K	27	33	22	19	38	16	13	42	34	53	29
Pss_zeil	K	7	4	0	17	13	3	12	17	3	70	17
Pst_punc	K	0	0	0	0	1	0	0	0	0	0	0
Ptd_gemm	K	0	0	0	0	0	0	0	0	0	0	0
Rei_sinu	K	0	0	0	0	0	0	0	0	0	0	0
Rho_abbr	K	2	4	23	14	64	33	47	34	53	1	69
Rhp_gibb	K	0	0	0	0	14	9	0	1	0	1	1
Rhp_gipa	K	0	0	0	0	0	0	0	0	0	0	0
Sel_pupu	K	0	0	0	0	0	0	0	0	0	0	0
Ske_cost	K	6	4	0	0	0	0	0	0	0	0	1
Std_hant	K	0	0	0	0	0	0	0	0	0	0	0
Std_minu	K	0	0	0	0	0	0	0	0	0	0	0
Stn_simu	K	0	0	0	0	0	0	0	0	0	0	0
Sur_breb	K	4	2	1	0	0	0	0	0	0	3	0
Syn_acus	K	0	0	0	0	0	0	0	0	0	0	0
Tab_fasc	K	52	50	41	15	35	79	48	38	29	17	47
Tab_tabu	K	4	0	0	0	15	5	2	4	28	5	42
Tab_waer	K	0	0	0	0	0	0	0	0	0	0	0
Tal_fene	K	0	0	0	0	0	0	0	0	0	0	0
Tal_floc	K	0	0	0	0	0	0	0	0	0	0	0
Tha_balt	K	0	0	0	0	0	0	0	0	1	0	1
Tha_leva	K	4	0	0	0	0	0	0	0	0	0	0
Tha_pros	K	0	0	0	0	0	0	0	3	1	0	0
Tha_pseu	K	0	0	1	0	0	0	0	0	0	2	0
Tro_dann	K	0	0	0	0	2	0	0	0	0	0	0
Try_angu	K	0	0	0	0	0	0	0	0	0	0	0
Try_apic	K	0	0	0	0	0	0	0	0	0	2	0
Try_ardu	K	0	0	1	0	0	0	0	0	0	0	0
Try_hung	K	0	0	0	0	0	0	0	0	0	0	0
Try_levi	K	0	0	0	0	0	0	0	0	0	0	0
