# Supplementary material for: Diatom Cell Size, Coloniality and Motility: Trade-Offs between Temperature, Salinity and Nutrient Supply with Climate Change
Source: PLoS One. 2014 Oct 3;9(10):e109993. doi: 10.1371/journal.pone.0109993 (PMC4184900; doi:10.1371/journal.pone.0109993)
Supplement: Table S3 — Dataset “Salinity” with 119 sites: Environmental and biotic variables. Abbreviations cf. Figure 6. (PDF) [file pone.0109993.s003.pdf]

Table S3. Dataset "Salinity" with 119 sites: Environmental and biotic variables. Abbreviations cf. Figure 6.

AREA	ABBR	SITE	SITE NAME	LATITUDE	LONGITUDE	SALIN	°C TEMP	µmol/L DIN	µmol/L DIP	µmol/L DSi	mol/mol N:P	1-5 scale EXP	mg/cm^2 SAND	1-6 scale BEACH	of 1000 RICHN	mg/cm^2 ADW	% of DW ADW%
Hanöbukten	Han	15	Listershuvud	56° 02' 05" N	14° 46' 55" E	7.8	3.3	0.36	0.23	1.07	1.58	5	0.098	6	28	0.914	34.6
Hanöbukten	Han	16	Listershuvud	56° 02' 08" N	14° 46' 53" E	7.1	6.0	1.29	16.13	2.14	0.08	1	0.042	3	26	1.115	31.9
Hanöbukten	Han	17	Hörvik	56° 02' 32" N	14° 46' 10" E	7.3	5.3	0.21	0.19	0.71	1.11	2	0.551	3	35	2.651	55.9
Hanöbukten	Han	18	Knutsäng	56° 02' 35" N	14° 45' 37" E	7.1	4.0	0.43	0.65	2.86	0.66	4	0.124	1	29	1.374	53.6
Hanöbukten	Han	19	Mjällbynabben	56° 03' 12" N	14° 45' 27" E	7.3	4.0	0.36	0.42	3.21	0.85	4	0.295	3	38	6.025	43.0
Hanöbukten	Han	20	Nogersund	56° 00' 26" N	14° 44' 40" E	7.6	5.3	0.43	0.55	2.50	0.78	2	0.788	2	46	1.475	15.2
Hanöbukten	Han	21	Nogersund hamn	56° 00' 15" N	14° 44' 06" E	6.6	6.0	0.36	0.71	4.29	0.50	1	0.376	3	42	8.610	13.9
Hanöbukten	Han	22	Getabjär	56° 01' 05" N	14° 45' 52" E	7.1	4.0	0.36	0.16	3.21	2.22	4	0.613	4	27	7.511	40.1
Hanöbukten	Han	23	Nogersund	56° 00' 18" N	14° 44' 23" E	6.2	4.6	0.43	0.74	2.50	0.58	3	1.533	3	31	4.339	31.4
Kalmarsund	Kal	25	Figeholm	57° 22' 10" N	16° 33' 19" E	6.2	2.7	0.50	0.55	6.07	0.91	3	0.009	3	36	3.250	49.6
Kalmarsund	Kal	26	Djupviken	57° 21' 26" N	16° 33' 16" E	7.3	2.0	0.50	0.52	5.00	0.97	4	0.013	3	33	6.230	54.1
Kalmarsund	Kal	29	Långholme	57° 20' 59" N	16° 33' 40" E	7.1	2.0	0.93	1.55	7.86	0.60	4	0.035	5	29	5.207	47.5
Kalmarsund	Kal	30	Lökö	57° 20' 48" N	16° 34' 32" E	7.0	1.3	0.50	1.84	7.14	0.27	5	0.024	5	33	3.394	57.3
Kalmarsund	Kal	31	Kråkelund	57° 26' 48" N	16° 43' 42" E	7.5	1.3	0.50	0.74	2.50	0.67	5	0.007	6	39	6.110	61.8
Kalmarsund	Kal	32	Kråkelund	57° 26' 45" N	16° 43' 32" E	7.0	3.3	0.64	0.71	3.21	0.91	2	0.114	4	34	8.192	52.8
Kvädöfjärden	Kvä	33	Skogsholmen	58° 00' 25" N	16° 44' 25" E	6.8	7.9	0.29	0.03	6.49	8.92	2	0.209	5	42	2.675	28.4
Kvädöfjärden	Kvä	34	Skogsholmen	58° 00' 20" N	16° 44' 19" E	6.7	6.5	0.29	0.45	4.29	0.63	4	0.063	5	45	4.753	44.3
Kvädöfjärden	Kvä	35	Östra Ed	58° 01' 46" N	16° 39' 52" E	6.6	7.2	0.64	1.32	8.21	0.49	3	1.404	2	48	3.093	26.7
Kvädöfjärden	Kvä	36	Sandviken	58° 00' 47" N	16° 40' 16" E	6.5	7.2	0.43	0.35	4.64	1.21	3	0.528	2	44	3.706	30.0
Kvädöfjärden	Kvä	37	Björnhällen	57° 59' 49" N	16° 41' 21" E	6.7	6.5	0.36	0.42	6.43	0.85	4	0.308	5	49	3.979	24.8
Kvädöfjärden	Kvä	38	Berkholmen	57° 59' 39" N	16° 43' 47" E	6.2	6.5	0.36	0.29	5.71	1.23	4	0.030	5	49	3.098	46.0
Kvädöfjärden	Kvä	39	Skogsholmen	58° 00' 30" N	16° 44' 15" E	7.0	7.2	0.36	0.52	7.50	0.69	3	0.704	5	53	2.812	33.2
Kvädöfjärden	Kvä	40	Högholmen	58° 00' 15" N	16° 46' 42" E	6.1	6.5	0.29	0.23	2.86	1.26	4	0.171	5	41	5.223	49.4
Kvädöfjärden	Kvä	41	Fjärdholmen	58° 01' 07" N	16° 46' 04" E	6.9	8.6	0.36	0.29	3.93	1.23	1	0.044	2	34	1.837	47.2
Kvädöfjärden	Kvä	42	Stora Rötskär	58° 01' 12" N	16° 47' 12" E	7.1	6.5	0.29	0.65	5.36	0.44	4	0.026	5	28	3.414	54.3
Kvädöfjärden	Kvä	44	Torrö	58° 00' 28" N	16° 48' 39" E	7.0	7.9	0.29	0.42	3.57	0.68	2	0.009	5	32	2.352	55.3
Himmerfjärden	Him	45	Sibble	59° 06' 18" N	17° 45' 17" E	5.6	7.2	2.57	0.03	4.64	80.33	3	1.053	1	55	2.982	14.3
Himmerfjärden	Him	46	Sandviken	59° 03' 14" N	17° 41' 27" E	3.5	6.5	0.50	0.03	3.21	15.62	4	0.518	5	44	4.291	32.3
Himmerfjärden	Him	47	Sandviken	59° 03' 04" N	17° 41' 44" E	5.1	6.5	0.71	0.10	3.57	7.39	4	0.319	2	41	6.815	30.6
Himmerfjärden	Him	48	Salsudd	59° 02' 34" N	17° 41' 46" E	5.1	7.2	0.71	0.03	1.43	22.31	3	2.319	2	44	1.904	30.9
Himmerfjärden	Him	49	Mölleviken	59° 02' 17" N	17° 41' 30" E	5.4	6.5	0.50	0.10	6.07	5.17	4	4.049	1	43	7.500	23.1
Himmerfjärden	Him	50	Axviken	59° 01' 06" N	17° 41' 48" E	5.6	7.9	0.71	0.03	5.36	22.30	2	0.618	2	60	4.151	13.0
Himmerfjärden	Him	51	Oaxen	58° 58' 09" N	17° 42' 24" E	5.8	6.5	0.21	0.03	3.93	6.69	4	0.802	3	37	4.559	30.8
Himmerfjärden	Him	52	Tunanäs	58° 59' 01" N	17° 42' 27" E	5.8	6.5	0.21	0.45	7.50	0.47	4	7.503	3	60	4.570	9.3
Himmerfjärden	Him	53	Tunaviken	58° 59' 05" N	17° 41' 58" E	5.8	7.9	0.21	0.29	2.50	0.74	2	0.432	2	47	5.166	21.2
Himmerfjärden	Him	55	Ekö	58° 54' 48" N	17° 41' 36" E	6.1	5.9	0.43	0.03	2.86	13.39	5	0.033	6	49	8.513	39.6
Himmerfjärden	Him	56	Ekö	58° 54' 38" N	17° 41' 36" E	6.3	6.5	0.36	0.03	3.57	11.15	4	0.334	2	37	4.490	33.6
Himmerfjärden	Him	57	Ekö	58° 54' 44" N	17° 41' 23" E	6.2	7.9	0.79	0.32	6.43	2.44	2	0.210	2	46	2.166	20.4
Gräsö	Grä	59	Örkärssundet	60° 30' 14" N	18° 23' 37" E	5.1	10.4	1.07	0.03	10.00	33.21	1	0.075	2	73	4.725	18.4
Gräsö	Grä	60	Örkärssundet	60° 30' 18" N	18° 23' 35" E	5.1	8.4	0.43	0.03	6.07	13.29	4	0.045	5	66	1.922	29.9
Gräsö	Grä	61	Örkärssundet	60° 30' 29" N	18° 23' 58" E	5.1	7.7	0.64	0.03	5.71	19.93	5	0.027	6	52	2.450	33.7
Gräsö	Grä	62	Örkärssundet	60° 30' 24" N	18° 23' 57" E	5.1	8.4	0.79	0.03	6.07	24.36	4	0.096	2	57	3.117	26.8
Gräsö	Grä	63	Björnören	60° 24' 49" N	18° 32' 23" E	5.1	7.7	0.86	0.03	7.50	26.57	5	0.021	6	52	4.265	36.0
Gräsö	Grä	64	Björnören	60° 24' 53" N	18° 28' 22" E	5.1	8.4	0.86	0.03	6.79	26.57	4	0.070	3	57	2.515	32.6
Gräsö	Grä	65	Björnören	60° 24' 55" N	18° 28' 15" E	5.1	9.7	0.86	0.13	6.43	6.64	2	2.729	2	56	2.664	24.9
Gräsö	Grä	66	Djursten	60° 22' 27" N	18° 24' 21" E	5.1	9.1	0.71	0.03	6.43	22.14	3	0.224	2	52	0.897	16.6
Gräsö	Grä	67	Djursten	60° 22' 18" N	18° 24' 18" E	5.0	10.4	1.43	0.03	5.71	44.29	1	0.497	2	65	4.472	8.6
Gräsö	Grä	68	Djursten	60° 22' 14" N	18° 24' 11" E	5.1	8.4	0.93	0.03	4.64	28.79	4	0.214	6	53	2.849	36.9
Gävlebukten	Gäv	101	Eskön	60° 53' 34" N	17° 21' 23" E	5.1	1.0	0.36	0.29	7.86	1.23	5	0.005	5	35	1.097	47.7
Gävlebukten	Gäv	102	Eskön	60° 55' 57" N	17° 20' 25" E	5.1	2.3	0.21	0.13	9.64	1.66	3	0.009	5	55	1.309	30.2
Gävlebukten	Gäv	103	Eskön	60° 56' 09" N	17° 20' 37" E	5.0	1.7	0.21	0.03	5.71	6.64	4	0.029	5	33	4.245	45.8
Gävlebukten	Gäv	104	Eskön	60° 56' 00" N	17° 19' 15" E	5.1	2.3	0.21	0.06	7.86	3.32	3	0.086	5	57	2.971	24.2

Gävlebukten	Gäv	105	Eskön	60° 51' 55" N	17° 18' 52" E	4.9	3.0	0.86	0.52	6.43	1.66	2	0.240	5	61	5.251	27.9
Gävlebukten	Gäv	106	Eskön	60° 52' 02" N	17° 18' 55" E	4.6	3.7	1.00	0.26	7.50	3.88	1	0.266	2	59	1.865	28.9
Gävlebukten	Gäv	119	Gåsholma	61° 00' 16" N	17° 14' 42" E	4.8	3.7	2.00	0.29	15.36	6.89	1	0.247	2	78	2.886	24.0
Gävlebukten	Gäv	121	Kusön	61° 02' 53" N	17° 14' 10" E	4.4	1.0	0.79	0.03	20.36	24.36	5	0.007	5	29	1.431	53.7
Gävlebukten	Gäv	122	Kusön	61° 02' 38" N	17° 15' 17" E	4.4	1.7	0.43	0.03	17.50	13.29	4	0.049	5	19	1.091	33.1
Gävlebukten	Gäv	123	Kusön	61° 01' 51" N	17° 14' 57" E	4.5	3.0	0.43	0.03	13.57	13.29	2	0.020	1	59	1.599	29.4
Horslandet	Hor	125	Arnöviken	61° 42' 27" N	17° 23' 03" E	5.1	5.0	0.50	0.42	15.71	1.19	3	0.168	5	47	1.745	31.1
Horslandet	Hor	126	Rikstensberget	61° 43' 32" N	17° 24' 53" E	4.6	4.4	0.36	0.23	12.86	1.58	4	2.753	5	58	5.663	11.8
Horslandet	Hor	127	Fisklösviken	61° 43' 58" N	17° 27' 09" E	5.0	5.0	0.36	0.06	16.43	5.54	3	0.037	5	44	4.276	39.6
Horslandet	Hor	128	Björnviken	61° 43' 35" N	17° 28' 04" E	4.8	5.7	0.21	0.06	16.79	3.32	2	0.009	5	51	2.579	51.4
Horslandet	Hor	129	Björnviken	61° 43' 46" N	17° 28' 08" E	4.8	4.4	0.36	0.03	16.79	11.07	4	0.034	5	53	2.151	46.1
Horslandet	Hor	130	Notholmsharet	61° 43' 16" N	17° 32' 35" E	5.0	3.7	0.36	0.10	17.50	3.69	5	0.057	5	31	5.020	44.2
Horslandet	Hor	131	Notholmsharet	61° 43' 16" N	17° 32' 45" E	5.0	3.7	0.36	0.16	17.50	2.21	5	0.440	5	45	7.184	28.4
Horslandet	Hor	132	Tångviken	61° 43' 18" N	17° 32' 44" E	5.0	5.7	0.36	0.16	10.71	2.21	2	7.071	2	46	5.711	18.7
Horslandet	Hor	133	Skräddarharet	61° 43' 35" N	17° 31' 12" E	5.0	4.4	0.79	0.06	13.57	12.18	4	0.029	5	43	3.211	42.8
Horslandet	Hor	134	Bålsö	61° 43' 46" N	17° 31' 30" E	5.0	6.4	0.36	0.16	15.36	2.21	1	0.280	2	68	1.979	11.0
Omnefjärden	Omn	136	Rävsö	62° 55' 32" N	18° 30' 29" E	5.0	1.4	0.36	0.35	16.79	1.01	5	0.398	5	25	2.989	39.3
Omnefjärden	Omn	137	Rammberget	62° 57' 22" N	18° 30' 27" E	5.0	2.1	0.86	0.19	17.86	4.43	4	0.001	6	19	0.377	30.7
Omnefjärden	Omn	138	Trollarviken	62° 57' 44" N	18° 30' 21" E	4.7	2.7	0.50	0.13	20.36	3.88	3	0.485	2	61	3.173	7.0
Omnefjärden	Omn	139	Rävelviken	62° 56' 32" N	18° 28' 29" E	5.0	2.7	4.14	0.42	20.71	9.88	3	1.588	5	73	2.365	10.0
Omnefjärden	Omn	140	Bossviksberget	62° 58' 00" N	18° 26' 34" E	5.0	2.7	2.14	0.19	24.64	11.07	3	0.418	5	64	4.740	26.3
Omnefjärden	Omn	141	Storöran	62° 57' 21" N	18° 25' 57" E	5.0	2.1	0.36	0.29	15.00	1.23	4	0.440	5	59	2.103	22.6
Omnefjärden	Omn	142	Storöran	62° 57' 22" N	18° 26' 34" E	5.0	1.4	0.36	0.03	15.00	11.07	5	0.394	5	51	5.118	31.1
Omnefjärden	Omn	143	Måviken-hamn	62° 58' 02" N	18° 25' 22" E	5.0	2.7	0.43	0.32	16.07	1.33	3	0.722	5	66	2.587	20.5
Omnefjärden	Omn	144	Mjällomsviken	62° 58' 40" N	18° 25' 09" E	5.1	2.7	1.43	0.39	17.14	3.69	3	0.350	2	51	1.451	17.7
Omnefjärden	Omn	145	Mjällomsviken	62° 58' 14" N	18° 24' 45" E	4.7	3.4	1.29	0.16	20.36	7.97	2	0.152	2	67	0.604	31.6
Omnefjärden	Omn	146	Björnånäset	62° 57' 53" N	18° 24' 33" E	4.3	3.4	0.50	0.32	28.93	1.55	2	0.181	2	49	2.439	28.1
Holmön	Hol	147	Stor Fjäderägg	63° 48' 44" N	21° 00' 35" E	3.1	1.4	3.00	0.16	18.57	8.40	5	0.055	5	38	1.861	35.4
Holmön	Hol	148	Stor Fjäderägg	63° 48' 05" N	21° 00' 05" E	3.0	2.1	2.36	0.03	18.21	33.00	4	0.766	5	49	2.790	15.0
Holmön	Hol	149	Långögern	63° 47' 31" N	20° 57' 53" E	3.2	3.4	3.79	0.19	22.86	8.83	2	0.050	5	57	2.856	33.2
Holmön	Hol	150	Långögern	63° 47' 32" N	20° 58' 01" E	3.2	2.1	1.71	0.16	19.29	4.80	4	0.029	5	39	2.284	28.8
Holmön	Hol	151	Gåsflötan	63° 47' 18" N	20° 56' 49" E	3.2	2.7	4.64	0.03	26.07	65.00	3	0.029	2	59	2.664	23.8
Holmön	Hol	152	Svärtögern	63° 48' 05" N	20° 55' 52" E	3.1	2.1	3.64	0.13	22.86	12.75	4	0.068	5	57	1.811	27.1
Holmön	Hol	153	Lill Fjäderägg	63° 48' 49" N	20° 55' 59" E	3.3	4.1	1.36	0.10	16.07	6.33	1	0.511	2	75	2.688	21.0
Holmön	Hol	154	Gädobäckssundet	63° 45' 50" N	20° 53' 20" E	2.9	3.4	2.36	0.16	23.57	6.60	2	0.026	5	68	1.329	30.4
Holmön	Hol	155	Sörsundet	63° 44' 52" N	20° 52' 44" E	3.2	1.4	0.29	0.13	13.21	1.00	5	0.643	5	65	3.877	20.0
Holmön	Hol	156	Sörviken	63° 45' 43" N	20° 51' 16" E	3.3	3.4	2.21	2.19	16.07	0.46	2	0.015	5	63	3.309	28.2
Holmön	Hol	157	Gåsflötanögern	63° 45' 30" N	20° 49' 12" E	3.1	1.4	3.00	0.45	18.21	3.00	5	0.021	5	24	1.478	37.8
Holmön	Hol	158	Gåsflötanögern	63° 45' 34" N	20° 49' 19" E	3.3	1.4	1.93	0.03	15.36	27.00	5	0.056	5	26	1.843	42.2
Holmön	Hol	159	Vedaögern	63° 46' 34" N	20° 49' 34" E	3.0	3.4	2.00	0.03	13.93	28.00	2	0.432	6	48	1.193	21.7
Holmön	Hol	160	Lillhällan	63° 48' 40" N	20° 51' 44" E	3.0	1.4	5.43	0.10	25.00	25.33	5	0.011	6	30	0.737	31.6
Skelleftehamn	Ske	162	Nygrundet	64° 40' 26" N	21° 20' 49" E	2.4	1.0	4.14	0.03	26.79	58.00	5	0.010	5	35	0.995	17.4
Skelleftehamn	Ske	163	Nygrundet	64° 40' 29" N	21° 20' 36" E	2.5	1.7	4.57	0.06	29.29	32.00	4	0.027	5	45	0.757	17.3
Skelleftehamn	Ske	164	Storgrundet	64° 42' 04" N	21° 16' 49" E	2.4	1.7	3.79	0.03	25.71	53.00	4	0.025	5	51	1.682	18.8
Skelleftehamn	Ske	166	Skataudden	64° 44' 22" N	21° 13' 00" E	2.8	1.7	5.14	0.26	25.71	9.00	4	0.676	5	40	2.450	13.7
Skelleftehamn	Ske	167	Kågnäsudden	64° 44' 43" N	21° 17' 29" E	2.8	1.7	4.00	0.10	25.36	18.67	4	0.050	5	49	1.540	14.3
Skelleftehamn	Ske	168	Djupskär	64° 42' 57" N	21° 16' 00" E	2.6	1.7	4.64	0.10	27.50	21.67	4	0.018	5	49	0.497	11.5
Skelleftehamn	Ske	169	Djupviken	64° 42' 19" N	21° 15' 11" E	2.4	3.7	4.00	0.03	31.07	56.00	1	0.039	5	69	1.826	17.9
Skelleftehamn	Ske	171	Bredskär	64° 41' 10" N	21° 18' 43" E	2.6	3.0	2.57	0.03	22.86	36.00	2	0.249	5	63	4.797	14.3
Skelleftehamn	Ske	172	Bredskär	64° 41' 03" N	21° 18' 34" E	2.7	1.7	1.93	0.03	22.50	37.00	4	0.475	5	53	4.627	19.1
Skelleftehamn	Ske	173	Gåsören	64° 39' 56" N	21° 18' 56" E	2.7	1.7	5.79	0.06	30.71	40.50	4	0.005	5	33	0.674	27.0
Skelleftehamn	Ske	174	Nörd Olsgrundet	64° 39' 22" N	21° 16' 05" E	2.1	1.7	2.43	0.19	21.43	5.67	4	0.011	5	18	0.665	26.9
Skelleftehamn	Ske	175	Vi. Kalkgrundet	64° 36' 36" N	21° 18' 59" E	1.6	2.3	3.57	0.13	37.50	12.50	3	0.004	5	22	0.679	37.0
Skelleftehamn	Ske	176	Långskuttun	64° 37' 49" N	21° 17' 01" E	2.4	1.0	5.36	0.03	33.21	75.00	5	0.006	5	28	0.678	32.6
Skelleftehamn	Ske	177	Sör Olsgrundet	64° 38' 34" N	21° 16' 27" E	2.1	1.7	4.79	0.10	29.64	22.33	4	0.045	5	41	1.049	15.6
Rånefjärden	Rån	178	Lill Långöran	65° 46' 10" N	22° 28' 06" E	1.2	1.0	3.43	0.26	48.93	6.00	5	0.057	5	64	1.690	13.1
Rånefjärden	Rån	179	Skorven	65° 46' 43" N	22° 27' 37" E	0.9	1.7	4.21	0.16	52.14	11.80	4	0.099	5	73	1.577	10.3
Rånefjärden	Rån	180	Långholmen	65° 47' 34" N	22° 29' 04" E	0.8	1.7	1.43	0.03	43.21	20.00	4	0.034	5	79	1.143	13.1
Rånefjärden	Rån	181	Köpmanholmen	65° 48' 41" N	22° 29' 07" E	0.5	2.3	2.64	0.52	40.00	2.31	3	0.120	5	99	1.933	9.3

Rånefjärden	Rån	182	Hällholmen	65° 49' 14" N	22° 27' 37" E	0.5	2.3	3.57	0.06	50.89	35.00	4	0.147	4	71	1.887	9.6
Rånefjärden	Rån	185	Lill Furuön	65° 39' 14" N	22° 42' 03" E	2.3	1.0	7.29	0.03	44.64	102.00	5	0.001	6	78	0.373	47.2
Rånefjärden	Rån	186	Fjuksön	65° 39' 30" N	22° 38' 16" E	2.2	1.0	4.21	0.32	38.21	5.90	5	0.049	4	51	1.803	11.8
Rånefjärden	Rån	187	Rövarklubben	65° 40' 42" N	22° 39' 43" E	2.5	1.7	6.00	0.39	42.50	7.00	4	0.093	5	55	2.263	13.2
Rånefjärden	Rån	189	Fjuksögrundet	65° 42' 54" N	22° 35' 33" E	1.8	1.7	5.36	0.13	45.00	18.75	4	0.018	5	51	1.696	16.5
Rånefjärden	Rån	190	Lövören	65° 43' 22" N	22° 35' 49" E	1.8	2.3	5.93	0.16	51.07	16.60	3	0.046	5	78	2.003	12.3
Rånefjärden	Rån	191	Siksundsön	65° 45' 02" N	22° 40' 14" E	2.3	2.3	6.43	0.10	46.43	30.00	3	0.056	5	64	4.088	21.6
Rånefjärden	Rån	192	Kungsörarna	65° 45' 11" N	22° 41' 46" E	2.7	1.7	7.36	0.29	48.21	11.44	4	0.243	5	73	3.237	18.3
