# Supplementary material for: Diatom Cell Size, Coloniality and Motility: Trade-Offs between Temperature, Salinity and Nutrient Supply with Climate Change
Source: PLoS One. 2014 Oct 3;9(10):e109993. doi: 10.1371/journal.pone.0109993 (PMC4184900; doi:10.1371/journal.pone.0109993)
Supplement: Table S4 — Dataset “Salinity” with 119 sites and 355 diatom taxa: 1000 valves/sample. (PDF) [file pone.0109993.s004.pdf]

Table S4. Dataset "Salinity" with 119 sites and 355 diatom taxa: 1000 valves/sample

AREA	Han	Han	Han	Han	Han	Han	Han	Han	Han
SITE	15	16	17	18	19	20	21	22	23
Acd_exil	0	0	0	0	0	0	0	0	0
Acd_minu	0	0	0	0	0	0	0	0	0
Acd_pyre	0	0	0	0	0	0	0	0	0
Ach_brev	0	0	0	0	8	0	0	0	0
Ach_brin	0	0	0	0	4	1	0	0	0
Ach_foge	0	0	0	0	0	0	0	0	0
Ach_leon	0	0	0	0	0	0	0	0	0
Ach_long	0	0	0	0	2	0	0	0	0
Ach_luth	0	0	0	0	0	0	0	0	0
Ach_misc	3	0	0	0	0	2	0	0	0
Ach_vist	0	0	0	0	0	0	0	0	0
Act_nosu	0	0	0	0	0	0	0	0	0
Act_occr	0	0	0	3	0	0	0	0	0
Ami_spec	0	0	0	0	0	0	0	0	0
Amp_copu	0	0	0	0	0	0	0	0	0
Amp_fleb	0	0	0	0	0	0	0	0	0
Amp_hele	0	0	0	0	0	2	0	0	0
Amp_inar	0	0	0	0	0	0	0	0	0
Amp_line	0	0	0	0	0	0	6	0	2
Amp_micr	0	0	0	0	0	0	0	0	0
Amp_oval	0	0	0	0	0	0	0	0	0
Amp_pedi	0	0	0	0	2	8	2	4	0
Amp_prof	0	0	0	0	0	0	0	0	0
Ane_mino	0	0	0	0	0	0	0	0	0
Ane_tusc	0	0	0	0	0	0	0	0	0
Ano_spha	0	0	0	0	0	0	0	0	0
Asa_bahu	0	0	0	0	0	0	0	0	0
Ast_form	0	0	0	0	0	0	0	0	0
Aul_spp	0	0	0	2	0	0	0	0	0
Bac_paxi	0	0	0	0	0	0	0	0	0
Ber_fenn	0	0	0	0	0	0	0	4	2
Ber_ruti	66	33	21	36	35	77	187	73	9
Ber_scop	0	0	0	0	0	0	14	0	0
Bir_luce	0	0	0	0	0	0	0	0	0
Bra_apon	0	0	0	0	0	0	0	0	0
Bra_vitr	0	0	0	0	0	0	0	0	0
Bre_lanc	0	0	2	0	0	0	0	0	0

Cal_aemu	0	0	0	0	0	0	0	0	0
Cal_amph	0	0	0	0	0	0	0	0	0
Cal_baci	0	0	0	0	0	0	0	0	0
Cal_cras	0	0	0	0	0	0	0	0	0
Cal_sili	0	0	0	0	0	0	0	0	0
Cam_clyp	0	0	0	0	0	0	0	0	0
Cat_adha	0	2	0	0	0	0	0	0	0
Cav_lapi	0	0	0	0	0	0	0	0	0
Cav_pseu	0	0	0	0	0	0	0	0	0
Cer_clos	0	0	0	0	0	0	0	0	0
Chp_clam	0	0	0	0	0	0	0	0	0
Chp_marg	0	0	0	0	0	0	0	0	0
Coc_cost	1	9	2	0	0	13	0	0	2
Coc_neot	0	31	20	13	20	14	0	0	0
Coc_pedi	19	28	21	19	48	39	1	16	8
Coc_pelt	0	0	0	0	0	0	0	0	0
Coc_plac	4	12	13	3	0	17	1	16	8
Coc_pleu	0	0	0	0	0	0	0	0	0
Coc_plli	0	0	0	0	0	0	0	0	0
Coc_psli	0	0	0	0	0	0	0	0	0
Coc_psth	0	0	0	0	0	0	0	0	0
Coc_scut	62	55	74	53	66	40	0	43	21
Coc_stau	11	46	50	51	40	15	0	0	0
Com_pusi	0	0	0	0	0	0	0	0	0
Cos_aste	0	0	0	0	0	0	0	0	0
Cte_pulc	0	0	0	0	0	0	0	0	0
Cyc_atom	0	0	0	0	0	0	0	0	0
Cyc_choc	0	0	0	0	0	0	0	0	0
Cyc_mene	0	0	0	0	0	0	0	0	0
Cyc_radi	0	0	0	0	0	0	0	0	0
Cyc_ross	0	0	0	0	0	0	0	0	0
Cyc_stel	0	0	0	0	0	0	0	0	0
Cyc_stri	0	0	0	0	0	0	0	0	0
Cyc_trip	0	0	0	0	0	0	0	0	0
Cym_affi	0	0	0	0	0	0	0	0	0
Cym_cist	0	0	0	0	0	0	0	0	0
Cym_helv	0	0	0	0	0	0	0	0	0
Cym_lanc	0	0	0	0	0	0	0	0	0
Cym_prox	0	0	0	0	0	0	0	0	0
Cyp_sole	0	0	0	0	0	0	0	0	0
Cys_dubi	0	0	0	0	0	0	0	0	0

Den_tecr	0	0	0	0	0	0	0	0	0
Dia_bott	0	0	0	0	0	0	0	0	0
Dia_moni	0	0	0	0	0	1	1	0	0
Dia_prob	0	0	0	0	0	0	0	0	0
Dia_tenu	0	0	0	0	0	0	0	0	0
Dia_vulg	0	0	0	0	0	0	0	0	0
Dic_subi	0	0	0	0	0	10	0	0	0
Dip_depa	0	0	0	0	0	0	0	0	0
Dip_dydi	0	0	0	0	0	0	0	0	0
Dip_elli	0	0	0	0	0	0	0	0	0
Dip_inte	0	0	0	0	0	2	0	0	0
Dip_ocul	0	0	0	0	0	0	0	0	0
Dip_smdi	0	0	0	0	0	0	0	0	0
Dip_smpu	0	0	0	0	0	0	0	0	0
Dip_smrh	0	0	0	0	0	2	0	0	0
Dip_subo	0	0	0	0	0	0	0	0	0
Enc_caes	0	0	0	0	0	0	0	0	0
Enc_lacu	0	0	0	0	0	0	0	0	0
Enc_minu	0	0	0	0	0	0	0	0	0
Enc_obsc	0	0	0	0	0	0	0	0	0
Enc_pros	0	0	0	0	0	0	0	0	0
Enc_sile	0	0	0	0	0	0	0	0	0
Enc_tenu	0	0	0	0	0	0	0	0	0
Enp_behr	0	0	0	0	0	0	0	0	0
Enp_kram	0	0	0	0	0	0	0	0	0
Ent_palu	0	0	0	0	0	0	0	0	0
Ent_pseu	0	0	0	0	0	0	0	0	0
Ent_punc	0	0	0	0	0	0	0	0	0
Epi_adna	0	0	0	0	0	0	0	0	0
Epi_goep	0	0	0	0	0	0	0	0	0
Epi_sore	0	0	0	10	15	0	0	0	0
Epi_turg	0	0	0	0	0	0	0	0	0
Epi_tuwe	2	0	0	0	0	0	0	0	0
Euc_laev	0	0	0	0	0	0	0	0	0
Eun_arcu	0	0	0	0	0	0	0	0	0
Eun_peun	0	0	0	0	0	0	0	0	0
Fal_clep	0	0	0	0	2	0	0	0	0
Fal_cryp	0	0	0	0	0	0	0	0	0
Fal_flor	0	0	0	0	0	0	0	0	0
Fal_psny	0	0	0	0	0	0	0	0	0
Fal_pygm	0	0	0	0	2	0	0	0	0

Fis_sapr	0	0	0	0	0	0	0	0	0
Fra_amic	0	0	0	0	0	0	0	0	0
Fra_capu	0	0	0	0	0	0	0	0	0
Fra_caru	0	0	0	0	0	0	0	0	0
Fra_cava	0	0	0	0	0	0	0	0	0
Fra_cons	3	0	0	0	0	0	0	0	0
Fra_geda	0	0	0	0	0	0	0	0	0
Fra_grac	0	0	0	0	0	0	0	0	0
Fra_hydu	0	0	0	0	0	0	0	0	0
Fra_lept	0	0	0	0	0	0	0	0	0
Fra_nitz	0	0	0	0	0	0	0	0	0
Frp_cyli	0	0	0	0	0	0	0	0	0
Fru_vulg	0	0	0	0	0	0	0	0	0
Gom_clav	0	0	0	0	0	0	0	0	0
Gom_intr	0	0	0	0	0	0	0	0	0
Gom_oliv	0	4	13	8	6	8	2	0	0
Gom_parv	0	0	0	0	0	6	0	0	0
Gon_exig	0	0	0	0	0	4	0	0	0
Gos_expl	0	0	0	0	0	0	0	0	0
Gos_obsc	0	6	2	0	10	8	2	6	6
Gra_mari	0	0	0	0	0	0	0	0	0
Gra_ocea	15	16	4	2	10	21	1	12	2
Gyr_acum	0	0	0	0	2	0	0	0	2
Gyr_atte	0	0	0	0	0	0	0	0	0
Gyr_stri	0	0	0	0	0	0	0	0	0
Gyr_tenu	0	0	0	0	0	0	0	0	0
Hal_coff	0	0	0	0	0	0	4	0	0
Hal_exig	0	0	0	0	0	0	0	0	0
Hal_hols	0	0	0	0	0	0	0	0	0
Hal_hybr	0	0	0	0	0	0	0	0	0
Hal_luci	0	0	2	0	0	0	4	0	0
Hal_norm	0	0	0	0	0	0	0	0	0
Hal_subh	0	0	0	0	0	0	0	0	0
Hal_tene	0	0	4	0	2	0	0	0	0
Hal_vene	0	0	0	0	0	0	0	0	0
Han_balt	0	0	0	0	0	0	0	0	0
Has_spic	0	0	0	0	0	0	0	2	0
Hip_capi	0	0	0	0	0	0	0	0	0
Hip_hung	0	0	0	0	0	0	0	0	0
Hip_lesm	0	0	0	0	0	0	0	0	0
Kar_amoe	0	0	0	0	0	0	0	0	0

Kar_clev	0	0	0	0	0	0	0	0	0
Kar_niti	0	0	0	0	0	0	0	0	0
Kar_subm	0	0	0	0	0	0	0	0	0
Lemn_hung	0	0	0	0	0	0	0	0	0
Lic_debi	0	0	6	2	2	4	2	0	0
Lic_gran	0	0	0	0	0	0	3	0	0
Lic_oedi	2	2	0	0	0	0	4	0	6
Lic_rhom	0	0	0	0	0	0	1	0	0
Lun_bise	0	0	0	0	0	0	0	0	0
Lut_muti	0	0	0	0	0	0	0	0	0
Mar_atom	0	0	0	0	0	0	0	0	0
Mar_mart	0	0	0	0	0	0	0	0	0
Mar_schu	0	0	0	0	0	0	0	0	0
Mas_balt	0	0	0	0	0	0	0	0	0
Mas_brau	0	0	0	0	0	0	0	0	0
Mas_elli	0	0	0	0	0	0	0	0	0
Mas_exig	0	0	0	0	0	0	0	0	0
Mas_pumi	0	0	0	0	0	0	0	0	0
Mas_pusi	0	0	0	0	0	0	0	0	0
Mas_smam	0	0	0	0	0	0	0	0	0
Mas_smit	0	0	0	0	0	0	0	0	0
Mel_arct	0	0	0	0	0	0	0	0	0
Mel_line	0	0	0	0	0	0	0	0	0
Mel_moni	0	0	2	11	1	0	0	0	0
Mel_numm	0	0	6	2	2	0	3	0	0
Mel_vari	0	0	0	0	0	0	0	0	0
Nav_aren	0	0	0	0	0	0	0	0	0
Nav_bipu	0	0	0	0	0	0	0	0	0
Nav_boss	0	8	3	2	2	0	0	0	0
Nav_bott	6	0	0	0	0	0	0	4	0
Nav_brem	0	0	0	0	0	0	0	0	0
Nav_capi	0	0	0	0	0	0	0	0	0
Nav_cinc	0	0	0	0	0	0	0	0	0
Nav_clem	0	0	2	0	0	0	0	0	0
Nav_cryp	0	0	0	0	5	15	4	0	0
Nav_digi	0	0	0	0	0	0	0	0	0
Nav_duer	0	0	0	0	0	0	0	0	0
Nav_eidr	0	0	0	0	0	0	0	0	0
Nav_flan	0	0	0	0	0	0	0	0	0
Nav_geii	0	0	0	0	0	4	0	0	0
Nav_gepo	0	0	0	0	0	0	0	0	0

Nav_greg	0	0	6	0	2	1	26	0	0
Nav_haca	0	0	0	0	0	0	0	0	0
Nav_hani	0	1	0	0	0	0	0	0	0
Nav_hust	0	0	0	0	0	0	0	0	0
Nav_lanc	2	0	13	1	2	28	30	26	15
Nav_late	0	0	0	0	0	0	0	0	0
Nav_marg	0	0	0	0	0	0	0	0	0
Nav_meni	0	0	0	0	0	2	0	0	0
Nav_midi	0	0	0	0	0	0	0	0	0
Nav_nole	0	0	0	0	0	0	0	0	0
Nav_oblo	0	0	0	0	0	0	0	0	0
Nav_oest	0	0	0	0	0	0	0	0	0
Nav_pere	0	0	0	0	0	2	0	0	0
Nav_perm	197	147	97	57	152	128	136	230	157
Nav_phyl	0	0	0	0	0	8	26	24	12
Nav_port	0	0	0	0	0	0	0	0	0
Nav_radi	0	0	0	0	0	0	0	0	0
Nav_ramo	74	108	73	69	81	9	69	75	69
Nav_rato	2	0	0	0	0	1	0	0	0
Nav_rece	0	0	0	0	0	0	0	0	0
Nav_rhyn	0	0	0	0	0	0	12	0	0
Nav_sana	0	0	0	0	0	1	32	0	0
Nav_sjoe	11	14	3	4	2	5	0	6	6
Nav_star	0	0	0	0	0	0	0	0	0
Nav_subr	0	0	0	0	0	0	0	0	0
Nav_supr	0	0	0	0	0	0	0	0	0
Nav_trip	0	0	0	0	0	0	0	0	0
Nav_vene	0	0	0	0	0	0	0	0	0
Ncy_pusi	0	0	0	0	0	0	0	0	0
Nit_acic	0	0	0	0	0	0	0	0	0
Nit_anfo	0	0	0	0	0	0	0	0	0
Nit_anla	0	0	0	0	0	0	0	0	0
Nit_arch	0	0	0	0	0	0	0	0	0
Nit_aura	0	0	0	0	0	0	0	0	0
Nit_baci	0	0	0	6	0	2	0	0	0
Nit_dime	0	0	0	0	0	0	0	0	0
Nit_dipp	0	0	0	0	0	0	0	0	0
Nit_diss	0	0	0	0	0	0	0	0	0
Nit_dist	0	0	0	0	0	0	20	0	0
Nit_dubi	0	0	0	0	0	0	2	0	0
Nit_eleg	0	0	0	0	0	0	0	0	0

Nit_frig	0	0	0	0	0	0	0	0	0
Nit_frus	0	0	14	19	52	6	0	0	0
Nit_gros	0	0	0	0	0	0	0	0	0
Nit_heuf	0	0	0	0	0	0	0	0	0
Nit_inco	21	7	32	38	42	38	14	30	162
Nit_inte	0	0	0	0	0	0	0	0	0
Nit_lieb	0	0	0	0	0	0	0	0	0
Nit_lisu	0	0	0	0	0	0	0	0	0
Nit_lore	0	0	0	0	0	0	0	0	0
Nit_micr	0	0	0	0	22	7	78	18	6
Nit_pale	0	0	0	0	0	0	0	0	0
Nit_pcea	0	0	0	0	0	0	0	0	0
Nit_pell	0	0	0	0	0	0	0	0	0
Nit_perm	0	0	0	0	0	0	0	0	0
Nit_pusi	0	0	0	0	0	0	0	0	0
Nit_rect	0	0	0	0	0	0	0	0	0
Nit_rose	0	0	0	0	0	0	0	0	0
Nit_saco	0	0	0	0	0	0	11	0	0
Nit_sdea	0	0	0	0	0	0	0	0	0
Nit_sigm	0	0	0	0	0	0	0	0	0
Nit_soci	0	0	0	0	0	0	0	0	0
Nit_soli	0	0	0	0	0	0	0	0	0
Nit_subl	0	0	0	0	0	0	0	0	0
Nit_ther	0	0	0	0	0	0	0	0	0
Nit_umbo	0	0	0	0	0	0	0	0	0
Nit_vald	0	0	0	0	0	0	0	0	0
Nit_verm	0	0	0	0	0	0	0	0	0
Nit_vitr	0	0	0	0	0	0	0	0	0
Nit_wuel	0	0	0	0	0	0	0	0	0
Ope_krum	0	1	10	0	0	24	26	24	35
Ope_muta	16	12	38	16	2	36	155	77	29
Par_prot	0	0	0	0	0	0	0	0	0
Pau_taen	0	0	0	0	0	0	0	0	0
Pet_hume	0	0	2	2	0	0	0	0	0
Pin_appe	0	0	0	0	0	0	0	0	0
Pin_dimi	0	0	0	0	0	0	0	0	0
Pin_kroc	0	0	0	0	0	0	0	0	0
Pin_micr	0	0	0	0	0	0	0	0	0
Plc_plac	0	0	0	0	0	0	0	0	0
Plt_cons	0	0	0	0	0	0	0	0	0
Plt_deli	3	2	13	12	15	38	4	4	10

Plt_disp	0	0	0	0	0	0	0	0	0
Plt_freq	0	0	0	0	0	0	0	0	0
Plt_hauc	2	0	0	0	6	0	0	3	0
Plt_lanc	0	0	0	0	0	0	0	0	0
Plt_lemm	0	0	1	0	0	0	0	0	0
Plt_link	0	0	0	0	0	0	0	0	0
Plt_peri	0	0	0	0	0	0	0	0	5
Plt_rost	0	0	0	0	0	0	0	0	0
Plt_sept	0	0	0	0	0	0	0	3	4
Psf_tene	0	0	0	0	0	0	0	0	0
Pss_brev	0	0	0	0	0	0	0	0	0
Pss_brin	0	0	0	0	0	0	0	0	0
Pss_elli	0	0	0	0	0	0	6	0	6
Pss_perm	0	0	0	0	0	0	0	0	0
Pss_robu	0	0	0	0	0	0	0	0	0
Pss_subs	0	0	0	0	0	0	0	0	0
Pss_zeil	0	2	3	0	2	12	18	24	10
Pst_bior	0	0	0	0	0	0	0	0	0
Pst_oblo	0	0	0	0	0	0	0	0	0
Pst_punc	6	0	0	0	0	2	0	0	0
Pst_ross	0	0	0	0	0	0	0	0	0
Pst_sacc	0	0	0	0	0	0	0	0	0
Pte_inan	12	15	0	0	0	0	4	157	236
Rei_sinu	0	0	0	0	0	0	0	0	0
Rho_abbr	109	118	50	249	139	147	5	92	105
Rhp_acum	0	0	0	0	0	0	0	0	0
Rhp_breb	0	0	0	2	2	0	0	0	0
Rhp_gibb	0	0	0	0	2	0	0	0	0
Sel_pupu	0	0	0	0	0	0	0	0	0
Sel_stro	0	0	0	0	0	0	0	0	0
Ske_cost	0	0	0	0	0	0	0	0	0
Ske_subs	0	0	0	0	0	0	0	0	0
Sla_lapp	0	0	0	0	0	0	0	0	0
Std_hant	0	0	0	0	0	0	0	0	0
Std_parv	0	0	0	0	0	0	0	0	0
Stn_legu	0	0	0	0	0	0	0	0	0
Stn_phoe	0	0	0	0	0	0	0	0	0
Stn_simu	0	0	2	0	0	1	48	4	0
Stn_smka	0	0	0	0	0	0	0	0	0
Stp_anus	0	0	0	0	0	0	0	0	8
Stp_wisl	0	0	0	0	0	0	0	0	0

Sts_punc	0	0	0	0	0	0	0	0	0
Sts_vent	0	0	0	0	0	0	0	0	0
Sur_angu	0	0	0	0	0	0	0	0	0
Sur_brba	0	0	0	0	0	0	0	0	0
Sur_breb	0	0	0	0	0	0	0	0	0
Sur_minu	0	0	0	0	0	0	0	0	0
Sur_oval	0	0	0	0	0	0	0	0	0
Syn_acus	0	0	0	0	0	0	0	0	0
Syn_ulna	0	0	0	0	0	0	0	0	0
Tab_fasc	343	299	392	304	191	187	22	23	51
Tab_kten	0	0	0	0	0	0	0	0	0
Tab_laev	3	0	0	0	0	0	2	0	4
Tab_tabu	0	0	0	0	0	0	0	0	0
Tab_waer	2	0	0	0	0	0	0	0	0
Tal_fene	0	0	0	0	0	0	0	0	0
Tal_floc	0	0	0	0	0	0	0	0	0
Tet_glan	0	0	0	0	0	0	0	0	0
Tha_balt	0	0	0	0	0	0	0	0	0
Tha_hyla	0	0	0	0	0	0	0	0	0
Tha_leva	3	22	0	4	0	2	0	0	2
Tha_pros	0	0	4	0	0	0	0	0	0
Tha_pseu	0	0	0	0	0	0	0	0	0
Tha_punc	0	0	0	0	0	0	0	0	0
Try_angu	0	0	0	0	0	0	0	0	0
Try_apic	0	0	0	0	0	0	0	0	0
Try_ardu	0	0	0	0	0	0	0	0	0
Try_hung	0	0	0	0	0	0	10	0	0
Try_levi	0	0	0	0	0	0	0	0	0
Try_lite	0	0	0	0	0	0	2	0	0
Try_sali	0	0	0	0	0	0	0	0	0
Try_scal	0	0	0	0	0	0	0	0	0

AREA<br>SITE	Kal<br>25	Kal<br>26	Kal<br>29	Kal<br>30	Kal<br>31	Kal<br>32
Acd_exil	0	0	0	0	0	0
Acd_minu	2	0	0	0	0	0
Acd_pyre	0	0	0	0	0	0
Ach_brev	0	0	0	0	0	0
Ach_brin	1	2	0	0	0	0
Ach_foge	0	0	0	0	0	0
Ach_leon	0	0	0	0	0	0
Ach_long	0	0	0	0	0	0
Ach_luth	0	0	0	0	0	0
Ach_misc	0	0	0	0	0	0
Ach_vist	0	0	0	0	0	0
Act_nosu	0	0	0	0	0	0
Act_occr	0	0	0	0	0	0
Ami_spec	0	6	0	0	0	0
Amp_copu	0	0	0	0	0	0
Amp_fleb	0	0	0	0	0	0
Amp_hele	0	0	0	0	0	0
Amp_inar	0	0	0	0	0	0
Amp_line	0	0	0	0	0	0
Amp_micr	0	0	0	0	0	0
Amp_oval	0	0	0	0	0	0
Amp_pedi	0	0	0	0	0	0
Amp_prof	0	0	0	0	4	0
Ane_mino	0	0	0	0	0	0
Ane_tusc	0	0	0	0	0	0
Ano_spha	0	0	0	0	0	0
Asa_bahu	0	0	0	0	0	0
Ast_form	0	0	0	0	0	0
Aul_spp	0	0	0	0	0	0
Bac_paxi	0	0	0	0	2	0
Ber_fenn	0	0	0	0	0	0
Ber_ruti	48	24	27	69	69	54
Ber_scop	0	0	0	0	0	0
Bir_luce	0	0	0	0	0	0
Bra_apon	0	0	0	0	0	0
Bra_vitr	0	0	0	0	0	0
Bre_lanc	0	0	0	0	0	0
Cal_aemu	0	0	0	0	0	0

Cal_amph	0	0	0	0	0	0
Cal_baci	0	0	0	0	0	0
Cal_cras	0	0	0	0	0	0
Cal_sili	0	0	0	0	0	0
Cam_clyp	0	0	0	0	0	0
Cat_adha	0	0	0	0	0	0
Cav_lapi	0	0	0	0	0	0
Cav_pseu	0	0	0	0	0	0
Cer_clos	0	0	0	0	0	0
Chp_clam	0	0	0	0	0	0
Chp_marg	0	0	0	0	0	0
Coc_cost	0	0	0	1	0	0
Coc_neot	0	0	0	3	7	0
Coc_pedi	4	10	1	4	14	2
Coc_pelt	0	0	0	0	0	0
Coc_plac	1	5	0	1	0	4
Coc_pleu	0	0	1	0	0	0
Coc_plli	0	0	0	1	0	0
Coc_psli	0	0	0	0	0	2
Coc_psth	0	0	0	0	0	0
Coc_scut	0	0	0	0	10	0
Coc_stau	0	0	0	1	15	0
Com_pusi	0	0	0	0	0	0
Cos_aste	0	0	0	0	2	0
Cte_pulc	0	0	0	5	0	1
Cyc_atom	0	13	0	0	0	0
Cyc_choc	4	14	8	0	0	0
Cyc_mene	0	0	0	0	0	0
Cyc_radi	0	0	0	0	0	0
Cyc_ross	0	0	0	0	0	0
Cyc_stel	0	0	0	0	0	0
Cyc_stri	0	0	0	0	0	0
Cyc_trip	0	0	0	0	0	0
Cym_affi	0	0	0	0	0	0
Cym_cist	0	0	0	0	0	0
Cym_helv	0	0	0	0	0	0
Cym_lanc	0	0	0	0	0	0
Cym_prox	0	0	0	0	0	0
Cyp_sole	0	0	0	0	0	0
Cys_dubi	0	0	0	0	0	0
Den_tecr	0	0	0	0	0	0

Dia_bott	0	0	0	0	0	0
Dia_moni	27	0	4	11	25	37
Dia_prob	0	0	0	0	0	0
Dia_tenu	0	0	0	0	0	0
Dia_vulg	0	0	0	0	0	0
Dic_subi	0	0	0	0	0	0
Dip_depa	2	0	0	0	0	0
Dip_dydi	0	0	0	0	0	0
Dip_elli	0	0	0	0	0	0
Dip_inte	0	0	0	0	0	0
Dip_ocul	0	0	0	0	0	0
Dip_smdi	0	0	0	0	0	0
Dip_smpu	0	0	0	0	0	0
Dip_smrh	0	0	0	0	0	0
Dip_subo	0	0	0	0	0	0
Enc_caes	0	0	0	0	0	0
Enc_lacu	0	0	0	0	0	0
Enc_minu	0	0	0	0	0	0
Enc_obsc	0	0	0	0	0	0
Enc_pros	0	0	0	0	0	0
Enc_sile	0	0	0	0	0	0
Enc_tenu	0	0	0	0	0	0
Enp_behr	0	0	0	0	0	0
Enp_kram	0	0	0	0	0	0
Ent_palu	0	0	0	0	0	0
Ent_pseu	0	0	0	0	0	0
Ent_punc	0	0	0	0	0	0
Epi_adna	0	0	0	0	0	0
Epi_goep	0	0	0	0	0	0
Epi_sore	9	3	0	0	0	10
Epi_turg	3	0	0	0	1	0
Epi_tuwe	0	0	0	2	2	2
Euc_laev	0	0	0	0	0	0
Eun_arcu	0	0	0	0	0	0
Eun_peun	0	0	0	0	0	0
Fal_clep	0	2	0	0	0	0
Fal_cryp	0	2	0	0	0	0
Fal_flor	0	0	0	0	0	0
Fal_psny	0	0	0	0	0	0
Fal_pygm	2	0	0	0	0	0
Fis_sapr	0	0	0	0	0	0

Fra_amic	0	0	0	0	0	0
Fra_capu	0	0	0	0	0	0
Fra_caru	10	0	8	0	0	0
Fra_cava	0	0	0	0	0	0
Fra_cons	0	0	0	0	0	0
Fra_geda	0	0	0	0	0	0
Fra_grac	0	0	0	0	0	0
Fra_hydu	0	0	0	9	88	0
Fra_lept	0	0	0	0	0	0
Fra_nitz	0	0	0	0	0	0
Frp_cyli	0	0	0	0	0	0
Fru_vulg	0	1	0	0	0	0
Gom_clav	0	0	0	0	0	0
Gom_intr	0	0	0	0	0	0
Gom_oliv	0	0	0	14	19	0
Gom_parv	0	0	0	0	0	0
Gon_exig	0	0	0	6	21	16
Gos_expl	0	0	0	0	18	0
Gos_obsc	0	0	0	0	0	2
Gra_mari	0	0	0	0	0	0
Gra_ocea	1	0	0	0	0	0
Gyr_acum	0	0	0	0	0	0
Gyr_atte	0	0	0	0	0	0
Gyr_stri	0	0	0	0	0	0
Gyr_tenu	0	0	0	0	0	0
Hal_coff	2	4	0	0	0	0
Hal_exig	0	0	0	0	0	0
Hal_hols	0	0	0	0	0	0
Hal_hybr	0	0	0	0	0	0
Hal_luci	0	4	2	0	2	0
Hal_norm	0	0	0	0	0	0
Hal_subh	0	0	0	0	0	0
Hal_tene	10	0	0	0	0	0
Hal_vene	0	0	0	0	0	0
Han_balt	0	0	0	0	0	0
Has_spic	0	0	0	0	0	0
Hip_capi	0	0	0	0	0	0
Hip_hung	0	0	0	0	0	0
Hip_lesm	0	0	0	0	0	0
Kar_amoe	0	0	0	0	0	0
Kar_clev	0	0	0	0	0	0

Kar_niti	0	0	0	0	0	0
Kar_subm	8	0	0	0	0	0
Lemn_hung	0	0	0	0	0	0
Lic_debi	0	0	0	0	0	0
Lic_gran	0	0	0	0	3	1
Lic_oedi	0	0	0	0	0	0
Lic_rhom	0	0	0	0	0	0
Lun_bise	0	0	0	0	2	0
Lut_muti	0	0	0	0	0	0
Mar_atom	0	0	227	83	31	131
Mar_mart	0	0	0	0	0	0
Mar_schu	0	0	0	0	0	0
Mas_balt	0	0	0	0	0	0
Mas_brau	0	0	0	0	0	0
Mas_elli	0	2	0	0	0	0
Mas_exig	4	0	0	4	0	0
Mas_pumi	0	2	0	0	0	0
Mas_pusi	0	0	0	0	0	0
Mas_smam	0	0	0	0	0	0
Mas_smit	0	4	0	0	0	0
Mel_arct	0	0	0	0	0	0
Mel_line	16	5	7	0	0	3
Mel_moni	0	0	0	0	1	0
Mel_numm	2	0	0	2	0	0
Mel_vari	0	0	0	0	0	0
Nav_aren	0	0	0	0	0	0
Nav_bipu	0	0	0	0	0	0
Nav_boss	0	0	0	4	6	0
Nav_bott	0	0	0	12	2	0
Nav_brem	0	0	0	0	0	0
Nav_capi	0	0	0	0	0	0
Nav_cinc	0	0	0	0	0	0
Nav_clem	0	0	0	0	0	0
Nav_cryp	0	0	0	0	0	0
Nav_digi	0	2	0	0	0	0
Nav_duer	0	0	0	0	0	0
Nav_eidr	0	0	0	0	0	0
Nav_flan	0	0	0	0	0	0
Nav_geii	0	0	0	0	0	0
Nav_gepo	0	0	0	0	0	0
Nav_greg	0	0	2	0	0	0

Nav_haca	0	0	0	0	0	0
Nav_hani	0	0	0	0	0	0
Nav_hust	0	0	0	0	0	0
Nav_lanc	0	0	0	0	0	0
Nav_late	0	0	0	0	0	0
Nav_marg	0	0	0	0	0	0
Nav_meni	0	0	0	0	0	0
Nav_midi	0	0	0	0	0	0
Nav_nole	0	0	0	0	0	0
Nav_oblo	0	0	0	0	0	0
Nav_oest	0	0	0	0	0	0
Nav_pere	0	0	0	0	0	0
Nav_perm	0	4	11	107	81	111
Nav_phyl	15	7	4	1	1	2
Nav_port	0	0	0	0	0	0
Nav_radi	0	0	0	0	0	0
Nav_ramo	0	0	0	21	28	34
Nav_rato	0	0	0	0	0	0
Nav_rece	0	0	0	0	0	0
Nav_rhyn	0	6	4	0	0	1
Nav_sana	0	0	0	0	0	0
Nav_sjoe	0	0	0	0	0	2
Nav_star	0	0	0	0	0	0
Nav_subr	0	0	0	0	0	0
Nav_supr	0	0	0	0	0	0
Nav_trip	0	0	0	0	0	0
Nav_vene	0	0	0	0	0	0
Ncy_pusi	0	6	0	0	0	0
Nit_acic	0	0	0	0	0	0
Nit_anfo	0	0	0	0	0	0
Nit_anla	0	0	0	0	0	0
Nit_arch	0	0	0	0	0	0
Nit_aura	0	0	0	0	0	0
Nit_baci	0	0	0	0	0	0
Nit_dime	0	0	0	0	0	0
Nit_dipp	0	0	0	0	0	0
Nit_diss	0	0	0	0	0	0
Nit_dist	0	0	0	0	0	0
Nit_dubi	0	0	0	0	0	0
Nit_eleg	0	0	0	0	0	0
Nit_frig	0	0	0	0	0	0

Nit_frus	0	16	14	22	7	0
Nit_gros	0	0	0	0	0	0
Nit_heuf	0	0	0	0	0	0
Nit_inco	20	6	0	8	37	57
Nit_inte	0	0	0	0	0	0
Nit_lieb	0	0	0	0	0	0
Nit_lisu	0	0	0	0	0	0
Nit_lore	0	0	0	0	0	0
Nit_micr	0	0	8	4	7	4
Nit_pale	0	0	0	0	0	0
Nit_pcea	0	0	0	0	0	0
Nit_pell	0	0	0	0	0	0
Nit_perm	0	0	0	0	0	0
Nit_pusi	0	0	0	2	0	0
Nit_rect	0	0	0	0	0	0
Nit_rose	0	0	0	0	0	0
Nit_saco	0	0	0	0	0	0
Nit_sdea	0	0	0	0	0	0
Nit_sigm	0	0	0	0	0	0
Nit_soci	0	0	0	0	0	0
Nit_soli	0	0	0	0	0	0
Nit_subl	0	0	0	0	0	0
Nit_ther	0	0	0	0	0	0
Nit_umbo	0	0	0	0	0	0
Nit_vald	0	0	0	0	0	0
Nit_verm	0	0	0	0	0	0
Nit_vitr	0	0	0	0	0	0
Nit_wuel	0	0	0	0	0	0
Ope_krum	88	62	122	58	10	44
Ope_muta	13	28	64	17	4	9
Par_prot	0	0	0	0	0	0
Pau_taen	0	0	0	0	0	0
Pet_hume	0	0	0	0	0	0
Pin_appe	0	0	0	0	0	0
Pin_dimi	0	0	0	0	0	0
Pin_kroc	0	0	0	0	0	0
Pin_micr	0	0	0	0	0	0
Plc_plac	0	0	0	0	0	0
Plt_cons	0	0	0	0	0	0
Plt_deli	2	0	19	0	1	4
Plt_disp	0	0	0	0	0	0

Plt_freq	0	0	0	0	0	0
Plt_hauc	0	0	0	0	0	0
Plt_lanc	0	0	0	0	0	0
Plt_lemm	5	4	0	0	0	0
Plt_link	0	0	0	0	0	0
Plt_peri	0	0	0	0	0	0
Plt_rost	0	0	0	0	0	0
Plt_sept	21	0	0	0	0	2
Psf_tene	0	0	3	0	0	0
Pss_brev	7	0	61	0	0	0
Pss_brin	0	0	0	0	0	0
Pss_elli	175	269	255	45	34	113
Pss_perm	0	0	0	0	0	0
Pss_robu	0	0	0	0	0	0
Pss_subs	195	189	20	0	0	13
Pss_zeil	266	248	41	2	0	17
Pst_bior	0	0	0	0	0	0
Pst_oblo	0	0	0	0	0	0
Pst_punc	0	0	0	0	0	0
Pst_ross	0	0	0	0	0	0
Pst_sacc	0	0	0	0	0	0
Pte_inan	3	0	0	0	6	0
Rei_sinu	0	0	0	0	0	0
Rho_abbr	13	0	4	191	129	58
Rhp_acum	2	0	0	0	0	0
Rhp_breb	6	0	0	0	0	0
Rhp_gibb	0	0	0	0	0	0
Sel_pupu	0	0	0	0	0	0
Sel_stro	0	0	0	0	0	0
Ske_cost	0	0	0	0	0	0
Ske_subs	0	0	0	0	0	0
Sla_lapp	0	0	0	0	0	0
Std_hant	0	0	0	0	0	0
Std_parv	0	0	0	0	0	0
Stn_legu	0	0	0	0	0	0
Stn_phoe	0	2	0	0	0	0
Stn_simu	0	0	0	4	0	0
Stn_smka	0	0	0	0	0	0
Stp_anus	0	0	0	0	0	0
Stp_wisl	0	0	0	0	0	0
Sts_punc	0	47	34	0	10	41

Sts_vent	0	0	0	0	0	0
Sur_angu	0	0	0	0	0	0
Sur_brba	0	0	0	0	0	0
Sur_breb	0	0	5	0	0	0
Sur_minu	0	0	0	0	0	0
Sur_oval	0	0	0	0	0	0
Syn_acus	0	0	0	0	0	0
Syn_ulna	0	0	0	0	0	0
Tab_fasc	0	0	36	209	101	146
Tab_kten	0	0	4	0	1	0
Tab_laev	0	0	0	0	0	0
Tab_tabu	12	0	0	0	0	3
Tab_waer	0	0	4	77	197	70
Tal_fene	0	0	0	0	0	0
Tal_floc	0	0	0	0	0	0
Tet_glan	0	0	0	0	0	0
Tha_balt	0	0	0	0	0	2
Tha_hyla	0	1	0	0	0	0
Tha_leva	1	0	0	0	2	2
Tha_pros	0	0	0	0	0	0
Tha_pseu	0	0	0	0	0	0
Tha_punc	0	0	0	0	0	0
Try_angu	0	0	0	0	0	0
Try_apic	0	0	0	0	0	0
Try_ardu	0	0	0	0	0	0
Try_hung	0	0	0	0	0	0
Try_levi	0	0	0	0	0	0
Try_lite	0	0	0	0	0	0
Try_sali	0	0	0	0	0	0
Try_scal	0	0	0	0	0	0

AREA	Kvä	Kvä	Kvä	Kvä	Kvä	Kvä	Kvä	Kvä	Kvä	Kvä	Kvä
SITE	33	34	35	36	37	38	39	40	41	42	44
Acd_exil	0	0	0	0	0	0	0	0	0	0	0
Acd_minu	0	0	0	0	0	0	0	0	0	0	0
Acd_pyre	0	0	0	0	0	0	0	0	0	0	0
Ach_brev	0	1	0	0	0	0	0	0	0	0	0
Ach_brin	1	0	0	0	0	0	7	0	0	0	0
Ach_foge	0	0	0	0	0	0	0	0	0	0	0
Ach_leon	0	0	0	0	0	0	0	0	0	0	0
Ach_long	0	0	0	0	0	0	0	0	0	0	0
Ach_luth	0	0	0	0	0	0	0	0	0	0	0
Ach_misc	0	3	2	0	0	0	8	0	0	0	0
Ach_vist	8	0	0	0	0	0	4	0	2	0	0
Act_nosu	0	0	0	0	0	0	0	0	0	0	0
Act_occr	0	0	0	0	0	0	0	0	0	0	0
Ami_spec	0	0	0	0	0	0	0	0	0	0	0
Amp_copu	0	0	2	0	2	6	2	0	0	0	0
Amp_fleb	0	4	0	0	0	0	0	0	0	0	0
Amp_hele	8	0	0	0	0	12	8	0	0	0	0
Amp_inar	0	0	0	0	0	0	0	0	0	0	0
Amp_line	5	2	0	2	0	0	0	0	0	0	0
Amp_micr	0	0	0	0	0	0	0	0	0	0	0
Amp_oval	0	0	0	0	0	0	0	0	0	0	0
Amp_pedi	0	0	0	2	4	0	0	0	0	2	0
Amp_prof	0	0	0	0	0	0	0	0	0	0	0
Ane_mino	0	0	0	0	0	0	0	0	0	0	0
Ane_tusc	0	0	0	0	0	0	0	0	0	0	0
Ano_spha	0	0	0	0	0	0	0	0	0	0	0
Asa_bahu	0	0	0	0	0	0	0	0	0	0	0
Ast_form	0	0	0	0	0	0	0	0	0	0	0
Aul_spp	0	0	0	0	0	0	0	0	0	0	0
Bac_paxi	0	0	0	0	0	0	0	0	0	0	0
Ber_fenn	0	0	0	0	0	0	0	0	0	0	0
Ber_ruti	74	48	71	45	54	38	45	62	1	64	95
Ber_scop	0	0	0	0	0	0	0	0	0	0	0
Bir_luce	0	0	0	0	0	0	0	0	0	0	0
Bra_apon	0	0	0	0	0	0	0	0	0	0	0
Bra_vitr	0	0	0	0	0	0	0	0	0	0	0
Bre_lanc	0	0	2	0	0	0	0	0	0	0	0
Cal_aemu	0	0	0	0	0	0	0	0	0	0	0

Cal_amph	0	0	0	0	0	0	0	0	0	0	0
Cal_baci	0	0	0	0	0	0	0	0	0	0	0
Cal_cras	0	0	0	0	0	0	0	0	0	0	0
Cal_sili	0	0	0	0	0	0	0	0	0	0	0
Cam_clyp	0	0	0	0	0	0	0	0	0	0	0
Cat_adha	0	0	0	0	0	0	0	0	0	0	0
Cav_lapi	0	0	0	0	0	0	0	0	0	0	0
Cav_pseu	0	0	0	0	0	0	0	0	0	0	0
Cer_clos	0	0	0	0	0	0	0	0	0	0	0
Chp_clam	0	0	0	0	0	0	0	0	2	0	0
Chp_marg	0	0	0	0	0	0	0	0	0	0	0
Coc_cost	0	0	0	0	0	0	0	0	0	0	0
Coc_neot	0	0	0	0	0	0	0	0	0	0	0
Coc_pedi	0	2	2	2	6	24	18	1	1	0	0
Coc_pelt	0	0	0	0	0	0	0	0	0	0	0
Coc_plac	1	0	12	4	0	1	5	2	0	0	0
Coc_pleu	0	2	22	8	8	13	10	0	7	0	0
Coc_plli	0	0	0	0	0	0	0	3	0	0	0
Coc_psli	0	0	0	0	1	0	1	0	0	0	0
Coc_psth	0	0	0	0	0	0	0	0	0	0	0
Coc_scut	0	0	0	0	0	0	0	9	0	7	4
Coc_stau	0	0	0	0	0	2	0	0	0	0	6
Com_pusi	0	0	0	1	0	0	0	0	0	0	0
Cos_aste	0	0	0	0	0	0	0	0	0	0	0
Cte_pulc	24	27	41	71	48	28	6	18	0	0	0
Cyc_atom	0	0	0	0	0	1	0	0	0	0	0
Cyc_choc	0	0	0	0	0	0	0	0	0	0	0
Cyc_mene	0	0	0	0	0	0	0	1	0	1	0
Cyc_radi	0	0	0	0	0	0	0	0	0	0	0
Cyc_ross	0	0	0	0	0	0	0	0	0	0	0
Cyc_stel	0	0	0	0	0	0	0	0	0	0	0
Cyc_stri	0	0	0	0	0	0	0	0	0	0	0
Cyc_trip	0	0	0	0	0	0	0	0	0	0	0
Cym_affi	0	0	0	0	0	0	0	0	0	0	0
Cym_cist	0	0	0	0	0	0	0	0	0	0	0
Cym_helv	0	0	0	0	0	0	0	0	0	0	0
Cym_lanc	0	0	0	0	0	0	0	0	0	0	0
Cym_prox	0	0	0	0	0	0	0	0	0	0	0
Cyp_sole	0	0	0	0	0	0	0	0	0	0	0
Cys_dubi	0	0	0	0	0	0	0	0	0	0	0
Den_tecr	0	0	0	0	0	0	0	0	0	0	0

Dia_bott	0	0	0	0	2	2	2	0	0	3	1
Dia_moni	8	81	112	45	19	12	58	151	3	150	14
Dia_prob	0	0	0	0	0	0	0	0	0	0	0
Dia_tenu	0	0	0	0	0	0	0	0	0	0	0
Dia_vulg	0	0	4	0	0	0	0	0	0	0	0
Dic_subi	0	0	0	0	0	0	0	0	0	0	0
Dip_depa	0	0	0	0	0	0	0	0	0	0	0
Dip_dydi	0	0	0	0	0	0	0	0	0	0	0
Dip_elli	0	0	0	0	0	0	0	0	0	0	0
Dip_inte	0	0	0	0	0	0	0	0	0	0	0
Dip_ocul	0	0	0	0	0	0	0	0	0	0	0
Dip_smdi	0	0	0	0	0	0	4	0	0	0	0
Dip_smpu	0	0	0	0	0	0	0	0	0	0	0
Dip_smrh	0	0	0	0	0	0	0	0	0	0	0
Dip_subo	0	0	0	0	0	0	0	0	0	0	0
Enc_caes	0	0	0	0	0	0	0	0	0	0	0
Enc_lacu	0	0	0	0	0	0	0	0	0	0	0
Enc_minu	0	0	0	0	0	0	0	0	0	0	0
Enc_obsc	0	0	0	0	0	0	0	0	0	0	0
Enc_pros	0	0	0	0	0	0	0	0	0	0	0
Enc_sile	0	0	0	0	0	0	0	0	0	0	0
Enc_tenu	0	0	0	0	0	0	0	0	0	0	0
Enp_behr	0	0	0	0	0	0	0	0	0	0	0
Enp_kram	0	0	0	0	0	0	0	0	0	0	0
Ent_palu	0	0	0	0	0	0	0	0	0	0	0
Ent_pseu	0	0	0	0	0	0	0	0	0	0	0
Ent_punc	0	0	0	0	0	0	0	0	0	0	0
Epi_adna	0	0	2	0	2	2	2	0	2	0	0
Epi_goep	0	0	0	0	0	0	0	0	0	0	0
Epi_sore	4	2	12	0	11	12	10	0	2	6	9
Epi_turg	0	2	2	0	6	13	2	4	0	0	0
Epi_tuwe	2	10	11	4	6	39	15	1	2	4	5
Euc_laev	0	0	0	0	0	0	0	0	0	0	0
Eun_arcu	0	0	0	0	0	0	0	0	0	0	0
Eun_peun	0	0	0	0	0	0	0	0	0	0	0
Fal_clep	2	0	0	0	5	0	2	0	0	0	0
Fal_cryp	0	0	0	0	0	0	0	0	0	0	0
Fal_flor	0	0	0	0	0	0	0	0	0	0	0
Fal_psny	0	0	0	0	0	0	0	0	0	0	0
Fal_pygm	0	0	0	0	0	0	0	0	0	0	0
Fis_sapr	0	0	0	0	0	0	0	0	0	0	0

Fra_amic	0	0	0	0	0	0	0	0	0	0	0
Fra_capu	0	0	0	0	0	0	0	0	0	0	0
Fra_caru	0	0	2	0	0	0	0	0	0	0	0
Fra_cava	0	0	0	0	0	0	0	0	0	0	0
Fra_cons	0	0	0	0	0	0	0	0	0	0	0
Fra_geda	4	0	0	2	10	2	6	0	47	0	0
Fra_grac	0	0	0	0	0	0	0	0	0	0	0
Fra_hydu	2	32	0	0	0	0	1	0	0	36	0
Fra_lept	0	0	0	0	0	0	0	0	0	0	0
Fra_nitz	0	0	0	0	0	0	0	0	0	0	0
Frp_cyli	0	0	0	0	0	0	0	0	0	0	0
Fru_vulg	0	0	0	0	0	0	0	0	0	0	0
Gom_clav	0	0	0	0	0	0	0	0	0	0	0
Gom_intr	0	0	0	0	0	0	0	0	0	0	0
Gom_oliv	5	6	8	11	7	4	2	3	0	14	0
Gom_parv	0	0	0	0	0	0	0	0	0	0	0
Gon_exig	18	14	0	6	0	0	4	4	0	0	4
Gos_expl	0	0	0	0	0	0	0	0	0	0	0
Gos_obsc	0	0	0	0	0	0	0	0	0	6	4
Gra_mari	0	0	0	0	0	0	0	0	0	0	0
Gra_ocea	0	0	0	0	0	0	0	0	0	0	0
Gyr_acum	0	0	0	0	0	0	0	0	0	0	0
Gyr_atte	0	0	0	0	0	0	0	0	0	0	0
Gyr_stri	0	0	0	0	0	0	0	0	0	0	0
Gyr_tenu	0	0	0	0	0	0	0	0	0	0	0
Hal_coff	0	0	0	0	0	0	0	0	0	0	0
Hal_exig	0	0	0	0	0	0	0	0	0	0	0
Hal_hols	0	0	0	0	0	0	0	0	0	0	0
Hal_hybr	0	0	0	0	0	0	0	0	0	0	0
Hal_luci	0	0	0	0	0	2	2	0	0	0	0
Hal_norm	0	0	0	0	0	0	0	0	0	0	0
Hal_subh	0	0	0	0	0	0	0	0	0	0	0
Hal_tene	7	1	2	0	4	2	0	0	0	0	0
Hal_vene	0	4	4	0	0	0	0	0	0	0	2
Han_balt	0	0	0	0	0	0	0	0	0	0	0
Has_spic	0	0	0	0	0	0	0	0	0	0	0
Hip_capi	0	0	0	0	0	0	0	0	0	0	0
Hip_hung	0	0	0	0	0	0	0	0	0	0	0
Hip_lesm	0	0	0	0	0	0	0	0	0	0	0
Kar_amoe	0	0	0	0	0	0	0	0	0	0	0
Kar_clev	0	0	0	0	0	0	0	0	0	0	0

Kar_niti	0	0	0	0	0	0	0	0	0	0	0
Kar_subm	0	0	0	0	0	0	0	0	0	0	0
Lemn_hung	0	0	0	0	0	0	0	0	0	0	0
Lic_debi	0	0	0	0	0	0	0	0	0	0	0
Lic_gran	2	10	0	9	4	1	0	1	0	55	3
Lic_oedi	0	0	0	0	0	0	0	0	0	0	7
Lic_rhom	0	0	0	0	0	0	0	0	0	0	0
Lun_bise	0	0	0	0	0	0	0	0	0	0	0
Lut_muti	0	0	0	0	0	0	0	0	0	0	0
Mar_atom	54	23	12	18	68	26	45	36	116	0	14
Mar_mart	0	0	0	0	0	0	0	0	0	0	0
Mar_schu	0	0	2	2	0	0	0	0	0	0	0
Mas_balt	0	0	0	0	0	0	0	0	0	0	0
Mas_brau	0	0	0	0	0	0	0	0	0	0	0
Mas_elli	2	2	0	0	0	3	0	0	0	0	0
Mas_exig	0	0	0	0	0	0	0	0	0	0	0
Mas_pumi	0	0	0	0	0	17	2	0	0	0	0
Mas_pusi	0	0	0	0	0	0	0	0	0	0	0
Mas_smam	0	0	0	0	0	0	0	0	0	0	0
Mas_smit	0	3	0	0	0	0	0	0	0	0	0
Mel_arct	0	0	0	0	0	0	0	0	0	0	0
Mel_line	1	1	3	3	0	0	0	0	0	0	0
Mel_moni	0	9	1	0	17	20	32	1	4	0	0
Mel_numm	2	7	0	0	2	0	7	1	0	3	5
Mel_vari	0	0	0	0	0	0	0	0	0	0	0
Nav_aren	0	0	0	0	0	0	0	0	0	0	0
Nav_bipu	0	0	0	0	0	0	0	0	0	0	0
Nav_boss	0	0	0	0	0	0	0	0	0	0	0
Nav_bott	0	2	0	1	0	2	0	0	0	21	0
Nav_brem	0	0	0	0	0	0	0	0	0	0	0
Nav_capi	0	0	0	0	0	0	0	0	0	0	0
Nav_cinc	0	0	0	0	0	0	0	0	0	0	0
Nav_clem	0	0	2	0	0	0	0	0	0	0	0
Nav_cryp	0	0	0	8	0	0	0	0	0	0	0
Nav_digi	0	0	0	0	0	0	0	0	0	0	0
Nav_duer	0	0	0	0	0	0	0	0	0	0	0
Nav_eidr	0	0	0	0	0	0	0	0	0	0	0
Nav_flan	0	0	0	0	0	0	0	0	0	0	0
Nav_geii	0	0	0	0	0	0	0	0	0	0	0
Nav_gepo	0	0	0	0	0	0	0	0	0	0	0
Nav_greg	23	1	23	23	18	8	0	0	0	0	1

Nav_haca	0	0	0	0	0	0	0	0	0	0	0
Nav_hani	0	0	0	0	0	0	0	0	0	0	0
Nav_hust	0	0	0	0	0	0	0	0	0	0	0
Nav_lanc	0	0	15	6	0	3	0	1	0	0	0
Nav_late	0	0	0	0	0	0	0	0	0	0	0
Nav_marg	0	0	0	0	0	0	0	0	4	0	0
Nav_meni	0	0	0	0	0	0	0	0	0	0	0
Nav_midi	0	0	0	0	0	0	0	0	0	0	0
Nav_nole	0	0	0	0	0	0	0	0	0	0	0
Nav_oblo	0	0	0	0	0	0	0	0	0	0	0
Nav_oest	0	0	0	0	0	0	0	0	0	0	0
Nav_pere	0	0	0	0	0	0	0	0	0	0	0
Nav_perm	81	53	63	86	49	43	9	17	8	36	128
Nav_phyl	21	15	10	8	16	26	13	33	9	2	4
Nav_port	0	0	0	0	0	0	0	0	0	0	0
Nav_radi	0	0	0	0	0	0	0	0	0	0	0
Nav_ramo	6	2	0	2	9	4	3	2	0	10	70
Nav_rato	0	0	0	0	0	0	1	0	0	0	0
Nav_rece	0	0	0	0	0	0	0	0	0	0	0
Nav_rhyn	0	0	3	1	2	0	0	1	0	0	0
Nav_sana	0	0	0	0	0	0	2	0	0	0	0
Nav_sjoe	0	0	0	2	2	0	0	1	0	9	3
Nav_star	0	0	0	0	2	6	8	28	0	0	0
Nav_subr	0	0	0	0	0	0	0	0	0	0	0
Nav_supr	0	0	0	0	0	0	0	0	0	0	0
Nav_trip	0	1	0	0	0	0	2	0	0	0	0
Nav_vene	0	0	0	0	0	0	0	0	0	0	0
Ncy_pusi	0	0	0	0	0	0	0	0	0	0	0
Nit_acic	0	0	0	0	0	0	0	0	0	0	0
Nit_anfo	0	0	0	0	0	0	0	0	0	0	0
Nit_anla	0	0	0	0	0	0	0	0	0	0	0
Nit_arch	0	0	0	0	0	0	0	0	0	0	0
Nit_aura	0	0	0	0	0	0	0	0	0	0	0
Nit_baci	0	0	0	0	0	0	0	4	0	0	0
Nit_dime	0	0	0	0	0	0	0	0	0	0	0
Nit_dipp	0	0	0	0	0	0	0	0	0	0	0
Nit_diss	0	0	0	0	0	0	0	0	0	0	0
Nit_dist	0	0	0	0	0	0	2	0	0	0	0
Nit_dubi	0	0	0	0	0	0	0	0	0	0	0
Nit_eleg	0	2	0	0	0	0	0	0	2	0	0
Nit_frig	0	0	0	0	0	0	0	0	0	0	0

Nit_frus	37	17	13	14	31	119	145	137	26	6	28
Nit_gros	0	0	0	0	0	0	0	0	0	0	0
Nit_heuf	0	0	0	0	0	0	0	0	0	0	0
Nit_inco	230	86	142	77	61	187	31	28	42	69	139
Nit_inte	0	0	0	0	0	0	0	0	0	0	0
Nit_lieb	0	0	0	0	0	0	0	0	0	0	0
Nit_lisu	0	0	0	0	0	0	0	0	0	0	0
Nit_lore	0	0	0	0	0	0	0	0	0	0	0
Nit_micr	17	39	5	14	12	4	18	3	22	0	18
Nit_pale	0	0	0	0	0	0	0	0	0	0	0
Nit_pcea	0	0	0	0	0	0	0	0	0	0	0
Nit_pell	0	0	0	0	0	0	0	0	0	0	0
Nit_perm	0	0	0	0	0	0	0	0	0	0	0
Nit_pusi	0	0	0	0	0	0	0	0	0	0	0
Nit_rect	0	0	0	0	0	0	0	0	0	0	0
Nit_rose	0	0	0	0	0	0	0	0	0	0	0
Nit_saco	0	0	0	0	0	0	0	0	0	0	0
Nit_sdea	0	0	0	0	0	0	0	0	0	0	0
Nit_sigm	0	0	0	0	2	0	0	2	0	0	0
Nit_soci	0	0	2	2	0	0	0	0	0	0	0
Nit_soli	0	0	0	0	0	0	0	0	0	0	0
Nit_subl	0	0	0	0	0	0	0	0	0	0	0
Nit_ther	11	0	0	0	8	0	0	0	0	0	0
Nit_umbo	0	0	0	0	0	0	0	0	0	0	0
Nit_vald	0	0	0	0	0	2	0	0	0	0	0
Nit_verm	0	0	0	0	0	0	0	0	0	0	0
Nit_vitr	0	0	0	0	0	0	0	0	0	0	0
Nit_wuel	0	0	0	0	0	0	0	0	0	0	0
Ope_krum	42	100	25	56	53	32	44	14	40	16	43
Ope_muta	19	2	20	10	6	6	30	0	140	0	2
Par_prot	0	0	0	0	0	0	0	0	0	0	0
Pau_taen	0	0	0	0	0	0	0	0	0	0	0
Pet_hume	0	0	0	0	0	0	0	0	0	0	0
Pin_appe	0	0	0	0	0	0	0	0	0	0	0
Pin_dimi	0	0	0	0	0	0	0	0	0	0	0
Pin_kroc	0	0	0	0	2	0	0	0	0	0	0
Pin_micr	0	0	0	0	0	0	0	0	0	0	0
Plc_plac	0	0	0	0	0	0	0	0	0	0	0
Plt_cons	0	0	0	0	0	0	0	0	0	0	0
Plt_deli	24	10	46	16	31	16	26	4	9	0	2
Plt_disp	0	0	0	0	0	0	0	0	0	0	0

Plt_freq	0	0	0	0	0	0	0	0	0	0	0
Plt_hauc	0	0	0	0	0	1	0	0	1	0	0
Plt_lanc	0	0	0	0	0	0	0	0	0	0	0
Plt_lemm	0	0	5	2	0	0	0	0	2	0	0
Plt_link	0	0	0	0	0	0	0	0	0	0	0
Plt_peri	0	0	0	0	0	0	0	0	0	0	0
Plt_rost	0	0	0	0	0	0	0	0	0	0	0
Plt_sept	1	0	0	0	0	0	0	0	0	0	0
Psf_tene	0	0	3	0	1	0	1	0	3	0	0
Pss_brev	2	0	4	4	18	18	34	0	52	0	0
Pss_brin	0	0	0	0	0	0	0	0	0	0	0
Pss_elli	60	28	9	2	80	27	34	22	71	10	16
Pss_perm	0	0	0	0	0	0	0	0	0	0	0
Pss_robu	0	0	0	0	0	0	0	0	0	0	0
Pss_subs	0	0	1	2	10	4	0	0	93	2	4
Pss_zeil	4	6	11	3	15	22	28	4	221	0	14
Pst_bior	0	0	1	0	0	0	0	0	0	0	0
Pst_oblo	0	0	0	0	0	0	0	0	0	0	0
Pst_punc	4	0	5	0	4	0	0	1	4	0	0
Pst_ross	0	0	0	0	0	0	0	0	0	0	0
Pst_sacc	0	0	0	0	0	0	0	0	0	0	0
Pte_inan	0	0	0	0	0	0	0	0	0	0	0
Rei_sinu	0	0	0	0	0	0	0	0	0	0	0
Rho_abbr	31	50	62	89	26	88	31	135	24	153	171
Rhp_acum	0	0	0	0	0	0	0	0	0	0	0
Rhp_breb	0	0	0	0	0	2	0	0	0	0	0
Rhp_gibb	0	0	0	0	0	0	0	0	0	0	0
Sel_pupu	0	0	0	0	0	0	0	0	0	0	0
Sel_stro	0	0	0	0	0	0	0	0	0	0	0
Ske_cost	0	4	1	11	2	0	0	0	0	0	0
Ske_subs	0	0	0	0	0	0	0	0	0	0	0
Sla_lapp	0	0	0	0	0	0	0	0	0	0	0
Std_hant	0	0	0	0	0	0	0	0	0	0	0
Std_parv	0	0	0	0	0	0	0	0	0	0	0
Stn_legu	0	0	0	0	0	0	0	0	0	0	0
Stn_phoe	0	0	0	0	0	0	0	0	0	0	0
Stn_simu	0	0	0	0	0	0	0	0	0	1	0
Stn_smka	0	0	0	0	0	0	0	0	0	0	0
Stp_anus	0	0	0	0	0	0	0	0	4	0	0
Stp_wisl	0	0	0	0	0	0	0	0	0	0	0
Sts_punc	10	8	0	0	11	0	11	8	32	0	11

Sts_vent	0	0	0	0	0	0	0	2	0	0	0
Sur_angu	0	0	0	0	0	0	0	0	0	0	0
Sur_brba	0	0	0	0	0	0	2	0	0	0	0
Sur_breb	2	0	0	0	0	0	0	0	0	0	0
Sur_minu	0	0	0	0	0	0	0	0	0	0	0
Sur_oval	0	0	0	0	0	0	0	0	0	0	0
Syn_acus	0	0	0	0	0	0	0	0	0	0	0
Syn_ulna	0	0	0	0	0	0	0	0	0	0	0
Tab_fasc	116	192	173	298	189	88	205	228	2	186	75
Tab_kten	0	0	0	0	0	0	0	0	0	0	0
Tab_laev	0	0	0	0	0	0	2	2	0	0	0
Tab_tabu	0	0	1	1	0	2	1	3	0	1	0
Tab_waer	25	85	24	25	52	0	7	20	0	127	98
Tal_fene	0	0	0	0	0	0	0	0	0	0	0
Tal_floc	0	0	0	0	0	0	0	0	0	0	0
Tet_glan	0	0	0	0	0	0	0	0	0	0	0
Tha_balt	0	0	0	0	0	0	0	0	0	0	0
Tha_hyla	0	0	0	0	0	0	0	0	0	0	0
Tha_leva	0	0	0	0	0	0	0	0	0	0	0
Tha_pros	0	1	0	0	2	0	0	0	0	0	0
Tha_pseu	0	0	0	0	0	0	0	0	0	0	0
Tha_punc	0	0	0	0	0	0	0	0	0	0	0
Try_angu	0	0	0	0	0	0	0	0	0	0	0
Try_apic	0	0	0	2	0	2	0	2	0	0	0
Try_ardu	0	0	0	0	0	6	0	0	0	0	0
Try_hung	0	0	0	0	0	0	0	0	0	0	0
Try_levi	0	0	0	0	0	0	0	0	0	0	0
Try_lite	0	0	0	0	0	0	0	0	0	0	0
Try_sali	0	0	0	0	0	0	0	0	0	0	0
Try_scal	0	0	0	0	0	0	0	0	0	0	0

AREA	Him	Him	Him	Him	Him	Him	Him	Him	Him	Him	Him	Him
SITE	45	46	47	48	49	50	51	52	53	55	56	57
Acd_exil	0	0	0	0	0	0	0	0	0	0	0	0
Acd_minu	0	0	0	0	0	0	0	0	0	0	0	0
Acd_pyre	0	0	0	0	0	0	0	0	0	0	0	0
Ach_brev	0	0	0	0	0	0	0	0	0	0	0	0
Ach_brin	0	0	0	0	0	0	0	0	0	0	0	0
Ach_foge	0	0	0	0	0	0	0	0	0	0	0	0
Ach_leon	21	0	0	0	0	0	0	0	0	0	0	0
Ach_long	0	0	0	0	0	0	0	0	0	0	0	0
Ach_luth	0	0	0	0	0	0	0	0	0	0	0	0
Ach_misc	7	0	0	0	0	0	0	19	1	0	0	0
Ach_vist	0	0	0	0	0	5	0	0	21	0	0	0
Act_nosu	0	0	0	0	0	0	0	0	0	0	0	0
Act_occr	0	0	0	0	0	0	0	0	0	0	0	0
Ami_spec	0	0	0	0	0	0	0	0	0	0	0	0
Amp_copu	0	2	0	6	2	6	4	0	0	0	0	0
Amp_fleb	0	0	0	0	0	0	0	0	4	0	0	0
Amp_hele	0	0	0	0	0	0	0	0	0	23	0	0
Amp_inar	0	0	0	19	0	14	12	5	0	0	0	0
Amp_line	6	2	0	0	0	0	0	0	3	0	0	0
Amp_micr	0	0	0	0	0	0	0	2	6	0	0	0
Amp_oval	0	0	0	0	0	2	0	0	0	0	0	0
Amp_pedi	6	0	5	4	24	42	33	5	6	8	0	3
Amp_prof	0	0	0	0	2	0	0	0	0	0	0	0
Ane_mino	0	0	0	0	0	0	0	0	0	0	0	0
Ane_tusc	0	0	0	0	0	0	0	0	0	0	0	0
Ano_spha	0	0	0	0	0	0	0	0	0	0	0	0
Asa_bahu	0	0	0	0	0	0	0	7	0	3	0	0
Ast_form	0	0	0	0	0	0	0	0	0	0	0	0
Aul_spp	0	0	0	0	0	0	0	0	0	0	0	0
Bac_paxi	0	0	0	0	0	0	0	0	0	0	0	0
Ber_fenn	0	0	0	0	0	0	0	0	0	0	0	0
Ber_ruti	141	40	81	13	2	4	25	12	38	1	46	20
Ber_scop	0	0	0	0	0	0	0	0	0	0	0	0
Bir_luce	0	0	2	0	2	0	0	0	0	0	0	0
Bra_apon	0	0	0	0	0	0	0	0	0	0	0	0
Bra_vitr	0	0	0	0	0	0	0	0	0	0	0	0
Bre_lanc	0	0	0	0	0	0	0	0	0	0	0	0
Cal_aemu	0	0	0	0	0	0	0	0	0	0	0	0

Cal_amph	0	0	0	0	0	0	0	0	0	0	0	0
Cal_baci	0	0	0	0	0	0	0	0	0	0	0	0
Cal_cras	0	0	0	1	2	0	0	0	0	0	0	0
Cal_sili	0	0	0	0	0	0	0	0	0	0	0	0
Cam_clyp	0	0	0	0	0	0	0	0	0	0	0	0
Cat_adha	0	0	0	0	5	2	0	0	0	0	0	0
Cav_lapi	0	0	0	0	0	0	0	0	0	0	0	0
Cav_pseu	0	0	0	0	0	0	0	0	0	0	0	0
Cer_clos	6	0	0	0	0	0	0	0	5	0	0	0
Chp_clam	0	0	0	0	0	0	0	0	0	0	0	0
Chp_marg	0	0	0	0	0	0	0	0	0	0	0	0
Coc_cost	0	0	0	0	0	0	0	0	0	0	0	0
Coc_neot	0	0	0	3	8	9	0	0	0	0	0	1
Coc_pedi	0	7	2	1	0	2	5	2	1	17	0	0
Coc_pelt	2	0	0	2	0	0	0	0	0	0	0	0
Coc_plac	1	0	0	6	2	26	4	4	0	7	6	12
Coc_pleu	4	1	0	7	1	18	16	5	1	10	2	5
Coc_plli	0	0	0	0	0	0	0	0	0	0	0	0
Coc_psli	0	0	0	0	0	0	0	0	0	0	0	0
Coc_psth	0	0	0	0	0	0	0	1	0	0	0	0
Coc_scut	0	0	1	0	0	6	1	0	0	64	3	1
Coc_stau	0	0	0	0	0	0	0	0	0	0	0	0
Com_pusi	0	0	0	0	0	0	0	0	0	0	0	0
Cos_aste	0	0	0	0	0	0	0	0	0	0	0	0
Cte_pulc	11	15	24	10	27	6	6	6	9	0	6	3
Cyc_atom	0	0	0	0	0	2	0	2	0	0	0	0
Cyc_choc	0	1	1	1	0	7	1	3	0	0	2	0
Cyc_mene	0	0	2	0	0	0	0	0	0	0	0	0
Cyc_radi	1	1	0	0	0	0	0	0	0	0	0	0
Cyc_ross	0	0	0	0	0	0	0	0	0	0	0	0
Cyc_stel	0	0	0	0	0	0	0	0	0	0	0	0
Cyc_stri	0	0	0	0	0	0	0	0	0	2	0	0
Cyc_trip	0	0	0	0	0	0	0	0	0	7	0	0
Cym_affi	0	0	0	0	0	0	0	0	0	0	0	0
Cym_cist	0	0	0	0	0	0	0	0	0	0	0	0
Cym_helv	0	0	0	0	0	0	0	0	0	0	0	0
Cym_lanc	0	0	0	0	0	0	0	0	0	0	0	0
Cym_prox	0	0	0	0	0	0	0	0	0	0	0	0
Cyp_sole	0	0	0	0	0	0	0	0	0	0	0	0
Cys_dubi	0	0	0	0	0	0	0	2	0	0	0	0
Den_tecr	0	0	0	0	0	0	0	0	0	0	0	0

Dia_bott	0	0	0	0	0	0	0	0	0	0	0	0
Dia_moni	98	62	32	15	81	27	19	36	97	10	23	47
Dia_prob	0	0	0	0	0	0	0	0	0	0	0	0
Dia_tenu	0	0	0	0	0	0	0	0	0	0	0	0
Dia_vulg	0	2	0	2	0	0	0	2	0	0	0	0
Dic_subi	0	0	0	0	0	0	0	2	0	0	0	0
Dip_depa	0	0	0	0	0	0	0	0	0	0	0	0
Dip_dydi	0	0	0	0	4	0	0	0	0	0	0	0
Dip_elli	0	0	0	0	0	0	0	0	0	0	0	0
Dip_inte	0	0	0	0	0	0	0	0	0	0	0	0
Dip_ocul	0	0	0	0	0	0	0	0	0	0	0	0
Dip_smdi	0	0	2	0	0	0	0	0	0	0	0	0
Dip_smpu	0	0	0	0	0	0	0	0	0	0	0	0
Dip_smrh	0	0	0	0	0	2	0	0	0	0	0	0
Dip_subo	0	0	0	0	0	0	0	0	0	0	0	0
Enc_caes	0	0	0	0	0	0	0	0	0	0	0	0
Enc_lacu	0	0	0	0	0	0	0	0	0	0	0	0
Enc_minu	0	0	0	0	0	0	0	0	0	0	0	0
Enc_obsc	0	0	0	0	0	0	0	0	0	0	0	0
Enc_pros	0	0	0	0	0	0	0	0	0	0	0	0
Enc_sile	0	0	0	0	0	0	0	0	0	0	0	0
Enc_tenu	0	0	0	0	0	0	0	0	0	0	0	0
Enp_behr	0	0	0	0	0	0	0	0	0	0	0	0
Enp_kram	0	0	0	0	0	0	0	0	0	0	0	0
Ent_palu	0	0	0	0	0	0	0	0	0	0	0	0
Ent_pseu	2	0	0	0	0	0	0	0	0	0	0	0
Ent_punc	0	0	0	0	0	0	0	0	0	0	0	0
Epi_adna	0	0	0	0	0	2	0	2	0	0	2	2
Epi_goep	0	0	0	0	0	0	0	0	0	0	0	0
Epi_sore	0	0	4	2	0	0	0	0	0	0	1	2
Epi_turg	0	0	0	0	0	0	0	0	0	0	0	0
Epi_tuwe	2	0	0	0	0	0	0	0	0	0	2	2
Euc_laev	0	0	0	0	0	0	0	0	0	0	0	0
Eun_arcu	0	0	0	0	0	0	0	0	0	0	0	0
Eun_peun	0	0	0	0	0	0	0	0	0	0	0	0
Fal_clep	0	9	0	10	7	0	9	8	0	8	1	0
Fal_cryp	0	0	0	0	0	0	0	0	0	0	0	0
Fal_flor	0	0	0	6	2	2	0	0	0	0	0	0
Fal_psny	0	0	0	0	0	0	0	1	0	0	0	0
Fal_pygm	0	0	0	0	0	0	0	0	0	0	0	0
Fis_sapr	0	0	0	0	0	0	0	0	0	0	0	0

Fra_amic	0	0	0	0	0	0	0	0	0	0	0	0
Fra_capu	0	0	0	0	0	0	0	0	0	0	0	0
Fra_caru	0	0	0	0	0	0	0	1	10	0	0	0
Fra_cava	0	0	0	0	0	0	0	0	0	0	0	0
Fra_cons	0	0	0	0	0	0	0	0	0	0	0	0
Fra_geda	1	0	0	0	0	2	0	4	0	0	0	3
Fra_grac	0	0	0	0	0	0	0	0	0	0	0	0
Fra_hydu	0	1	0	0	0	0	0	0	0	0	0	0
Fra_lept	0	0	0	0	0	0	0	0	0	0	0	0
Fra_nitz	0	0	0	0	0	0	0	0	0	0	0	0
Frp_cyli	0	0	0	0	0	0	0	0	0	0	0	0
Fru_vulg	0	0	0	0	0	0	0	0	0	0	0	0
Gom_clav	0	0	0	0	0	0	0	0	0	0	0	0
Gom_intr	0	0	0	0	0	0	0	0	0	0	0	0
Gom_oliv	2	3	3	2	9	11	3	0	1	3	6	3
Gom_parv	0	0	0	0	0	0	0	0	0	0	0	0
Gon_exig	0	22	2	0	5	3	7	4	0	0	20	6
Gos_expl	0	0	0	0	0	0	0	0	0	0	0	0
Gos_obsc	0	0	0	0	0	6	0	2	1	16	2	0
Gra_mari	0	0	0	0	0	2	0	0	0	0	0	0
Gra_ocea	0	0	0	0	0	0	0	0	0	0	0	0
Gyr_acum	0	6	2	0	0	0	0	0	4	0	0	0
Gyr_atte	0	0	0	0	0	0	0	0	0	0	0	0
Gyr_stri	0	0	0	0	0	0	0	0	0	0	0	0
Gyr_tenu	0	0	0	0	0	0	0	0	0	0	0	0
Hal_coff	0	0	0	0	2	0	7	2	0	4	0	0
Hal_exig	0	0	0	3	0	0	0	0	0	0	0	0
Hal_hols	0	0	0	0	0	0	0	2	0	0	0	0
Hal_hybr	0	0	0	0	0	0	0	0	0	0	0	0
Hal_luci	0	0	14	0	0	1	0	6	7	4	0	0
Hal_norm	0	0	0	0	0	0	0	0	0	0	0	0
Hal_subh	0	0	0	0	0	0	0	0	0	2	0	0
Hal_tene	6	2	21	0	4	0	0	0	0	2	0	0
Hal_vene	0	0	3	0	3	2	0	2	0	0	0	0
Han_balt	0	0	0	0	0	0	0	0	0	0	0	0
Has_spic	0	0	0	0	0	0	0	0	0	0	0	0
Hip_capi	0	0	0	0	0	0	0	0	0	0	0	0
Hip_hung	0	0	0	0	0	0	0	0	0	0	0	0
Hip_lesm	0	0	0	0	0	0	0	0	0	0	0	0
Kar_amoe	1	0	0	0	0	0	0	0	0	0	0	0
Kar_clev	0	0	0	0	0	0	0	0	0	0	0	0

Kar_niti	0	0	0	0	0	0	0	0	0	0	0	0
Kar_subm	0	0	0	0	0	0	0	0	0	0	0	0
Lemn_hung	0	0	0	0	0	0	0	0	0	0	0	0
Lic_debi	0	2	0	0	0	0	0	0	0	0	0	0
Lic_gran	0	0	0	2	3	9	29	0	0	2	0	21
Lic_oedi	0	0	2	0	0	0	0	0	0	0	0	0
Lic_rhom	0	2	0	0	0	0	0	0	0	0	0	0
Lun_bise	4	12	2	0	0	0	0	0	6	8	2	2
Lut_muti	0	0	0	0	0	2	0	0	0	0	0	0
Mar_atom	9	0	0	0	0	0	0	15	10	6	0	35
Mar_mart	0	0	0	0	0	5	0	0	0	0	0	0
Mar_schu	0	2	0	22	8	0	0	19	0	0	0	0
Mas_balt	0	0	0	0	0	0	0	0	0	0	0	0
Mas_brau	0	0	0	0	0	0	0	0	0	0	0	0
Mas_elli	0	0	0	0	0	0	0	0	0	0	0	0
Mas_exig	0	0	0	0	0	0	0	0	0	0	0	0
Mas_pumi	0	0	2	0	0	0	0	2	0	0	2	0
Mas_pusi	0	0	0	0	0	0	0	0	0	0	0	0
Mas_smam	0	0	0	0	0	0	0	0	0	0	0	0
Mas_smit	0	0	0	0	0	0	0	0	2	0	0	0
Mel_arct	0	0	0	0	0	0	0	0	0	0	0	0
Mel_line	1	0	0	0	0	0	0	0	0	0	0	0
Mel_moni	2	1	0	0	0	0	0	0	1	3	0	2
Mel_numm	2	0	0	0	0	0	2	0	0	0	0	0
Mel_vari	0	0	0	0	0	0	0	0	0	0	0	0
Nav_aren	0	2	0	0	0	0	0	0	0	0	0	0
Nav_bipu	0	0	0	2	0	0	0	0	0	0	0	0
Nav_boss	0	0	15	0	2	2	0	0	0	0	0	0
Nav_bott	0	0	0	3	13	0	0	0	0	2	2	0
Nav_brem	0	0	0	0	0	0	0	0	0	0	0	0
Nav_capi	0	0	0	0	0	0	0	0	0	0	0	0
Nav_cinc	0	0	0	0	0	1	0	0	0	0	0	0
Nav_clem	0	2	0	0	0	2	0	0	0	0	0	0
Nav_cryp	12	38	0	0	0	0	0	14	32	1	0	26
Nav_digi	0	0	0	0	0	0	0	0	0	0	0	0
Nav_duer	0	0	0	0	0	0	0	0	1	0	0	0
Nav_eidr	0	0	0	0	0	0	0	0	0	0	0	0
Nav_flan	3	0	0	0	0	2	1	1	0	0	0	1
Nav_geii	0	0	0	2	16	0	0	0	0	0	0	0
Nav_gepo	0	0	0	0	0	0	0	0	0	0	0	0
Nav_greg	7	0	42	14	15	21	26	1	10	7	15	0

Nav_haca	0	0	0	2	0	0	0	0	0	0	0	0
Nav_hani	0	0	0	0	0	0	0	0	0	0	0	0
Nav_hust	0	0	0	0	0	1	0	0	0	0	0	0
Nav_lanc	22	24	32	17	4	15	26	12	12	2	0	4
Nav_late	0	0	0	0	0	0	0	0	0	0	0	0
Nav_marg	2	2	0	10	10	0	0	9	0	0	0	0
Nav_meni	0	0	0	0	2	0	0	0	0	0	0	0
Nav_midi	0	1	0	0	0	0	0	0	0	0	0	0
Nav_nole	0	0	0	0	0	0	0	0	0	0	0	0
Nav_oblo	0	0	0	0	0	0	0	0	0	0	0	0
Nav_oest	0	0	0	0	0	0	0	0	0	0	0	0
Nav_pere	0	0	0	0	0	0	0	0	0	0	0	0
Nav_perm	183	117	139	77	25	99	77	98	128	123	116	108
Nav_phyl	5	7	0	6	4	8	8	28	17	18	3	22
Nav_port	0	0	0	0	0	0	0	0	0	0	0	0
Nav_radi	0	0	0	0	0	0	0	0	0	0	0	0
Nav_ramo	0	0	2	1	0	9	8	3	1	24	12	11
Nav_rato	0	0	0	0	0	0	0	0	0	0	0	0
Nav_rece	0	0	0	0	0	0	0	0	0	0	0	0
Nav_rhyn	7	0	0	0	0	0	0	0	2	0	0	0
Nav_sana	0	0	0	0	0	0	0	0	0	0	0	0
Nav_sjoe	18	10	5	30	17	2	6	40	16	17	51	8
Nav_star	0	0	18	0	0	0	0	0	0	0	0	0
Nav_subr	0	0	0	0	0	0	0	0	0	0	0	0
Nav_supr	0	0	0	0	0	0	0	0	0	0	0	0
Nav_trip	0	2	2	0	0	0	2	0	0	8	0	0
Nav_vene	0	0	0	0	0	0	0	0	0	0	0	0
Ncy_pusi	0	0	0	0	0	0	0	0	0	0	0	0
Nit_acic	0	0	0	0	0	0	0	0	0	0	0	0
Nit_anfo	0	0	0	0	0	0	0	0	0	0	0	0
Nit_anla	0	0	0	0	0	0	0	0	0	0	0	0
Nit_arch	0	0	0	0	0	0	0	0	0	0	0	0
Nit_aura	0	0	0	0	0	0	0	0	0	0	0	0
Nit_baci	0	0	0	0	0	0	0	0	0	0	0	0
Nit_dime	0	0	0	0	0	0	0	0	0	0	0	0
Nit_dipp	0	0	0	0	0	0	0	0	0	0	0	0
Nit_diss	0	0	0	0	0	0	0	0	0	2	0	0
Nit_dist	0	0	0	0	0	0	0	0	0	2	0	0
Nit_dubi	0	0	0	0	0	0	0	0	0	0	0	0
Nit_eleg	0	0	0	0	0	0	0	0	0	0	0	0
Nit_frig	0	0	6	0	0	0	0	0	0	0	0	0

Nit_frus	9	66	43	6	10	31	79	11	20	11	37	15
Nit_gros	0	0	0	0	0	0	0	0	0	0	0	0
Nit_heuf	0	0	0	0	0	0	0	0	0	0	0	0
Nit_inco	70	235	199	380	298	155	266	356	259	240	210	102
Nit_inte	0	0	0	0	0	0	0	0	0	0	0	0
Nit_lieb	0	0	0	0	0	0	0	0	0	0	0	0
Nit_lisu	0	0	0	0	0	0	0	0	0	0	0	0
Nit_lore	0	0	0	0	0	0	0	0	0	0	0	0
Nit_micr	4	8	20	0	0	0	0	3	35	7	9	8
Nit_pale	0	0	0	0	0	0	0	0	0	0	0	0
Nit_pcea	0	0	0	0	0	0	0	0	0	0	0	0
Nit_pell	0	0	0	0	0	0	0	0	0	0	0	0
Nit_perm	0	0	0	0	0	0	0	0	0	0	0	0
Nit_pusi	0	0	0	0	0	0	0	0	0	0	0	0
Nit_rect	0	0	0	0	0	0	0	0	0	0	0	0
Nit_rose	0	0	0	0	0	0	0	0	0	0	0	0
Nit_saco	0	0	0	0	0	0	0	0	0	0	0	0
Nit_sdea	0	0	0	0	0	0	0	0	0	0	0	0
Nit_sigm	4	0	3	0	0	0	0	0	0	0	1	2
Nit_soci	4	0	0	0	0	0	0	2	0	0	0	0
Nit_soli	0	0	0	0	0	0	0	0	0	0	0	0
Nit_subl	0	0	0	0	0	0	0	0	0	0	0	0
Nit_ther	35	9	0	0	0	0	0	0	0	0	0	0
Nit_umbo	0	0	0	0	0	0	0	0	0	0	0	0
Nit_vald	0	16	0	0	0	4	2	0	7	41	0	0
Nit_verm	0	0	0	0	0	0	0	0	0	0	0	0
Nit_vitr	0	0	0	0	0	0	0	0	0	0	0	0
Nit_wuel	0	0	0	0	0	0	0	0	0	0	0	0
Ope_krum	0	0	0	0	0	2	2	10	12	8	6	112
Ope_muta	0	0	0	82	44	25	0	18	0	0	2	2
Par_prot	2	0	0	0	0	0	0	0	0	0	0	0
Pau_taen	0	0	0	2	0	0	0	0	0	0	0	0
Pet_hume	0	0	0	0	0	0	0	0	0	0	0	0
Pin_appe	2	0	0	0	0	0	0	0	0	0	0	0
Pin_dimi	0	0	0	0	0	0	0	0	0	0	0	0
Pin_kroc	0	0	0	0	0	2	0	0	0	0	0	0
Pin_micr	0	0	0	0	0	0	0	0	0	0	0	0
Plc_plac	0	0	0	0	0	0	0	0	0	0	0	0
Plt_cons	0	0	0	0	0	0	0	0	0	0	0	0
Plt_deli	22	33	22	52	30	54	43	52	9	44	8	25
Plt_disp	0	0	0	0	0	0	0	0	1	0	1	4

Plt_freq	0	0	0	0	0	0	0	0	0	0	0	0
Plt_hauc	10	1	4	1	16	13	0	4	0	0	0	1
Plt_lanc	0	0	0	0	0	0	0	0	0	0	0	0
Plt_lemm	0	1	0	9	13	2	0	2	0	1	0	5
Plt_link	4	0	0	4	0	0	0	1	0	3	2	2
Plt_peri	0	0	0	0	0	0	0	0	0	0	0	0
Plt_rost	0	0	0	0	0	0	0	0	0	0	0	0
Plt_sept	0	0	0	0	0	0	0	0	0	0	0	0
Psf_tene	0	0	0	0	0	2	0	1	0	0	0	0
Pss_brev	0	0	0	0	0	0	0	2	0	4	0	10
Pss_brin	0	0	0	0	0	0	0	0	0	0	0	0
Pss_elli	0	0	0	0	0	0	0	0	0	0	0	10
Pss_perm	0	0	0	0	0	0	0	0	0	0	0	0
Pss_robu	0	0	0	0	0	0	0	0	0	0	0	0
Pss_subs	0	0	0	0	0	9	0	0	0	0	0	0
Pss_zeil	0	0	0	0	0	5	0	2	0	0	0	3
Pst_bior	0	0	0	0	0	0	0	0	0	0	0	0
Pst_oblo	0	0	0	0	0	0	0	0	0	0	0	0
Pst_punc	14	8	8	39	10	5	9	3	1	1	2	3
Pst_ross	0	0	0	0	0	0	0	0	0	0	0	0
Pst_sacc	0	0	0	0	0	0	0	0	0	0	0	0
Pte_inan	0	0	0	0	0	0	0	0	0	0	0	0
Rei_sinu	0	0	0	0	0	0	0	0	0	0	0	0
Rho_abbr	55	83	42	67	65	72	54	24	27	96	102	22
Rhp_acum	0	0	0	0	0	0	0	0	0	0	0	0
Rhp_breb	0	0	0	0	0	0	0	0	0	0	0	0
Rhp_gibb	0	0	0	0	0	0	0	0	0	0	0	0
Sel_pupu	0	0	0	0	0	0	0	0	0	0	0	0
Sel_stro	0	0	0	0	0	0	0	0	0	0	0	0
Ske_cost	0	0	0	0	0	0	0	0	0	0	0	0
Ske_subs	0	0	0	0	0	0	0	0	0	0	1	0
Sla_lapp	0	0	0	0	0	0	0	0	0	0	0	0
Std_hant	0	0	0	0	0	0	0	0	0	0	0	0
Std_parv	4	1	0	0	0	0	0	0	0	0	0	0
Stn_legu	0	0	0	0	0	0	0	0	0	0	0	0
Stn_phoe	0	0	0	0	0	0	0	0	0	0	0	0
Stn_simu	4	0	13	0	0	0	4	0	2	0	6	0
Stn_smka	0	0	0	0	0	0	0	0	0	0	0	0
Stp_anus	6	0	0	0	0	0	0	0	4	0	0	0
Stp_wisl	0	0	0	0	0	0	0	0	0	0	0	0
Sts_punc	0	0	0	0	0	3	4	8	8	2	0	54

Sts_vent	0	0	0	0	0	0	0	0	0	0	0	0
Sur_angu	0	0	0	0	0	0	0	0	0	0	0	0
Sur_brba	0	0	0	0	0	0	0	0	0	0	0	0
Sur_breb	20	0	0	0	0	1	0	2	5	0	0	8
Sur_minu	0	0	0	0	0	0	0	0	0	0	0	0
Sur_oval	5	0	0	0	0	0	0	0	0	0	0	0
Syn_acus	0	0	0	0	0	0	0	0	0	0	0	0
Syn_ulna	0	0	0	0	0	0	0	0	0	0	0	0
Tab_fasc	109	137	157	53	164	215	197	102	147	99	262	231
Tab_kten	0	0	0	0	0	0	0	0	0	0	0	0
Tab_laev	0	0	0	0	0	0	0	0	0	0	0	0
Tab_tabu	0	0	0	0	0	0	0	0	2	0	0	0
Tab_waer	12	2	11	4	36	1	0	4	6	20	26	29
Tal_fene	0	0	0	0	0	0	0	0	0	0	0	0
Tal_floc	0	0	0	0	0	0	0	0	0	0	0	0
Tet_glan	0	0	0	0	0	0	0	0	0	0	0	0
Tha_balt	0	0	0	0	0	0	0	0	0	0	0	0
Tha_hyla	0	0	0	0	0	1	0	0	0	0	0	0
Tha_leva	1	0	10	0	1	9	1	0	2	2	0	0
Tha_pros	0	0	0	0	0	0	0	0	0	0	0	0
Tha_pseu	0	0	0	0	0	0	0	0	0	0	0	0
Tha_punc	0	0	0	0	0	0	0	0	0	0	0	0
Try_angu	0	0	0	0	0	0	0	0	0	0	0	0
Try_apic	7	0	0	0	0	0	0	2	0	5	0	2
Try_ardu	0	0	0	0	0	0	0	0	0	0	0	0
Try_hung	0	0	0	0	0	0	0	0	0	0	0	0
Try_levi	0	0	0	0	0	0	0	0	0	0	0	0
Try_lite	0	0	0	0	0	0	0	0	0	0	0	0
Try_sali	2	0	0	0	0	0	2	0	0	0	0	0
Try_scal	0	0	0	0	0	0	0	0	0	0	0	0

AREA	Grä	Grä	Grä	Grä	Grä	Grä	Grä	Grä	Grä	Grä
SITE	59	60	61	62	63	64	65	66	67	68
Acd_exil	0	0	0	0	0	0	0	0	0	0
Acd_minu	10	23	2	1	3	2	4	0	5	5
Acd_pyre	0	0	0	0	0	0	0	0	0	0
Ach_brev	0	0	0	0	0	0	0	0	0	0
Ach_brin	0	0	0	0	0	0	0	0	0	0
Ach_foge	0	0	0	0	0	0	3	0	0	1
Ach_leon	0	0	0	0	0	0	0	0	0	0
Ach_long	0	0	0	0	0	0	0	0	0	0
Ach_luth	0	0	0	0	0	0	0	0	0	0
Ach_misc	0	0	0	1	0	2	0	1	0	0
Ach_vist	0	0	0	0	0	0	0	0	0	0
Act_nosu	0	0	0	0	0	0	0	0	0	0
Act_occr	0	0	0	0	0	0	0	0	0	0
Ami_spec	0	0	0	0	0	0	0	0	0	0
Amp_copu	0	1	0	3	0	0	2	0	3	1
Amp_fleb	6	10	1	3	2	0	2	0	1	1
Amp_hele	0	0	0	0	0	0	0	0	0	0
Amp_inar	0	0	0	0	0	0	0	0	0	0
Amp_line	1	3	0	1	0	0	0	0	0	0
Amp_micr	0	0	0	0	0	0	0	0	0	0
Amp_oval	0	0	0	0	0	0	0	0	0	0
Amp_pedi	15	19	16	12	1	10	26	42	3	20
Amp_prof	0	0	0	0	0	0	0	0	0	0
Ane_mino	0	0	0	1	1	0	0	0	0	0
Ane_tusc	0	0	0	0	0	0	0	0	0	0
Ano_spha	0	0	0	0	0	0	0	0	0	0
Asa_bahu	0	0	0	0	0	0	0	0	0	0
Ast_form	0	0	0	0	0	0	0	0	0	0
Aul_spp	0	0	0	0	0	0	0	0	0	0
Bac_paxi	2	0	0	0	0	0	0	0	0	0
Ber_fenn	0	33	33	9	6	3	1	12	11	26
Ber_ruti	58	24	4	3	7	6	1	13	186	0
Ber_scop	0	0	0	0	0	0	0	0	0	0
Bir_luce	0	0	0	0	0	0	0	0	0	0
Bra_apon	0	0	0	0	0	0	0	0	0	0
Bra_vitr	0	0	0	0	0	0	0	0	0	0
Bre_lanc	0	1	0	1	0	0	0	0	0	0
Cal_aemu	0	0	0	0	0	0	0	0	0	0

Cal_amph	0	0	0	0	0	0	0	0	0	0
Cal_baci	0	0	0	0	0	0	0	3	0	2
Cal_cras	0	0	0	0	0	0	0	0	0	0
Cal_sili	0	0	0	0	0	0	0	0	0	0
Cam_clyp	0	0	0	0	0	0	0	0	0	0
Cat_adha	3	0	0	0	0	0	0	0	0	0
Cav_lapi	0	0	0	0	0	0	0	0	0	0
Cav_pseu	0	0	0	0	0	0	0	0	0	0
Cer_clos	0	0	0	0	0	0	0	0	0	0
Chp_clam	0	0	0	0	0	0	0	0	0	0
Chp_marg	0	0	0	0	0	0	0	2	0	2
Coc_cost	0	0	0	0	0	0	0	0	0	0
Coc_neot	4	6	9	1	4	4	31	4	0	5
Coc_pedi	2	1	24	4	7	0	5	4	0	33
Coc_pelt	0	0	0	0	0	0	0	0	0	0
Coc_plac	2	3	1	2	4	4	11	3	1	11
Coc_pleu	0	0	0	0	0	0	0	0	0	0
Coc_plli	0	0	0	0	0	0	0	0	0	0
Coc_psli	0	0	0	0	0	0	0	0	0	0
Coc_psth	0	0	0	0	0	0	0	0	0	0
Coc_scut	0	0	0	0	0	0	0	0	0	2
Coc_stau	0	0	0	0	0	0	0	0	0	0
Com_pusi	0	0	0	0	0	0	0	0	0	0
Cos_aste	0	0	0	0	0	0	0	0	0	0
Cte_pulc	1	8	19	4	24	26	20	8	3	8
Cyc_atom	0	0	0	0	0	0	0	0	0	0
Cyc_choc	0	0	0	0	0	0	0	0	0	0
Cyc_mene	0	0	0	0	0	0	0	0	0	0
Cyc_radi	0	0	0	0	0	0	0	0	0	0
Cyc_ross	0	0	0	0	0	0	0	0	0	0
Cyc_stel	0	0	0	0	0	0	0	0	0	0
Cyc_stri	0	0	0	0	0	0	0	0	0	0
Cyc_trip	0	0	0	0	0	0	0	0	0	0
Cym_affi	0	0	0	0	0	0	0	0	0	0
Cym_cist	0	0	0	0	0	0	0	0	0	0
Cym_helv	0	0	2	0	0	0	0	0	0	0
Cym_lanc	0	1	1	1	0	0	0	0	0	0
Cym_prox	0	0	0	0	0	0	0	0	0	0
Cyp_sole	0	0	0	0	0	0	0	0	0	0
Cys_dubi	0	0	0	0	0	0	0	0	0	0
Den_tecr	0	0	0	0	0	0	0	0	0	0

Dia_bott	0	0	0	0	0	0	0	0	0	0
Dia_moni	41	148	172	222	164	123	52	5	125	85
Dia_prob	0	0	0	0	0	0	0	0	0	0
Dia_tenu	0	0	0	0	0	0	0	0	0	0
Dia_vulg	0	1	0	0	0	0	0	0	1	0
Dic_subi	0	0	0	0	0	0	0	0	0	0
Dip_depa	0	0	0	0	0	0	0	0	0	0
Dip_dydi	0	0	0	0	0	0	0	0	0	0
Dip_elli	0	0	0	0	0	0	0	0	0	0
Dip_inte	0	0	0	0	0	0	0	0	0	0
Dip_ocul	0	0	0	0	0	0	0	0	0	0
Dip_smdi	0	0	0	0	0	0	0	0	1	0
Dip_smpu	0	0	0	0	0	0	0	0	0	0
Dip_smrh	0	0	0	0	0	0	0	0	0	0
Dip_subo	0	0	0	0	0	0	0	0	0	0
Enc_caes	0	1	3	1	0	0	0	3	0	3
Enc_lacu	0	0	0	0	0	0	0	0	0	0
Enc_minu	0	0	0	0	0	0	0	0	0	0
Enc_obsc	0	0	0	0	0	0	0	0	0	0
Enc_pros	0	0	0	0	0	0	0	0	0	0
Enc_sile	0	0	0	0	0	0	0	0	0	0
Enc_tenu	0	0	0	0	0	0	0	0	0	0
Enp_behr	0	0	0	0	0	0	0	0	0	0
Enp_kram	0	0	0	0	0	0	0	0	0	0
Ent_palu	1	0	0	1	0	0	0	0	1	0
Ent_pseu	1	1	0	1	0	0	0	0	2	0
Ent_punc	1	0	0	0	0	0	0	0	0	0
Epi_adna	0	1	0	0	0	2	0	1	0	0
Epi_goep	0	0	0	0	0	0	0	0	0	0
Epi_sore	7	8	10	2	5	9	5	15	2	4
Epi_turg	0	0	2	0	2	1	1	1	0	2
Epi_tuwe	0	0	0	0	0	0	0	0	0	0
Euc_laev	0	0	0	0	0	0	0	0	0	0
Eun_arcu	0	0	0	0	0	0	0	0	0	0
Eun_peun	0	0	0	0	0	0	0	0	0	0
Fal_clep	1	1	0	1	2	1	0	0	0	2
Fal_cryp	0	0	0	0	0	0	0	0	0	0
Fal_flor	0	0	0	0	0	0	0	0	0	0
Fal_psny	0	0	0	0	0	0	0	0	0	0
Fal_pygm	1	0	0	0	0	0	0	0	0	0
Fis_sapr	0	0	0	0	0	0	0	0	0	0

Fra_amic	0	0	0	0	0	0	0	0	0	0
Fra_capu	0	0	0	0	0	0	0	0	0	0
Fra_caru	6	4	4	9	0	9	3	0	0	0
Fra_cava	0	0	0	0	0	0	0	0	0	0
Fra_cons	0	0	0	0	0	0	0	0	0	0
Fra_geda	6	9	0	0	3	0	2	0	1	1
Fra_grac	0	0	0	0	0	0	0	0	0	0
Fra_hydu	0	0	0	0	0	0	0	0	0	0
Fra_lept	0	0	0	0	0	0	0	0	0	0
Fra_nitz	0	0	0	0	0	0	0	0	0	0
Frp_cyli	0	0	0	0	0	0	0	0	0	0
Fru_vulg	0	0	0	0	0	0	0	0	0	0
Gom_clav	0	0	0	0	0	0	0	0	0	0
Gom_intr	0	0	0	0	0	0	0	0	1	0
Gom_oliv	0	23	24	76	4	2	2	8	22	11
Gom_parv	0	0	0	0	0	0	0	0	0	0
Gon_exig	0	0	0	0	0	0	0	0	0	1
Gos_expl	0	0	0	0	0	0	0	0	0	0
Gos_obsc	0	0	0	0	0	0	0	0	0	1
Gra_mari	0	0	0	0	0	0	0	0	0	0
Gra_ocea	0	0	0	0	0	0	0	0	0	0
Gyr_acum	2	0	0	0	0	0	0	0	0	0
Gyr_atte	0	0	0	0	0	1	0	0	0	0
Gyr_stri	0	0	0	0	0	0	0	0	0	0
Gyr_tenu	0	0	0	0	0	0	0	0	0	0
Hal_coff	2	3	3	1	0	2	1	1	4	0
Hal_exig	0	0	0	0	0	0	0	0	0	0
Hal_hols	0	0	0	0	0	0	0	1	0	0
Hal_hybr	0	0	0	0	0	0	0	0	0	0
Hal_luci	0	0	0	0	0	0	3	0	0	0
Hal_norm	0	0	0	0	0	0	0	0	0	0
Hal_subh	0	0	0	0	0	0	0	0	0	0
Hal_tene	8	1	1	2	4	0	15	5	5	1
Hal_vene	0	0	0	0	0	0	0	0	0	0
Han_balt	0	0	0	0	0	0	0	0	0	0
Has_spic	0	2	0	0	0	2	0	0	0	0
Hip_capi	0	0	0	0	0	0	0	0	0	0
Hip_hung	0	0	0	0	0	0	0	0	0	0
Hip_lesm	0	0	0	0	0	0	0	0	0	0
Kar_amoe	1	3	0	0	0	1	3	0	1	0
Kar_clev	1	1	0	0	1	0	0	0	0	0

Kar_niti	0	0	0	0	0	0	0	0	0	0
Kar_subm	0	0	0	0	0	0	5	0	0	0
Lemn_hung	0	0	0	0	0	0	0	0	0	0
Lic_debi	0	0	0	0	0	0	0	0	0	0
Lic_gran	2	0	1	2	2	1	0	0	1	1
Lic_oedi	0	0	0	0	0	0	0	0	0	0
Lic_rhom	0	0	0	0	0	0	0	0	0	0
Lun_bise	17	6	12	11	8	2	7	12	28	11
Lut_muti	0	0	0	0	0	0	0	0	0	0
Mar_atom	95	25	10	20	9	46	30	5	7	15
Mar_mart	0	0	2	0	0	0	0	0	0	0
Mar_schu	0	0	0	0	0	0	2	0	0	0
Mas_balt	0	1	0	0	0	1	0	0	0	0
Mas_brau	0	0	0	0	0	0	0	1	0	0
Mas_elli	0	0	0	0	1	0	1	0	0	0
Mas_exig	0	0	0	0	0	0	0	0	0	0
Mas_pumi	2	2	0	1	1	2	1	2	0	0
Mas_pusi	0	0	0	0	0	0	0	0	0	0
Mas_smam	0	0	0	0	0	0	0	0	0	0
Mas_smit	1	11	20	5	2	23	3	21	1	8
Mel_arct	0	0	0	0	0	0	0	0	0	0
Mel_line	1	1	0	0	0	1	0	0	1	0
Mel_moni	0	0	0	0	0	0	0	0	0	0
Mel_numm	0	0	0	0	0	0	0	0	0	0
Mel_vari	0	0	0	0	0	0	0	0	0	0
Nav_aren	0	0	0	0	0	0	0	0	0	0
Nav_bipu	0	0	0	0	0	0	0	0	0	0
Nav_boss	0	0	0	0	0	0	0	2	4	0
Nav_bott	0	0	0	0	0	0	0	2	0	1
Nav_brem	0	0	0	0	0	0	0	0	0	0
Nav_capi	0	0	0	0	0	0	0	0	0	0
Nav_cinc	0	0	0	0	0	0	0	0	0	0
Nav_clem	0	0	0	0	0	0	0	0	0	0
Nav_cryp	0	0	0	0	1	1	1	3	4	2
Nav_digi	0	0	0	0	0	0	0	0	0	0
Nav_duer	0	0	0	0	0	0	0	0	0	0
Nav_eidr	0	0	0	0	0	0	0	0	0	0
Nav_flan	0	0	0	0	0	0	0	0	0	0
Nav_geii	3	2	0	0	0	0	14	0	0	0
Nav_gepo	0	0	0	0	0	0	0	0	0	0
Nav_greg	19	14	7	5	8	7	6	19	22	16

Nav_haca	0	0	0	0	0	0	0	0	0	0
Nav_hani	2	1	0	0	0	0	4	2	8	3
Nav_hust	0	0	0	0	0	0	0	0	0	0
Nav_lanc	5	7	24	19	2	3	3	56	45	22
Nav_late	0	0	0	1	0	0	0	0	0	0
Nav_marg	0	0	0	0	0	0	0	0	0	0
Nav_meni	0	0	0	0	0	0	0	0	0	0
Nav_midi	0	0	0	0	0	0	0	0	0	0
Nav_nole	0	0	0	0	0	0	0	0	0	0
Nav_oblo	0	0	0	0	0	0	0	0	0	0
Nav_oest	0	0	0	0	0	0	0	0	0	0
Nav_pere	0	0	0	0	16	10	0	0	0	0
Nav_perm	14	62	56	53	70	37	50	45	174	49
Nav_phyl	19	3	2	2	4	7	37	8	12	3
Nav_port	0	0	0	0	0	0	0	0	0	0
Nav_radi	0	0	0	0	0	0	0	0	0	0
Nav_ramo	0	0	0	0	0	0	0	0	1	1
Nav_rato	0	0	0	0	0	0	0	0	0	0
Nav_rece	0	0	0	0	0	0	0	0	0	0
Nav_rhyn	1	1	1	1	0	2	1	3	0	0
Nav_sana	1	0	0	0	0	0	0	0	0	0
Nav_sjoe	1	0	1	1	0	0	1	4	13	0
Nav_star	2	0	0	0	0	1	1	0	0	0
Nav_subr	0	0	0	0	0	0	0	0	0	0
Nav_supr	7	3	0	0	3	1	0	0	0	0
Nav_trip	0	0	0	0	0	0	0	0	0	0
Nav_vene	1	0	0	0	0	0	0	0	0	0
Ncy_pusi	0	0	0	0	0	0	0	0	0	0
Nit_acic	0	0	0	0	0	0	0	0	0	0
Nit_anfo	0	0	0	0	0	0	0	0	0	0
Nit_anla	0	0	0	0	4	1	0	0	0	0
Nit_arch	11	3	2	1	0	7	0	0	15	0
Nit_aura	0	0	0	0	0	0	0	0	1	0
Nit_baci	2	1	1	2	0	0	0	2	0	0
Nit_dime	0	0	0	0	0	0	0	0	0	0
Nit_dipp	0	0	0	0	0	0	0	0	0	0
Nit_diss	0	0	0	0	0	0	0	0	0	0
Nit_dist	4	0	0	0	0	0	0	0	0	0
Nit_dubi	0	0	0	0	0	0	0	0	0	0
Nit_eleg	0	0	0	0	0	0	0	0	0	0
Nit_frig	0	0	0	0	0	0	0	0	0	0

Nit_frus	39	134	316	178	262	365	182	556	118	402
Nit_gros	7	3	0	3	0	0	0	2	7	0
Nit_heuf	0	1	1	0	0	0	0	0	7	0
Nit_inco	0	0	0	0	0	0	0	0	0	0
Nit_inte	0	0	0	0	0	0	0	0	0	0
Nit_lieb	0	0	0	0	0	0	0	0	0	0
Nit_lisu	0	1	0	5	0	0	0	0	0	0
Nit_lore	1	0	0	0	0	0	0	0	0	0
Nit_micr	7	5	4	8	10	9	17	1	1	0
Nit_pale	3	0	4	0	0	0	0	0	13	0
Nit_pcea	0	0	0	0	0	0	0	0	1	0
Nit_pell	0	0	0	0	0	0	0	0	0	0
Nit_perm	0	0	0	0	0	0	0	0	0	0
Nit_pusi	6	13	8	19	6	14	0	0	15	2
Nit_rect	0	0	0	0	0	0	0	0	0	0
Nit_rose	2	34	17	21	1	7	7	6	14	8
Nit_saco	0	0	0	0	0	0	0	0	0	0
Nit_sdea	0	0	0	0	0	0	0	0	0	0
Nit_sigm	2	1	0	0	0	1	0	0	2	2
Nit_soci	0	0	0	0	0	0	0	0	0	0
Nit_soli	0	0	0	0	0	0	0	0	0	0
Nit_subl	0	0	0	0	0	0	0	0	0	0
Nit_ther	0	2	1	3	0	0	0	0	23	1
Nit_umbo	0	0	0	0	0	0	0	0	0	0
Nit_vald	6	5	0	0	9	0	4	3	0	0
Nit_verm	0	0	0	0	0	0	0	0	1	0
Nit_vitr	0	0	0	0	0	0	0	0	1	0
Nit_wuel	0	0	0	0	0	0	0	0	0	0
Ope_krum	0	0	0	0	0	0	0	0	0	0
Ope_muta	0	0	0	0	0	0	0	0	0	0
Par_prot	1	0	0	0	0	0	0	0	0	0
Pau_taen	0	0	0	1	0	0	0	0	0	0
Pet_hume	0	0	0	0	0	0	0	0	0	0
Pin_appe	2	0	0	0	0	0	0	0	2	0
Pin_dimi	0	0	0	0	0	0	0	0	0	0
Pin_kroc	0	0	0	0	0	0	0	0	0	0
Pin_micr	0	0	0	0	0	0	0	0	0	0
Plc_plac	0	0	0	0	0	0	0	0	0	0
Plt_cons	0	0	0	0	0	0	0	0	0	0
Plt_deli	8	2	3	0	7	6	18	7	2	5
Plt_disp	0	0	0	0	0	0	0	0	0	0

Plt_freq	0	0	0	0	0	0	0	0	0	0
Plt_hauc	27	4	3	1	4	7	18	9	3	3
Plt_lanc	0	0	0	0	0	0	0	0	0	0
Plt_lemm	0	0	0	0	0	0	2	0	0	0
Plt_link	0	0	0	0	0	0	0	0	0	0
Plt_peri	0	0	0	0	0	0	0	1	0	0
Plt_rost	0	0	0	0	0	0	0	0	0	0
Plt_sept	0	0	0	0	0	0	0	0	0	0
Psf_tene	0	0	0	0	0	0	0	0	0	0
Pss_brev	59	0	0	0	3	0	0	0	0	0
Pss_brin	14	4	0	0	0	0	1	0	0	0
Pss_elli	15	17	0	8	6	7	17	1	5	0
Pss_perm	49	39	4	7	8	23	28	19	5	2
Pss_robu	0	0	0	0	0	0	0	0	0	0
Pss_subs	0	0	0	0	0	0	0	0	0	0
Pss_zeil	75	53	18	23	17	10	12	9	2	1
Pst_bior	0	0	0	0	0	0	0	0	0	0
Pst_oblo	0	0	0	0	0	0	0	0	0	0
Pst_punc	0	0	2	0	0	0	0	0	2	0
Pst_ross	1	0	0	0	0	0	0	0	0	0
Pst_sacc	0	0	0	0	0	0	0	0	0	0
Pte_inan	0	0	0	0	0	0	0	0	0	0
Rei_sinu	0	0	0	0	0	0	0	0	0	0
Rho_abbr	10	50	85	169	195	89	223	54	13	190
Rhp_acum	0	0	0	0	0	0	0	0	0	0
Rhp_breb	0	0	0	0	0	0	0	0	0	0
Rhp_gibb	0	0	0	0	1	4	0	0	0	0
Sel_pupu	0	0	0	0	0	0	0	0	0	0
Sel_stro	0	0	0	0	0	0	0	0	0	0
Ske_cost	0	0	0	0	0	0	0	0	0	0
Ske_subs	0	0	0	0	0	0	0	0	0	0
Sla_lapp	0	0	0	0	0	0	0	0	0	0
Std_hant	0	0	0	0	0	0	0	0	0	0
Std_parv	0	0	0	0	0	0	0	0	0	0
Stn_legu	0	0	0	0	0	0	0	0	0	0
Stn_phoe	0	0	0	0	0	0	0	0	0	0
Stn_simu	0	0	0	0	0	0	0	0	0	0
Stn_smka	0	0	0	0	0	0	0	0	0	0
Stp_anus	0	0	0	0	0	0	0	0	0	0
Stp_wisl	0	0	0	0	0	0	0	0	0	0
Sts_punc	243	127	35	40	63	66	97	8	7	8

Sts_vent	15	0	0	0	0	0	0	0	0	0
Sur_angu	0	0	0	0	0	0	0	0	0	0
Sur_brba	0	0	0	0	0	0	0	0	0	0
Sur_breb	4	8	4	5	4	6	2	1	13	2
Sur_minu	0	0	0	0	0	0	0	0	0	0
Sur_oval	0	0	0	0	0	0	0	0	0	0
Syn_acus	0	0	0	0	0	0	0	0	0	0
Syn_ulna	0	0	0	0	0	0	0	0	0	0
Tab_fasc	0	0	3	1	7	6	1	0	1	1
Tab_kten	0	0	0	0	0	0	0	0	0	0
Tab_laev	6	7	2	3	14	8	6	2	3	1
Tab_tabu	0	2	2	0	1	0	0	1	3	4
Tab_waer	0	0	0	0	0	1	2	0	1	0
Tal_fene	0	0	0	0	0	0	0	0	0	0
Tal_floc	0	0	0	0	0	0	0	0	0	0
Tet_glan	0	0	0	0	0	0	0	0	0	0
Tha_balt	1	0	0	0	2	1	0	0	0	1
Tha_hyla	0	0	0	0	0	0	0	0	0	1
Tha_leva	3	5	17	18	5	6	3	1	17	6
Tha_pros	0	0	0	0	0	0	0	0	0	0
Tha_pseu	0	0	0	0	0	0	0	0	0	0
Tha_punc	0	0	0	0	0	0	0	0	0	0
Try_angu	0	0	0	0	0	0	0	0	0	0
Try_apic	1	0	1	0	0	1	0	0	1	0
Try_ardu	0	0	0	0	0	0	0	0	0	0
Try_hung	0	0	0	0	0	0	0	0	0	0
Try_levi	0	0	0	0	0	0	0	0	0	0
Try_lite	0	0	0	0	0	0	0	0	0	0
Try_sali	0	0	0	0	0	0	0	0	1	0
Try_scal	0	0	0	0	0	0	0	0	0	0

AREA	Gäv	Gäv	Gäv	Gäv	Gäv	Gäv	Gäv	Gäv	Gäv	Gäv
SITE	101	102	103	104	105	106	119	121	122	123
Acd_exil	0	0	0	0	0	0	0	0	0	0
Acd_minu	8	17	2	21	102	113	4	13	4	12
Acd_pyre	0	0	0	0	0	0	0	0	0	0
Ach_brev	0	0	0	0	0	0	0	0	0	0
Ach_brin	0	0	0	0	0	0	0	0	0	0
Ach_foge	0	0	0	0	0	0	3	0	0	0
Ach_leon	0	0	0	0	0	0	0	0	0	0
Ach_long	0	0	0	0	0	0	0	0	0	0
Ach_luth	0	0	0	0	0	0	0	0	0	0
Ach_misc	0	0	0	0	0	0	0	0	0	16
Ach_vist	0	0	0	0	0	0	2	0	0	0
Act_nosu	0	0	0	0	0	0	0	0	0	0
Act_occr	0	0	0	0	0	0	0	0	0	0
Ami_spec	0	0	0	0	0	0	0	0	0	0
Amp_copu	0	1	0	1	0	1	1	0	0	1
Amp_fleb	1	3	13	0	9	0	3	0	0	5
Amp_hele	0	0	0	0	0	0	0	0	0	0
Amp_inar	0	0	0	0	0	0	0	0	0	0
Amp_line	0	0	3	0	1	0	4	0	0	0
Amp_micr	0	0	0	0	0	0	0	0	0	0
Amp_oval	0	0	0	0	0	0	0	0	0	0
Amp_pedi	2	11	0	9	21	22	8	24	2	23
Amp_prof	0	0	0	0	0	0	0	0	0	0
Ane_mino	0	0	0	0	0	1	1	0	0	0
Ane_tusc	0	0	0	0	0	0	0	0	0	0
Ano_spha	0	0	0	0	0	0	0	0	0	0
Asa_bahu	0	0	0	0	0	0	0	0	0	0
Ast_form	0	0	0	0	0	0	0	0	0	0
Aul_spp	0	0	0	0	0	0	0	0	0	0
Bac_paxi	0	1	0	0	0	0	4	0	0	0
Ber_fenn	7	20	20	3	6	9	1	1	0	1
Ber_ruti	58	43	13	31	8	1	87	13	3	5
Ber_scop	0	0	0	0	0	0	0	0	0	0
Bir_luce	0	0	0	0	0	0	0	0	0	0
Bra_apon	0	0	0	0	2	0	0	0	0	0
Bra_vitr	0	0	0	0	0	0	0	0	0	0
Bre_lanc	0	2	0	5	1	0	0	0	0	0
Cal_aemu	0	0	0	0	0	0	0	0	0	0

Cal_amph	0	0	0	0	0	0	0	0	0	0
Cal_baci	0	0	0	2	0	0	0	0	0	0
Cal_cras	0	0	0	0	0	0	0	0	0	0
Cal_sili	0	0	0	0	0	0	0	0	0	0
Cam_clyp	0	0	0	1	1	1	0	0	0	0
Cat_adha	0	0	0	0	0	0	0	0	0	0
Cav_lapi	0	4	0	0	0	0	0	0	0	0
Cav_pseu	0	0	0	0	0	0	0	0	0	0
Cer_clos	0	0	0	0	0	0	0	0	0	0
Chp_clam	0	0	0	0	0	0	0	0	0	0
Chp_marg	1	3	0	0	1	0	0	0	0	1
Coc_cost	0	0	0	0	0	0	0	0	0	0
Coc_neot	0	2	0	0	2	12	2	0	0	5
Coc_pedi	2	2	1	0	0	3	2	0	0	5
Coc_pelt	0	0	0	0	0	0	0	0	0	0
Coc_plac	0	4	1	2	0	6	0	0	0	5
Coc_pleu	0	0	0	0	0	0	0	0	0	0
Coc_plli	0	0	0	0	0	0	0	0	0	0
Coc_psli	0	0	0	0	0	0	0	0	0	0
Coc_psth	0	0	0	0	0	0	0	0	0	0
Coc_scut	0	0	0	0	0	0	0	0	0	0
Coc_stau	0	0	0	0	0	0	0	0	0	0
Com_pusi	0	0	0	0	0	0	0	0	0	0
Cos_aste	0	0	0	0	0	0	0	0	0	0
Cte_pulc	1	16	2	12	2	5	9	4	2	4
Cyc_atom	0	0	0	0	0	0	0	0	0	0
Cyc_choc	0	0	0	0	0	0	0	0	0	0
Cyc_mene	0	0	0	0	0	0	0	0	0	0
Cyc_radi	0	0	0	0	0	0	0	0	0	0
Cyc_ross	0	0	0	0	0	0	0	0	0	0
Cyc_stel	0	0	0	0	0	0	0	0	0	0
Cyc_stri	0	0	0	0	0	0	0	0	0	0
Cyc_trip	0	0	0	0	0	0	0	0	0	0
Cym_affi	0	0	0	0	0	0	0	0	0	0
Cym_cist	0	0	0	0	0	0	0	0	0	0
Cym_helv	0	0	0	0	0	0	0	0	0	0
Cym_lanc	0	2	0	0	0	0	0	0	0	0
Cym_prox	0	0	0	0	0	0	0	0	0	0
Cyp_sole	0	0	0	0	0	0	0	0	0	0
Cys_dubi	0	0	0	0	0	0	0	0	0	0
Den_tecr	0	0	0	0	0	0	0	0	0	0

Dia_bott	0	0	0	0	0	0	0	0	0	0
Dia_moni	504	321	660	371	306	63	99	640	824	135
Dia_prob	0	0	0	0	0	0	0	0	0	0
Dia_tenu	0	0	0	1	0	0	0	0	0	0
Dia_vulg	0	0	1	0	1	0	2	15	6	3
Dic_subi	0	0	0	0	0	0	0	0	0	0
Dip_depa	0	0	0	0	0	0	0	0	0	0
Dip_dydi	0	0	0	0	0	0	0	0	0	0
Dip_elli	0	0	0	0	0	0	0	0	0	0
Dip_inte	0	0	0	0	0	0	0	0	0	0
Dip_ocul	0	0	0	0	0	0	0	0	0	0
Dip_smdi	0	0	0	0	0	0	0	0	0	0
Dip_smpu	0	0	0	0	0	0	0	0	0	0
Dip_smrh	0	0	0	1	0	0	0	0	0	0
Dip_subo	0	0	0	0	0	0	0	0	0	0
Enc_caes	0	0	0	2	0	3	0	0	0	0
Enc_lacu	0	0	0	0	0	0	0	0	0	0
Enc_minu	0	0	0	0	0	0	0	0	0	0
Enc_obsc	0	0	0	0	0	0	0	0	0	0
Enc_pros	0	0	0	0	0	0	0	0	0	0
Enc_sile	0	0	0	0	0	0	0	0	0	0
Enc_tenu	0	0	0	0	0	0	0	0	0	0
Enp_behr	0	0	0	0	0	0	0	0	0	0
Enp_kram	0	0	0	0	0	0	0	0	0	0
Ent_palu	0	0	0	0	0	1	1	0	0	3
Ent_pseu	0	2	0	5	3	0	1	0	0	0
Ent_punc	0	0	0	0	0	0	1	0	0	1
Epi_adna	0	0	0	2	0	1	0	0	0	0
Epi_goep	0	0	0	0	0	0	0	0	0	0
Epi_sore	1	9	7	5	3	6	2	0	0	2
Epi_turg	0	2	0	0	0	0	0	0	0	0
Epi_tuwe	0	0	0	0	0	0	2	0	0	0
Euc_laev	0	0	0	0	0	0	0	0	0	0
Eun_arcu	0	0	0	0	0	0	0	0	0	0
Eun_peun	0	0	0	0	0	0	0	0	0	0
Fal_clep	0	1	0	0	0	0	2	0	0	0
Fal_cryp	0	0	0	0	0	0	0	0	0	0
Fal_flor	0	0	0	0	0	0	0	0	0	0
Fal_psny	0	0	0	0	0	0	0	0	0	0
Fal_pygm	0	0	0	0	0	0	0	0	0	0
Fis_sapr	0	0	0	0	0	0	0	0	0	0

Fra_amic	0	0	0	0	0	0	0	0	0	0
Fra_capu	0	0	0	0	0	0	0	0	0	0
Fra_caru	0	0	0	7	15	20	5	1	0	0
Fra_cava	0	0	0	0	0	0	0	0	0	0
Fra_cons	0	0	0	0	0	0	0	0	0	0
Fra_geda	0	0	0	0	2	7	0	0	0	1
Fra_grac	0	0	0	0	0	0	0	0	0	0
Fra_hydu	0	0	0	0	0	0	0	0	0	0
Fra_lept	0	0	0	0	0	0	0	0	0	0
Fra_nitz	0	0	0	0	0	0	0	0	0	0
Frp_cyli	0	0	0	0	0	0	0	0	0	0
Fru_vulg	0	0	0	0	0	0	0	0	0	0
Gom_clav	0	0	0	0	0	0	0	0	0	0
Gom_intr	0	0	0	0	0	0	0	0	0	0
Gom_oliv	51	12	6	12	3	0	1	63	41	7
Gom_parv	0	0	0	0	0	0	0	0	0	0
Gon_exig	0	0	0	0	0	0	0	0	0	1
Gos_expl	0	0	0	0	0	0	0	0	0	0
Gos_obsc	0	0	0	0	0	0	0	0	0	1
Gra_mari	0	0	0	0	0	0	0	0	0	0
Gra_ocea	0	0	0	0	0	0	0	0	0	0
Gyr_acum	0	0	0	0	0	0	0	0	0	0
Gyr_atte	0	0	0	0	0	0	1	0	0	0
Gyr_stri	0	0	0	0	0	0	0	0	0	0
Gyr_tenu	0	0	0	0	0	0	0	0	0	0
Hal_coff	0	0	0	0	2	7	6	0	0	0
Hal_exig	0	0	0	0	0	0	0	0	0	0
Hal_hols	0	0	0	0	0	0	0	0	0	0
Hal_hybr	0	0	0	0	0	0	0	0	0	0
Hal_luci	0	0	0	0	0	0	0	0	0	0
Hal_norm	0	0	0	0	0	0	0	0	0	0
Hal_subh	0	0	0	0	0	0	0	0	0	0
Hal_tene	2	2	0	2	8	0	6	0	0	2
Hal_vene	0	0	0	0	0	0	0	0	0	0
Han_balt	0	0	0	0	0	0	0	0	0	0
Has_spic	0	0	0	0	1	1	0	0	0	0
Hip_capi	0	0	0	0	0	0	0	0	0	0
Hip_hung	0	0	0	0	0	0	0	0	0	0
Hip_lesm	0	0	0	0	0	0	0	0	0	0
Kar_amoe	0	0	0	0	1	0	0	0	0	1
Kar_clev	0	0	0	0	0	1	0	0	0	0

Kar_niti	0	0	0	0	0	0	0	0	0	0
Kar_subm	0	1	0	2	0	0	0	0	0	0
Lemn_hung	0	0	0	0	0	0	0	0	0	0
Lic_debi	0	0	0	0	0	0	0	0	0	0
Lic_gran	10	0	11	0	0	0	0	0	0	1
Lic_oedi	0	0	0	0	0	0	0	0	0	0
Lic_rhom	0	0	0	0	0	0	0	0	0	0
Lun_bise	6	14	0	21	24	13	41	9	8	23
Lut_muti	0	0	0	0	0	0	0	0	0	0
Mar_atom	0	13	0	33	60	70	34	0	0	15
Mar_mart	0	0	0	19	0	3	0	0	0	0
Mar_schu	0	0	0	0	0	1	1	0	0	0
Mas_balt	0	0	0	0	0	1	0	0	0	1
Mas_brau	0	0	0	0	0	0	0	0	0	0
Mas_elli	0	1	0	0	0	0	0	0	0	0
Mas_exig	0	0	0	0	0	0	0	0	0	0
Mas_pumi	0	0	0	1	0	4	0	0	0	1
Mas_pusi	0	0	0	0	0	0	0	0	0	0
Mas_smam	0	0	0	0	0	0	0	0	0	0
Mas_smit	0	3	6	1	3	8	2	0	0	4
Mel_arct	0	0	0	0	0	0	0	0	0	0
Mel_line	0	1	0	0	4	0	3	0	0	0
Mel_moni	0	0	0	0	0	0	0	0	0	0
Mel_numm	0	0	0	0	0	1	2	0	0	0
Mel_vari	0	0	0	0	0	0	0	0	0	0
Nav_aren	0	0	0	0	0	0	0	0	0	0
Nav_bipu	0	0	0	0	0	0	0	0	0	0
Nav_boss	0	0	0	0	0	0	0	0	0	0
Nav_bott	0	0	1	0	0	0	0	2	0	0
Nav_brem	0	0	0	0	0	0	0	0	0	0
Nav_capi	0	0	0	0	0	0	0	0	0	0
Nav_cinc	0	0	0	0	0	0	1	0	0	0
Nav_clem	0	0	0	0	0	0	0	0	0	0
Nav_cryp	0	0	0	0	0	0	0	0	0	0
Nav_digi	0	0	0	0	0	0	0	0	0	0
Nav_duer	0	0	0	0	0	0	0	0	0	0
Nav_eidr	0	0	0	0	0	0	0	0	0	0
Nav_flan	0	0	0	0	0	0	0	0	0	0
Nav_geii	0	0	0	0	0	0	0	0	0	0
Nav_gepo	0	0	0	0	0	0	0	0	0	0
Nav_greg	3	12	0	11	7	16	94	3	1	21

Nav_haca	0	0	0	0	0	0	0	0	0	0
Nav_hani	1	1	2	2	0	0	24	2	3	22
Nav_hust	0	0	0	0	0	0	0	0	0	0
Nav_lanc	4	2	2	5	4	0	13	4	2	11
Nav_late	0	0	0	0	0	0	0	0	0	0
Nav_marg	0	0	0	0	0	0	0	0	0	0
Nav_meni	0	0	0	0	0	0	0	0	0	0
Nav_midi	0	0	0	0	0	0	0	0	0	0
Nav_nole	0	0	0	0	0	0	0	0	0	0
Nav_oblo	0	0	0	0	0	0	0	0	0	0
Nav_oest	0	0	0	0	1	0	1	0	0	0
Nav_pere	0	0	0	0	0	0	0	0	0	0
Nav_perm	59	108	44	39	10	2	102	29	45	125
Nav_phyl	0	5	0	4	6	6	14	0	0	2
Nav_port	0	0	0	0	0	0	0	0	0	0
Nav_radi	0	0	0	0	0	0	0	0	0	0
Nav_ramo	0	0	2	1	0	0	2	0	0	0
Nav_rato	0	0	0	0	0	0	0	0	0	0
Nav_rece	0	0	0	0	0	0	0	0	0	0
Nav_rhyn	0	2	0	0	0	2	5	0	0	0
Nav_sana	0	0	0	0	0	0	0	0	0	0
Nav_sjoe	5	0	1	0	0	0	2	15	19	11
Nav_star	0	0	0	0	0	0	0	0	0	0
Nav_subr	0	0	0	0	0	0	0	0	0	0
Nav_supr	0	0	0	0	2	0	2	0	0	0
Nav_trip	0	0	0	0	0	0	0	0	0	0
Nav_vene	0	0	1	0	1	3	0	0	0	0
Ncy_pusi	0	0	0	0	0	0	0	0	0	0
Nit_acic	0	0	0	0	0	0	0	0	0	0
Nit_anfo	0	0	0	0	0	0	0	0	0	0
Nit_anla	0	0	0	0	0	0	0	0	0	0
Nit_arch	3	5	2	6	6	8	11	0	0	3
Nit_aura	0	0	0	0	0	1	0	0	0	0
Nit_baci	0	0	0	7	6	0	0	0	0	0
Nit_dime	0	0	0	0	0	0	0	0	0	0
Nit_dipp	0	0	0	0	0	0	1	0	0	0
Nit_diss	0	0	0	0	0	0	9	1	0	0
Nit_dist	0	0	0	0	1	0	2	0	0	1
Nit_dubi	0	0	0	0	0	0	0	0	0	0
Nit_eleg	0	0	0	0	2	2	1	0	0	0
Nit_frig	0	0	0	0	0	0	0	0	0	0

Nit_frus	158	181	98	138	16	15	108	131	18	327
Nit_gros	0	0	0	9	0	2	5	0	0	0
Nit_heuf	0	1	0	0	0	0	0	2	0	2
Nit_inco	0	0	0	0	0	0	0	0	0	0
Nit_inte	0	0	0	0	0	0	0	0	0	0
Nit_lieb	0	0	0	0	0	0	0	0	0	0
Nit_lisu	0	0	0	0	1	0	0	0	0	0
Nit_lore	0	0	0	0	0	0	0	0	0	0
Nit_micr	0	1	2	16	2	7	7	0	0	9
Nit_pale	0	2	0	4	7	2	7	0	0	0
Nit_pcea	0	0	0	0	0	0	1	0	0	0
Nit_pell	0	0	0	0	0	0	0	0	0	0
Nit_perm	0	0	0	0	0	0	0	0	0	0
Nit_pusi	1	17	0	14	20	1	6	3	1	5
Nit_rect	0	0	0	0	0	0	0	0	0	0
Nit_rose	2	9	6	2	3	2	6	0	0	2
Nit_saco	0	0	0	0	0	0	0	0	0	0
Nit_sdea	0	0	0	0	0	0	0	0	0	0
Nit_sigm	0	0	0	0	1	0	1	0	0	0
Nit_soci	0	0	0	0	0	0	0	0	0	0
Nit_soli	0	0	0	0	0	0	0	0	0	0
Nit_subl	0	0	0	0	0	0	0	0	0	0
Nit_ther	5	9	0	2	3	0	5	2	0	3
Nit_umbo	0	0	0	0	0	0	0	0	0	0
Nit_vald	2	0	0	1	4	6	10	2	0	2
Nit_verm	0	0	0	0	0	0	0	0	0	0
Nit_vitr	0	0	0	0	0	0	0	0	0	0
Nit_wuel	0	0	0	0	0	0	0	0	0	0
Ope_krum	0	0	0	0	0	0	0	0	0	0
Ope_muta	0	0	0	0	0	0	0	0	0	0
Par_prot	0	0	0	0	0	1	0	0	0	0
Pau_taen	0	0	0	0	0	0	0	0	0	0
Pet_hume	0	0	0	0	0	0	0	0	0	0
Pin_appe	0	0	0	0	0	0	2	0	0	0
Pin_dimi	0	0	0	0	0	0	0	0	0	0
Pin_kroc	0	0	0	0	0	0	0	0	0	0
Pin_micr	0	0	0	0	0	0	0	0	0	0
Plc_plac	0	0	0	0	0	0	0	0	0	0
Plt_cons	0	0	0	0	0	0	0	0	0	0
Plt_deli	1	1	0	1	5	2	11	3	0	5
Plt_disp	0	0	0	0	0	0	0	0	0	0

Plt_freq	0	0	0	0	0	0	0	0	0	0
Plt_hauc	0	0	0	0	5	10	10	0	0	3
Plt_lanc	0	0	0	0	0	1	0	0	0	0
Plt_lemm	0	0	0	0	0	0	0	0	0	0
Plt_link	0	0	0	0	0	0	0	0	0	0
Plt_peri	0	0	0	0	0	0	0	0	0	0
Plt_rost	0	0	0	0	0	0	0	0	0	0
Plt_sept	0	0	0	0	0	0	0	0	0	0
Psf_tene	0	0	0	0	0	0	0	0	0	0
Pss_brev	1	7	0	1	9	49	0	0	0	1
Pss_brin	0	0	0	7	18	42	1	0	0	0
Pss_elli	0	0	0	10	18	24	6	0	0	2
Pss_perm	0	2	0	17	31	67	20	0	0	6
Pss_robu	0	0	0	0	0	0	0	0	0	0
Pss_subs	0	0	0	0	0	0	0	0	0	0
Pss_zeil	0	7	0	33	91	209	21	0	0	7
Pst_bior	0	0	0	0	0	0	0	0	0	0
Pst_oblo	0	0	0	0	0	0	0	0	0	0
Pst_punc	0	0	0	0	2	0	1	1	0	0
Pst_ross	0	0	0	0	0	0	0	0	0	0
Pst_sacc	0	0	0	0	0	0	0	0	0	0
Pte_inan	0	0	0	0	0	0	0	0	0	0
Rei_sinu	0	0	0	0	0	0	0	0	0	0
Rho_abbr	78	59	63	31	4	13	21	11	13	109
Rhp_acum	0	0	0	0	0	0	0	0	0	0
Rhp_breb	0	0	0	0	0	0	0	0	0	0
Rhp_gibb	0	0	1	0	0	0	0	0	0	0
Sel_pupu	0	0	0	0	0	0	0	0	0	0
Sel_stro	0	0	0	0	0	0	0	0	0	0
Ske_cost	0	0	0	0	0	0	0	0	0	0
Ske_subs	0	0	0	0	0	0	0	0	0	0
Sla_lapp	0	0	0	0	0	0	0	0	0	0
Std_hant	0	0	0	0	0	0	0	0	0	0
Std_parv	0	0	0	0	0	0	0	0	0	0
Stn_legu	0	0	0	0	0	0	2	0	0	0
Stn_phoe	0	0	0	0	0	0	0	0	0	0
Stn_simu	0	0	0	0	0	0	0	0	0	0
Stn_smka	0	0	0	0	0	0	0	0	0	0
Stp_anus	0	0	0	0	0	0	0	0	0	0
Stp_wisl	0	0	0	0	0	0	0	0	0	0
Sts_punc	2	13	7	40	100	100	73	0	0	11

Sts_vent	0	0	0	0	0	0	0	0	0	0
Sur_angu	0	0	0	0	0	0	0	0	0	0
Sur_brba	0	0	0	0	0	0	0	0	0	0
Sur_breb	3	2	0	2	7	3	4	0	1	4
Sur_minu	0	0	0	0	0	0	0	0	0	0
Sur_oval	0	0	0	0	0	0	0	0	0	0
Syn_acus	0	0	0	0	0	0	0	0	0	0
Syn_ulna	0	0	0	0	0	0	4	0	0	0
Tab_fasc	3	4	8	5	0	0	7	0	6	3
Tab_kten	0	0	0	0	0	0	0	0	0	0
Tab_laev	1	17	4	11	6	16	21	2	0	4
Tab_tabu	1	4	4	1	0	2	1	1	0	2
Tab_waer	10	6	4	6	0	0	1	2	0	3
Tal_fene	0	0	0	0	0	0	0	0	0	0
Tal_floc	0	0	0	0	0	0	0	0	0	0
Tet_glan	0	0	0	0	0	0	0	0	0	0
Tha_balt	0	3	0	0	0	0	0	0	0	0
Tha_hyla	0	0	0	0	0	0	0	0	0	0
Tha_leva	3	7	2	1	4	0	6	1	1	7
Tha_pros	0	0	0	0	0	0	0	0	0	0
Tha_pseu	0	0	0	0	0	0	0	0	0	0
Tha_punc	0	0	0	0	0	0	0	0	0	0
Try_angu	0	0	0	0	0	0	0	0	0	0
Try_apic	0	0	0	1	0	0	0	0	0	0
Try_ardu	0	0	0	0	0	0	0	0	0	0
Try_hung	0	0	0	0	0	0	0	0	0	0
Try_levi	0	0	0	0	0	0	0	0	0	0
Try_lite	0	0	0	0	0	0	0	0	0	0
Try_sali	0	0	0	0	5	1	5	0	0	2
Try_scal	0	0	0	0	0	0	0	0	0	0

AREA<br>SITE	Hor<br>125	Hor<br>126	Hor<br>127	Hor<br>128	Hor<br>129	Hor<br>130	Hor<br>131	Hor<br>132	Hor<br>133	Hor<br>134
Acd_exil	0	0	0	0	0	0	0	0	0	0
Acd_minu	10	102	6	77	41	34	172	49	64	8
Acd_pyre	0	0	0	0	0	0	0	0	0	0
Ach_brev	0	0	0	0	0	0	0	0	0	0
Ach_brin	0	0	0	0	0	0	0	0	0	0
Ach_foge	0	0	0	0	0	0	0	0	0	0
Ach_leon	0	0	0	0	0	0	0	0	0	0
Ach_long	0	0	0	0	0	0	0	0	0	0
Ach_luth	0	0	0	0	0	0	0	0	0	0
Ach_misc	1	1	7	9	0	0	2	3	2	18
Ach_vist	0	0	0	0	0	0	0	0	0	0
Act_nosu	0	0	0	0	0	0	0	0	0	1
Act_occr	0	0	0	0	0	0	0	0	0	0
Ami_spec	0	0	0	0	0	0	0	0	0	0
Amp_copu	0	2	0	0	2	0	0	2	0	4
Amp_fleb	1	2	2	8	0	2	3	3	0	4
Amp_hele	0	0	0	0	0	0	0	0	0	0
Amp_inar	0	0	0	0	0	0	0	0	0	0
Amp_line	0	0	0	0	0	0	0	0	0	3
Amp_micr	0	0	0	0	0	0	0	0	0	0
Amp_oval	0	0	0	0	0	0	0	0	0	0
Amp_pedi	9	7	13	7	10	10	2	12	10	11
Amp_prof	0	0	0	0	0	0	0	0	0	0
Ane_mino	1	0	0	0	0	0	0	0	0	0
Ane_tusc	0	0	0	0	0	0	0	0	0	0
Ano_spha	0	0	0	0	0	0	0	0	0	0
Asa_bahu	0	0	0	0	0	0	0	0	0	0
Ast_form	0	0	0	0	0	0	0	0	0	0
Aul_spp	0	0	0	0	0	0	0	0	0	0
Bac_paxi	1	0	0	0	0	0	0	0	0	0
Ber_fenn	16	4	6	3	12	1	8	4	5	12
Ber_ruti	0	0	0	0	0	0	0	0	0	0
Ber_scop	0	0	0	0	0	0	0	0	0	0
Bir_luce	0	0	0	0	0	0	0	0	0	0
Bra_apon	0	0	0	0	0	0	0	0	0	0
Bra_vitr	0	0	0	0	0	0	0	0	0	0
Bre_lanc	0	0	0	0	1	0	0	0	0	0
Cal_aemu	0	0	0	0	0	0	0	0	0	0

Cal_amph	0	0	0	0	0	0	0	0	0	0
Cal_baci	0	0	0	4	1	0	0	0	0	1
Cal_cras	0	0	0	0	0	0	0	0	0	0
Cal_sili	0	0	0	0	0	0	0	0	0	0
Cam_clyp	0	0	0	0	0	0	0	0	0	0
Cat_adha	0	0	0	0	0	0	0	0	0	0
Cav_lapi	0	0	0	0	0	0	0	0	0	0
Cav_pseu	0	0	0	0	0	0	0	0	0	0
Cer_clos	0	0	0	0	0	0	0	0	0	0
Chp_clam	0	0	0	0	0	0	0	0	0	0
Chp_marg	0	0	0	0	1	0	3	1	1	0
Coc_cost	0	0	0	0	0	0	0	0	0	0
Coc_neot	0	1	0	4	1	0	1	3	9	1
Coc_pedi	2	8	2	3	3	1	9	13	8	4
Coc_pelt	0	0	0	0	0	0	0	0	0	0
Coc_plac	0	3	0	0	0	0	0	1	0	4
Coc_pleu	0	0	0	0	0	0	0	0	0	0
Coc_plli	0	0	0	0	0	0	0	0	0	0
Coc_psli	0	0	0	0	0	0	0	0	0	0
Coc_psth	0	0	0	0	0	0	0	0	0	0
Coc_scut	0	1	0	0	0	0	0	0	0	0
Coc_stau	0	0	0	0	0	0	0	0	0	0
Com_pusi	0	0	0	0	0	0	0	0	0	0
Cos_aste	0	0	0	0	0	0	0	0	0	0
Cte_pulc	7	31	14	11	21	12	5	2	29	8
Cyc_atom	0	0	0	0	0	0	0	0	0	0
Cyc_choc	0	0	0	0	0	0	0	0	0	0
Cyc_mene	0	0	0	0	0	0	0	0	0	0
Cyc_radi	0	0	0	0	0	0	0	0	0	0
Cyc_ross	0	0	0	0	0	0	0	0	0	0
Cyc_stel	0	0	0	0	0	0	0	0	0	0
Cyc_stri	0	0	0	0	0	0	0	0	0	0
Cyc_trip	0	0	0	0	0	0	0	0	0	0
Cym_affi	0	0	0	0	0	0	0	0	0	0
Cym_cist	0	0	0	0	0	0	0	0	0	0
Cym_helv	0	0	0	0	0	0	0	0	0	0
Cym_lanc	0	0	0	0	0	0	0	0	1	0
Cym_prox	0	0	0	0	0	0	0	0	0	0
Cyp_sole	0	0	0	0	0	0	0	0	0	0
Cys_dubi	0	0	0	0	0	0	0	0	0	0
Den_tecr	0	0	0	0	0	0	0	0	0	0

Dia_bott	0	0	0	0	0	0	0	0	0	0
Dia_moni	171	350	589	48	229	683	280	373	510	62
Dia_prob	0	0	0	0	0	0	0	0	0	0
Dia_tenu	0	0	0	0	0	0	0	0	0	0
Dia_vulg	0	0	0	0	0	0	0	0	0	0
Dic_subi	0	0	0	0	0	0	0	0	0	0
Dip_depa	0	0	0	0	0	0	0	0	0	0
Dip_dydi	0	0	0	0	0	0	0	0	0	0
Dip_elli	0	0	0	0	0	0	0	0	0	0
Dip_inte	0	1	0	0	0	0	0	0	0	0
Dip_ocul	0	0	0	0	0	0	0	0	0	0
Dip_smdi	0	0	0	0	0	0	0	0	0	0
Dip_smpu	0	0	0	0	0	0	0	0	0	0
Dip_smrh	0	0	0	1	0	0	0	0	0	0
Dip_subo	0	0	0	0	0	0	0	0	0	0
Enc_caes	1	2	0	2	2	0	1	0	1	0
Enc_lacu	0	0	0	0	0	0	0	0	0	0
Enc_minu	0	0	0	0	0	0	0	0	0	0
Enc_obsc	0	0	0	0	0	0	0	0	0	0
Enc_pros	0	0	0	0	0	0	0	0	0	0
Enc_sile	1	0	0	0	0	0	0	0	0	0
Enc_tenu	0	0	0	0	0	0	0	0	0	0
Enp_behr	0	0	0	0	0	0	0	0	0	0
Enp_kram	0	0	0	0	0	0	0	0	0	0
Ent_palu	0	0	1	0	0	0	0	0	0	1
Ent_pseu	0	0	0	0	0	0	0	0	0	0
Ent_punc	0	0	0	0	0	0	0	0	0	0
Epi_adna	0	0	0	1	0	0	0	0	0	0
Epi_goep	0	0	0	0	0	0	0	0	0	0
Epi_sore	10	13	6	22	17	1	10	2	3	3
Epi_turg	2	5	1	1	2	1	2	3	2	2
Epi_tuwe	0	0	0	0	0	0	0	0	0	0
Euc_laev	0	0	0	0	0	0	0	0	0	0
Eun_arcu	0	0	0	0	1	0	0	0	0	0
Eun_peun	0	0	0	0	0	0	0	0	0	0
Fal_clep	0	1	0	0	1	0	0	0	0	0
Fal_cryp	0	0	0	0	0	0	0	0	0	0
Fal_flor	0	0	0	0	0	0	0	0	0	0
Fal_psny	0	0	0	0	0	0	0	0	0	0
Fal_pygm	0	0	0	0	0	0	0	0	0	2
Fis_sapr	0	0	0	0	0	0	0	0	0	0

Fra_amic	0	0	0	0	0	0	0	0	0	0
Fra_capu	0	0	0	0	0	0	0	0	0	0
Fra_caru	52	0	1	36	12	0	0	0	0	0
Fra_cava	0	0	0	0	0	0	0	0	0	0
Fra_cons	0	0	0	0	0	0	0	0	0	0
Fra_geda	0	0	0	14	3	0	0	0	1	0
Fra_grac	0	0	0	0	0	0	0	0	1	0
Fra_hydu	0	0	0	0	0	0	0	0	0	0
Fra_lept	0	0	0	0	0	0	0	0	0	0
Fra_nitz	0	0	0	0	0	0	0	0	0	0
Frp_cyli	0	0	0	0	0	0	0	0	1	1
Fru_vulg	0	0	0	0	0	0	0	0	0	0
Gom_clav	0	0	0	0	0	0	0	0	0	0
Gom_intr	1	0	0	0	0	0	0	0	0	0
Gom_oliv	0	6	9	1	10	5	7	12	20	4
Gom_parv	0	0	0	0	0	0	0	0	0	0
Gon_exig	0	0	0	0	1	23	5	3	4	1
Gos_expl	0	0	0	0	0	0	0	0	0	0
Gos_obsc	0	3	0	0	3	0	0	0	0	0
Gra_mari	0	0	0	0	0	0	0	0	0	0
Gra_ocea	0	0	0	0	0	0	0	0	0	1
Gyr_acum	0	0	0	0	0	0	0	0	0	0
Gyr_atte	0	0	0	0	0	0	0	0	0	0
Gyr_stri	0	0	0	0	0	0	0	0	0	0
Gyr_tenu	0	0	0	0	0	0	0	0	0	0
Hal_coff	2	4	1	0	2	0	5	0	0	7
Hal_exig	0	0	0	0	0	0	0	0	0	0
Hal_hols	0	0	0	0	0	1	0	0	0	0
Hal_hybr	0	0	0	0	0	0	0	0	0	0
Hal_luci	0	0	0	0	0	0	0	0	0	0
Hal_norm	0	0	0	0	0	0	0	0	0	0
Hal_subh	0	0	0	0	0	0	0	0	0	0
Hal_tene	0	2	0	0	0	0	0	1	3	0
Hal_vene	0	0	0	0	0	0	0	0	0	0
Han_balt	0	0	0	0	0	0	0	0	0	0
Has_spic	0	0	1	0	0	0	0	0	0	0
Hip_capi	0	0	0	0	0	0	0	0	0	1
Hip_hung	0	0	0	0	0	0	0	0	0	0
Hip_lesm	0	0	0	0	0	0	0	0	0	0
Kar_amoe	0	0	0	0	0	0	0	3	0	1
Kar_clev	0	0	0	0	0	0	0	0	0	0

Kar_niti	0	0	0	0	0	0	0	0	0	0
Kar_subm	0	4	0	0	1	0	1	0	0	0
Lemn_hung	0	0	0	0	0	0	0	0	0	0
Lic_debi	0	0	0	0	0	0	0	0	0	0
Lic_gran	0	1	4	0	1	10	16	24	13	2
Lic_oedi	0	0	0	0	0	0	0	0	0	0
Lic_rhom	0	0	0	0	0	0	0	0	0	0
Lun_bise	6	32	6	1	0	0	1	1	3	9
Lut_muti	0	0	0	0	0	0	0	0	0	0
Mar_atom	10	1	0	104	59	0	3	1	1	100
Mar_mart	0	0	0	0	0	0	0	0	0	6
Mar_schu	1	0	0	6	3	0	0	0	0	13
Mas_balt	0	0	0	0	0	0	0	0	0	0
Mas_brau	0	0	0	0	0	0	0	0	0	0
Mas_elli	0	2	0	2	0	0	0	0	0	0
Mas_exig	0	0	0	0	0	0	0	0	0	0
Mas_pumi	0	0	0	0	0	0	0	0	0	0
Mas_pusi	0	0	0	0	0	0	0	0	0	0
Mas_smam	0	0	0	0	0	0	0	0	0	0
Mas_smit	7	6	1	12	9	0	2	0	3	4
Mel_arct	0	0	0	0	0	0	0	0	0	0
Mel_line	0	0	0	0	0	0	0	0	0	1
Mel_moni	0	4	0	0	1	0	0	1	0	0
Mel_numm	0	0	0	0	0	0	0	0	0	0
Mel_vari	0	0	0	0	0	0	0	0	0	0
Nav_aren	0	0	0	0	0	0	0	0	0	0
Nav_bipu	0	0	0	0	0	0	0	0	0	0
Nav_boss	0	0	0	0	0	0	2	0	0	3
Nav_bott	0	3	1	1	0	4	3	3	0	0
Nav_brem	0	0	0	0	0	0	0	0	0	0
Nav_capi	0	0	0	0	0	0	0	0	0	0
Nav_cinc	0	0	0	0	1	1	1	0	0	0
Nav_clem	0	0	0	0	0	0	0	0	0	0
Nav_cryp	0	2	1	0	0	0	0	0	0	0
Nav_digi	0	0	0	0	0	0	0	0	0	0
Nav_duer	0	0	0	0	0	0	0	0	0	4
Nav_eidr	0	0	0	0	0	0	0	0	0	0
Nav_flan	0	0	0	0	0	0	0	0	0	0
Nav_geii	0	0	0	0	0	0	0	0	0	0
Nav_gepo	0	0	0	0	0	0	0	0	0	0
Nav_greg	12	12	10	16	11	3	9	13	4	27

Nav_haca	0	0	0	0	0	0	0	0	0	0
Nav_hani	0	8	9	0	10	11	28	16	16	0
Nav_hust	0	0	0	0	0	0	0	0	0	0
Nav_lanc	1	4	5	4	0	2	3	4	0	5
Nav_late	0	1	0	0	1	0	0	0	0	2
Nav_marg	0	0	0	0	0	0	0	0	0	0
Nav_meni	0	0	0	0	0	0	0	0	0	1
Nav_midi	0	0	0	0	0	0	0	0	0	0
Nav_nole	0	0	0	0	0	0	0	0	0	0
Nav_oblo	0	0	0	0	0	0	0	0	0	0
Nav_oest	0	0	0	0	0	0	0	0	0	0
Nav_pere	0	0	0	0	0	0	0	0	0	0
Nav_perm	16	39	60	17	34	26	78	70	32	61
Nav_phyl	16	7	6	0	2	3	11	5	0	16
Nav_port	0	0	0	0	0	0	0	0	0	0
Nav_radi	0	0	0	0	0	0	0	0	0	0
Nav_ramo	1	1	3	0	0	2	11	12	0	0
Nav_rato	0	0	0	0	0	0	0	0	0	0
Nav_rece	0	0	0	0	0	0	0	0	0	0
Nav_rhyn	1	0	0	2	0	0	0	0	0	7
Nav_sana	0	0	0	0	0	0	0	0	0	0
Nav_sjoe	3	3	8	1	4	4	15	18	0	0
Nav_star	0	0	0	0	0	0	0	0	0	0
Nav_subr	0	0	0	0	0	0	0	0	0	0
Nav_supr	0	0	0	0	0	0	0	0	0	0
Nav_trip	0	0	0	0	0	0	0	0	0	0
Nav_vene	1	1	0	0	0	0	0	2	0	0
Ncy_pusi	0	0	0	0	0	0	0	0	0	0
Nit_acic	0	0	0	0	0	0	0	0	0	0
Nit_anfo	0	0	0	0	0	0	0	0	0	0
Nit_anla	0	0	0	0	0	0	0	0	0	0
Nit_arch	0	0	0	0	0	0	0	0	0	0
Nit_aura	0	0	0	0	0	0	0	0	0	0
Nit_baci	0	0	0	0	0	0	0	0	0	0
Nit_dime	0	0	0	0	0	0	0	0	0	0
Nit_dipp	1	3	0	0	0	0	0	0	0	0
Nit_diss	1	0	0	0	0	0	0	0	0	0
Nit_dist	0	0	0	0	0	0	0	0	0	0
Nit_dubi	0	0	0	0	0	0	0	0	0	0
Nit_eleg	0	0	0	0	2	0	0	0	0	0
Nit_frig	0	0	0	0	0	0	0	0	0	0

Nit_frus	477	168	120	196	223	67	159	181	134	72
Nit_gros	0	0	0	0	0	0	0	0	0	0
Nit_heuf	0	0	1	0	1	0	0	0	0	0
Nit_inco	0	0	0	0	0	0	0	0	0	0
Nit_inte	0	0	0	0	0	0	0	0	0	0
Nit_lieb	0	0	0	0	0	0	0	0	0	0
Nit_lisu	0	0	0	0	0	0	0	0	0	0
Nit_lore	0	0	0	0	0	0	0	0	0	0
Nit_micr	26	11	26	6	6	0	11	1	0	9
Nit_pale	0	0	0	5	0	0	0	0	0	0
Nit_pcea	6	1	2	8	0	0	0	0	0	0
Nit_pell	0	0	0	0	0	0	0	0	0	0
Nit_perm	0	0	0	0	0	0	0	0	0	0
Nit_pusi	2	1	2	2	0	0	0	0	6	3
Nit_rect	0	0	0	0	0	0	0	0	0	0
Nit_rose	0	0	2	5	10	1	2	1	5	0
Nit_saco	0	0	0	0	0	0	0	0	0	0
Nit_sdea	0	0	0	0	0	0	0	0	0	0
Nit_sigm	0	0	0	0	0	0	0	0	0	0
Nit_soci	0	0	0	0	0	0	0	0	0	0
Nit_soli	0	0	0	0	0	0	0	0	0	0
Nit_subl	0	0	0	0	0	0	0	0	0	0
Nit_ther	1	0	0	0	0	0	0	0	0	4
Nit_umbo	0	0	0	0	0	0	0	0	0	0
Nit_vald	13	1	3	2	1	0	4	0	2	5
Nit_verm	0	0	0	0	0	0	0	0	0	0
Nit_vitr	0	0	0	0	0	0	0	0	0	0
Nit_wuel	0	0	0	0	0	0	0	0	0	0
Ope_krum	0	0	0	0	0	0	0	0	0	10
Ope_muta	0	0	0	0	0	0	0	0	0	6
Par_prot	0	0	0	0	0	0	0	0	0	2
Pau_taen	0	0	0	0	0	0	0	0	1	2
Pet_hume	0	0	0	0	0	0	0	0	0	0
Pin_appe	0	0	0	1	0	0	0	0	0	0
Pin_dimi	0	0	0	0	0	0	0	0	0	0
Pin_kroc	0	0	0	0	0	0	0	0	0	0
Pin_micr	0	0	0	0	0	0	0	0	0	0
Plc_plac	0	0	0	0	0	0	0	0	0	0
Plt_cons	0	0	0	0	0	0	0	0	0	0
Plt_deli	4	4	4	3	5	2	1	9	1	16
Plt_disp	0	0	0	0	0	0	0	0	0	0

Plt_freq	0	0	0	0	0	0	0	0	0	0
Plt_hauc	13	6	0	8	6	0	1	15	2	10
Plt_lanc	0	0	0	0	0	0	0	0	0	0
Plt_lemm	0	0	0	0	0	0	0	0	0	0
Plt_link	0	0	0	0	0	0	0	0	0	0
Plt_peri	0	1	0	0	0	0	0	0	0	8
Plt_rost	0	0	0	0	0	0	0	0	0	0
Plt_sept	0	0	0	0	0	0	0	0	0	0
Psf_tene	0	0	0	0	0	0	0	0	0	0
Pss_brev	0	0	0	0	0	0	0	0	0	56
Pss_brin	0	0	0	0	0	0	1	0	0	6
Pss_elli	0	0	0	8	5	0	0	0	0	17
Pss_perm	10	1	0	55	21	2	7	5	3	171
Pss_robu	0	0	0	0	0	0	0	0	0	0
Pss_subs	0	0	0	0	0	0	0	0	0	0
Pss_zeil	2	3	1	13	24	0	0	3	0	26
Pst_bior	0	0	0	0	0	0	0	0	0	0
Pst_oblo	0	0	0	3	0	0	0	0	0	0
Pst_punc	5	1	1	0	2	0	0	5	0	1
Pst_ross	0	0	0	0	0	0	0	0	0	0
Pst_sacc	0	0	0	0	0	0	0	0	0	0
Pte_inan	0	0	0	0	0	0	0	0	0	0
Rei_sinu	0	0	0	0	0	0	0	0	0	0
Rho_abbr	58	87	20	171	130	40	64	65	51	18
Rhp_acum	0	0	0	0	0	0	0	0	0	0
Rhp_breb	0	0	0	0	0	0	0	0	0	0
Rhp_gibb	0	0	0	0	0	0	0	0	0	0
Sel_pupu	0	0	0	0	0	0	0	0	0	0
Sel_stro	0	0	0	0	0	0	0	0	0	0
Ske_cost	0	0	0	0	0	0	0	0	0	0
Ske_subs	0	0	0	0	0	0	0	0	0	0
Sla_lapp	0	0	0	0	0	0	0	0	0	0
Std_hant	0	0	0	0	0	0	0	0	0	0
Std_parv	0	0	0	0	0	0	0	0	0	0
Stn_legu	0	0	0	0	0	0	0	0	0	0
Stn_phoe	0	0	0	0	0	0	0	0	0	0
Stn_simu	0	0	0	0	0	0	0	0	0	0
Stn_smka	0	0	0	0	0	0	0	0	0	0
Stp_anus	0	0	0	0	0	0	0	0	0	0
Stp_wisl	0	0	0	0	0	0	0	0	0	1
Sts_punc	5	0	0	83	39	0	0	5	4	95

Sts_vent	0	0	0	2	0	0	0	0	0	16
Sur_angu	0	0	0	0	0	0	0	0	0	0
Sur_brba	0	0	0	0	0	0	0	0	0	0
Sur_breb	8	3	2	1	0	0	0	0	2	0
Sur_minu	0	0	0	0	0	0	0	0	0	0
Sur_oval	0	0	0	0	0	0	0	0	0	0
Syn_acus	0	0	0	0	0	0	0	0	0	0
Syn_ulna	0	0	0	0	0	0	0	0	0	0
Tab_fasc	0	12	14	1	3	39	40	33	24	6
Tab_kten	0	0	0	0	0	0	0	0	0	0
Tab_laev	4	11	20	5	4	1	4	2	5	9
Tab_tabu	0	3	2	0	4	0	0	4	4	1
Tab_waer	0	2	3	0	0	4	4	6	3	0
Tal_fene	0	0	0	0	0	0	0	0	0	0
Tal_floc	0	0	0	1	1	0	0	0	0	0
Tet_glan	0	0	0	0	0	0	0	0	0	0
Tha_balt	0	0	0	0	0	0	0	0	0	0
Tha_hyla	1	0	0	0	0	0	2	0	2	0
Tha_leva	0	1	4	3	0	4	1	5	4	3
Tha_pros	0	0	0	0	0	0	0	0	0	0
Tha_pseu	0	0	0	0	0	0	0	0	0	0
Tha_punc	0	0	0	0	0	0	0	0	0	0
Try_angu	0	0	0	0	0	0	0	0	0	0
Try_apic	0	0	0	0	0	0	0	0	0	0
Try_ardu	0	0	0	0	0	0	0	0	0	0
Try_hung	0	0	0	0	0	0	0	0	0	0
Try_levi	0	0	0	0	0	0	0	0	0	0
Try_lite	0	0	0	0	0	0	0	0	0	0
Try_sali	0	0	0	0	0	0	0	0	0	1
Try_scal	0	0	0	0	0	0	0	0	0	0

AREA<br>SITE	Omn<br>136	Omn<br>137	Omn<br>138	Omn<br>139	Omn<br>140	Omn<br>141	Omn<br>142	Omn<br>143	Omn<br>144	Omn<br>145	Omn<br>146
Acd_exil	0	0	0	0	0	0	0	0	0	0	0
Acd_minu	13	15	4	19	6	34	23	41	27	18	43
Acd_pyre	0	0	0	0	0	0	0	0	0	0	0
Ach_brev	0	0	0	0	0	0	0	0	0	0	0
Ach_brin	0	0	0	0	0	0	0	0	0	0	0
Ach_foge	0	0	0	0	0	0	0	0	0	0	0
Ach_leon	0	0	0	0	0	0	0	0	0	0	0
Ach_long	0	0	0	0	0	0	0	0	0	0	0
Ach_luth	0	0	0	0	0	0	0	0	0	0	0
Ach_misc	3	0	4	2	0	2	3	1	0	3	2
Ach_vist	5	0	4	6	6	3	2	1	0	0	0
Act_nosu	0	0	0	0	0	0	0	0	0	0	0
Act_occr	0	0	0	0	0	0	0	0	0	0	0
Ami_spec	0	0	0	0	0	0	0	0	0	0	0
Amp_copu	0	0	1	0	0	0	0	1	0	3	0
Amp_fleb	0	0	0	0	0	0	0	0	0	0	0
Amp_hele	0	0	0	0	0	0	0	0	0	0	0
Amp_inar	0	0	0	0	0	0	0	0	0	0	0
Amp_line	0	0	3	1	1	2	0	0	0	0	0
Amp_micr	0	0	0	2	0	4	0	0	0	0	0
Amp_oval	0	0	0	0	0	0	0	0	0	0	0
Amp_pedi	0	0	0	7	6	11	2	9	1	4	2
Amp_prof	0	0	0	0	0	0	0	0	0	0	0
Ane_mino	0	0	0	0	0	0	0	0	0	0	0
Ane_tusc	0	0	0	0	0	0	0	0	0	0	0
Ano_spha	0	0	0	0	0	0	0	0	0	0	0
Asa_bahu	0	0	0	0	0	0	0	0	0	0	0
Ast_form	0	0	0	0	0	0	0	0	0	0	0
Aul_spp	0	0	0	3	0	0	0	0	0	0	0
Bac_paxi	0	0	0	0	0	0	0	0	0	0	0
Ber_fenn	0	0	53	4	21	18	4	11	0	1	32
Ber_ruti	33	4	14	34	47	19	0	54	28	10	2
Ber_scop	0	0	0	0	0	0	0	0	0	0	0
Bir_luce	0	0	0	0	0	0	0	0	0	0	0
Bra_apon	0	0	0	0	0	0	0	0	0	0	0
Bra_vitr	0	0	0	0	0	0	0	0	0	0	1
Bre_lanc	0	0	0	0	1	0	0	0	0	0	0
Cal_aemu	0	0	0	0	0	0	0	0	0	0	0

Cal_amph	0	0	0	0	0	0	0	0	0	0	0
Cal_baci	0	0	0	0	0	0	0	0	0	0	0
Cal_cras	0	0	0	0	0	0	0	0	0	0	0
Cal_sili	0	0	0	0	0	0	0	0	0	0	0
Cam_clyp	0	0	0	0	0	0	0	0	0	0	0
Cat_adha	0	0	0	0	1	0	0	0	0	1	0
Cav_lapi	0	0	0	0	0	0	0	0	0	0	0
Cav_pseu	0	0	0	0	0	0	0	0	0	0	0
Cer_clos	0	0	0	0	0	0	0	0	0	0	0
Chp_clam	0	0	0	0	0	0	0	0	0	0	0
Chp_marg	0	1	1	2	1	1	0	0	0	0	0
Coc_cost	0	0	0	0	0	0	0	0	0	0	0
Coc_neot	0	0	2	4	0	3	1	5	0	1	0
Coc_pedi	0	0	3	6	3	5	25	6	4	15	2
Coc_pelt	0	0	0	0	0	0	0	0	0	0	0
Coc_plac	1	0	1	1	1	0	3	0	0	0	0
Coc_pleu	0	0	0	0	0	0	0	0	0	0	0
Coc_plli	0	0	0	0	0	0	0	0	0	0	0
Coc_psli	0	0	0	0	0	0	0	0	0	0	0
Coc_psth	0	0	0	0	0	0	0	0	0	0	0
Coc_scut	0	0	0	0	0	0	0	0	0	0	0
Coc_stau	0	0	0	0	0	0	0	0	0	0	0
Com_pusi	0	0	0	0	0	0	0	0	0	0	0
Cos_aste	0	0	0	0	0	0	0	0	0	0	0
Cte_pulc	6	0	9	21	54	25	66	38	140	63	47
Cyc_atom	0	0	0	0	0	0	0	0	0	0	0
Cyc_choc	0	0	0	3	0	0	0	0	0	0	0
Cyc_mene	0	0	0	0	0	0	0	0	0	0	0
Cyc_radi	0	0	0	2	0	0	0	3	0	0	0
Cyc_ross	0	0	0	6	0	0	0	0	0	0	0
Cyc_stel	0	0	0	0	0	0	0	0	0	0	0
Cyc_stri	0	0	0	0	0	0	0	0	0	0	0
Cyc_trip	0	0	0	0	0	0	0	0	0	0	0
Cym_affi	0	0	0	1	0	0	0	0	0	0	0
Cym_cist	0	0	0	0	0	0	0	0	0	0	0
Cym_helv	0	0	1	0	0	0	0	0	0	1	0
Cym_lanc	0	0	0	0	0	0	0	0	0	0	1
Cym_prox	0	0	0	0	0	0	0	0	0	0	0
Cyp_sole	0	0	0	0	0	0	0	0	0	0	0
Cys_dubi	0	0	0	3	0	0	0	0	0	0	0
Den_tecr	0	0	0	0	0	0	0	0	0	0	0

Dia_bott	0	0	8	5	35	1	6	12	19	15	29
Dia_moni	722	918	68	160	229	335	603	311	266	189	488
Dia_prob	0	0	0	0	0	0	0	0	0	0	0
Dia_tenu	0	0	0	0	0	0	0	1	4	0	0
Dia_vulg	0	2	24	13	30	6	4	4	9	5	12
Dic_subi	0	0	0	0	0	0	0	0	0	0	0
Dip_depa	0	0	0	0	0	0	0	0	0	0	0
Dip_dydi	0	0	0	0	0	0	0	0	0	0	0
Dip_elli	0	0	0	0	0	0	0	0	0	0	0
Dip_inte	0	0	0	0	0	0	0	0	0	0	0
Dip_ocul	0	0	0	0	0	0	0	0	0	0	0
Dip_smdi	0	0	0	0	0	0	0	0	0	0	0
Dip_smpu	0	0	0	0	0	0	0	0	0	0	0
Dip_smrh	0	0	0	0	0	0	0	0	0	0	0
Dip_subo	0	0	0	0	0	0	0	0	0	0	0
Enc_caes	0	0	1	0	1	0	1	0	1	0	0
Enc_lacu	0	0	0	0	0	0	0	0	0	1	0
Enc_minu	0	0	0	0	0	0	0	0	0	0	0
Enc_obsc	0	0	0	0	0	0	0	0	0	0	0
Enc_pros	0	0	0	0	0	0	0	0	0	0	0
Enc_sile	0	0	11	1	0	0	0	0	0	0	0
Enc_tenu	0	0	0	0	0	0	0	0	0	0	0
Enp_behr	0	0	0	0	0	0	0	0	0	0	0
Enp_kram	0	0	0	0	0	0	0	0	0	0	0
Ent_palu	0	0	1	0	0	0	0	2	0	0	0
Ent_pseu	0	0	1	0	0	0	0	1	0	0	0
Ent_punc	0	0	0	0	0	0	0	0	0	0	0
Epi_adna	0	0	0	0	0	2	0	0	0	69	0
Epi_goep	0	0	0	0	0	0	0	0	0	0	0
Epi_sore	0	0	8	7	3	6	1	6	0	170	6
Epi_turg	0	0	0	0	0	0	0	0	0	1	0
Epi_tuwe	0	0	1	1	4	2	1	1	0	3	1
Euc_laev	0	0	0	0	0	0	0	0	2	0	0
Eun_arcu	0	0	0	0	0	0	0	0	0	0	0
Eun_peun	0	0	0	0	0	0	0	0	0	0	0
Fal_clep	0	0	0	0	0	0	0	0	0	0	0
Fal_cryp	0	0	0	0	0	0	0	0	0	0	0
Fal_flor	0	0	0	0	0	0	0	0	0	0	0
Fal_psny	0	0	0	0	0	0	0	0	0	0	0
Fal_pygm	0	0	0	0	0	1	0	0	0	0	0
Fis_sapr	0	0	0	0	0	0	0	0	0	0	0

Fra_amic	0	0	0	0	0	0	0	0	0	0	0
Fra_capu	0	0	0	0	0	0	0	0	0	0	0
Fra_caru	0	0	11	0	4	5	3	5	1	1	1
Fra_cava	0	0	0	0	0	2	1	0	0	2	0
Fra_cons	0	0	0	0	0	0	0	0	0	0	0
Fra_geda	0	0	0	0	0	0	0	0	0	0	0
Fra_grac	0	0	0	0	0	0	0	0	0	0	0
Fra_hydu	0	0	0	0	0	0	0	0	0	0	0
Fra_lept	0	0	0	0	0	0	0	0	0	0	0
Fra_nitz	0	0	0	0	0	0	0	0	0	0	0
Frp_cyli	0	0	6	2	0	2	1	0	2	0	1
Fru_vulg	0	0	0	0	0	0	0	0	0	0	0
Gom_clav	0	0	0	0	0	0	0	0	0	0	0
Gom_intr	0	0	0	0	0	0	0	0	0	0	0
Gom_oliv	2	9	19	15	8	6	9	17	2	2	5
Gom_parv	0	0	0	0	0	0	0	0	0	0	0
Gon_exig	2	0	0	1	2	0	8	0	0	1	3
Gos_expl	0	0	0	0	0	0	0	0	0	0	0
Gos_obsc	0	0	0	0	0	0	0	0	0	0	0
Gra_mari	0	0	0	0	0	0	0	0	0	0	0
Gra_ocea	0	0	0	0	0	0	0	0	0	0	0
Gyr_acum	0	0	0	0	0	0	0	0	0	0	0
Gyr_atte	0	0	0	0	0	0	0	0	0	0	0
Gyr_stri	0	0	0	0	0	0	0	0	0	0	0
Gyr_tenu	0	0	0	0	0	0	0	0	0	0	0
Hal_coff	0	0	0	0	0	0	0	0	0	0	3
Hal_exig	0	0	0	1	0	0	0	0	0	0	0
Hal_hols	0	0	0	0	0	0	0	0	0	0	0
Hal_hybr	0	0	0	0	0	0	0	0	0	0	0
Hal_luci	0	0	2	0	0	0	0	3	1	0	0
Hal_norm	0	0	0	0	0	0	0	0	0	0	0
Hal_subh	0	0	0	0	0	0	0	0	0	0	0
Hal_tene	0	2	10	1	7	8	0	7	3	0	3
Hal_vene	1	0	0	0	0	0	0	0	1	0	0
Han_balt	0	0	1	2	0	0	0	0	0	0	0
Has_spic	0	0	0	0	0	0	0	0	0	0	0
Hip_capi	0	0	0	0	0	0	0	0	0	0	0
Hip_hung	0	0	0	0	0	0	0	0	0	0	0
Hip_lesm	0	0	0	0	0	0	0	0	0	0	0
Kar_amoe	0	0	0	0	0	0	0	0	0	1	0
Kar_clev	0	0	0	0	0	0	0	0	0	0	0

Kar_niti	0	0	0	0	0	0	0	0	0	0	0
Kar_subm	0	0	0	0	0	0	0	0	0	0	0
Lemn_hung	0	0	0	0	0	0	0	0	0	0	0
Lic_debi	0	0	0	0	0	0	0	0	0	0	0
Lic_gran	12	3	3	4	9	9	38	1	3	4	13
Lic_oedi	0	0	0	0	0	0	0	0	0	0	0
Lic_rhom	0	0	0	0	0	0	0	0	0	0	0
Lun_bise	3	1	27	24	22	24	8	18	37	8	11
Lut_muti	0	0	0	0	0	0	0	0	0	0	0
Mar_atom	1	0	0	0	2	2	1	0	0	0	1
Mar_mart	0	0	2	2	0	10	1	1	0	3	0
Mar_schu	0	0	0	0	0	0	0	0	0	3	0
Mas_balt	0	0	0	0	0	0	0	0	0	0	0
Mas_brau	0	0	0	0	0	0	0	0	0	0	0
Mas_elli	0	0	1	0	0	0	0	1	2	1	0
Mas_exig	0	0	0	0	0	0	0	0	0	0	0
Mas_pumi	0	0	0	0	0	0	0	0	0	0	0
Mas_pusi	0	0	0	0	0	1	0	0	0	0	0
Mas_smam	0	0	6	0	1	2	6	0	0	3	1
Mas_smit	0	0	1	2	0	0	3	1	0	2	8
Mel_arct	0	0	0	0	0	0	0	0	4	0	0
Mel_line	0	0	1	0	5	0	1	13	5	1	1
Mel_moni	0	0	0	0	0	0	0	2	0	4	0
Mel_numm	0	0	0	0	0	0	0	2	0	0	0
Mel_vari	0	0	0	0	0	0	0	0	5	0	0
Nav_aren	0	0	0	0	0	0	0	0	0	0	0
Nav_bipu	0	0	0	0	0	0	0	0	0	1	0
Nav_boss	0	0	0	0	0	0	0	0	0	0	0
Nav_bott	2	1	1	5	9	0	0	0	0	0	0
Nav_brem	0	0	0	0	0	0	0	0	0	0	0
Nav_capi	0	0	0	0	0	0	0	0	0	0	0
Nav_cinc	0	0	0	0	0	0	0	0	0	0	0
Nav_clem	0	0	0	0	0	0	0	0	0	0	0
Nav_cryp	0	0	0	0	0	0	0	0	0	0	0
Nav_digi	0	0	0	0	0	0	0	0	0	0	0
Nav_duer	0	0	0	0	0	0	0	0	0	0	0
Nav_eidr	0	0	0	0	0	0	0	0	0	0	0
Nav_flan	0	0	0	0	0	0	0	0	0	0	0
Nav_geii	0	0	0	0	0	0	0	0	0	0	1
Nav_gepo	0	0	0	2	3	5	0	3	6	2	0
Nav_greg	8	2	21	18	26	4	1	23	8	3	0

Nav_haca	0	0	0	0	0	0	0	0	0	0	0
Nav_hani	0	0	0	0	0	0	0	0	0	0	0
Nav_hust	0	0	0	0	0	0	0	0	0	0	0
Nav_lanc	0	1	0	27	8	0	0	4	4	3	0
Nav_late	0	0	0	0	0	0	0	0	0	0	0
Nav_marg	0	0	0	0	0	0	0	0	0	0	0
Nav_meni	0	0	0	1	0	0	0	0	0	0	0
Nav_midi	0	0	0	0	0	0	0	0	0	0	0
Nav_nole	0	0	0	0	0	0	0	0	0	0	0
Nav_oblo	0	0	0	0	0	0	0	0	0	0	0
Nav_oest	0	0	0	0	0	0	0	0	0	0	0
Nav_pere	0	0	0	0	0	0	0	0	0	0	0
Nav_perm	52	3	163	215	73	69	23	80	26	24	22
Nav_phyl	1	0	0	9	2	11	0	10	4	3	1
Nav_port	0	0	0	1	0	0	0	0	0	0	0
Nav_radi	0	0	0	0	0	0	0	0	0	0	0
Nav_ramo	0	0	0	0	0	0	0	0	0	0	0
Nav_rato	0	0	0	0	0	0	0	0	0	0	0
Nav_rece	0	0	0	0	0	0	0	0	0	0	0
Nav_rhyn	0	0	0	2	0	2	2	6	6	9	1
Nav_sana	0	0	0	0	0	0	0	0	0	0	0
Nav_sjoe	14	16	16	16	18	15	2	8	0	1	5
Nav_star	0	0	0	0	0	0	0	0	0	0	0
Nav_subr	0	0	0	0	0	0	0	0	0	0	0
Nav_supr	0	0	0	0	0	0	0	0	0	0	0
Nav_trip	0	0	0	0	0	0	0	0	0	0	0
Nav_vene	0	0	0	1	0	0	0	0	0	1	0
Ncy_pusi	0	0	0	0	0	0	0	0	0	0	0
Nit_acic	0	0	0	0	0	0	0	0	0	0	0
Nit_anfo	5	0	1	0	0	0	0	0	0	0	0
Nit_anla	0	0	0	0	0	0	0	0	0	0	0
Nit_arch	0	0	0	0	0	0	0	0	0	0	0
Nit_aura	0	0	47	2	10	11	1	0	1	0	1
Nit_baci	0	0	0	0	0	0	0	0	0	0	0
Nit_dime	0	0	0	0	1	0	0	0	0	0	0
Nit_dipp	0	0	4	2	0	0	0	1	0	0	0
Nit_diss	0	0	0	0	0	0	0	0	0	0	0
Nit_dist	0	0	0	0	0	0	0	0	0	0	0
Nit_dubi	0	0	0	0	0	0	0	0	0	0	0
Nit_eleg	0	0	0	0	0	0	0	0	0	0	0
Nit_frig	0	0	0	0	0	0	0	0	2	0	0

Nit_frus	0	0	20	5	9	17	6	17	23	5	3
Nit_gros	0	0	2	0	0	0	0	0	0	0	0
Nit_heuf	0	0	3	0	1	0	0	1	0	0	0
Nit_inco	74	11	94	180	152	130	54	107	104	83	125
Nit_inte	0	0	0	0	0	0	0	0	0	0	0
Nit_lieb	0	2	1	1	12	6	1	21	3	1	14
Nit_lisu	0	0	43	0	3	7	1	0	0	1	0
Nit_lore	0	0	0	0	0	0	0	0	0	0	0
Nit_micr	0	0	44	11	5	23	8	11	9	1	5
Nit_pale	0	0	0	0	1	0	0	0	0	0	0
Nit_pcea	0	0	0	2	1	6	0	4	0	0	0
Nit_pell	0	0	0	0	0	0	0	0	0	0	0
Nit_perm	0	0	0	0	0	0	0	0	0	0	0
Nit_pusi	0	0	13	11	14	8	3	2	0	0	12
Nit_rect	0	0	0	0	0	0	0	0	0	0	0
Nit_rose	0	0	151	4	22	37	6	3	2	2	22
Nit_saco	0	0	0	0	0	0	0	0	0	2	0
Nit_sdea	0	0	0	0	0	0	0	0	0	0	0
Nit_sigm	0	0	0	0	1	1	0	2	0	0	1
Nit_soci	0	0	0	0	0	0	0	0	3	0	0
Nit_soli	0	0	0	0	0	0	0	0	0	0	0
Nit_subl	0	0	0	0	0	0	0	0	0	0	0
Nit_ther	0	0	2	11	9	6	1	3	1	0	4
Nit_umbo	0	0	0	0	0	0	0	0	0	0	0
Nit_vald	0	0	1	1	2	0	0	2	0	1	0
Nit_verm	0	0	0	0	0	0	0	0	0	0	0
Nit_vitr	0	0	0	0	0	0	0	1	0	0	0
Nit_wuel	0	0	0	0	0	0	0	0	0	0	0
Ope_krum	0	0	0	0	0	0	0	0	0	0	0
Ope_muta	0	0	0	0	0	0	0	0	0	2	0
Par_prot	0	0	0	0	0	0	0	0	0	0	0
Pau_taen	1	0	6	3	4	5	0	1	0	0	0
Pet_hume	0	0	0	0	0	0	0	0	0	0	0
Pin_appe	0	0	0	1	0	0	0	0	1	0	0
Pin_dimi	0	0	0	0	0	0	0	0	0	0	0
Pin_kroc	0	0	0	0	0	0	0	0	0	0	0
Pin_micr	0	0	0	0	0	0	0	0	0	0	0
Plc_plac	0	0	0	0	0	0	0	0	0	0	0
Plt_cons	0	0	0	0	0	0	0	0	0	0	0
Plt_deli	0	0	7	6	1	7	3	15	1	5	4
Plt_disp	0	0	0	0	0	0	0	0	0	0	0

Plt_freq	0	0	0	0	0	0	0	0	1	0	0
Plt_hauc	0	0	0	14	0	4	2	7	6	2	0
Plt_lanc	0	0	0	0	0	0	0	0	0	8	0
Plt_lemm	0	0	0	3	0	0	0	0	0	1	0
Plt_link	0	0	0	0	0	0	0	0	0	0	0
Plt_peri	1	0	0	0	0	0	0	0	0	0	0
Plt_rost	0	0	0	0	0	0	0	0	0	0	0
Plt_sept	0	0	0	1	2	0	0	0	0	0	0
Psf_tene	0	0	0	0	0	0	0	0	0	0	0
Pss_brev	0	0	0	0	0	0	0	0	0	0	0
Pss_brin	0	0	0	0	0	0	0	0	0	0	0
Pss_elli	0	0	0	0	1	6	1	1	2	2	0
Pss_perm	0	0	0	0	0	5	0	0	0	0	0
Pss_robu	0	0	0	0	0	0	0	0	0	0	0
Pss_subs	0	0	0	0	0	0	0	0	0	0	0
Pss_zeil	0	0	0	0	1	0	0	0	3	0	0
Pst_bior	0	0	0	0	0	0	0	0	0	0	0
Pst_oblo	0	0	0	0	0	0	0	0	0	0	0
Pst_punc	0	0	0	0	0	0	1	1	0	1	1
Pst_ross	0	0	0	0	0	0	0	0	0	1	0
Pst_sacc	0	0	0	0	0	0	0	0	0	0	0
Pte_inan	0	0	0	0	0	0	0	0	0	0	0
Rei_sinu	0	0	0	0	0	0	0	0	0	0	0
Rho_abbr	10	5	21	33	32	37	28	18	3	188	27
Rhp_acum	0	0	0	0	0	0	0	0	0	0	0
Rhp_breb	0	0	0	4	0	0	0	0	0	0	0
Rhp_gibb	0	0	0	0	0	0	0	2	2	0	0
Sel_pupu	0	0	0	0	0	0	0	0	0	0	0
Sel_stro	0	0	0	0	0	0	0	0	0	0	0
Ske_cost	0	0	0	0	0	0	0	0	0	0	0
Ske_subs	0	0	0	0	0	0	0	0	0	0	0
Sla_lapp	0	0	0	0	0	0	0	0	0	0	0
Std_hant	0	0	0	0	0	0	0	1	0	0	0
Std_parv	0	0	0	1	0	0	0	0	0	0	0
Stn_legu	0	0	0	0	0	0	0	0	0	0	0
Stn_phoe	0	0	0	0	0	0	0	0	0	0	0
Stn_simu	0	0	0	0	0	0	0	0	0	0	0
Stn_smka	0	0	0	0	0	0	0	0	0	0	0
Stp_anus	0	0	0	0	0	0	0	0	0	0	0
Stp_wisl	0	0	0	0	0	0	0	0	0	0	0
Sts_punc	0	0	0	0	0	0	0	0	0	0	0

Sts_vent	0	0	0	0	0	0	0	0	0	0	0
Sur_angu	0	0	0	0	0	0	0	0	0	0	0
Sur_brba	0	0	0	0	0	0	0	0	0	1	0
Sur_breb	0	0	7	6	8	2	0	9	1	1	8
Sur_minu	0	0	0	0	0	0	0	0	0	0	0
Sur_oval	0	0	0	0	0	0	0	0	0	0	0
Syn_acus	0	0	0	0	0	0	0	0	0	0	0
Syn_ulna	0	0	0	0	0	0	0	0	0	0	0
Tab_fasc	27	2	13	9	44	9	24	45	202	28	15
Tab_kten	0	0	0	0	5	0	0	0	0	0	0
Tab_laev	0	0	0	0	0	0	0	0	0	0	0
Tab_tabu	1	0	1	1	1	1	1	4	3	4	1
Tab_waer	0	0	2	19	2	10	2	2	3	1	3
Tal_fene	0	0	0	0	0	0	0	0	0	0	0
Tal_floc	0	0	0	2	0	0	0	0	0	0	0
Tet_glan	0	0	0	0	0	0	0	0	0	1	0
Tha_balt	0	2	0	0	1	0	1	3	3	1	0
Tha_hyla	0	0	0	0	0	1	0	0	0	1	1
Tha_leva	0	0	0	1	1	4	2	1	0	0	4
Tha_pros	0	0	3	0	3	0	0	0	0	0	0
Tha_pseu	0	0	0	0	2	0	1	2	0	0	0
Tha_punc	0	0	0	0	0	0	0	0	0	0	0
Try_angu	0	0	0	0	0	0	0	0	0	0	0
Try_apic	0	0	0	0	0	0	0	0	0	0	0
Try_ardu	0	0	0	0	0	0	0	0	0	0	0
Try_hung	0	0	0	0	0	0	0	0	0	0	0
Try_levi	0	0	0	1	0	0	0	0	0	0	0
Try_lite	0	0	0	0	0	0	0	0	0	0	0
Try_sali	0	0	0	0	0	0	0	0	0	0	0
Try_scal	0	0	0	0	0	0	0	0	0	0	0

AREA	Hol	Hol	Hol	Hol	Hol	Hol	Hol	Hol	Hol	Hol	Hol	Hol	Hol	Hol
SITE	147	148	149	150	151	152	153	154	155	156	157	158	159	160
Acd_exil	0	0	0	0	0	0	0	0	0	0	0	0	0	0
Acd_minu	48	43	29	10	30	82	37	110	252	194	9	6	17	6
Acd_pyre	0	0	0	0	0	0	0	0	0	0	0	0	0	0
Ach_brev	0	0	0	0	0	0	0	0	0	0	0	0	0	0
Ach_brin	0	0	0	0	0	0	0	0	0	0	0	0	0	0
Ach_foge	0	0	0	0	0	0	0	0	0	0	0	0	0	0
Ach_leon	0	0	0	0	0	0	0	0	0	0	0	0	0	0
Ach_long	0	0	0	0	0	0	0	0	0	0	0	0	0	0
Ach_luth	0	1	0	0	0	0	0	0	0	0	0	0	0	0
Ach_misc	0	0	0	0	0	0	0	0	0	0	0	0	0	0
Ach_vist	0	0	0	0	0	0	0	0	0	0	0	0	0	0
Act_nosu	0	0	0	0	0	0	0	0	0	0	0	0	0	0
Act_occr	0	0	0	0	0	0	0	0	0	0	0	0	0	0
Ami_spec	0	0	0	0	0	0	0	0	0	0	0	0	0	0
Amp_copu	2	2	1	1	2	1	0	3	0	1	0	1	0	0
Amp_fleb	0	0	0	0	0	0	0	0	0	0	0	0	0	0
Amp_hele	0	0	0	0	0	0	0	0	0	0	0	0	0	0
Amp_inar	0	0	0	0	0	0	0	0	0	0	0	0	0	0
Amp_line	0	0	0	0	0	0	0	1	0	0	0	0	0	0
Amp_micr	0	0	0	0	1	0	0	0	0	0	0	0	0	0
Amp_oval	0	0	0	0	1	0	0	0	0	0	0	0	0	0
Amp_pedi	2	4	12	0	21	7	40	7	16	29	0	0	9	5
Amp_prof	0	0	0	0	0	0	0	0	0	0	0	0	0	0
Ane_mino	0	0	1	0	0	0	1	0	0	1	0	0	1	0
Ane_tusc	0	0	0	0	0	0	0	1	0	0	0	0	0	0
Ano_spha	0	0	0	0	0	0	0	0	0	0	0	0	0	0
Asa_bahu	0	0	0	0	0	0	0	0	0	0	0	0	0	0
Ast_form	0	0	0	0	0	0	0	0	0	0	0	0	0	0
Aul_spp	0	0	0	0	0	0	0	0	0	0	0	0	0	0
Bac_paxi	0	0	0	0	0	0	0	0	0	0	0	0	0	0
Ber_fenn	3	10	1	2	43	0	19	0	13	7	0	0	1	0
Ber_ruti	0	0	0	0	0	0	0	0	5	0	0	0	0	0
Ber_scop	0	0	0	0	0	0	0	0	0	0	0	0	0	0
Bir_luce	0	0	0	0	0	0	0	0	0	0	0	0	0	0
Bra_apon	1	0	5	0	25	1	2	0	3	10	0	0	0	0
Bra_vitr	1	0	0	0	1	0	0	0	0	0	0	0	0	0
Bre_lanc	0	0	0	0	0	0	0	0	0	0	0	0	0	0
Cal_aemu	0	0	0	0	0	0	0	0	0	0	0	0	0	0

Cal_amph	0	0	0	0	0	0	0	0	0	0	0	0	0	0
Cal_baci	0	0	0	1	0	0	1	0	0	0	0	0	0	0
Cal_cras	0	0	0	0	0	0	0	0	0	0	0	0	0	0
Cal_sili	0	0	0	0	0	0	0	0	0	0	0	0	0	0
Cam_clyp	0	0	0	0	0	0	0	0	0	0	0	0	0	0
Cat_adha	0	0	0	0	0	0	0	0	0	0	0	0	0	0
Cav_lapi	0	0	0	0	0	0	0	0	0	0	0	0	0	0
Cav_pseu	0	0	0	0	0	0	0	0	0	0	0	0	0	0
Cer_clos	0	0	0	0	0	0	0	0	0	0	0	0	0	0
Chp_clam	0	0	0	0	0	0	0	0	0	0	0	0	0	0
Chp_marg	0	0	0	0	1	1	0	0	0	0	0	1	0	0
Coc_cost	0	0	0	0	0	0	0	0	0	0	0	0	0	0
Coc_neot	2	3	2	1	4	2	14	9	13	10	0	0	1	0
Coc_pedi	8	1	3	3	1	6	7	1	0	2	1	3	3	3
Coc_pelt	0	0	0	0	0	0	0	0	0	0	0	0	0	0
Coc_plac	0	2	1	1	2	4	11	2	1	7	0	0	2	0
Coc_pleu	0	0	0	0	0	0	0	0	0	0	0	0	0	0
Coc_plli	0	0	0	0	0	0	0	0	0	0	0	0	0	0
Coc_psli	0	0	0	0	0	0	0	0	0	0	0	0	0	0
Coc_psth	0	0	0	0	0	0	0	0	0	0	0	0	0	0
Coc_scut	0	0	0	0	0	0	0	0	0	0	0	0	0	0
Coc_stau	0	0	0	0	0	0	0	0	0	0	0	0	0	0
Com_pusi	0	0	0	0	0	0	0	0	0	0	0	0	0	0
Cos_aste	0	0	0	0	0	0	0	0	0	0	0	0	0	0
Cte_pulc	8	51	11	5	38	19	18	5	15	13	0	14	12	6
Cyc_atom	0	0	0	0	0	0	0	0	0	0	0	0	0	0
Cyc_choc	0	0	0	0	0	0	0	0	0	0	0	0	0	0
Cyc_mene	0	0	0	0	0	0	1	4	0	0	0	0	0	0
Cyc_radi	0	0	0	0	0	0	0	0	0	0	0	0	0	0
Cyc_ross	0	0	0	0	0	0	0	0	1	1	0	0	0	0
Cyc_stel	0	0	0	0	0	0	0	0	0	0	0	0	0	0
Cyc_stri	0	0	0	0	0	0	0	0	0	0	0	0	0	0
Cyc_trip	0	0	0	0	0	0	0	0	0	0	0	0	0	0
Cym_affi	15	7	2	1	9	5	8	0	2	3	0	0	5	1
Cym_cist	0	0	0	0	1	0	0	0	0	0	0	0	0	0
Cym_helv	11	26	4	9	10	2	8	1	2	0	11	47	4	5
Cym_lanc	2	3	0	0	0	1	0	0	0	0	0	1	0	1
Cym_prox	0	0	0	0	0	0	0	0	0	0	0	0	0	0
Cyp_sole	0	0	0	0	0	0	0	0	0	0	0	0	0	0
Cys_dubi	0	0	1	0	0	0	0	0	0	0	2	0	0	0
Den_tecr	13	20	27	2	33	35	35	0	5	4	0	1	1	0

Dia_bott	4	2	4	3	2	5	0	1	0	1	12	3	6	4
Dia_moni	717	530	692	790	450	575	179	324	209	199	878	440	815	713
Dia_prob	0	0	0	0	0	0	1	0	0	0	0	0	0	0
Dia_tenu	0	0	0	0	0	1	0	0	0	0	0	0	0	0
Dia_vulg	0	0	0	0	0	0	0	0	0	0	0	0	0	0
Dic_subi	0	0	0	0	0	0	0	0	0	0	0	0	0	0
Dip_depa	0	0	0	0	0	0	0	0	0	0	0	0	0	0
Dip_dydi	0	0	0	0	0	0	0	0	0	0	0	0	0	0
Dip_elli	0	0	0	0	0	0	0	0	0	0	0	0	0	0
Dip_inte	0	0	0	0	0	0	0	0	0	0	0	0	0	0
Dip_ocul	0	0	0	0	0	0	0	0	0	0	0	0	0	0
Dip_smdi	0	0	0	0	0	0	0	0	0	0	0	0	0	0
Dip_smpu	0	0	0	0	0	0	0	1	0	0	0	0	0	0
Dip_smrh	0	0	0	0	0	0	0	0	0	0	0	0	0	0
Dip_subo	0	0	0	0	0	0	0	0	0	0	0	0	0	0
Enc_caes	1	3	0	0	3	0	1	0	2	2	0	0	0	0
Enc_lacu	0	0	0	0	1	0	0	0	0	1	1	0	0	0
Enc_minu	0	0	0	0	0	0	0	0	0	0	0	0	0	0
Enc_obsc	0	0	0	0	0	0	0	0	0	0	0	0	0	0
Enc_pros	0	0	0	0	0	0	0	0	2	0	0	0	0	0
Enc_sile	0	0	0	0	0	0	0	0	1	0	0	0	0	0
Enc_tenu	0	0	0	0	0	0	1	0	0	0	0	0	0	0
Enp_behr	0	0	1	0	0	0	0	0	0	0	0	0	0	0
Enp_kram	2	1	1	0	9	4	1	1	0	0	0	0	0	1
Ent_palu	0	0	0	1	0	1	1	1	1	1	0	0	1	0
Ent_pseu	0	0	0	0	0	0	0	0	0	0	0	0	0	0
Ent_punc	0	0	0	0	0	0	0	0	0	0	0	0	0	0
Epi_adna	0	0	0	0	0	0	0	1	0	0	0	0	0	0
Epi_goep	0	0	0	0	0	0	0	0	0	0	0	0	0	0
Epi_sore	0	0	1	0	0	1	1	5	0	6	0	0	1	0
Epi_turg	2	1	1	0	0	3	0	1	0	1	0	0	1	0
Epi_tuwe	0	0	0	0	0	0	0	0	0	0	0	0	0	0
Euc_laev	0	0	0	0	0	0	0	1	0	0	0	0	0	0
Eun_arcu	0	0	0	0	0	0	0	3	0	0	0	0	0	0
Eun_peun	0	0	0	0	0	0	0	0	0	0	0	0	0	0
Fal_clep	0	0	0	0	0	1	0	0	0	0	0	0	0	0
Fal_cryp	0	0	0	0	0	0	0	0	0	0	0	0	0	0
Fal_flor	0	0	0	0	0	0	0	0	0	0	0	0	0	0
Fal_psny	0	0	0	0	0	0	0	0	0	0	0	0	0	0
Fal_pygm	0	0	0	0	0	0	1	1	0	0	0	0	0	0
Fis_sapr	0	0	0	0	0	0	0	0	0	0	0	0	0	0

Fra_amic	0	0	0	0	0	0	0	0	0	0	0	0	0	0
Fra_capu	0	0	0	0	0	0	0	0	0	0	0	0	6	1
Fra_caru	23	59	29	17	44	30	12	60	87	36	4	2	13	3
Fra_cava	16	72	43	36	56	46	17	3	32	43	15	29	17	11
Fra_cons	0	0	0	0	0	0	0	0	0	0	0	0	0	0
Fra_geda	0	0	0	0	0	0	0	0	0	0	0	0	0	0
Fra_grac	0	0	0	0	9	2	6	52	4	0	0	0	0	0
Fra_hydu	0	0	0	0	0	0	0	0	0	0	0	0	0	0
Fra_lept	0	0	0	0	0	0	0	0	0	0	0	0	0	0
Fra_nitz	0	0	0	0	0	0	0	0	0	3	0	0	0	0
Frp_cyli	1	2	2	1	4	2	0	0	2	2	3	0	1	4
Fru_vulg	0	0	0	0	0	0	0	0	0	0	0	0	0	0
Gom_clav	0	0	0	0	0	0	0	0	0	0	0	0	0	0
Gom_intr	2	2	2	2	5	2	1	3	1	6	8	0	1	27
Gom_oliv	18	20	13	9	6	8	5	0	9	37	23	30	5	4
Gom_parv	0	0	0	0	0	0	0	0	0	0	0	0	0	1
Gon_exig	0	0	0	0	0	0	0	0	0	0	0	0	0	0
Gos_expl	0	0	0	0	0	0	0	0	0	0	0	0	0	0
Gos_obsc	0	0	0	0	0	0	0	0	0	0	0	0	0	0
Gra_mari	0	0	0	0	0	0	0	0	0	0	0	0	0	0
Gra_ocea	0	0	0	0	0	0	0	0	0	0	0	0	0	0
Gyr_acum	0	0	0	0	0	0	0	0	0	0	0	0	0	0
Gyr_atte	0	0	0	0	0	0	0	0	0	0	0	0	0	0
Gyr_stri	0	0	0	0	0	0	0	0	0	0	0	0	0	0
Gyr_tenu	0	0	0	0	0	0	0	0	0	0	0	0	0	0
Hal_coff	0	0	0	0	0	0	4	1	2	4	0	0	0	0
Hal_exig	0	0	0	0	0	0	1	0	0	0	0	0	0	0
Hal_hols	0	0	0	0	0	0	0	0	0	0	0	0	1	0
Hal_hybr	0	0	0	0	0	0	0	1	0	0	0	0	2	0
Hal_luci	0	0	0	0	0	0	1	0	0	0	0	0	0	0
Hal_norm	0	0	0	0	0	0	0	0	0	0	0	0	0	0
Hal_subh	0	0	0	0	0	0	0	0	0	0	0	0	0	0
Hal_tene	0	0	3	1	0	0	18	3	0	0	0	0	0	0
Hal_vene	1	3	1	0	3	2	5	8	1	0	0	0	0	1
Han_balt	0	0	0	0	0	0	0	1	0	0	0	0	0	0
Has_spic	0	0	0	0	0	0	0	0	2	0	0	0	0	0
Hip_capi	0	0	0	0	0	0	2	1	0	0	0	0	0	0
Hip_hung	0	0	0	0	0	0	0	0	0	0	0	0	0	0
Hip_lesm	0	0	0	0	0	0	0	0	0	0	0	0	0	0
Kar_amoe	0	0	0	0	0	0	0	0	0	0	0	0	0	0
Kar_clev	0	0	1	0	0	0	0	0	2	3	0	0	1	0

Kar_niti	0	0	0	0	0	0	0	0	0	0	0	0	0	0
Kar_subm	0	0	0	0	0	0	0	0	0	0	0	0	0	0
Lemn_hung	0	1	0	0	0	0	0	1	0	0	0	0	0	0
Lic_debi	0	0	0	0	0	0	0	0	0	0	0	0	0	0
Lic_gran	0	0	0	0	0	0	0	0	0	0	0	0	0	0
Lic_oedi	0	0	0	0	0	0	0	0	0	0	0	0	0	0
Lic_rhom	0	0	0	0	0	0	0	0	0	0	0	0	0	0
Lun_bise	0	4	2	1	1	1	10	1	8	0	0	0	0	6
Lut_muti	0	0	0	0	0	0	0	0	0	0	0	0	0	0
Mar_atom	0	0	0	0	0	0	41	12	25	30	0	0	0	0
Mar_mart	0	0	2	0	6	0	44	14	8	32	0	0	5	0
Mar_schu	0	0	1	0	7	1	33	89	22	7	1	0	2	0
Mas_balt	0	0	0	0	0	0	0	0	0	0	0	0	0	0
Mas_brau	0	0	0	0	0	0	0	0	0	0	0	0	0	0
Mas_elli	0	0	0	0	1	0	0	0	0	0	0	0	0	0
Mas_exig	0	0	0	0	0	0	0	0	0	0	0	0	0	0
Mas_pumi	0	0	0	0	0	0	0	0	0	0	0	0	0	0
Mas_pusi	0	0	0	0	0	0	0	0	0	0	0	0	0	0
Mas_smam	1	0	0	0	4	0	0	2	0	2	0	0	0	0
Mas_smit	0	0	2	0	0	1	1	0	1	1	0	0	1	0
Mel_arct	0	0	0	0	0	0	0	0	0	0	0	0	1	0
Mel_line	0	0	0	0	0	0	0	0	0	0	0	0	0	0
Mel_moni	0	0	0	0	0	0	0	0	0	0	0	0	0	0
Mel_numm	0	0	0	0	0	0	0	0	0	0	0	0	0	0
Mel_vari	0	0	0	0	0	0	0	0	0	0	0	0	0	0
Nav_aren	0	0	0	0	0	0	0	0	0	0	0	0	0	0
Nav_bipu	0	0	0	0	0	0	0	0	0	0	0	0	0	0
Nav_boss	0	0	0	0	0	0	0	0	0	0	0	0	0	0
Nav_bott	1	1	1	0	0	0	2	0	0	0	0	1	0	1
Nav_brem	0	0	0	0	0	0	0	0	0	0	0	0	0	0
Nav_capi	0	0	0	0	0	0	0	0	0	0	0	0	0	0
Nav_cinc	0	0	0	0	0	0	0	0	0	1	0	0	0	0
Nav_clem	0	0	0	0	0	0	2	0	0	0	0	0	1	0
Nav_cryp	2	3	0	0	7	1	2	2	7	1	0	0	0	0
Nav_digi	0	0	0	0	0	0	0	0	0	0	0	0	0	0
Nav_duer	0	0	0	0	0	0	0	0	0	0	0	0	0	0
Nav_eidr	0	0	0	0	0	0	0	0	0	0	0	0	0	0
Nav_flan	0	0	0	0	0	0	0	0	1	0	0	0	0	0
Nav_geii	0	0	0	0	0	0	0	0	0	0	0	0	0	0
Nav_gepo	0	0	0	0	0	0	4	0	0	0	0	0	0	0
Nav_greg	0	3	1	1	2	3	26	12	7	6	0	0	3	0

Nav_haca	0	0	0	0	0	0	0	0	0	0	0	0	0	0
Nav_hani	0	0	0	0	0	0	0	0	0	0	0	0	0	0
Nav_hust	0	0	0	0	0	0	0	0	0	0	0	0	0	0
Nav_lanc	0	0	0	0	0	0	0	0	0	0	0	0	0	0
Nav_late	0	0	1	0	0	0	0	0	0	0	0	0	0	0
Nav_marg	0	0	0	0	0	0	0	0	0	0	0	0	0	0
Nav_meni	0	0	0	0	0	0	0	0	0	0	1	0	0	0
Nav_midi	0	0	0	0	0	0	0	0	0	0	0	0	0	0
Nav_nole	0	0	0	0	0	0	0	0	0	0	0	0	0	0
Nav_oblo	0	0	0	0	0	0	0	0	0	0	0	0	0	0
Nav_oest	0	0	0	0	0	0	0	0	0	0	0	0	0	0
Nav_pere	0	0	0	0	0	0	0	0	0	0	0	0	0	0
Nav_perm	1	0	0	0	0	0	1	2	1	2	0	0	0	0
Nav_phyl	0	1	0	1	0	1	9	3	3	1	0	0	1	0
Nav_port	0	0	0	0	0	0	0	0	0	0	0	0	0	0
Nav_radi	0	0	0	0	0	0	0	0	0	0	0	0	0	0
Nav_ramo	0	0	0	0	0	0	0	0	0	0	0	0	0	0
Nav_rato	0	0	0	0	0	0	0	0	0	0	0	0	0	0
Nav_rece	0	0	0	0	0	0	0	0	0	0	0	0	0	0
Nav_rhyn	1	1	1	0	0	1	6	10	0	0	0	0	0	0
Nav_sana	0	0	0	0	0	0	8	0	1	0	0	0	0	0
Nav_sjoe	0	0	0	0	0	0	0	0	0	0	0	0	0	0
Nav_star	0	0	0	0	0	0	0	0	0	0	0	0	0	0
Nav_subr	0	0	0	0	0	0	0	0	0	0	0	0	0	0
Nav_supr	0	0	0	0	0	0	0	0	0	0	0	0	0	0
Nav_trip	0	0	0	0	0	0	0	0	0	0	0	0	0	0
Nav_vene	0	1	0	0	1	1	2	2	0	0	0	0	0	0
Ncy_pusi	0	0	0	0	0	1	0	1	0	0	0	0	0	0
Nit_acic	0	0	0	0	0	0	0	0	0	0	0	0	0	0
Nit_anfo	0	0	0	0	0	0	0	0	0	0	0	0	1	0
Nit_anla	0	0	0	0	0	0	0	0	0	0	0	0	0	0
Nit_arch	0	0	0	0	0	0	0	0	0	0	0	0	0	0
Nit_aura	0	14	10	3	6	7	0	0	3	4	0	6	2	3
Nit_baci	0	0	0	0	0	0	0	0	0	0	0	0	0	0
Nit_dime	0	0	0	0	0	0	0	0	0	0	0	0	0	0
Nit_dipp	0	0	0	0	0	0	0	0	0	2	0	0	0	0
Nit_diss	0	0	0	0	0	0	0	0	0	0	0	0	0	0
Nit_dist	0	0	0	0	0	0	0	0	0	0	0	0	0	0
Nit_dubi	0	0	0	0	0	0	0	0	0	0	0	0	0	0
Nit_eleg	0	0	0	0	0	0	0	0	0	0	0	0	0	0
Nit_frig	0	0	0	0	0	0	0	0	0	0	0	0	0	0

Nit_frus	1	0	6	1	9	3	6	1	22	9	1	10	5	4
Nit_gros	0	0	0	0	0	0	0	0	0	0	0	0	0	0
Nit_heuf	0	0	1	1	2	2	0	0	0	0	0	0	0	0
Nit_inco	7	6	16	17	12	6	100	1	56	8	9	69	10	69
Nit_inte	0	0	0	0	0	0	0	0	0	0	0	0	0	0
Nit_lieb	0	18	6	6	28	2	11	1	8	18	1	31	1	84
Nit_lisu	3	7	10	6	11	11	19	0	3	0	0	0	0	0
Nit_lore	0	0	0	0	0	0	0	0	0	0	0	0	0	0
Nit_micr	0	0	1	0	2	0	18	3	4	2	0	0	0	0
Nit_pale	0	1	0	1	0	0	0	0	2	0	0	0	0	0
Nit_pcea	0	3	0	2	0	0	3	0	2	1	1	0	0	0
Nit_pell	0	0	0	0	0	0	0	0	0	0	0	0	0	0
Nit_perm	0	0	0	0	0	0	2	0	3	0	0	0	0	0
Nit_pusi	2	2	2	2	8	2	6	0	1	0	0	0	0	0
Nit_rect	0	0	0	0	0	0	0	0	0	0	0	0	0	0
Nit_rose	1	1	5	4	21	6	9	3	12	12	6	185	3	21
Nit_saco	0	0	0	0	0	0	0	0	0	0	0	0	0	0
Nit_sdea	0	0	0	0	0	0	0	0	0	0	0	0	0	0
Nit_sigm	0	0	0	0	0	0	0	0	1	0	0	0	0	0
Nit_soci	0	0	0	0	0	0	0	7	0	0	0	1	0	0
Nit_soli	0	7	2	7	1	8	0	0	0	0	0	1	1	0
Nit_subl	0	0	0	0	0	0	0	0	0	0	0	0	0	0
Nit_ther	0	2	0	0	0	2	1	0	2	1	0	0	0	1
Nit_umbo	0	0	0	0	0	0	0	0	0	0	0	0	0	0
Nit_vald	1	2	1	0	1	1	3	7	8	11	0	0	0	0
Nit_verm	0	0	0	0	0	0	0	0	0	0	0	0	0	0
Nit_vitr	1	0	2	2	4	7	2	1	0	1	0	1	0	3
Nit_wuel	0	0	0	0	0	0	0	0	0	0	0	0	0	0
Ope_krum	0	0	0	0	0	0	0	0	0	0	0	0	0	0
Ope_muta	0	0	0	0	0	0	0	0	0	0	0	0	0	0
Par_prot	0	0	1	0	0	0	2	1	0	0	0	0	1	0
Pau_taen	0	1	1	0	0	0	0	0	0	0	0	1	0	0
Pet_hume	0	0	0	0	0	0	0	0	0	0	0	0	0	0
Pin_appe	0	1	0	0	0	0	0	0	0	0	0	0	0	0
Pin_dimi	0	0	0	0	0	0	0	0	0	0	0	0	0	0
Pin_kroc	0	0	0	0	0	0	0	0	0	0	0	0	0	0
Pin_micr	0	0	0	0	0	0	0	4	0	0	0	0	0	0
Plc_plac	0	0	0	0	0	0	0	0	0	0	0	0	0	0
Plt_cons	0	0	0	0	0	0	0	0	0	0	0	0	0	0
Plt_deli	0	0	1	0	2	0	9	3	2	1	0	0	0	0
Plt_disp	0	0	0	0	0	0	0	0	0	0	0	0	0	0

Plt_freq	0	0	0	0	0	0	0	0	0	0	0	0	0	0
Plt_hauc	0	0	0	0	3	0	23	14	11	8	0	0	1	0
Plt_lanc	0	0	0	0	0	0	0	0	0	0	0	0	0	0
Plt_lemm	0	0	0	0	0	0	0	0	0	0	0	0	0	0
Plt_link	0	0	0	0	0	0	0	0	0	0	0	0	0	0
Plt_peri	0	0	0	0	0	0	1	0	0	0	0	0	0	0
Plt_rost	0	0	0	0	0	0	0	0	0	0	0	0	0	0
Plt_sept	0	0	0	0	0	0	3	0	0	0	0	0	0	0
Psf_tene	0	0	0	0	0	0	0	0	0	0	0	0	0	0
Pss_brev	0	0	1	0	7	2	11	29	0	8	0	0	0	0
Pss_brin	0	0	0	0	3	1	2	0	0	41	0	0	0	0
Pss_elli	0	0	0	0	1	0	31	29	26	53	2	1	1	0
Pss_perm	0	0	1	0	0	0	4	3	1	1	0	0	0	0
Pss_robu	0	0	0	1	3	1	0	0	1	1	0	0	0	0
Pss_subs	0	0	0	0	0	0	0	0	0	0	0	0	0	0
Pss_zeil	0	0	0	0	0	8	10	69	16	43	0	0	1	0
Pst_bior	0	0	0	0	0	0	0	0	0	0	0	0	1	0
Pst_oblo	0	0	0	0	0	0	0	0	0	0	0	0	0	0
Pst_punc	0	0	0	0	0	0	0	0	0	0	0	0	0	0
Pst_ross	0	0	0	0	0	0	0	0	0	0	0	0	0	0
Pst_sacc	0	0	0	0	0	0	0	0	0	0	0	0	0	0
Pte_inan	0	0	0	0	0	0	0	0	0	0	0	0	0	0
Rei_sinu	0	0	0	0	0	0	0	0	0	0	0	0	0	0
Rho_abbr	74	42	19	43	22	66	32	5	14	43	6	112	25	7
Rhp_acum	0	0	0	0	0	0	0	0	0	0	0	0	0	0
Rhp_breb	0	0	0	0	0	0	0	0	0	0	0	0	0	0
Rhp_gibb	0	0	0	0	0	0	0	0	0	0	0	0	0	0
Sel_pupu	0	0	0	0	0	0	0	0	0	0	0	0	0	0
Sel_stro	0	0	0	0	0	0	0	0	0	0	0	0	0	0
Ske_cost	0	0	0	0	0	0	0	0	0	0	0	0	0	0
Ske_subs	0	0	0	0	0	0	0	0	0	0	0	0	0	0
Sla_lapp	0	0	0	0	0	0	0	0	0	0	0	0	0	0
Std_hant	0	0	0	0	0	0	5	1	1	0	0	0	0	0
Std_parv	0	0	0	0	0	0	0	0	0	0	0	0	0	0
Stn_legu	0	0	0	0	0	0	0	0	0	0	0	0	0	0
Stn_phoe	0	0	0	0	0	0	0	0	0	0	0	0	0	0
Stn_simu	0	0	0	0	0	0	0	0	0	0	0	0	0	0
Stn_smka	0	0	0	0	0	0	0	0	0	0	0	0	0	0
Stp_anus	0	0	0	0	0	0	0	0	0	0	0	0	0	0
Stp_wisl	0	0	0	0	0	0	0	0	0	0	0	0	0	0
Sts_punc	0	0	0	0	0	0	0	0	0	0	0	0	0	0

Sts_vent	0	0	1	0	0	0	30	42	18	14	0	0	0	0
Sur_angu	0	0	0	0	0	0	0	0	0	0	0	0	0	0
Sur_brba	0	0	0	0	0	0	0	0	0	0	0	0	0	0
Sur_breb	0	3	2	4	8	3	0	1	3	1	0	0	3	1
Sur_minu	0	0	0	0	0	0	0	0	0	0	0	0	0	0
Sur_oval	0	0	0	0	0	0	0	0	0	0	0	0	0	0
Syn_acus	0	0	0	0	0	0	0	0	0	0	0	0	0	0
Syn_ulna	0	0	0	0	0	0	0	0	0	0	0	0	0	0
Tab_fasc	0	5	6	0	1	1	4	5	4	1	1	0	1	0
Tab_kten	0	0	0	0	0	0	0	0	0	0	0	0	0	0
Tab_laev	0	0	0	0	0	0	0	0	0	0	0	0	0	0
Tab_tabu	1	0	0	0	0	0	0	0	6	5	0	0	0	0
Tab_waer	0	0	0	0	0	0	0	0	1	0	0	0	0	0
Tal_fene	0	0	0	0	0	0	0	0	0	0	0	0	0	0
Tal_floc	0	0	0	0	0	0	0	1	0	0	0	0	0	0
Tet_glan	0	0	0	0	0	0	0	0	0	0	0	0	0	0
Tha_balt	0	0	0	1	1	1	2	1	0	1	2	3	2	3
Tha_hyla	0	0	0	0	0	0	0	0	0	0	0	0	0	0
Tha_leva	0	0	0	0	0	0	0	0	0	0	2	0	0	0
Tha_pros	0	0	0	0	0	0	0	0	0	0	0	0	0	0
Tha_pseu	0	0	0	0	0	0	0	0	0	0	0	0	0	0
Tha_punc	0	0	0	0	0	0	0	0	0	0	0	0	0	0
Try_angu	0	2	0	0	0	0	0	0	0	0	0	0	0	0
Try_apic	0	0	0	0	0	0	0	0	0	0	0	0	0	0
Try_ardu	0	0	0	0	0	0	0	0	0	0	0	0	0	0
Try_hung	0	0	0	0	0	0	0	0	0	0	0	0	0	0
Try_levi	0	0	0	0	0	0	0	0	0	0	0	0	0	0
Try_lite	0	0	0	0	0	0	0	0	0	0	0	0	0	0
Try_sali	0	0	0	0	0	0	0	0	0	0	0	0	0	0
Try_scal	0	0	0	0	0	0	0	0	0	0	0	0	0	0

AREA	Ske	Ske	Ske	Ske	Ske	Ske	Ske	Ske	Ske	Ske	Ske	Ske	Ske	Ske
SITE	162	163	164	166	167	168	169	171	172	173	174	175	176	177
Acd_exil	0	0	0	1	0	0	0	0	0	0	0	0	0	2
Acd_minu	403	314	162	99	310	206	73	330	81	98	80	92	161	476
Acd_pyre	0	0	0	0	0	0	0	0	0	0	0	0	0	0
Ach_brev	0	0	0	0	0	0	0	0	0	0	0	0	0	0
Ach_brin	0	0	0	0	0	0	0	0	0	0	0	0	0	0
Ach_foge	0	0	0	0	0	0	0	0	0	0	0	0	0	0
Ach_leon	0	0	0	0	0	0	0	0	0	0	0	0	0	0
Ach_long	0	0	0	0	0	0	0	0	0	0	0	0	0	0
Ach_luth	0	0	0	0	0	0	0	0	0	0	0	0	0	0
Ach_misc	0	0	0	0	0	0	0	0	0	0	0	0	0	0
Ach_vist	0	0	0	0	0	0	0	0	0	0	0	0	0	0
Act_nosu	0	0	0	0	0	0	0	0	0	0	0	0	0	0
Act_occr	0	0	0	0	0	0	0	0	0	0	0	0	0	0
Ami_spec	0	0	0	0	0	0	0	0	0	0	0	0	0	0
Amp_copu	0	1	0	0	1	2	1	4	0	0	0	0	0	1
Amp_fleb	0	0	0	0	0	0	0	0	0	0	0	0	0	0
Amp_hele	0	0	0	0	0	0	0	0	0	0	0	0	0	0
Amp_inar	0	0	0	0	0	0	0	0	0	0	0	0	0	0
Amp_line	0	0	0	0	0	0	0	0	0	0	0	0	0	0
Amp_micr	0	0	0	0	0	0	0	0	0	0	0	0	0	0
Amp_oval	0	0	0	0	0	0	0	0	0	0	0	0	0	0
Amp_pedi	7	3	2	3	11	17	13	2	2	2	0	0	0	1
Amp_prof	0	0	0	0	0	0	0	0	0	0	0	0	0	0
Ane_mino	0	0	0	0	0	0	0	0	0	0	0	0	0	0
Ane_tusc	0	0	0	0	1	0	0	0	0	0	0	0	0	0
Ano_spha	0	0	0	0	0	0	2	0	0	0	0	0	0	0
Asa_bahu	0	0	0	0	0	0	0	0	0	0	0	0	0	0
Ast_form	0	0	0	0	0	0	0	1	0	0	0	0	0	0
Aul_spp	1	0	0	0	0	0	0	0	0	0	0	0	0	1
Bac_paxi	0	0	0	0	0	0	0	0	0	0	0	0	0	0
Ber_fenn	7	2	4	1	2	3	4	127	1	1	3	0	0	7
Ber_ruti	0	0	0	0	0	0	0	0	2	0	0	0	0	0
Ber_scop	0	0	0	0	0	0	0	0	0	0	0	0	0	0
Bir_luce	0	0	0	0	0	0	0	0	0	0	0	0	0	0
Bra_apon	0	0	1	0	1	2	3	16	0	0	0	0	0	0
Bra_vitr	35	11	11	2	6	9	0	22	1	1	0	5	2	17
Bre_lanc	0	0	0	0	0	0	0	0	0	0	0	0	0	0
Cal_aemu	0	0	0	0	0	0	0	0	0	0	0	0	0	0

Cal_amph	0	0	0	0	0	0	0	1	0	0	0	0	0	0
Cal_baci	0	0	1	0	0	0	2	0	0	0	0	0	0	0
Cal_cras	0	0	0	0	0	0	0	0	0	0	0	0	0	0
Cal_sili	0	0	0	0	0	0	1	0	0	0	0	0	0	0
Cam_clyp	0	0	0	0	0	0	0	0	0	0	0	0	0	0
Cat_adha	0	0	0	0	0	0	0	0	0	0	0	0	0	0
Cav_lapi	0	0	0	0	0	0	0	0	0	0	0	0	0	0
Cav_pseu	0	0	0	0	0	0	0	0	0	0	0	0	0	0
Cer_clos	0	0	0	0	0	0	0	0	0	0	0	0	0	0
Chp_clam	0	0	0	0	0	0	0	0	0	0	0	0	0	0
Chp_marg	0	0	0	0	0	1	0	0	0	0	0	0	0	1
Coc_cost	0	0	0	0	0	0	0	0	0	0	0	0	0	0
Coc_neot	0	0	0	3	0	0	2	0	0	0	0	0	0	1
Coc_pedi	1	0	1	4	1	0	0	0	3	0	0	0	0	0
Coc_pelt	0	0	0	0	0	0	0	0	0	0	0	0	0	0
Coc_plac	2	3	5	0	1	3	9	7	2	0	0	1	1	4
Coc_pleu	0	0	0	0	0	0	0	0	0	0	0	0	0	0
Coc_plli	0	0	0	0	0	0	0	0	0	0	0	0	0	0
Coc_psli	0	0	0	0	0	0	0	0	0	0	0	0	0	0
Coc_psth	0	0	0	0	0	0	0	0	0	0	0	0	0	0
Coc_scut	0	0	0	0	0	0	0	0	0	0	0	0	0	0
Coc_stau	0	0	0	0	0	0	0	0	0	0	0	0	0	0
Com_pusi	0	0	0	0	0	0	0	0	0	0	0	0	0	0
Cos_aste	0	0	0	0	0	0	0	0	0	0	0	0	0	0
Cte_pulc	1	11	13	181	17	5	0	0	6	2	0	9	0	4
Cyc_atom	0	0	0	0	0	0	0	0	0	0	0	0	0	0
Cyc_choc	0	0	0	0	0	0	0	0	0	0	0	0	0	0
Cyc_mene	0	0	0	0	0	0	2	0	0	0	0	0	0	0
Cyc_radi	0	0	0	0	0	0	0	0	0	0	0	0	0	0
Cyc_ross	0	0	1	0	0	0	0	2	0	0	0	0	0	0
Cyc_stel	0	0	0	0	0	0	0	1	0	0	0	0	0	0
Cyc_stri	0	0	0	0	0	0	0	0	0	0	0	0	0	0
Cyc_trip	0	0	0	0	0	0	0	0	0	0	0	0	0	0
Cym_affi	38	47	29	6	24	53	4	10	2	10	7	12	32	42
Cym_cist	0	0	0	0	0	0	0	0	0	0	0	0	0	0
Cym_helv	7	11	11	4	6	8	3	9	2	0	1	2	7	11
Cym_lanc	0	0	2	0	0	0	0	0	1	0	0	0	0	1
Cym_prox	0	0	0	0	0	0	0	0	0	0	0	0	0	0
Cyp_sole	0	0	0	0	0	0	0	0	0	0	0	0	0	0
Cys_dubi	0	0	0	0	0	0	0	0	0	0	0	0	0	0
Den_tecr	114	90	103	68	115	91	6	75	41	3	2	3	11	39

Dia_bott	3	7	0	4	1	17	0	0	0	3	1	2	2	1
Dia_moni	72	238	310	401	211	365	81	65	409	825	879	796	659	204
Dia_prob	0	0	0	0	0	0	0	0	0	0	0	0	0	0
Dia_tenu	0	0	0	0	0	5	0	0	0	6	1	1	0	0
Dia_vulg	0	0	0	0	0	0	0	0	0	0	0	0	0	0
Dic_subi	0	0	0	0	0	0	0	0	0	0	0	0	0	0
Dip_depa	0	0	0	0	0	0	0	0	0	0	0	0	0	0
Dip_dydi	0	0	0	0	0	0	0	0	0	0	0	0	0	0
Dip_elli	0	0	0	0	0	0	0	0	0	0	0	0	0	0
Dip_inte	0	0	0	0	0	0	0	0	0	0	0	0	0	0
Dip_ocul	0	0	0	0	0	0	0	0	0	0	0	0	0	0
Dip_smdi	0	0	0	0	0	0	0	0	0	0	0	0	0	0
Dip_smpu	0	0	1	0	0	1	0	0	0	0	0	0	0	0
Dip_smrh	0	0	0	0	0	0	0	0	0	0	0	0	0	0
Dip_subo	0	0	0	0	0	0	0	0	0	0	0	0	0	0
Enc_caes	0	1	0	0	0	0	0	1	0	0	0	0	0	0
Enc_lacu	0	1	0	0	0	0	0	0	0	1	0	0	0	1
Enc_minu	0	0	0	0	0	0	0	0	0	0	0	0	1	0
Enc_obsc	0	0	0	0	0	0	0	0	0	0	0	0	0	0
Enc_pros	0	0	0	0	0	0	0	0	0	0	0	0	0	0
Enc_sile	8	9	0	0	8	0	0	0	2	0	0	0	2	0
Enc_tenu	0	0	0	0	0	0	0	0	0	0	0	0	0	0
Enp_behr	0	0	0	0	0	0	0	0	0	0	0	0	0	0
Enp_kram	62	13	21	5	6	14	7	6	4	3	0	2	0	11
Ent_palu	0	0	2	0	2	1	3	5	1	0	1	2	1	1
Ent_pseu	0	0	0	0	0	0	0	0	0	0	0	0	0	0
Ent_punc	0	0	0	0	0	0	0	0	0	0	0	0	0	0
Epi_adna	0	0	0	0	0	0	0	0	0	0	0	0	0	0
Epi_goep	0	0	0	0	0	0	1	0	1	0	0	0	0	0
Epi_sore	0	1	1	0	1	1	1	0	0	0	0	1	0	0
Epi_turg	0	0	0	0	0	0	0	0	0	0	0	0	0	0
Epi_tuwe	0	0	0	0	0	0	0	0	0	0	0	0	0	0
Euc_laev	3	0	1	0	0	0	0	0	0	0	0	2	0	0
Eun_arcu	0	0	0	0	0	0	0	0	0	0	0	0	0	0
Eun_peun	0	0	0	0	0	0	0	0	0	0	0	0	1	0
Fal_clep	0	0	0	0	0	0	0	0	0	0	0	0	0	0
Fal_cryp	0	0	0	0	0	0	0	0	0	0	0	0	0	0
Fal_flor	0	0	0	0	0	0	0	0	0	0	0	0	0	0
Fal_psny	0	0	0	0	0	0	0	0	0	0	0	0	0	0
Fal_pygm	0	0	0	0	0	0	0	0	0	0	0	0	0	0
Fis_sapr	0	0	0	0	0	0	0	0	0	0	0	0	0	0

Fra_amic	0	0	0	0	0	0	9	0	0	0	0	0	0	0
Fra_capu	0	2	3	1	1	1	8	0	1	0	0	0	0	0
Fra_caru	15	56	115	39	75	47	29	26	22	4	4	15	39	47
Fra_cava	7	18	62	58	39	23	65	29	64	3	4	9	12	10
Fra_cons	0	0	0	0	0	0	0	0	0	0	0	0	0	0
Fra_geda	0	0	0	0	0	0	0	0	0	0	0	0	0	0
Fra_grac	3	5	0	1	0	1	14	3	0	1	4	12	21	14
Fra_hydu	0	0	0	0	0	0	0	0	0	0	0	0	0	0
Fra_lept	0	0	0	0	0	0	0	0	0	0	0	0	0	0
Fra_nitz	0	0	0	0	0	0	1	0	0	0	0	0	0	0
Frp_cyli	0	3	0	2	1	1	0	0	0	0	0	0	0	0
Fru_vulg	0	0	0	0	0	0	0	0	0	0	0	0	0	0
Gom_clav	0	0	0	0	0	0	0	0	0	0	0	0	0	0
Gom_intr	15	17	5	2	14	12	8	0	5	2	0	3	4	5
Gom_oliv	7	2	14	3	2	10	1	1	7	2	1	0	6	17
Gom_parv	0	0	0	0	0	0	0	0	0	0	0	0	0	0
Gon_exig	0	0	0	0	0	0	0	0	0	0	0	0	0	0
Gos_expl	0	0	0	0	0	0	0	0	0	0	0	0	0	0
Gos_obsc	0	0	0	0	0	0	0	0	0	0	0	0	0	0
Gra_mari	0	0	0	0	0	0	0	0	0	0	0	0	0	0
Gra_ocea	0	0	0	0	0	0	0	0	0	0	0	0	0	0
Gyr_acum	0	0	0	0	0	0	0	0	0	0	0	0	0	0
Gyr_atte	0	0	0	0	0	0	0	0	0	0	0	0	0	0
Gyr_stri	0	0	0	0	0	0	0	1	0	0	0	0	0	0
Gyr_tenu	0	0	0	0	0	0	0	0	1	0	0	0	0	0
Hal_coff	1	1	1	1	0	1	10	56	2	1	0	0	0	0
Hal_exig	0	0	1	0	0	0	0	0	0	0	0	0	0	0
Hal_hols	0	0	0	0	0	0	0	0	0	0	0	0	0	0
Hal_hybr	0	0	0	0	0	0	0	0	0	0	0	0	0	0
Hal_luci	0	0	0	0	0	0	0	0	0	0	0	0	0	0
Hal_norm	0	0	0	0	0	0	0	0	0	0	0	0	0	0
Hal_subh	0	0	0	0	0	0	0	0	0	0	0	0	0	0
Hal_tene	0	0	0	0	0	0	0	2	0	0	0	0	0	0
Hal_vene	8	3	7	0	1	3	6	18	0	0	2	0	1	2
Han_balt	0	0	0	0	0	0	0	0	0	0	0	0	0	0
Has_spic	0	0	0	0	0	0	1	0	0	0	0	0	0	0
Hip_capi	0	0	0	0	0	0	0	0	0	0	0	0	0	0
Hip_hung	0	0	0	0	0	0	2	1	2	0	0	0	0	0
Hip_lesm	0	0	0	0	0	0	0	0	0	0	0	0	0	0
Kar_amoe	0	0	0	0	0	0	0	0	0	0	0	0	0	0
Kar_clev	0	0	0	0	0	0	0	0	0	0	0	0	0	0

Kar_niti	0	0	0	0	0	0	0	0	0	0	0	0	0	0
Kar_subm	0	0	0	0	0	0	0	0	0	0	0	0	0	0
Lemn_hung	0	0	0	0	0	0	0	0	0	0	0	0	0	0
Lic_debi	0	0	0	0	0	0	0	0	0	0	0	0	0	0
Lic_gran	0	0	0	0	0	0	0	0	0	0	0	0	0	0
Lic_oedi	0	0	0	0	0	0	0	0	0	0	0	0	0	0
Lic_rhom	0	0	0	0	0	0	0	0	0	0	0	0	0	0
Lun_bise	0	0	1	0	2	2	1	7	4	2	0	0	0	0
Lut_muti	0	0	0	0	0	0	0	0	0	0	0	0	0	0
Mar_atom	0	0	2	4	0	0	57	1	3	0	0	0	0	1
Mar_mart	0	0	1	0	0	0	2	9	21	1	0	0	0	0
Mar_schu	1	0	6	4	3	1	108	3	0	1	0	0	0	1
Mas_balt	0	0	0	0	0	0	0	0	0	0	0	0	0	0
Mas_brau	0	0	0	0	0	0	0	0	0	0	0	0	0	0
Mas_elli	0	0	0	0	0	0	0	0	0	0	0	0	0	0
Mas_exig	0	0	0	0	0	0	0	0	0	0	0	0	0	0
Mas_pumi	0	0	0	0	0	0	0	0	0	0	0	0	0	0
Mas_pusi	0	0	0	0	0	0	0	0	0	0	0	0	0	0
Mas_smam	0	0	0	0	0	0	2	0	0	0	0	0	0	0
Mas_smit	0	0	0	0	0	0	2	0	0	0	0	0	0	0
Mel_arct	0	0	0	0	0	0	0	0	0	0	0	0	0	0
Mel_line	0	0	0	0	0	0	0	0	0	0	0	0	0	0
Mel_moni	0	0	0	0	0	0	0	0	0	0	0	0	0	0
Mel_numm	0	0	0	0	0	0	0	0	0	0	0	0	0	0
Mel_vari	0	0	0	0	0	0	0	0	0	0	0	0	0	0
Nav_aren	0	0	0	0	0	0	0	0	0	0	0	0	0	0
Nav_bipu	0	0	0	0	0	0	0	0	0	0	0	0	0	0
Nav_boss	0	0	0	0	0	0	0	0	0	0	0	0	0	0
Nav_bott	3	0	0	0	0	0	0	0	0	0	0	0	0	0
Nav_brem	0	0	0	0	0	0	0	0	0	0	0	0	0	0
Nav_capi	0	0	0	0	0	0	0	0	0	0	0	0	0	0
Nav_cinc	0	0	0	1	0	0	0	0	0	0	0	0	0	0
Nav_clem	0	0	0	0	0	0	0	1	0	0	0	0	0	0
Nav_cryp	9	4	8	3	2	3	2	2	4	1	1	0	2	0
Nav_digi	0	0	0	0	0	0	0	0	0	0	0	0	0	0
Nav_duer	0	0	0	0	0	0	0	0	0	0	0	0	0	0
Nav_eidr	0	1	0	0	0	0	0	0	0	0	0	0	0	0
Nav_flan	0	0	0	0	0	0	0	0	0	0	0	0	0	0
Nav_geii	0	0	0	0	0	0	0	0	0	0	0	0	0	0
Nav_gepo	0	0	0	0	0	0	0	0	0	0	0	0	0	0
Nav_greg	0	2	2	1	7	0	11	9	10	3	0	0	0	1

Nav_haca	0	0	0	0	0	0	0	0	0	0	0	0	0	0
Nav_hani	0	0	0	0	0	0	0	0	0	0	0	0	0	0
Nav_hust	0	0	0	0	0	0	0	0	0	0	0	0	0	0
Nav_lanc	0	0	0	0	0	0	0	0	0	0	0	0	0	0
Nav_late	0	0	0	0	0	0	0	0	0	0	0	0	0	0
Nav_marg	0	0	0	0	0	0	0	0	0	0	0	0	0	0
Nav_meni	0	1	0	0	0	0	0	0	0	0	0	0	0	0
Nav_midi	0	0	0	0	0	0	0	0	0	0	0	0	0	0
Nav_nole	0	0	0	0	0	0	0	0	0	0	0	0	0	0
Nav_oblo	0	0	0	0	0	0	0	1	0	0	0	0	0	0
Nav_oest	0	0	0	0	0	0	0	0	0	0	0	0	0	0
Nav_pere	0	0	0	0	0	0	0	0	0	0	0	0	0	0
Nav_perm	0	0	0	0	1	0	0	0	0	0	0	0	0	0
Nav_phyl	0	0	0	0	1	0	3	3	0	1	0	0	0	0
Nav_port	0	0	0	0	0	0	0	0	0	0	0	0	0	0
Nav_radi	0	0	0	0	0	0	1	0	0	0	0	0	0	0
Nav_ramo	0	0	0	0	0	0	0	0	0	0	0	0	0	0
Nav_rato	0	0	0	0	0	0	0	0	0	0	0	0	0	0
Nav_rece	1	0	0	0	0	0	0	0	0	0	0	0	0	0
Nav_rhyn	0	1	1	1	0	1	12	2	0	0	0	0	1	0
Nav_sana	0	0	0	0	0	0	0	0	0	0	0	0	0	0
Nav_sjoe	0	0	0	0	0	0	0	0	0	0	0	0	0	0
Nav_star	0	0	0	0	0	0	0	0	0	0	0	0	0	0
Nav_subr	0	0	0	0	0	0	0	0	0	0	0	0	0	0
Nav_supr	0	0	0	0	0	0	0	0	0	0	0	0	0	0
Nav_trip	0	0	0	0	0	0	0	0	0	0	0	0	0	0
Nav_vene	0	0	0	0	0	0	2	0	0	0	1	0	0	0
Ncy_pusi	0	0	0	0	0	0	2	0	1	0	0	0	0	0
Nit_acic	0	0	0	0	0	0	0	0	0	0	0	0	0	0
Nit_anfo	0	0	1	0	5	0	0	0	0	0	0	0	0	0
Nit_anla	0	0	0	0	0	0	0	0	0	0	0	0	0	0
Nit_arch	0	0	0	0	0	0	0	0	0	0	0	0	0	0
Nit_aura	17	7	0	0	16	10	1	0	0	0	0	0	0	0
Nit_baci	0	0	0	0	0	0	0	5	9	0	2	0	2	2
Nit_dime	1	0	0	0	0	0	0	0	0	0	0	0	0	0
Nit_dipp	0	1	0	0	0	0	0	0	0	0	0	0	0	0
Nit_diss	0	0	0	0	0	0	0	1	1	0	0	0	0	2
Nit_dist	0	0	0	0	0	0	0	0	0	0	0	0	0	0
Nit_dubi	0	0	0	0	0	0	0	0	0	0	0	0	0	0
Nit_eleg	0	0	0	0	0	0	0	0	0	0	0	0	0	0
Nit_frig	0	0	0	0	0	0	0	0	0	0	0	0	0	0

Nit_frus	15	11	12	4	34	16	1	4	9	3	0	0	1	1
Nit_gros	0	0	0	0	0	0	0	0	0	0	0	0	0	0
Nit_heuf	0	0	1	0	1	0	0	1	0	0	0	0	0	2
Nit_inco	4	0	1	1	0	2	1	1	24	1	0	0	0	1
Nit_inte	0	0	0	0	12	33	0	1	0	0	0	0	0	0
Nit_lieb	55	44	18	8	18	0	7	3	20	2	0	9	1	7
Nit_lisu	0	0	0	0	0	0	0	21	30	1	0	0	0	0
Nit_lore	0	0	0	0	0	0	0	0	0	0	0	0	0	0
Nit_micr	0	0	1	0	1	0	5	9	0	0	0	0	0	0
Nit_pale	0	0	0	0	0	0	0	0	0	0	0	0	0	0
Nit_pcea	0	0	0	0	0	3	2	6	1	0	0	0	0	0
Nit_pell	0	0	0	0	0	0	0	0	0	0	0	0	0	0
Nit_perm	0	0	0	0	0	0	0	0	0	0	0	0	0	0
Nit_pusi	0	1	0	0	2	0	2	7	0	0	0	0	0	0
Nit_rect	0	0	1	0	0	1	0	0	0	0	0	0	0	0
Nit_rose	70	42	22	4	16	9	6	16	22	1	0	15	12	42
Nit_saco	0	0	0	0	0	0	0	0	0	0	0	0	0	0
Nit_sdea	0	0	0	0	0	0	0	0	0	0	0	0	0	0
Nit_sigm	0	0	0	0	0	1	0	0	0	0	0	0	0	0
Nit_soci	0	0	0	0	0	0	4	0	2	0	0	0	0	0
Nit_soli	0	0	0	0	0	0	1	0	0	0	0	0	0	0
Nit_subl	0	0	0	0	0	0	0	0	0	0	0	0	0	0
Nit_ther	0	0	0	0	4	3	4	10	1	1	0	5	0	0
Nit_umbo	0	0	0	0	0	0	0	0	0	0	0	0	0	0
Nit_vald	0	0	0	2	0	0	5	0	0	0	0	0	0	0
Nit_verm	0	0	0	0	0	0	0	0	0	0	0	0	0	0
Nit_vitr	1	2	1	0	3	1	0	0	0	0	0	0	0	0
Nit_wuel	0	0	0	0	0	0	0	0	0	0	0	0	0	0
Ope_krum	0	0	0	0	0	0	0	0	0	0	0	0	0	0
Ope_muta	0	0	0	0	0	0	0	0	0	0	0	0	0	0
Par_prot	0	0	0	0	0	0	0	0	0	0	0	0	0	0
Pau_taen	0	0	0	0	0	1	0	0	0	0	0	0	0	0
Pet_hume	0	0	0	0	0	0	0	0	0	0	0	0	0	0
Pin_appe	0	0	0	0	0	0	3	0	0	0	0	0	0	0
Pin_dimi	0	0	0	0	0	0	0	0	0	0	0	0	0	1
Pin_kroc	0	0	0	0	0	0	0	0	0	0	0	0	0	0
Pin_micr	0	0	0	0	0	0	0	0	0	0	0	0	0	0
Plc_plac	0	0	0	0	0	0	0	0	0	0	0	0	0	0
Plt_cons	0	0	0	0	0	0	0	0	0	0	0	0	0	0
Plt_deli	0	0	0	1	0	0	0	0	2	1	0	0	0	1
Plt_disp	0	0	0	0	0	0	0	0	0	0	0	0	0	0

Plt_freq	0	0	0	0	0	0	0	0	1	0	0	0	0	0
Plt_hauc	0	1	3	3	1	2	2	5	24	3	0	0	2	1
Plt_lanc	0	0	0	0	0	0	0	0	0	0	0	0	0	0
Plt_lemm	0	0	0	0	0	0	0	0	0	0	0	0	0	0
Plt_link	0	0	0	0	0	0	0	0	0	0	0	0	0	0
Plt_peri	0	0	0	0	0	0	0	0	0	0	0	0	0	0
Plt_rost	0	0	0	0	0	0	0	0	0	0	0	0	0	0
Plt_sept	0	0	0	0	0	0	0	0	0	0	0	0	0	0
Psf_tene	0	0	0	0	0	0	0	0	0	0	0	0	0	0
Pss_brev	0	0	0	0	0	0	8	7	15	0	0	0	0	0
Pss_brin	0	1	11	0	0	1	42	16	28	0	0	0	0	0
Pss_elli	0	2	0	0	0	0	112	4	0	0	0	0	0	0
Pss_perm	0	0	1	0	0	0	6	2	0	0	0	0	0	0
Pss_robu	0	0	1	1	0	0	0	0	0	0	0	0	0	0
Pss_subs	0	0	0	0	0	0	0	0	0	0	0	0	0	0
Pss_zeil	0	0	1	1	1	0	167	4	14	0	0	0	0	0
Pst_bior	0	0	0	0	0	0	0	0	0	0	0	0	0	0
Pst_oblo	0	0	0	0	0	0	0	0	0	0	0	0	0	0
Pst_punc	0	0	0	0	0	0	0	0	0	0	0	0	0	0
Pst_ross	0	0	0	0	0	0	0	0	0	0	0	0	0	0
Pst_sacc	0	0	0	0	0	0	0	0	0	0	0	0	0	0
Pte_inan	0	0	0	0	0	0	0	0	0	0	0	0	0	0
Rei_sinu	0	0	0	0	1	0	0	0	0	0	0	0	0	0
Rho_abbr	3	6	9	68	9	3	3	3	73	10	0	2	13	13
Rhp_acum	0	0	0	0	0	0	0	0	0	0	0	0	0	0
Rhp_breb	0	0	0	0	0	0	0	0	0	0	0	0	0	0
Rhp_gibb	0	0	0	0	0	0	0	0	0	0	0	0	0	0
Sel_pupu	0	0	0	0	0	0	0	0	0	0	0	0	0	0
Sel_stro	0	0	0	0	0	0	0	0	0	0	0	0	0	0
Ske_cost	0	0	0	0	0	0	0	0	0	0	0	0	0	0
Ske_subs	0	0	0	0	0	0	0	0	0	0	0	0	0	0
Sla_lapp	0	0	0	0	0	0	0	1	0	0	0	0	0	0
Std_hant	0	0	0	0	0	0	0	0	0	0	0	0	0	0
Std_parv	0	0	0	0	0	0	0	0	0	0	0	0	0	0
Stn_legu	0	0	0	0	0	0	0	0	0	0	0	0	0	0
Stn_phoe	0	0	0	0	0	0	0	0	0	0	0	0	0	0
Stn_simu	0	0	0	0	0	0	0	0	0	0	0	0	0	0
Stn_smka	0	0	0	0	0	0	0	0	0	0	0	0	0	0
Stp_anus	0	0	0	0	0	0	0	0	0	0	0	0	0	0
Stp_wisl	0	0	0	0	0	0	0	0	0	0	0	0	0	0
Sts_punc	0	0	0	0	0	0	0	0	0	0	0	0	0	0

Sts_vent	0	0	0	0	0	0	36	0	0	0	0	0	0	0
Sur_angu	0	0	0	0	0	0	1	0	1	0	0	0	0	0
Sur_brba	0	0	0	0	0	0	0	1	2	0	0	0	0	0
Sur_breb	0	1	5	3	0	2	2	6	7	0	6	0	2	3
Sur_minu	0	0	0	0	1	0	0	0	0	0	0	0	0	0
Sur_oval	0	0	0	0	0	0	0	0	0	0	0	0	0	0
Syn_acus	0	0	0	0	0	0	1	3	0	0	0	0	0	0
Syn_ulna	0	0	0	0	0	1	0	0	0	0	0	0	0	0
Tab_fasc	0	1	0	0	1	1	0	0	5	0	0	0	0	0
Tab_kten	0	0	0	0	0	0	0	0	0	0	0	0	0	0
Tab_laev	0	0	0	0	0	0	0	0	0	0	0	0	0	0
Tab_tabu	0	0	0	0	0	0	0	0	0	0	0	0	0	0
Tab_waer	0	0	0	0	0	0	0	0	0	0	0	0	0	0
Tal_fene	0	0	0	0	0	0	0	0	0	0	0	0	0	0
Tal_floc	0	0	0	0	0	0	0	0	0	0	0	0	1	0
Tet_glan	0	0	0	0	0	0	0	0	0	0	0	0	0	0
Tha_balt	0	0	0	0	0	0	0	0	1	0	0	0	0	0
Tha_hyla	0	0	0	0	0	0	0	0	0	0	0	0	0	0
Tha_leva	0	0	0	0	0	0	0	0	0	0	0	0	0	0
Tha_pros	0	0	0	0	0	0	0	0	0	0	0	0	0	0
Tha_pseu	0	0	0	0	0	0	0	0	0	0	0	0	0	0
Tha_punc	0	0	0	0	0	0	0	0	0	0	0	0	0	0
Try_angu	0	1	0	0	0	0	0	2	0	0	0	0	0	0
Try_apic	0	0	0	0	0	0	0	0	0	0	0	0	0	0
Try_ardu	0	0	0	0	0	0	1	2	0	0	0	0	0	0
Try_hung	0	0	0	0	0	0	0	0	0	0	0	0	0	0
Try_levi	0	0	0	0	0	0	0	0	0	0	0	0	0	0
Try_lite	0	0	0	0	0	0	0	0	0	0	0	0	0	0
Try_sali	0	0	0	1	0	0	0	0	0	0	0	0	0	0
Try_scal	0	0	0	0	0	0	0	0	0	0	0	0	0	0

AREA	Rån	Rån	Rån	Rån	Rån	Rån	Rån	Rån	Rån	Rån	Rån	Rån
SITE	178	179	180	181	182	185	186	187	189	190	191	192
Acd_exil	0	0	0	0	0	0	0	0	0	0	0	0
Acd_minu	258	267	81	85	84	193	370	226	395	151	340	309
Acd_pyre	0	0	0	0	0	2	0	0	0	0	0	0
Ach_brev	0	0	0	0	0	0	0	0	0	0	0	0
Ach_brin	0	0	0	0	0	0	0	0	0	0	0	0
Ach_foge	0	0	0	1	0	0	0	0	0	0	0	2
Ach_leon	0	0	0	0	0	0	0	0	0	0	0	0
Ach_long	0	0	0	0	0	0	0	0	0	0	0	0
Ach_luth	0	0	0	0	0	0	0	0	0	0	0	0
Ach_misc	0	0	0	0	0	0	0	0	0	0	0	1
Ach_vist	0	0	0	0	0	0	2	0	0	1	0	4
Act_nosu	0	0	0	0	0	0	0	0	0	0	0	0
Act_occr	0	0	0	0	0	0	0	0	0	0	0	0
Ami_spec	0	0	0	0	0	0	0	0	0	0	0	0
Amp_copu	1	0	1	0	0	6	0	1	3	1	1	0
Amp_fleb	0	0	0	0	0	0	0	0	0	0	0	0
Amp_hele	0	0	0	0	0	0	0	0	0	0	0	0
Amp_inar	0	0	0	4	2	1	0	0	0	0	0	2
Amp_line	0	0	0	0	0	0	0	0	0	0	0	0
Amp_micr	0	0	0	0	0	0	0	0	0	0	0	0
Amp_oval	0	0	0	0	0	0	0	3	0	0	3	0
Amp_pedi	29	17	16	21	18	9	7	17	6	53	17	24
Amp_prof	0	0	0	0	0	0	0	0	0	0	0	0
Ane_mino	3	1	5	0	0	0	1	1	0	0	0	0
Ane_tusc	0	0	1	0	0	0	0	0	0	0	0	2
Ano_spha	0	0	0	0	0	0	0	0	0	0	0	0
Asa_bahu	0	0	0	0	0	0	0	0	0	0	0	0
Ast_form	0	0	0	0	0	0	0	0	0	1	0	0
Aul_spp	2	1	0	0	0	3	0	0	0	2	0	1
Bac_paxi	0	0	0	0	0	0	0	0	0	0	0	0
Ber_fenn	30	9	1	0	0	2	37	58	40	7	99	3
Ber_ruti	0	1	0	0	0	0	0	0	0	0	0	0
Ber_scop	0	0	0	0	0	0	0	0	0	0	0	0
Bir_luce	0	0	0	0	0	0	0	0	0	0	0	0
Bra_apon	0	0	0	0	0	1	1	2	2	1	3	0
Bra_vitr	29	25	0	0	0	14	71	43	17	16	27	1
Bre_lanc	0	0	0	0	0	0	0	0	0	0	0	0
Cal_aemu	0	0	0	1	0	0	0	0	0	0	0	0

Cal_amph	0	0	0	0	0	0	0	0	0	0	0	0
Cal_baci	1	2	0	1	0	1	0	0	0	1	0	0
Cal_cras	0	0	0	0	0	0	0	0	0	0	0	0
Cal_sili	0	0	0	0	0	1	0	0	0	1	0	0
Cam_clyp	0	0	0	0	0	0	0	0	0	0	0	0
Cat_adha	0	0	0	0	0	0	0	0	0	0	0	0
Cav_lapi	0	0	0	0	0	0	0	0	0	0	0	0
Cav_pseu	0	0	0	1	0	0	0	0	0	0	0	0
Cer_clos	0	0	0	0	0	0	0	0	0	0	0	0
Chp_clam	0	0	0	0	0	0	0	0	0	0	0	0
Chp_marg	0	2	0	0	0	2	0	0	0	0	0	0
Coc_cost	0	0	0	0	0	0	0	0	0	0	0	0
Coc_neot	1	4	5	15	8	10	4	3	0	33	3	0
Coc_pedi	0	0	0	1	0	0	0	0	0	2	0	1
Coc_pelt	0	0	0	0	0	0	0	0	0	0	0	0
Coc_plac	5	6	9	3	18	0	0	2	1	15	2	8
Coc_pleu	0	0	0	0	0	0	0	0	0	0	0	0
Coc_plli	0	0	0	0	0	0	0	0	0	0	0	0
Coc_psli	0	0	0	0	0	0	0	0	0	0	0	0
Coc_psth	0	0	0	0	0	0	0	0	0	0	0	0
Coc_scut	0	0	0	0	0	0	0	0	0	0	0	0
Coc_stau	0	0	0	0	0	0	0	0	0	0	0	0
Com_pusi	0	0	0	0	0	0	0	0	0	0	0	0
Cos_aste	0	0	0	0	0	0	0	0	0	0	0	0
Cte_pulc	3	6	3	9	8	31	3	5	3	0	2	2
Cyc_atom	0	0	0	0	0	0	0	0	0	0	0	0
Cyc_choc	0	0	0	0	0	0	0	0	0	0	0	0
Cyc_mene	0	0	3	0	0	7	0	2	0	0	0	0
Cyc_radi	0	0	0	0	0	0	0	0	0	0	0	0
Cyc_ross	0	0	0	0	0	0	0	0	0	0	0	0
Cyc_stel	0	0	1	0	0	0	0	0	0	0	0	0
Cyc_stri	0	0	0	0	0	0	0	0	0	0	0	0
Cyc_trip	0	0	0	0	0	0	0	0	0	0	0	0
Cym_affi	22	19	23	10	12	26	17	20	41	17	12	13
Cym_cist	0	1	2	1	0	0	0	0	0	0	1	0
Cym_helv	5	4	5	1	0	6	12	30	32	5	12	10
Cym_lanc	0	1	0	1	0	2	0	1	1	2	1	0
Cym_prox	0	0	0	1	0	0	0	0	0	0	0	0
Cyp_sole	0	0	0	1	0	0	0	0	0	0	0	0
Cys_dubi	0	0	0	0	0	0	0	0	0	0	0	0
Den_tecr	24	27	5	0	8	31	40	140	25	26	17	55

Dia_bott	0	0	0	0	0	8	0	0	10	0	0	0
Dia_moni	89	28	334	215	220	186	24	42	43	96	94	93
Dia_prob	0	0	0	0	0	0	0	0	0	0	0	0
Dia_tenu	0	0	0	0	0	0	2	0	0	0	0	0
Dia_vulg	0	0	0	0	0	0	0	0	0	0	0	2
Dic_subi	0	0	0	0	0	0	0	0	0	0	0	0
Dip_depa	0	0	0	0	0	0	0	0	0	0	0	0
Dip_dydi	0	0	0	0	0	0	0	0	0	0	0	0
Dip_elli	0	0	2	3	0	0	0	0	0	0	0	0
Dip_inte	0	0	0	0	0	0	0	0	0	0	0	1
Dip_ocul	0	0	0	1	2	0	0	0	0	0	0	0
Dip_smdi	0	0	0	0	0	0	0	0	0	1	0	0
Dip_smpu	1	1	0	2	0	0	0	0	1	0	0	0
Dip_smrh	0	0	0	0	0	0	0	0	0	0	0	0
Dip_subo	0	1	0	0	0	0	0	0	0	0	0	0
Enc_caes	0	1	2	5	0	2	1	2	0	1	1	10
Enc_lacu	3	27	2	1	2	0	3	2	0	3	1	2
Enc_minu	0	1	1	7	12	6	5	2	6	6	31	4
Enc_obsc	0	0	0	0	0	0	0	1	0	0	0	0
Enc_pros	2	0	0	1	2	0	0	0	0	0	1	1
Enc_sile	18	16	21	47	36	24	12	0	7	8	10	15
Enc_tenu	0	0	0	0	0	0	0	0	0	0	0	0
Enp_behr	0	0	0	0	0	0	0	0	0	0	0	0
Enp_kram	80	68	21	2	0	9	50	73	55	32	16	22
Ent_palu	0	1	2	1	0	0	0	0	0	3	3	0
Ent_pseu	0	0	0	0	0	0	0	0	0	0	0	1
Ent_punc	0	0	0	0	0	0	0	0	0	0	0	0
Epi_adna	0	0	2	0	6	0	0	0	0	0	0	0
Epi_goep	0	0	0	0	0	0	0	0	0	0	0	0
Epi_sore	4	14	7	13	2	2	2	2	10	6	2	1
Epi_turg	0	0	1	2	4	2	1	0	2	1	0	1
Epi_tuwe	0	0	0	0	0	0	0	0	0	0	0	0
Euc_laev	0	0	2	0	0	1	0	0	0	0	0	0
Eun_arcu	0	0	0	0	0	0	0	0	0	0	0	0
Eun_peun	0	0	0	0	0	0	0	0	0	0	0	0
Fal_clep	0	1	0	1	0	0	0	0	0	0	0	0
Fal_cryp	0	0	0	0	0	0	0	0	0	0	0	0
Fal_flor	0	0	0	1	0	0	0	0	0	0	0	0
Fal_psny	0	0	0	0	0	0	0	0	0	0	0	0
Fal_pygm	0	0	2	0	2	0	0	0	0	0	0	0
Fis_sapr	0	0	0	1	0	1	0	0	0	0	0	0

Fra_amic	0	0	0	0	0	0	0	0	0	0	0	0
Fra_capu	0	0	0	0	0	0	21	0	0	0	0	0
Fra_caru	46	85	42	68	34	128	42	120	127	78	38	39
Fra_cava	118	162	175	117	170	53	76	15	37	29	12	37
Fra_cons	0	0	0	0	0	0	0	0	0	0	0	0
Fra_geda	0	0	0	0	0	0	0	0	0	0	0	0
Fra_grac	19	6	11	14	26	10	38	42	39	39	26	9
Fra_hydu	0	0	0	0	0	0	0	0	0	0	0	0
Fra_lept	0	0	0	0	0	0	0	0	0	0	1	0
Fra_nitz	0	0	0	0	0	0	0	0	0	0	0	0
Frp_cyli	0	0	0	0	0	0	0	0	0	0	0	1
Fru_vulg	0	0	0	0	0	0	0	0	0	0	0	0
Gom_clav	0	1	0	0	0	1	0	3	2	2	0	6
Gom_intr	6	2	0	1	8	9	29	5	3	0	1	0
Gom_oliv	3	12	27	1	0	4	7	0	8	2	12	3
Gom_parv	0	0	1	1	4	0	0	0	0	0	0	8
Gon_exig	0	0	0	0	0	0	0	0	0	0	0	0
Gos_expl	0	0	0	0	0	0	0	0	0	0	0	0
Gos_obsc	0	0	0	0	0	0	0	0	0	1	0	0
Gra_mari	0	0	0	0	0	0	0	0	0	0	0	0
Gra_ocea	0	0	0	0	0	0	0	0	0	0	0	0
Gyr_acum	0	0	0	0	0	0	0	0	0	0	0	0
Gyr_atte	0	0	0	2	0	0	0	0	0	0	0	0
Gyr_stri	0	0	0	0	0	0	0	0	0	0	0	0
Gyr_tenu	0	0	0	0	0	0	0	0	0	0	0	0
Hal_coff	5	1	1	5	6	1	9	10	5	10	48	5
Hal_exig	0	0	0	0	0	0	0	0	0	0	0	0
Hal_hols	0	0	0	0	0	0	0	0	0	0	0	0
Hal_hybr	0	0	0	1	0	0	0	0	0	0	0	0
Hal_luci	0	0	0	0	0	0	0	2	3	0	0	0
Hal_norm	0	0	0	0	4	0	0	0	0	0	0	0
Hal_subh	0	0	0	0	0	0	0	0	0	0	0	0
Hal_tene	0	0	3	0	0	0	0	0	0	0	0	0
Hal_vene	13	31	3	8	0	0	5	2	3	10	3	1
Han_balt	0	0	0	0	2	0	0	0	0	0	0	0
Has_spic	0	1	0	0	2	0	0	0	0	2	0	0
Hip_capi	0	0	0	15	14	0	0	0	0	0	0	0
Hip_hung	1	0	0	1	4	1	0	0	0	0	1	0
Hip_lesm	0	0	0	1	0	0	0	0	0	0	0	2
Kar_amoe	0	0	0	0	0	0	0	0	0	0	0	0
Kar_clev	1	2	1	2	0	6	4	0	0	7	1	0

Kar_niti	0	0	0	0	0	1	0	0	0	0	0	0
Kar_subm	0	0	0	0	0	0	0	0	0	0	0	0
Lemn_hung	0	0	1	0	0	0	0	0	0	0	0	0
Lic_debi	0	0	0	0	0	0	0	0	0	0	0	0
Lic_gran	0	0	0	0	0	0	0	0	0	0	0	0
Lic_oedi	0	0	0	0	0	0	0	0	0	0	0	0
Lic_rhom	0	0	0	0	0	0	0	0	0	0	0	0
Lun_bise	3	0	1	0	0	2	2	2	0	5	0	1
Lut_muti	0	0	0	0	0	0	0	0	0	0	0	0
Mar_atom	12	6	12	18	18	19	0	0	1	13	1	15
Mar_mart	3	1	2	3	6	3	0	0	0	3	0	1
Mar_schu	5	5	6	22	20	9	0	1	3	26	7	25
Mas_balt	0	0	0	0	0	0	0	0	0	0	0	0
Mas_brau	0	0	0	0	0	0	0	0	0	0	0	0
Mas_elli	0	0	0	2	0	0	0	0	0	0	0	0
Mas_exig	0	0	0	0	0	0	0	0	0	0	0	0
Mas_pumi	0	0	0	0	0	0	0	0	0	0	0	0
Mas_pusi	0	0	0	0	0	0	0	0	0	0	0	0
Mas_smam	8	1	2	6	6	3	1	0	2	3	6	2
Mas_smit	10	1	5	13	24	2	5	2	2	2	2	0
Mel_arct	0	0	0	0	0	0	0	0	0	0	0	0
Mel_line	0	1	3	1	2	1	1	0	0	0	0	2
Mel_moni	0	0	0	0	0	0	0	0	0	0	0	0
Mel_numm	0	0	0	0	0	0	0	0	0	0	0	0
Mel_vari	0	0	0	1	0	0	0	0	0	0	0	0
Nav_aren	0	0	0	0	0	0	0	0	0	0	0	0
Nav_bipu	0	0	0	0	0	0	0	0	0	0	0	0
Nav_boss	0	0	0	0	0	0	0	0	0	0	0	0
Nav_bott	0	0	0	0	0	1	0	0	0	0	1	0
Nav_brem	0	0	1	0	0	0	0	0	0	0	0	0
Nav_capi	0	0	0	9	4	0	0	0	0	0	1	0
Nav_cinc	0	0	0	0	0	0	0	0	0	0	0	0
Nav_clem	0	1	0	0	0	0	0	0	0	0	0	0
Nav_cryp	13	9	3	14	12	13	32	34	26	31	19	3
Nav_digi	0	0	0	0	0	0	0	0	0	1	0	0
Nav_duer	0	0	0	0	0	0	0	0	0	1	0	0
Nav_eidr	0	0	0	0	0	0	0	0	0	0	0	0
Nav_flan	0	0	0	0	0	0	0	0	0	0	0	0
Nav_geii	0	0	0	0	0	0	0	0	0	0	0	0
Nav_gepo	1	0	0	0	0	0	0	0	0	0	0	0
Nav_greg	12	8	10	5	2	9	0	0	0	0	1	2

Nav_haca	0	0	0	0	0	0	0	0	0	0	0	0
Nav_hani	0	0	0	0	0	0	0	0	0	0	0	0
Nav_hust	0	0	0	0	0	0	0	0	0	0	0	0
Nav_lanc	0	0	0	0	0	0	0	0	0	1	0	0
Nav_late	0	0	0	0	0	0	0	0	0	0	0	0
Nav_marg	0	0	0	0	0	0	0	0	0	0	0	0
Nav_meni	0	0	0	0	2	2	0	0	0	0	0	0
Nav_midi	0	0	0	0	0	0	0	0	0	0	0	0
Nav_nole	0	0	1	0	2	0	0	0	0	0	0	0
Nav_oblo	0	0	0	0	0	0	0	0	0	0	0	0
Nav_oest	0	0	0	0	0	0	0	0	0	0	0	0
Nav_pere	0	0	0	0	0	0	0	0	0	0	0	0
Nav_perm	0	2	4	3	2	2	0	0	0	1	1	0
Nav_phyl	1	1	3	1	2	3	1	1	0	5	0	26
Nav_port	0	0	0	0	0	0	0	0	0	0	0	0
Nav_radi	0	0	0	0	0	0	0	0	0	0	0	0
Nav_ramo	0	0	0	0	0	0	0	0	0	0	0	0
Nav_rato	0	0	0	0	0	0	0	0	0	0	0	0
Nav_rece	0	0	0	0	0	0	0	0	0	0	0	0
Nav_rhyn	0	4	7	7	2	2	2	0	1	1	0	6
Nav_sana	1	0	0	1	0	1	0	0	0	1	0	0
Nav_sjoe	0	0	0	0	0	0	0	0	0	0	0	0
Nav_star	0	0	0	0	0	0	0	0	0	0	0	0
Nav_subr	1	0	0	0	0	0	0	0	0	0	0	0
Nav_supr	0	0	0	0	0	0	0	0	0	0	0	0
Nav_trip	0	0	0	0	0	0	0	0	0	0	0	0
Nav_vene	0	1	1	3	12	7	0	0	2	4	0	0
Ncy_pusi	0	0	2	0	0	0	0	1	0	7	1	0
Nit_acic	0	0	1	1	0	1	0	0	0	0	1	0
Nit_anfo	0	0	0	0	0	0	0	0	0	0	0	0
Nit_anla	0	1	0	0	0	0	0	0	8	4	0	0
Nit_arch	0	0	0	0	0	0	0	0	0	0	0	0
Nit_aura	0	0	0	0	0	0	0	0	0	0	0	0
Nit_baci	5	2	1	0	0	3	0	3	1	14	3	2
Nit_dime	0	0	0	0	0	1	0	0	0	0	0	1
Nit_dipp	0	0	0	0	2	0	0	0	0	0	0	2
Nit_diss	0	2	3	8	14	8	0	7	2	1	1	13
Nit_dist	0	0	0	0	0	0	0	0	0	0	0	0
Nit_dubi	0	0	0	0	2	0	0	0	0	0	0	0
Nit_eleg	0	0	0	0	0	0	0	0	0	0	0	0
Nit_frig	0	0	0	0	0	0	0	0	0	0	0	0

Nit_frus	0	1	0	0	0	2	4	0	0	0	1	0
Nit_gros	0	0	0	0	0	0	0	0	0	0	0	0
Nit_heuf	0	1	3	3	0	0	0	0	1	2	0	0
Nit_inco	0	0	0	1	0	5	1	0	1	0	2	1
Nit_inte	0	0	0	0	0	0	0	0	0	0	0	0
Nit_lieb	3	2	4	1	0	13	2	7	3	1	1	14
Nit_lisu	0	0	1	1	2	0	14	0	0	0	8	0
Nit_lore	0	0	0	0	0	0	0	0	0	0	0	0
Nit_micr	4	1	2	0	0	0	0	3	0	3	11	19
Nit_pale	0	0	0	0	0	0	0	1	0	5	5	5
Nit_pcea	0	0	0	0	0	0	0	0	0	0	1	1
Nit_pell	0	0	5	6	8	1	0	2	0	0	0	1
Nit_perm	1	0	0	0	0	0	0	0	0	0	0	8
Nit_pusi	2	6	0	1	0	1	0	3	0	0	14	0
Nit_rect	0	0	0	0	0	0	0	0	0	0	0	1
Nit_rose	13	17	4	11	10	3	12	31	7	16	0	0
Nit_saco	0	0	0	0	0	0	0	0	0	0	0	0
Nit_sdea	0	0	2	0	0	0	0	0	0	0	0	0
Nit_sigm	0	1	1	1	0	0	0	0	0	0	0	0
Nit_soci	0	0	0	0	0	0	0	0	0	0	0	0
Nit_soli	0	0	0	0	0	0	0	0	1	0	0	0
Nit_subl	0	0	0	0	0	0	0	0	0	0	0	2
Nit_ther	4	3	7	4	2	1	3	4	2	1	1	0
Nit_umbo	0	0	0	0	0	0	0	0	0	1	0	0
Nit_vald	2	1	3	1	0	1	0	1	1	4	1	0
Nit_verm	0	0	0	0	0	0	0	0	0	0	0	0
Nit_vitr	0	0	0	0	0	0	0	0	0	1	0	0
Nit_wuel	0	0	0	1	0	0	0	0	0	0	0	0
Ope_krum	0	0	0	0	0	0	0	0	0	0	0	0
Ope_muta	0	0	0	0	0	0	0	0	0	0	0	0
Par_prot	0	0	0	1	2	0	0	0	0	0	0	2
Pau_taen	0	0	0	1	0	0	0	0	0	0	0	0
Pet_hume	0	0	0	0	0	0	0	0	0	0	0	0
Pin_appe	2	0	3	0	0	0	0	0	0	0	0	0
Pin_dimi	0	0	0	0	0	0	0	0	0	0	0	0
Pin_kroc	0	0	0	0	0	0	0	0	0	0	0	0
Pin_micr	0	0	0	0	0	0	0	0	0	0	0	0
Plc_plac	0	0	0	1	0	0	0	0	0	0	0	0
Plt_cons	0	0	0	0	2	0	0	0	0	0	0	0
Plt_deli	1	0	1	1	2	2	1	1	0	6	1	3
Plt_disp	0	0	0	0	0	0	0	0	0	0	0	0

Plt_freq	1	0	1	3	20	27	2	0	0	6	0	1
Plt_hauc	3	10	9	29	4	1	0	0	0	0	0	0
Plt_lanc	0	0	0	0	0	0	0	0	0	0	0	0
Plt_lemm	0	0	0	0	4	0	0	0	0	0	0	0
Plt_link	0	0	0	0	0	0	0	0	0	0	0	0
Plt_peri	0	0	0	4	2	0	0	0	0	0	0	0
Plt_rost	0	0	0	1	0	0	1	0	0	0	0	0
Plt_sept	0	0	0	1	0	0	0	0	0	0	0	0
Psf_tene	0	0	0	1	0	0	0	0	0	0	0	0
Pss_brev	10	0	5	0	0	0	0	0	2	0	0	22
Pss_brin	1	7	3	3	20	0	0	3	0	44	22	56
Pss_elli	4	14	32	69	2	7	0	0	0	1	0	1
Pss_perm	0	0	0	0	0	0	0	0	0	0	0	0
Pss_robu	27	14	0	2	0	1	9	7	2	100	32	35
Pss_subs	0	0	0	0	0	0	0	0	0	0	0	0
Pss_zeil	0	0	0	4	6	0	0	0	0	1	0	5
Pst_bior	0	0	0	0	0	0	0	0	0	0	0	0
Pst_oblo	0	0	0	0	0	0	0	0	0	0	0	0
Pst_punc	0	0	0	0	0	0	0	0	0	0	0	0
Pst_ross	0	0	0	0	0	18	0	0	0	0	0	0
Pst_sacc	0	1	0	0	0	0	0	0	0	0	0	0
Pte_inan	0	0	0	0	0	0	0	0	0	0	0	0
Rei_sinu	2	0	0	0	0	0	0	0	0	0	0	0
Rho_abbr	11	8	7	5	10	18	6	0	2	3	9	13
Rhp_acum	0	0	0	0	0	0	0	0	0	0	0	0
Rhp_breb	0	0	0	0	0	0	0	0	0	0	0	0
Rhp_gibb	0	4	4	7	2	0	0	2	0	1	2	0
Sel_pupu	0	0	0	0	2	0	0	0	0	0	0	0
Sel_stro	0	0	0	0	0	0	2	0	0	0	0	0
Ske_cost	0	0	0	0	0	0	0	0	0	0	0	0
Ske_subs	0	0	0	0	0	0	0	0	0	0	0	0
Sla_lapp	0	0	0	0	0	0	0	0	0	0	0	0
Std_hant	0	0	0	0	0	0	0	0	0	0	0	0
Std_parv	0	0	0	0	0	0	0	0	0	0	0	0
Stn_legu	0	0	0	0	0	1	0	0	0	0	0	0
Stn_phoe	0	0	0	0	0	0	0	0	0	0	0	0
Stn_simu	0	0	0	0	0	0	0	0	0	0	0	0
Stn_smka	0	0	0	1	0	0	0	0	0	0	0	0
Stp_anus	0	0	0	0	0	0	0	0	0	0	0	0
Stp_wisl	0	0	0	0	0	0	0	0	0	0	0	0
Sts_punc	0	0	0	0	0	0	0	0	0	0	0	0

Sts_vent	0	0	0	0	0	0	0	0	0	0	0	0
Sur_angu	0	0	0	0	0	0	0	0	0	0	0	0
Sur_brba	0	0	0	0	2	1	2	0	0	1	1	0
Sur_breb	2	3	1	13	22	1	0	2	2	1	1	2
Sur_minu	0	0	0	0	0	0	0	0	0	0	0	0
Sur_oval	0	0	0	0	0	0	0	0	0	0	0	0
Syn_acus	7	2	8	2	2	0	0	0	1	5	0	0
Syn_ulna	0	0	1	0	14	1	0	3	0	0	0	0
Tab_fasc	0	3	0	1	0	0	0	0	0	0	0	2
Tab_kten	0	0	0	0	0	0	0	0	0	0	0	0
Tab_laev	0	0	0	0	0	0	0	0	0	0	0	0
Tab_tabu	2	0	0	0	0	0	0	0	0	0	0	3
Tab_waer	0	0	0	0	0	0	0	0	0	0	0	0
Tal_fene	0	0	0	0	2	0	0	0	0	0	0	0
Tal_floc	0	0	0	0	2	0	0	0	0	0	0	0
Tet_glan	0	0	0	0	0	0	0	0	0	0	0	0
Tha_balt	1	0	0	0	0	0	0	1	0	0	0	0
Tha_hyla	0	0	0	0	0	0	0	0	0	0	0	0
Tha_leva	0	0	0	0	0	0	0	0	0	0	0	0
Tha_pros	0	0	0	0	0	0	0	0	0	0	0	0
Tha_pseu	0	0	0	0	0	0	0	0	0	0	0	0
Tha_punc	0	0	0	0	2	0	0	0	0	0	0	0
Try_angu	0	0	0	2	0	0	1	0	0	0	0	0
Try_apic	0	0	0	1	0	0	0	0	0	0	0	0
Try_ardu	0	0	0	0	0	0	0	0	0	0	0	0
Try_hung	0	0	0	0	0	0	0	1	0	0	0	0
Try_levi	0	0	0	1	0	0	0	0	0	0	0	0
Try_lite	0	0	0	0	0	0	0	0	0	0	0	0
Try_sali	0	0	0	0	0	0	0	0	0	0	0	0
Try_scal	0	0	0	0	0	0	0	0	0	1	0	0
