# Supplementary material for: Diatom Cell Size, Coloniality and Motility: Trade-Offs between Temperature, Salinity and Nutrient Supply with Climate Change
Source: PLoS One. 2014 Oct 3;9(10):e109993. doi: 10.1371/journal.pone.0109993 (PMC4184900; doi:10.1371/journal.pone.0109993)
Supplement: Table S5 — Ordinal scales used to estimate the magnitude of some environmental and biotic variables in the temperature and salinity gradients. (PDF) [file pone.0109993.s005.pdf]

Table S5. Ordinal scales used to estimate the magnitude of some environmental and biotic variables in the temperature and salinity gradients.

Temperature gradient		Salinity gradient	
FLOW	MACCOV	EXP	BEACH
Water movement flow factor	Macroalgal cover on the stones	Exposure to wave action	Type of substratum of the terrestrial part of the beach (not covered by water)
1 = Stagnant water<br>2 = Quiescent water<br>3 = Exposed to natural waves<br>4 = Slow unidirectional flow<br>5 = Unidirectional flow<br>6 = Rapid unidirectional flow	1 = < 1%, 1-5 individuals<br>2 = < 1%, 6-10 individuals<br>3 = < 1%, >10 individuals<br>4 = 1-5%<br>5 = 6-10%<br>6 = 11-25%<br>7 = 26-50%<br>8 = 51-75%<br>9 = 76-100%	1 = Stagnant water<br>2 = Very little exposed<br>3 = Little exposed<br>4 = Medium exposed<br>5 = Highly exposed	1 = >90% sand<br>2 = >25% sand and >25% stones<br>3 = >90% stones of <50 cm in size<br>4 = >90% stones of >50 cm in size<br>5 = >25% stones and >25% solid rock<br>6 = >90% solid rock
