# Supplementary material for: Diatom Cell Size, Coloniality and Motility: Trade-Offs between Temperature, Salinity and Nutrient Supply with Climate Change
Source: PLoS One. 2014 Oct 3;9(10):e109993. doi: 10.1371/journal.pone.0109993 (PMC4184900; doi:10.1371/journal.pone.0109993)
Supplement: Table S6 — List of the 405 diatom taxa included in the analyses. BMB nr = number under which photomicrographs and ecological information can be found in publications 39–43. Temp = temperature gradient data set, Salin = Salinity gradient data set, Freq = average frequency in 1000 valves, Abund = average abundance in 1000 valves, Salinity group = distribution in the Baltic Sea area (M = marine, B = brackish, F = freshwater), Euryhal group = Euryhalinity according to the species' salinity range in the Baltic Sea area after [39]–[43] (M = medium, L = large, XL = very large, XXL = extremely large). For further definitions of traits, see “Assignment of traits to the diatom taxa” in the “Materials and Methods” section. (PDF) [file pone.0109993.s006.pdf]

Table S6. List of the 405 diatom taxa included in the analyses. BMB nr = number under which photomicrographs and ecological information can be found in publications 39-43. Temp = temperature gradient data set, Salin = Salinity gradient data set, Freq = average frequency in 1000 valves, Abund = average abundance in 1000 valves, Salinity group = distribution in the Baltic Sea area (M = marine, B = brackish, F = freshwater), Euryhal group = Euryhalinity according to the species' salinity range in the Baltic Sea area after [39-43] (M = medium, L = large, XL = very large, XXL = extremely large). For further definitions of traits, see "Assignment of traits to the diatom taxa" in the "Materials and Methods" section.

Diatom taxon	BMB nr	Temp Freq	Temp Abund	Salin Freq	Salin Abund	Salinity group	Euryhal group	Life form	Motility	Colony form	Attachm type	Colony height	Length µm	Surface µm2	Volume µm3	S:V ratio	Shape
Achnanthes bicapitata Hustedt	-	1	0.02	-	-	MB	M	Attached solitary	Creeping	None	Adnate	Low	10	116	62	1.87	2.6
Achnanthes brevipes C.A. Agardh	101	21	0.32	2	0.076	BM	L	Attached colony	Attached	Ribbon	Short stalk	High	51	3495	12898	0.27	2.7
Achnanthes brevipes var. intermedia (Kützing) Cleve	2	46	4.95	6	0.134	MB	L	Attached colony	Attached	Ribbon	Short stalk	High	36	1875	5290	0.35	2.6
Achnanthes fogedii Håkansson	302	-	-	5	0.084	FB	M	Attached solitary	Creeping	None	Adnate	Low	8	110	95	1.16	1.7
Achnanthes leonardii Witkowski & Lange-Bertalot	-	-	-	1	0.176	MB	M	Attached solitary	Creeping	None	Adnate	Low	10	116	62	1.87	2.6
Achnanthes longipes C.A. Agardh	4	1	0.01	1	0.017	B	XL	Attached colony	Attached	Ribbon	Short stalk	High	77	7638	41618	0.18	2.9
Achnanthes lutheri Hustedt	-	-	-	1	0.008	FB	M	Attached solitary	Creeping	None	Adnate	Low	9	117	96	1.23	2.0
Achnanthes minuscula Hustedt	307	77	3.25	29	1.084	FB	XXL	Attached solitary	Creeping	None	Adnate	Low	9	86	44	1.95	2.3
Achnanthes vistulana Witkowski	405	11	0.14	16	0.639	B	M	Attached colony	Attached	Ribbon	Short stalk	High	9	67	26	2.58	3.7
Achnanthidium exile (Kützing) Heiberg	-	-	-	2	0.025	B	M	Attached colony	Attached	Ribbon	Short stalk	High	13	123	72	1.70	4.0
Achnanthidium minutissimum (Kützing) Czarnecki	406	83	4.54	81	64.504	FBM	XXL	Attached colony	Attached	Ribbon	Short stalk	High	15	141	77	1.83	5.4
Achnanthidium pyrenaicum (Hustedt) Kobayasi	-	10	0.14	1	0.017	MB	XL	Attached colony	Attached	Ribbon	Short stalk	High	13	123	72	1.70	4.0
Achnanthidium subatomoides (Hustedt) Monnier, Lange-Bertalot & Ector	-	5	0.08	-	-	BF	L	Attached solitary	Creeping	None	Adnate	Low	8	110	92	1.20	1.8
Actinocyclus normanii fo. subsalsus (Juhlin-Dannfelt) Hustedt	106	-	-	1	0.008	FB	L	Floating solitary	Floating	None	None	Low	26	2123	6899	0.31	1.0
Actinocyclus octonarius var. crassus (W. Smith) Hendey	206	3	0.03	1	0.025	BF	XL	Floating solitary	Floating	None	None	Low	31	3018	11693	0.26	1.0
Amicula specululum (Witkowski) Witkowski	-	-	-	1	0.050	BM	L	Motile solitary	Motile	None	None	Low	12	126	74	1.70	3.8
Amphora commutata Grunow	107	2	0.02	-	-	B	L	Motile solitary	Motile	None	None	Low	57	3260	10115	0.32	2.2
Amphora copulata (Kützing) Schoeman & Archibald	7	18	0.28	48	0.840	FB	XL	Motile solitary	Motile	None	None	Low	30	1170	2498	0.47	1.7
Amphora flebilis Simonsen	-	103	8.16	24	0.782	B	L	Motile solitary	Motile	None	None	Low	15	155	87	1.78	3.2
Amphora helenensis Giffen	409	-	-	5	0.445	MB	L	Motile solitary	Motile	None	None	Low	10	132	95	1.40	1.7
Amphora inariensis Krammer	-	6	0.08	8	0.496	FB	L	Motile solitary	Motile	None	None	Low	16	376	420	0.90	1.6
Amphora lineolata Ehrenberg	108	9	0.14	20	0.437	B	XL	Motile solitary	Motile	None	None	Low	44	1631	3308	0.49	2.6
Amphora micrometra Giffen	214	2	0.05	5	0.126	BM	XL	Motile solitary	Motile	None	None	Low	8	93	57	1.64	1.6
Amphora ovalis (Kützing) Kützing	9	3	0.06	4	0.076	FB	XL	Motile solitary	Motile	None	None	Low	49	3323	12172	0.27	1.6
Amphora pediculus (Kützing) Grunow	10	110	6.13	88	8.639	FB	XXL	Motile solitary	Motile	None	None	Low	11	147	110	1.33	1.7
Amphora profusa Giffen	-	-	-	2	0.050	MB	L	Motile solitary	Motile	None	None	Low	29	589	639	0.92	3.2
Amphora staurophora Juhlin-Dannfelt	318	1	0.02	-	-	BM	XL	Motile solitary	Motile	None	None	Low	16	233	189	1.23	2.3
Aneumastus minor (Hustedt) Lange-Bertalot	217	-	-	14	0.168	FB	L	Motile solitary	Motile	None	None	Low	19	573	890	0.64	2.1
Aneumastus tuscula (Ehrenberg) D.G. Mann & Stickle	319	3	0.02	4	0.042	FB	M	Motile solitary	Motile	None	None	Low	37	1820	4662	0.39	2.5
Anomoeoneis sphaerophora var. sculpta (Ehrenberg) O. Müller	110	-	-	1	0.017	FB	L	Motile solitary	Motile	None	None	Low	115	12800	73959	0.17	3.3

Astartiella bahusiensis (Grunow) Witkowski, Lange-Bertalot & Metzeltin	201	11	0.19	2	0.084	FB	L	Attached solitary	Creeping	None	Adnate	Low	19	310	280	1.11	3.1
Asterionella formosa Hassall	11	2	0.02	2	0.017	FB	M	Floating colony	Floating	Zigzag	None	Medium	63	542	265	2.05	31.5
Aulacoseira Thwaites spp.	113	17	0.32	9	0.134	FB	L	Floating colony	Floating	Chain	None	Medium	21	1342	3709	0.36	1.4
Bacillaria paxillifer (O.F. Müller) Marsson	12	75	17.78	5	0.084	FBM	XXL	Motile colony	Motile	Ribbon	None	Medium	91	2020	2801	0.72	15.2
Berkeleya fennica Juhlin-Dannfelt	418	-	-	69	8.151	B	XL	Attached colony	Motile	Tube	Tube	High	18	197	123	1.60	5.2
Berkeleya rutilans (Trentepohl) Grunow	13	116	46.16	70	22.395	B	XXL	Attached colony	Motile	Tube	Tube	High	24	412	400	1.03	5.0
Berkeleya scopulorum (Brébisson) Cox	-	-	-	1	0.118	MB	L	Attached colony	Motile	Tube	Tube	High	110	3509	6988	0.50	10.0
Biremis lucens (Hustedt) Sabbe, Witkowski & Vijverman	324	-	-	2	0.034	MB	L	Attached colony	Attached	Ribbon	Pad	Low	17	257	220	1.17	4.4
Brachysira aponina Kützing	14	13	1.54	19	0.689	B	L	Attached solitary	Attached	None	Pad	Medium	26	413	335	1.23	5.2
Brachysira vitrea (Grunow) Ross	419	-	-	24	3.092	FB	L	Attached solitary	Attached	None	Pad	Medium	23	390	341	1.14	4.2
Brebissonia lanceolata (C.A. Agardh) Mahoney & Reimer	15	32	1.35	9	0.134	FB	XL	Attached colony	Attached	Bush	Stalk	High	97	6454	21773	0.30	4.9
Caloneis aemula (A. Schmidt) Cleve	420	-	-	1	0.008	FB	L	Motile solitary	Motile	None	None	Low	30	726	999	0.73	4.4
Caloneis amphisbaena (Bory) Cleve	219	-	-	1	0.008	FB	XL	Motile solitary	Motile	None	None	Low	75	6900	28172	0.24	2.7
Caloneis bacillum (Grunow) Cleve	421	40	0.82	15	0.202	FB	L	Motile solitary	Motile	None	None	Low	17	273	247	1.11	3.1
Caloneis crassa (Gregory) Ross	325	-	-	2	0.025	MB	L	Motile solitary	Motile	None	None	Low	65	5756	26536	0.22	2.4
Caloneis silicula (Ehrenberg) Cleve	-	-	-	3	0.025	FB	M	Motile solitary	Motile	None	None	Low	36	626	680	0.92	6.0
Campylodiscus clypeus (Ehrenberg) Ehrenberg	17	-	-	3	0.025	FBM	XXL	Motile solitary	Motile	None	None	Low	143	44170	263179	0.17	1.0
Catenula adhaerens (Mereschkowsky) Mereschkowsky	19	-	-	6	0.118	BM	XL	Attached colony	Attached	Ribbon	Pad	Low	14	94	36	2.63	6.4
Cavinula lapidosa (Krasske) Lange-Bertalot	-	-	-	1	0.034	FB	M	Motile solitary	Motile	None	None	Low	16	396	501	0.79	2.0
Cavinula pseudoscutiformis (Hustedt) D.G. Mann & Stickle	220	-	-	1	0.008	FB	L	Motile solitary	Motile	None	None	Low	14	481	753	0.64	1.3
Ceratoneis closterium Ehrenberg	33	72	4.86	2	0.092	MB	XXL	Motile solitary	Motile	None	None	Low	50	423	158	2.68	18.0
Chaetoceros Ehrenberg spp.	22	100	9.48	-	-	MB	XXL	Floating colony	Floating	Chain	None	Medium	12	390	574	0.68	1.4
Chamaepinnularia clamans (Hustedt) Witkowski, Lange-Bertalot & Metzeltin	251	-	-	1	0.017	MB	M	Motile solitary	Motile	None	None	Low	13	307	342	0.90	2.2
Chamaepinnularia margaritiana (Witkowski) Witkowski	362	54	1.44	22	0.261	B	L	Motile solitary	Motile	None	None	Low	7	63	29	2.16	2.6
Cocconeis costata Gregory	334	-	-	6	0.235	FB	M	Attached solitary	Creeping	None	Adnate	Low	17	358	306	1.17	1.8
Cocconeis neodiminuta Krammer	-	93	2.88	-	-	FB	XXL	Attached solitary	Creeping	None	Adnate	Low	9	121	65	1.86	1.5
Cocconeis neothumensis Krammer	425	38	1.06	62	3.437	FB	XL	Attached solitary	Creeping	None	Adnate	Low	10	96	41	2.34	2.0
Cocconeis pediculus Ehrenberg	23	97	8.05	85	5.042	MB	L	Attached solitary	Creeping	None	Adnate	Low	26	1140	1722	0.66	1.3
Cocconeis peltoides Hustedt	119	8	0.09	2	0.034	FBM	XXL	Attached solitary	Creeping	None	Adnate	Low	13	255	196	1.30	1.5
Cocconeis placentula Ehrenberg	24	113	8.17	81	3.303	FB	XXL	Attached solitary	Creeping	None	Adnate	Low	20	471	465	1.01	1.8
Cocconeis placentula var. euglypta (Ehrenberg) Grunow	426	-	-	19	1.185	FB	M	Attached solitary	Creeping	None	Adnate	Low	15	300	242	1.24	1.7
Cocconeis placentula var. lineata (Ehrenberg) van Heurck	-	-	-	2	0.034	FB	L	Attached solitary	Creeping	None	Adnate	Low	20	471	465	1.01	1.8
Cocconeis pseudolineata (Geitler) Lange-Bertalot	-	-	-	3	0.034	FB	M	Attached solitary	Creeping	None	Adnate	Low	9	121	65	1.86	1.5
Cocconeis pseudothumensis Reichardt	-	21	0.26	1	0.008	MB	XL	Attached solitary	Creeping	None	Adnate	Low	9	121	65	1.86	1.5
Cocconeis scutellum Ehrenberg	25	25	0.40	20	4.395	MB	L	Attached solitary	Creeping	None	Adnate	Low	18	499	537	0.93	1.5
Cocconeis stauroneiformis (W. Smith) Okuno	27	-	-	10	1.992	B	XXL	Attached solitary	Creeping	None	Adnate	Low	19	481	496	0.97	1.6
Conticribra guillardii (Hasle) Stachura-Suchoples & Williams	96	8	0.15	-	-	MB	L	Floating colony	Floating	Chain	None	Medium	9	249	277	0.90	1.0
Conticribra weissflogii (Grunow) Stachura-Suchoples & Williams	398	1	0.01	-	-	MB	M	Floating colony	Floating	Chain	None	Medium	15	688	1272	0.54	1.0
Coscinodiscus asteromphalus Ehrenberg	223	-	-	1	0.017	FBM	XXL	Floating solitary	Floating	None	None	Low	174	95067	2067699	0.05	1.0
Cosmioneis pusilla (W. Smith) D.G. Mann & Stickle	225	-	-	1	0.008	B	L	Motile solitary	Motile	None	None	Low	39	2391	7045	0.34	2.0

Craticula cuspidata (Kützing) D.G. Mann	428	2	0.02	-	-	FB	M	Motile solitary	Motile	None	None	Low	110	8375	36086	0.23	4.1
Ctenophora pulchella (Ralfs) Williams & Round	29	77	4.02	95	14.437	FB	XL	Attached colony	Attached	Bush	Pad	Medium	97	2476	3535	0.70	13.7
Cyclostephanos dubius (Fricke) Round	30	-	-	4	0.067	FB	M	Floating solitary	Floating	None	None	Low	12	445	661	0.67	1.0
Cyclotella atomus Hustedt	226	14	0.22	4	0.151	FB	L	Floating solitary	Floating	None	None	Low	5	92	62	1.48	1.0
Cyclotella choctawhatcheeana Prasad	31	82	3.40	11	0.378	MB	XL	Floating colony	Floating	Chain	None	Medium	6	109	81	1.36	1.0
Cyclotella meneghiniana Kützing	32	8	0.08	9	0.193	FB	XL	Floating solitary	Floating	None	None	Low	20	1243	3093	0.40	1.0
Cyclotella radiosa (Grunow) Lemmermann	227	3	0.03	4	0.059	FB	L	Floating solitary	Floating	None	None	Low	20	1256	3140	0.40	1.0
Cyclotella rossii Håkansson	-	-	-	5	0.092	FB	L	Floating solitary	Floating	None	None	Low	11	612	1047	0.58	1.0
Cyclotella stelligera Cleve & Grunow	337	5	0.06	2	0.017	FB	M	Floating solitary	Floating	None	None	Low	9	254	286	0.89	1.0
Cyclotella striata (Kützing) Grunow	-	1	0.02	1	0.017	B	M	Floating solitary	Floating	None	None	Low	13	547	903	0.61	1.0
Cyclotella tripartita Håkansson	-	-	-	1	0.059	B	M	Floating solitary	Floating	None	None	Low	8	191	186	1.03	1.0
Cylindrotheca gracilis (Brébisson) Grunow	-	2	0.03	-	-	FBM	XXL	Motile solitary	Motile	None	None	Low	215	925	415	2.23	41.0
Cymatopleura solea (Brébisson) W. Smith	122	-	-	1	0.008	FB	M	Motile solitary	Motile	None	None	Low	78	4108	10359	0.40	5.1
Cymbella affinis Kützing	228	2	0.03	38	5.101	FB	M	Attached colony	Attached	Bush	Stalk	High	31	835	1169	0.71	3.5
Cymbella cistula (Ehrenberg) Kirchner	123	13	0.25	5	0.050	FB	L	Attached colony	Attached	Bush	Stalk	High	70	4020	11960	0.34	3.7
Cymbella helvetica Kützing	124	-	-	40	2.924	FB	M	Attached colony	Attached	Bush	Stalk	High	62	2720	6146	0.44	4.4
Cymbella lanceolata (C.Agardh) Kirchner	34	10	0.22	21	0.235	FB	L	Attached colony	Attached	Bush	Stalk	High	128	10740	46475	0.23	4.7
Cymbella proxima Reimer	-	-	-	1	0.008	FB	L	Attached colony	Attached	Bush	Stalk	High	78	4585	16933	0.27	3.8
Cymbopleura amphicephala (Nägeli) Krammer	-	1	0.01	-	-	FB	XL	Motile solitary	Motile	None	None	Low	27	646	965	0.67	3.3
Denticula creticola (Østrup) Lange Bertalot & Krammer	125	35	1.86	-	-	B	M	Attached colony	Attached	Ribbon	Pad	Low	13	147	91	1.61	4.6
Denticula sundayensis Archibald	338	7	0.21	-	-	B	M	Attached colony	Attached	Ribbon	Pad	Low	12	77	32	2.40	5.0
Denticula tenuis var. crassula (Nägeli) Hustedt	429	-	-	36	11.218	FB	M	Attached colony	Attached	Ribbon	Pad	Low	20	410	423	0.97	3.6
Diatoma bottnica Snoeijs	229	-	-	38	2.067	B	XL	Attached colony	Attached	Zigzag	Pad	High	57	1852	2841	0.65	7.0
Diatoma moniliforme (Kützing) Williams	36	120	76.12	111	238.992	FBM	XXL	Attached colony	Attached	Zigzag	Pad	High	26	352	269	1.31	7.6
Diatoma problematicum Lange-Bertalot	-	-	-	1	0.008	FB	M	Attached colony	Attached	Zigzag	Pad	High	10	157	161	0.98	1.7
Diatoma tenuis C.A. Agardh	37	82	6.56	9	0.185	FB	XL	Attached colony	Attached	Zigzag	Pad	High	70	959	749	1.28	21.8
Diatoma vulgaris Bory	38	60	11.20	23	1.269	FB	XL	Attached colony	Attached	Zigzag	Pad	High	30	1073	1958	0.55	3.0
Dickieia subinflata (Grunow) D.G. Mann	169	-	-	2	0.101	BM	L	Motile solitary	Motile	None	None	Low	21	516	720	0.72	3.0
Diploneis decipiens var. parallela A. Cleve	431	-	-	1	0.017	MB	L	Motile solitary	Motile	None	None	Low	46	3538	13163	0.27	1.8
Diploneis didyma (Ehrenberg) Ehrenberg	39	-	-	1	0.034	FBM	XXL	Motile solitary	Motile	None	None	Low	42	2944	9901	0.30	2.0
Diploneis domblittensis (Grunow) Cleve	231	1	0.02	-	-	B	L	Motile solitary	Motile	None	None	Low	23	1079	2258	0.48	1.6
Diploneis elliptica (Kützing) Cleve	-	-	-	2	0.042	FBM	XXL	Motile solitary	Motile	None	None	Low	60	5404	23479	0.23	2.1
Diploneis interrupta (Kützing) Cleve	40	-	-	3	0.034	B	XL	Motile solitary	Motile	None	None	Low	42	2980	8737	0.34	2.0
Diploneis oculata (Brébisson) Cleve	232	-	-	2	0.025	FB	L	Motile solitary	Motile	None	None	Low	17	364	371	0.98	2.6
Diploneis smithii (Brébisson) Cleve	127	7	0.10	-	-	FB	XL	Motile solitary	Motile	None	None	Low	60	5404	23479	0.23	2.1
Diploneis smithii var. dilatata (M. Peragallo) Terry	128	-	-	4	0.067	FBM	XXL	Motile solitary	Motile	None	None	Low	37	2569	8045	0.32	1.7
Diploneis smithii var. pumila (Grunow) Hustedt	433	1	0.01	7	0.067	BF	L	Motile solitary	Motile	None	None	Low	21	827	1452	0.57	1.8
Diploneis smithii var. rhombica Mereschkowsky	41	1	0.02	4	0.050	B	L	Motile solitary	Motile	None	None	Low	46	3538	13163	0.27	1.8
Diploneis subovalis Cleve	-	-	-	1	0.008	FB	M	Motile solitary	Motile	None	None	Low	43	1972	5712	0.35	2.7
Encyonema caespitosum Kützing	42	14	0.36	34	0.571	FB	L	Attached colony	Motile	Tube	Tube	High	29	1020	1869	0.55	2.6

Encyonema lacustre (C.A. Agardh) Mills	43	3	0.22	17	0.445	FB	L	Attached colony	Motile	Tube	Tube	High	39	1525	3037	0.50	3.8
Encyonema minutum (Hilse) D.G. Mann	-	-	-	12	0.689	FBM	XXL	Attached colony	Motile	Tube	Tube	High	20	311	310	1.00	3.5
Encyonema obscurum (Krasske) D.G. Mann	-	-	-	1	0.008	FB	M	Attached colony	Motile	Tube	Tube	High	24	598	709	0.84	3.2
Encyonema prostratum (Berkeley) Kützing	233	-	-	6	0.076	FB	L	Attached colony	Motile	Tube	Tube	High	48	2971	8806	0.34	2.7
Encyonema silesiacum (Bleisch) D.G. Mann	234	1	0.01	20	2.160	FB	L	Attached colony	Motile	Tube	Tube	High	24	598	709	0.84	3.2
Encyonema tenuissimum (Hustedt) D.G. Mann	-	-	-	1	0.008	FB	M	Attached colony	Motile	Tube	Tube	High	24	598	709	0.84	3.2
Encyonopsis behrei (Foged) Krammer & Metzeltin	435	-	-	1	0.008	FB	M	Motile solitary	Motile	None	None	Low	25	761	1205	0.63	2.8
Encyonopsis krammeri Reichardt	-	-	-	31	5.059	FB	M	Motile solitary	Motile	None	None	Low	16	170	112	1.51	4.4
Entomoneis costata (Hustedt) Reimer	-	2	0.04	-	-	B	M	Motile solitary	Motile	None	None	Low	29	1664	2353	0.71	1.9
Entomoneis gigantea (Grunow) Nizamuddin	-	4	0.03	-	-	MB	M	Motile solitary	Motile	None	None	Low	70	9107	29523	0.31	2.0
Entomoneis paludosa (W. Smith) Reimer	235	94	3.89	32	0.412	FBM	XXL	Motile solitary	Motile	None	None	Low	62	7962	25592	0.31	1.8
Entomoneis pseudoduplex Osada & Kobayasi	236	5	0.06	12	0.176	B	XL	Motile solitary	Motile	None	None	Low	29	1664	2353	0.71	1.9
Entomoneis punctulata (Grunow) Osada & Kobayasi	237	-	-	3	0.025	MB	L	Motile solitary	Motile	None	None	Low	64	7872	28180	0.28	1.8
Entomoneis surirelloides (Hendey) Poulin, Berard-Therriault & Cardinal 1987	-	18	0.18	-	-	B	M	Motile solitary	Motile	None	None	Low	70	9107	29523	0.31	2.0
Epithemia adnata (Kützing) Brébisson	132	23	0.34	20	0.891	FB	XL	Attached solitary	Creeping	None	Adnate	Low	42	1913	4701	0.41	2.0
Epithemia goeppertiana Hilse	-	-	-	2	0.017	BF	L	Attached solitary	Creeping	None	Adnate	Low	35	905	1112	0.81	3.2
Epithemia sorex Kützing	44	98	7.93	78	5.092	FB	XL	Attached solitary	Creeping	None	Adnate	Low	26	942	1866	0.50	1.6
Epithemia turgida (Ehrenberg) Kützing	238	-	-	41	0.756	FB	XL	Attached solitary	Creeping	None	Adnate	Low	74	5242	20141	0.26	2.3
Epithemia turgida var. westermannii (Ehrenberg) Grunow	45	36	0.71	27	1.084	FBM	XXL	Attached solitary	Creeping	None	Adnate	Low	67	5234	22046	0.24	1.9
Eucocconeis laevis (Østrup) Lange-Bertalot	-	-	-	7	0.101	FB	L	Attached solitary	Creeping	None	Adnate	Low	14	254	287	0.88	2.2
Eunotia arcus Ehrenberg	-	-	-	2	0.034	FB	L	Floating colony	Floating	Ribbon	None	Medium	48	827	898	0.92	7.9
Eunotia pectinalis var. undulata (Ralfs) Rabenhorst	-	-	-	1	0.008	FB	M	Motile solitary	Motile	None	None	Low	90	1958	2658	0.74	12.0
Fallacia clepsidroides Witkowski	239	-	-	25	0.681	BM	XXL	Motile solitary	Motile	None	None	Low	10	153	101	1.52	2.1
Fallacia cryptolyra (Brockmann) Stickle & D.G. Mann	240	31	0.55	1	0.017	FB	XL	Motile solitary	Motile	None	None	Low	11	197	162	1.22	1.8
Fallacia florinae (M. Møller) Witkowski	341	-	-	4	0.092	MB	L	Motile solitary	Motile	None	None	Low	10	162	117	1.39	1.9
Fallacia pseudolitoricola (Håkansson) Håkansson	133	1	0.02	-	-	B	M	Motile solitary	Motile	None	None	Low	10	153	101	1.52	2.1
Fallacia pseudony (Hustedt) D.G. Mann	343	-	-	1	0.008	BM	L	Motile solitary	Motile	None	None	Low	10	180	148	1.22	1.8
Fallacia pygmaea (Kützing) Stickle & D.G. Mann	134	3	0.06	9	0.118	BF	XL	Motile solitary	Motile	None	None	Low	27	841	1228	0.68	2.4
Fistulifera saprophila (Lange-Bertalot & Bonik) Lange-Bertalot	-	-	-	2	0.017	FB	M	Motile solitary	Motile	None	None	Low	7	73	29	2.51	2.6
Fragilaria amicorum Witkowski & Lange-Bertalot	135	104	9.15	1	0.076	B	XL	Attached solitary	Attached	None	Pad	Medium	6	64	36	1.79	2.1
Fragilaria capucina Desmazières	136	-	-	10	0.378	FB	M	Floating colony	Floating	Ribbon	None	Medium	34	490	397	1.23	9.4
Fragilaria capucina subsp. rumpens (Kützing) Lange-Bertalot	-	-	-	68	17.185	FB	XXL	Floating colony	Floating	Ribbon	None	Medium	39	416	277	1.50	9.9
Fragilaria capucina var. vaucheriae (Kützing) Lange-Bertalot	244	22	0.35	43	15.504	FB	XL	Floating colony	Floating	Ribbon	None	Medium	18	202	123	1.64	6.3
Fragilaria construens (Ehrenberg) Grunow	488	56	8.01	1	0.025	FB	L	Attached colony	Attached	Ribbon	Pad	Low	13	358	399	0.90	1.6
Fragilaria gedanensis Witkowski	137	-	-	22	1.101	B	L	Attached colony	Attached	Ribbon	Pad	Low	9	68	29	2.38	4.5
Fragilaria gracilis Østrup	-	-	-	29	3.630	FB	L	Floating colony	Floating	Ribbon	None	Medium	13	358	399	0.90	1.6
Fragilaria hyalina var. durietzii Cleve-Euler	46	1	0.02	7	1.420	B	L	Attached colony	Attached	Zigzag	Pad	High	26	746	1025	0.73	3.2
Fragilaria nitzschioides Grunow	-	-	-	2	0.034	MB	M	Floating colony	Floating	Ribbon	None	Medium	37	443	336	1.32	8.1
Fragilaria striatula Lyngbye	48	80	8.69	-	-	BF	XL	Floating colony	Floating	Ribbon	None	Medium	38	911	1238	0.74	5.1
Fragilariopsis cylindrus (Grunow) Krieger	138	2	0.02	24	0.403	FB	L	Floating colony	Floating	Ribbon	None	Medium	15	227	190	1.20	4.3

Frustulia creuzburgensis (Krasske) Hustedt	440	3	0.05	-	-	FB	M	Attached colony	Motile	Tube	Tube	High	33	873	1310	0.67	4.7
Frustulia vulgaris (Thwaites) De Toni	-	-	-	1	0.008	BM	XL	Attached colony	Motile	Tube	Tube	High	55	1695	3285	0.52	4.7
Gomphoneis exigua (Kützing) Medlin	50	2	0.03	32	1.866	B	L	Attached solitary	Attached	None	Pad	Medium	16	192	124	1.54	4.2
Gomphonema clavatum Ehrenberg	-	-	-	6	0.126	FB	M	Attached colony	Attached	Bush	Stalk	High	55	2119	4043	0.52	3.9
Gomphonema intricatum Kützing	-	-	-	36	1.849	FB	L	Attached colony	Attached	Bush	Stalk	High	55	2119	4043	0.52	3.9
Gomphonema olivaceum (Hornemann) Brébisson	49	92	21.48	102	8.866	FB	XL	Attached colony	Attached	Bush	Stalk	High	34	1424	3156	0.45	2.9
Gomphonema parvulum (Kützing) Kützing	139	1	0.01	6	0.176	FB	XL	Attached colony	Attached	Bush	Stalk	High	22	607	925	0.66	2.6
Gomphonemopsis exigua var. platypus (Østrup) Snoeijs	443	-	-	1	0.151	MB	XXL	Attached solitary	Attached	None	Pad	Medium	18	301	269	1.12	3.7
Gomphonemopsis obscura (Krasske) Lange-Bertalot	-	-	-	20	0.739	B	L	Attached solitary	Attached	None	Pad	Medium	16	192	124	1.54	4.2
Gomphonemopsis pseudexigua (Simonsen) Medlin	140	57	1.63	-	-	MB	L	Attached solitary	Attached	None	Pad	Medium	11	120	71	1.68	2.9
Grammatophora marina (Lyngbye) Kützing	345	-	-	1	0.017	MB	XL	Attached colony	Attached	Zigzag	Pad	High	47	4188	15418	0.27	1.7
Grammatophora oceanica Ehrenberg	141	2	0.03	11	0.714	FB	L	Attached colony	Attached	Zigzag	Pad	High	25	1593	4126	0.39	1.3
Gyrosigma acuminatum (Kützing) Rabenhorst	142	-	-	6	0.151	FB	L	Motile solitary	Motile	None	None	Low	145	6310	15590	0.40	9.1
Gyrosigma attenuatum (Kützing) Rabenhorst	143	-	-	3	0.034	B	M	Motile solitary	Motile	None	None	Low	190	12403	45965	0.27	7.9
Gyrosigma eximium (Thwaites) Boyer	145	1	0.02	-	-	B	L	Attached colony	Motile	Tube	Tube	High	69	2422	4694	0.52	6.2
Gyrosigma fasciola (Ehrenberg) Griffith & Henfrey	347	2	0.02	-	-	B	M	Motile solitary	Motile	None	None	Low	83	2558	3293	0.78	7.2
Gyrosigma strigilis (W. Smith) Cleve	146	-	-	1	0.008	B	M	Motile solitary	Motile	None	None	Low	362	34570	187367	0.18	10.3
Gyrosigma tenuissimum (W.Smith) Griffith & Henfrey	-	-	-	1	0.008	B	L	Motile solitary	Motile	None	None	Low	40	1080	1820	0.59	4.0
Halamphora acutiuscula (Kützing) Levkov	209	56	1.41	-	-	B	XXL	Motile solitary	Motile	None	None	Low	31	857	1267	0.68	2.5
Halamphora coffeaeformis (C.A. Agardh) Levkov	210	102	10.68	50	2.286	MB	XL	Motile solitary	Motile	None	None	Low	29	589	639	0.92	3.2
Halamphora exigua (Gregory) Levkov	315	27	0.76	4	0.050	B	XL	Motile solitary	Motile	None	None	Low	27	778	1216	0.64	2.1
Halamphora holsatica (Hustedt) Levkov	211	13	0.18	4	0.042	B	L	Motile solitary	Motile	None	None	Low	37	1168	2020	0.58	2.5
Halamphora hybrida (Grunow) Levkov	8	4	0.06	3	0.034	BM	XXL	Motile solitary	Motile	None	None	Low	37	1383	2789	0.50	2.2
Halamphora luciae (Cholnoky) Levkov	411	1	0.01	19	0.546	FB	M	Motile solitary	Motile	None	None	Low	21	323	270	1.20	3.0
Halamphora normanii (Rabenhorst) Levkov	-	-	-	1	0.034	B	M	Motile solitary	Motile	None	None	Low	25	273	142	1.93	5.4
Halamphora subholsatica (Krammer) Levkov	413	26	0.48	1	0.017	MB	XXL	Motile solitary	Motile	None	None	Low	41	1443	2755	0.52	2.6
Halamphora tenerrima (Aleem & Hustedt) Levkov	414	68	2.84	45	1.748	FB	XL	Motile solitary	Motile	None	None	Low	14	177	120	1.47	2.5
Halamphora veneta (Kützing) Levkov	216	2	0.02	38	1.487	B	L	Motile solitary	Motile	None	None	Low	16	250	215	1.16	2.2
Hantzschia baltica Simonsen	-	-	-	4	0.050	FBM	XXL	Motile solitary	Motile	None	None	Low	77	2847	5455	0.52	7.6
Haslea spicula (Hickie) Lange-Bertalot	188	69	2.75	11	0.143	FB	L	Motile solitary	Motile	None	None	Low	85	1878	2347	0.80	10.9
Hippodonta capitata (Ehrenberg) Lange-Bertalot, Metzeltin & Witkowski	-	-	-	5	0.277	FB	L	Motile solitary	Motile	None	None	Low	17	251	239	1.05	3.1
Hippodonta hungarica (Grunow) Lange-Bertalot, Metzeltin & Witkowski	156	17	0.45	8	0.109	FBM	XXL	Motile solitary	Motile	None	None	Low	19	392	413	0.95	3.3
Hippodonta lesmonensis (Hustedt) Lange-Bertalot, Metzeltin & Witkowski	361	37	0.88	2	0.025	MB	M	Motile solitary	Motile	None	None	Low	19	496	542	0.92	2.7
Hyalodiscus scoticus (Kützing) Grunow	147	18	0.27	-	-	B	L	Attached solitary	Attached	None	Pad	Medium	20	1256	3140	0.40	1.0
Karayevia amoena (Hustedt) Bukhtiyarova	1	11	1.02	11	0.143	FB	L	Attached solitary	Creeping	None	Adnate	Low	13	145	85	1.71	3.1
Karayevia clevei (Grunow) Bukhtiyarova	203	40	0.70	16	0.294	FB	M	Attached solitary	Creeping	None	Adnate	Low	13	196	156	1.26	2.2
Karayevia nitidiformis (Lange-Bertalot) Bukhtiyarova	-	-	-	1	0.008	B	L	Attached solitary	Creeping	None	Adnate	Low	14	171	131	1.30	3.3
Karayevia submarina (Hustedt) Bukhtiyarova	104	-	-	7	0.185	FB	M	Attached solitary	Creeping	None	Adnate	Low	10	116	62	1.87	2.6
Lemnicola hungarica (Grunow) Round & Basson	-	-	-	3	0.025	MB	M	Attached solitary	Creeping	None	Adnate	Low	24	418	454	0.92	4.0
Licmophora communis (Heiberg) Grunow	51	5	0.12	-	-	BM	XL	Attached colony	Attached	Bush	Stalk	High	41	2521	6941	0.36	2.3

Licmophora debilis (Kützing) Grunow	351	47	2.06	6	0.151	BM	XL	Attached solitary	Attached	None	Pad	Medium	36	1357	2234	0.61	3.0
Licmophora gracilis var. anglica (Kützing) H. Peragallo & M. Peragallo	352	6	0.08	46	3.025	MB	L	Attached solitary	Attached	None	Pad	Medium	83	8466	38844	0.22	2.8
Licmophora oedipus (Kützing) Grunow	149	-	-	6	0.193	B	M	Attached solitary	Attached	None	Pad	Medium	42	1470	2151	0.68	4.0
Licmophora rhombica Møller	150	14	0.51	2	0.025	MB	XL	Attached solitary	Attached	None	Pad	Medium	35	3312	12460	0.27	1.3
Lunella bisecta Snoeijs	353	85	5.24	69	5.227	FB	XL	Motile solitary	Motile	None	None	Low	8	80	35	2.27	2.6
Luticola mutica (Kützing) D.G. Mann	151	4	0.07	1	0.017	FB	XL	Motile solitary	Motile	None	None	Low	17	425	566	0.75	2.1
Martyana atomus (Hustedt) Snoeijs	53	119	22.44	68	17.000	FB	XL	Attached solitary	Attached	None	Pad	Medium	5	63	33	1.92	1.6
Martyana martyi (Héribaud) Round	354	-	-	31	1.857	BF	XL	Attached solitary	Attached	None	Pad	Medium	16	485	673	0.72	2.0
Martyana schulzii (Brockmann) Snoeijs	54	7	0.10	43	4.244	BF	L	Attached solitary	Attached	None	Pad	Medium	14	330	364	0.91	2.2
Mastogloia baltica Grunow	55	2	0.08	4	0.034	BF	L	Motile solitary	Motile	None	None	Low	36	1659	3318	0.50	3.0
Mastogloia braunii Grunow	56	6	0.17	1	0.008	BF	XL	Motile solitary	Motile	None	None	Low	39	1989	5165	0.39	2.7
Mastogloia elliptica (C.A. Agardh) Cleve	57	21	0.50	15	0.202	BM	XL	Motile solitary	Motile	None	None	Low	32	1339	2921	0.46	2.6
Mastogloia exigua Lewis	247	2	0.09	2	0.067	MB	L	Motile solitary	Motile	None	None	Low	35	1520	3108	0.49	2.9
Mastogloia pumila (Cleve & Möller) Cleve	58	40	3.76	16	0.370	FBM	XXL	Motile solitary	Motile	None	None	Low	26	744	1110	0.67	3.0
Mastogloia pusilla Grunow	249	-	-	1	0.008	FB	XL	Motile solitary	Motile	None	None	Low	19	499	662	0.75	2.7
Mastogloia smithii Thwaites	59	81	11.46	52	2.261	FB	M	Motile solitary	Motile	None	None	Low	34	1256	2402	0.52	3.1
Mastogloia smithii var. amphicephala Grunow	250	-	-	22	0.588	FB	XL	Motile solitary	Motile	None	None	Low	37	1628	3083	0.53	3.1
Mayamaea atomus (Kützing) Lange-Bertalot	-	1	0.01	-	-	B	XXL	Motile solitary	Motile	None	None	Low	8	84	53	1.58	2.4
Melosira arctica Dickie	60	4	0.07	2	0.042	MB	XL	Attached colony	Floating	Chain	Pad	High	20	1283	3490	0.37	1.3
Melosira lineata (Dillwyn) C.A. Agardh = M. jurgensii sensu Kützing 1844	61	77	4.53	31	0.765	MB	XXL	Attached colony	Floating	Chain	Pad	High	29	1628	4671	0.35	2.0
Melosira moniliformis (O.F. Müller) C.A. Agardh	62	67	5.73	21	1.008	MB	XXL	Attached colony	Floating	Chain	Pad	High	37	6172	37231	0.17	1.0
Melosira nummuloides C.A. Agardh	63	22	3.65	18	0.445	B	M	Attached colony	Floating	Chain	Pad	High	17	906	2055	0.44	1.4
Melosira sp. = M. lineata sensu Kützing 1844	-	96	67.92	-	-	FB	M	Attached colony	Floating	Chain	Pad	High	23	1671	4894	0.34	1.0
Melosira varians	64	-	-	2	0.050	BM	L	Attached colony	Floating	Chain	Pad	High	21	1118	2773	0.40	1.6
Navicula arenaria Donkin	452	1	0.01	1	0.017	BM	XL	Motile solitary	Motile	None	None	Low	48	2064	5033	0.41	3.8
Navicula bipustulata A. Mann	356	-	-	2	0.025	BM	XL	Motile solitary	Motile	None	None	Low	28	552	629	0.88	4.7
Navicula bossvikensis Busse & Snoeijs	-	-	-	13	0.462	B	XXL	Motile solitary	Motile	None	None	Low	13	159	103	1.54	3.0
Navicula bottnica Grunow in Cleve & Möller	155	-	-	36	1.017	FB	M	Motile solitary	Motile	None	None	Low	55	2178	4854	0.45	4.7
Navicula bremensis Hustedt	-	18	0.26	1	0.008	FB	M	Motile solitary	Motile	None	None	Low	7	73	29	2.51	2.6
Navicula capitoradiata Germain	-	-	-	3	0.118	BF	XL	Motile solitary	Motile	None	None	Low	34	714	950	0.75	4.6
Navicula cincta (Ehrenberg) Ralfs	-	1	0.05	7	0.059	FBM	XXL	Motile solitary	Motile	None	None	Low	22	306	266	1.15	4.6
Navicula clementis Grunow	183	1	0.01	8	0.109	FBM	XXL	Motile solitary	Motile	None	None	Low	21	551	688	0.80	2.7
Navicula cryptocephala Kützing	157	9	0.20	50	3.739	B	L	Motile solitary	Motile	None	None	Low	25	497	558	0.89	4.1
Navicula digitoradiata (Gregory) Ralfs	158	6	0.07	2	0.025	MB	M	Motile solitary	Motile	None	None	Low	47	2222	5929	0.37	3.2
Navicula duerrenbergiana Hustedt	65	67	3.50	3	0.050	BF	L	Motile solitary	Motile	None	None	Low	48	836	831	1.01	8.8
Navicula eidrigiana Carter	358	-	-	1	0.008	B	L	Motile solitary	Motile	None	None	Low	42	1073	1553	0.69	5.5
Navicula flanatica Grunow	254	7	0.07	6	0.076	FB	L	Motile solitary	Motile	None	None	Low	29	779	966	0.81	4.0
Navicula germainii Wallace	359	-	-	7	0.353	B	XL	Motile solitary	Motile	None	None	Low	46	1526	2597	0.59	4.7
Navicula germanopolonica Witkowski & Lange-Bertalot	255	-	-	8	0.218	FBM	XXL	Motile solitary	Motile	None	None	Low	10	172	135	1.28	2.3
Navicula gregaria Donkin	160	99	4.95	88	8.176	B	M	Motile solitary	Motile	None	None	Low	33	858	1279	0.67	4.0

Navicula hanseatica Lange-Bertalot & Stachura	-	-	-	1	0.017	MB	L	Motile solitary	Motile	None	None	Low	12	126	74	1.70	3.8
Navicula hanseni Møller	-	-	-	22	1.479	BM	L	Motile solitary	Motile	None	None	Low	12	126	74	1.70	3.8
Navicula hustedtiana Simonsen	-	-	-	1	0.008	B	M	Motile solitary	Motile	None	None	Low	12	126	74	1.70	3.8
Navicula infirmitata Giffen	454	62	4.28	-	-	FB	XXL	Motile solitary	Motile	None	None	Low	7	73	29	2.51	2.6
Navicula lanceolata Ehrenberg	66	26	0.52	57	5.218	FB	L	Motile solitary	Motile	None	None	Low	58	2069	4178	0.50	5.5
Navicula laterostrata Hustedt	161	-	-	5	0.050	B	M	Motile solitary	Motile	None	None	Low	24	601	706	0.85	3.4
Navicula margalithii Lange-Bertalot	-	-	-	6	0.311	BF	XL	Motile solitary	Motile	None	None	Low	46	1252	1932	0.65	5.7
Navicula meniscus Schumann	256	2	0.02	8	0.101	BF	L	Motile solitary	Motile	None	None	Low	40	2095	5160	0.41	2.7
Navicula microdigitoradiata Lange-Bertalot	257	-	-	1	0.008	FB	M	Motile solitary	Motile	None	None	Low	30	602	686	0.88	5.2
Navicula nolens Simonsen	-	3	0.04	2	0.025	BF	L	Motile solitary	Motile	None	None	Low	7	73	29	2.51	2.6
Navicula oblonga (Kützing) Kützing	162	-	-	1	0.008	B	L	Motile solitary	Motile	None	None	Low	131	7928	27258	0.29	7.7
Navicula oestrupi Schulz	363	-	-	2	0.017	B	L	Motile solitary	Motile	None	None	Low	23	669	944	0.71	3.0
Navicula peregrina (Ehrenberg) Kützing	163	5	0.07	3	0.235	MB	XXL	Motile solitary	Motile	None	None	Low	103	6510	23072	0.28	5.2
Navicula perminuta Grunow in Van Heurck	69	120	68.90	91	49.218	B	XXL	Attached colony	Motile	Tube	Tube	High	12	126	74	1.70	3.8
Navicula phyllepta Kützing	258	73	2.50	86	5.849	MB	M	Motile solitary	Motile	None	None	Low	14	236	207	1.14	2.8
Navicula portanova Riznyk	164	-	-	1	0.008	FB	M	Motile solitary	Motile	None	None	Low	35	1281	2423	0.53	3.4
Navicula radiosa Kützing	366	-	-	1	0.008	MB	XL	Motile solitary	Motile	None	None	Low	61	2386	5270	0.45	5.1
Navicula ramosissima (C.A. Agardh) Cleve	70	-	-	41	7.782	MB	L	Attached colony	Motile	Tube	Tube	High	27	519	564	0.92	4.6
Navicula ramosissima var. torquata (Harvey) Ross	456	40	1.42	3	0.034	B	M	Attached colony	Motile	Tube	Tube	High	29	662	853	0.78	4.1
Navicula recens (Lange-Bertalot) Lange-Bertalot	-	-	-	1	0.008	FBM	XXL	Motile solitary	Motile	None	None	Low	48	836	831	1.01	8.8
Navicula rhynchocephala Kützing	71	6	0.12	52	1.403	BF	XL	Motile solitary	Motile	None	None	Low	42	1918	4013	0.48	3.3
Navicula salinarum Grunow in Cleve & Grunow	167	8	0.14	10	0.412	MB	XL	Motile solitary	Motile	None	None	Low	25	822	1370	0.60	2.7
Navicula salinicola Hustedt	-	22	0.37	-	-	FBM	XXL	Motile solitary	Motile	None	None	Low	12	126	74	1.70	3.8
Navicula sjoersii Busse & Snoeijs	-	14	0.31	56	4.462	B	XL	Motile solitary	Motile	None	None	Low	10	116	71	1.63	2.4
Navicula starmachioides Witkowski & Lange-Bertalot	458	-	-	8	0.555	B	M	Motile solitary	Motile	None	None	Low	20	209	122	1.71	6.1
Navicula subrhynchocephala Hustedt	-	-	-	1	0.008	B	XL	Motile solitary	Motile	None	None	Low	36	844	1253	0.67	4.3
Navicula supralitoralis Aleem & Hustedt	459	13	0.18	6	0.151	FBM	XXL	Motile solitary	Motile	None	None	Low	7	73	29	2.51	2.6
Navicula tripunctata (O.F. Müller) Bory	260	13	0.21	6	0.143	B	L	Attached colony	Motile	Tube	Tube	High	46	1252	1932	0.65	5.7
Navicula veneta Kützing	-	21	0.41	23	0.437	FB	L	Motile solitary	Motile	None	None	Low	26	415	413	1.00	4.7
Navicymbula pusilla (Grunow) K.Krammer	35	29	0.82	9	0.185	FBM	XXL	Motile solitary	Motile	None	None	Low	20	318	263	1.21	4.0
Nitzschia acicularis (Kützing) W. Smith	73	-	-	4	0.034	FB	M	Motile solitary	Motile	None	None	Low	72	715	373	1.92	20.0
Nitzschia amphibia Grunow	-	11	0.15	-	-	B	L	Motile solitary	Motile	None	None	Low	26	314	242	1.30	6.0
Nitzschia angustatula Lange-Bertalot	460	26	0.41	5	0.151	FB	M	Motile solitary	Motile	None	None	Low	20	259	188	1.38	4.9
Nitzschia angustiforaminata Lange-Bertalot	-	-	-	5	0.109	FB	L	Motile solitary	Motile	None	None	Low	84	2099	2723	0.77	11.4
Nitzschia archibaldii Lange-Bertalot	-	-	-	14	0.697	BM	XL	Motile solitary	Motile	None	None	Low	23	150	61	2.46	10.2
Nitzschia aurariae Cholnoky	262	4	0.07	24	1.546	FB	XXL	Motile solitary	Motile	None	None	Low	12	121	64	1.90	4.1
Nitzschia bacillum Hustedt	462	-	-	24	0.731	FB	M	Motile solitary	Motile	None	None	Low	16	225	160	1.41	3.6
Nitzschia brevissima Grunow	368	9	0.21	-	-	B	XXL	Motile solitary	Motile	None	None	Low	31	661	629	1.05	6.0
Nitzschia commutata Grunow	-	2	0.02	-	-	B	L	Motile solitary	Motile	None	None	Low	80	3157	6463	0.49	7.3
Nitzschia dippelii Grunow	263	-	-	10	0.160	FB	L	Motile solitary	Motile	None	None	Low	84	2099	2723	0.77	11.4

Nitzschia dissipata (Kützing) Grunow	369	10	0.15	17	0.639	MB	XL	Motile solitary	Motile	None	None	Low	25	342	240	1.43	6.3
Nitzschia dissipata var. media (Hantzsch) Grunow	-	-	-	4	0.034	FB	XL	Motile solitary	Motile	None	None	Low	25	284	206	1.38	6.1
Nitzschia distans Gregory	370	-	-	7	0.269	BF	L	Motile solitary	Motile	None	None	Low	47	1303	1718	0.76	5.3
Nitzschia dubia W. Smith	264	2	0.03	2	0.034	B	L	Motile solitary	Motile	None	None	Low	80	3157	6463	0.49	7.3
Nitzschia elegantula Grunow	266	10	0.21	6	0.092	B	XL	Motile solitary	Motile	None	None	Low	15	185	107	1.73	4.1
Nitzschia filiformis (W. Smith) Van Heurck	74	49	10.55	-	-	B	M	Attached colony	Motile	Tube	Tube	High	64	1072	1007	1.06	12.1
Nitzschia flexa Schumann	-	10	0.20	-	-	BF	XL	Floating colony	Floating	Zigzag	None	Medium	104	2105	2388	0.88	16.3
Nitzschia frigida Grunow	75	-	-	2	0.067	FBM	XXL	Floating colony	Floating	Zigzag	None	Medium	42	729	706	1.03	9.1
Nitzschia frustulum (Kützing) Grunow	76	121	98.73	99	58.000	B	M	Motile solitary	Motile	None	None	Low	21	150	61	2.45	9.0
Nitzschia gandersheimiensis Krasske	-	6	0.11	-	-	BM	L	Motile solitary	Motile	None	None	Low	61	885	722	1.23	13.2
Nitzschia grossestriata Hustedt	371	-	-	9	0.336	BF	XL	Motile solitary	Motile	None	None	Low	36	489	373	1.31	8.4
Nitzschia heufleriana Grunow	372	4	0.05	24	0.353	FB	XXL	Motile solitary	Motile	None	None	Low	169	3178	3390	0.94	30.2
Nitzschia inconspicua Grunow	77	23	0.36	77	49.756	FB	M	Motile solitary	Motile	None	None	Low	8	55	21	2.59	3.9
Nitzschia intermedia Hantzsch	-	-	-	3	0.387	FB	L	Motile solitary	Motile	None	None	Low	80	1392	1512	0.92	13.3
Nitzschia liebetruthii Rabenhorst	-	77	3.71	46	4.370	FB	L	Motile solitary	Motile	None	None	Low	26	296	214	1.38	6.4
Nitzschia linearis var. subtilis (Grunow) Hustedt	-	7	0.11	24	1.765	B	M	Motile solitary	Motile	None	None	Low	105	1523	1378	1.10	21.0
Nitzschia lorenziana Grunow	464	1	0.02	1	0.008	FB	XXL	Motile solitary	Motile	None	None	Low	173	2474	1903	1.30	34.6
Nitzschia microcephala Grunow	171	110	17.82	77	6.790	FB	L	Motile solitary	Motile	None	None	Low	10	105	55	1.90	3.4
Nitzschia palea (Kützing) W. Smith	-	56	1.54	17	0.571	B	M	Motile solitary	Motile	None	None	Low	26	268	157	1.71	7.8
Nitzschia palea var. tenuirostris Grunow	-	27	0.94	-	-	BF	XL	Motile solitary	Motile	None	None	Low	28	244	132	1.84	9.3
Nitzschia paleacea Grunow	172	109	29.95	22	0.487	FB	M	Motile solitary	Motile	None	None	Low	24	135	43	3.13	13.2
Nitzschia pellucida Grunow	-	61	4.34	6	0.193	FB	M	Motile solitary	Motile	None	None	Low	60	1218	1544	0.79	8.6
Nitzschia perminuta (Grunow) M. Peragallo	-	12	0.20	4	0.118	BF	XXL	Motile solitary	Motile	None	None	Low	14	142	90	1.58	4.0
Nitzschia pusilla Grunow	267	65	5.65	49	2.487	FB	M	Motile solitary	Motile	None	None	Low	23	384	328	1.17	4.9
Nitzschia recta Hantzsch in Rabenhorst	268	-	-	3	0.025	MB	L	Motile solitary	Motile	None	None	Low	99	2150	2652	0.81	16.2
Nitzschia reversa W. Smith	269	11	0.17	-	-	FB	L	Motile solitary	Motile	None	None	Low	188	2471	1116	2.21	30.7
Nitzschia rosenstockii Lange-Bertalot	-	118	30.29	71	9.361	BF	L	Motile solitary	Motile	None	None	Low	14	125	62	2.02	5.0
Nitzschia salinicola Aleem & Hustedt	466	-	-	2	0.109	B	XL	Motile solitary	Motile	None	None	Low	32	529	452	1.17	6.8
Nitzschia sigma (Kützing) W. Smith	173	15	0.22	22	0.286	FB	XL	Motile solitary	Motile	None	None	Low	388	13855	27744	0.50	34.3
Nitzschia sigmoidea (Nitzsch) W. Smith	271	1	0.01	1	0.017	FB	L	Motile solitary	Motile	None	None	Low	314	23004	92325	0.25	15.9
Nitzschia sociabilis Hustedt	-	5	0.07	9	0.227	FB	M	Motile solitary	Motile	None	None	Low	38	512	390	1.31	9.5
Nitzschia solita Hustedt	-	-	-	9	0.244	B	M	Motile solitary	Motile	None	None	Low	35	609	662	0.92	5.8
Nitzschia subcapitellata Hustedt	-	3	0.05	-	-	FB	M	Motile solitary	Motile	None	None	Low	25	342	240	1.43	6.3
Nitzschia sublinearis Hustedt	-	-	-	1	0.017	FB	M	Motile solitary	Motile	None	None	Low	104	2105	2388	0.88	16.3
Nitzschia supralitorea Hustedt	-	50	1.31	-	-	BF	XXL	Motile solitary	Motile	None	None	Low	26	268	157	1.71	7.8
Nitzschia thermaloides Hustedt	78	76	4.73	50	1.958	FB	M	Motile solitary	Motile	None	None	Low	59	1319	1680	0.78	9.3
Nitzschia umbonata (Ehrenberg) Lange-Bertalot	-	-	-	1	0.008	FB	XL	Motile solitary	Motile	None	None	Low	25	342	240	1.43	6.3
Nitzschia valdestriata Aleem & Hustedt	174	69	2.06	51	1.857	FB	M	Motile solitary	Motile	None	None	Low	7	64	33	1.94	2.6
Nitzschia vermicularis (Kützing) Hantzsch	-	-	-	1	0.008	B	L	Motile solitary	Motile	None	None	Low	170	2958	3213	0.92	28.3
Nitzschia vitrea Norman	468	-	-	18	0.294	FB	M	Motile solitary	Motile	None	None	Low	66	1506	1940	0.78	9.4

Nitzschia wuellerstorfii Lange-Bertalot	-	-	-	1	0.008	B	L	Motile solitary	Motile	None	None	Low	26	268	157	1.71	7.8
Opephora krumbeinii Witkowski, Witak & Stachura	-	-	-	31	9.504	BM	XXL	Attached colony	Attached	Ribbon	Pad	Low	13	190	140	1.36	3.1
Opephora mutabilis (Grunow) Sabbe & Vijverman	79	118	21.54	32	7.832	MB	XL	Attached colony	Attached	Ribbon	Pad	Low	13	190	140	1.36	3.1
Parlibellus protracta (Grunow) Witkowski, Lange-Bertalot & Metzeltin	165	-	-	11	0.134	B	XXL	Attached colony	Motile	Tube	Tube	High	24	671	894	0.75	3.2
Pauliella taeniata (Grunow) Round & Basson	6	31	0.61	15	0.261	BM	XL	Floating colony	Floating	Ribbon	None	Medium	21	574	898	0.64	2.8
Petrodictyon gemma (Ehrenberg) D.G.Mann	179	1	0.01	-	-	MB	L	Motile solitary	Motile	None	None	Low	133	19607	118772	0.17	2.1
Petroneis humerosa (Brébisson) Stickle & D.G. Mann	180	-	-	2	0.034	B	XL	Motile solitary	Motile	None	None	Low	66	7181	32440	0.22	2.1
Pinnularia appendiculata (C. A. Agardh) Cleve	376	-	-	11	0.168	FB	XL	Motile solitary	Motile	None	None	Low	21	362	323	1.12	4.3
Pinnularia divergentissima var. minor Krammer	-	-	-	1	0.008	FB	M	Motile solitary	Motile	None	None	Low	22	281	229	1.23	4.8
Pinnularia elegans (W.Smith) Krammer	277	1	0.01	-	-	MB	L	Motile solitary	Motile	None	None	Low	78	5697	19820	0.29	3.5
Pinnularia krockii (Grunow) Hustedt	-	6	0.10	2	0.034	FB	M	Motile solitary	Motile	None	None	Low	26	452	491	0.92	4.3
Pinnularia microstauron (Ehrenberg) Cleve	-	1	0.02	1	0.034	FB	M	Motile solitary	Motile	None	None	Low	34	592	643	0.92	5.7
Placoneis placentula (Ehrenberg) Mereschkowsky	278	-	-	1	0.008	FB	L	Motile solitary	Motile	None	None	Low	24	869	1512	0.57	2.2
Planothidium conspicuum (Mayer) Morales	-	-	-	1	0.017	B	L	Attached solitary	Creeping	None	Adnate	Low	8	90	54	1.65	2.0
Planothidium delicatulum (Kützing) Round & Bukhtiyarova	3	58	1.03	91	7.832	FB	L	Attached solitary	Creeping	None	Adnate	Low	14	213	159	1.34	2.3
Planothidium dispar (Cleve) Witkowski, Lange-Bertalot & Metzeltin	102	1	0.02	3	0.050	FB	XXL	Attached solitary	Creeping	None	Adnate	Low	23	538	652	0.83	2.6
Planothidium frequentissimum (Lange-Bertalot) Round & Bukhtiyarova	304	11	0.12	10	0.529	FB	M	Attached solitary	Creeping	None	Adnate	Low	11	151	112	1.35	2.2
Planothidium hauckianum (Grunow) Round & Buktiarova	-	96	3.09	64	3.605	FB	XXL	Attached solitary	Creeping	None	Adnate	Low	8	90	54	1.65	2.0
Planothidium lanceolatum (Brébisson) Lange-Bertalot	-	-	-	2	0.076	B	L	Attached solitary	Creeping	None	Adnate	Low	23	522	756	0.69	2.8
Planothidium lemmermannii (Hustedt) Morales	305	52	1.12	17	0.521	FB	XL	Attached solitary	Creeping	None	Adnate	Low	10	116	62	1.87	2.6
Planothidium linkei (Hustedt) Lange-Bertalot	306	-	-	6	0.134	FB	M	Attached solitary	Creeping	None	Adnate	Low	27	680	820	0.83	3.0
Planothidium pericavum (J.R.Carter) Lange-Bertalot	404	-	-	8	0.193	B	L	Attached solitary	Creeping	None	Adnate	Low	17	270	237	1.14	2.9
Planothidium rostratum (Østrup) Round & Bukhtiyarova	311	-	-	2	0.017	BM	XL	Attached solitary	Creeping	None	Adnate	Low	14	191	138	1.38	2.9
Planothidium septentrionalis (Østrup) Round & Bukhtiyarova	103	14	0.26	9	0.319	FBM	XXL	Attached solitary	Creeping	None	Adnate	Low	20	362	337	1.08	2.9
Pleurosigma elongatum W. Smith	281	10	0.14	-	-	FB	M	Motile solitary	Motile	None	None	Low	297	20751	75318	0.28	11.6
Pleurosigma salinarum (Grunow) Grunow	283	9	0.19	-	-	FBM	XXL	Motile solitary	Motile	None	None	Low	106	4361	8602	0.51	7.6
Proschkinia bulnheimii (Grunow) Karayeva	-	40	1.98	-	-	B	M	Motile solitary	Motile	None	None	Low	26	746	1025	0.73	3.2
Psammothidium bioretii (Germain) Bukhtiyarova & Round	202	-	-	2	0.017	FB	XL	Attached solitary	Creeping	None	Adnate	Low	16	363	419	0.87	2.0
Psammothidium oblongellum (Østrup) Van de Vijver	308	-	-	1	0.025	BF	L	Attached solitary	Creeping	None	Adnate	Low	14	235	193	1.22	2.1
Psammothidium punctulatum (Simonsen) Bukhtiyarova & Round	309	21	0.34	34	1.311	FB	M	Attached solitary	Creeping	None	Adnate	Low	12	143	88	1.61	2.5
Psammothidium rossii (Hustedt) L.Bukhtiyarova & Round	310	-	-	3	0.168	B	M	Attached solitary	Creeping	None	Adnate	Low	12	167	120	1.38	2.1
Psammothidium sacculum (Carter) Bukhtiyarova	-	-	-	1	0.008	BF	XXL	Attached solitary	Creeping	None	Adnate	Low	12	167	120	1.38	2.1
Pseudofallacia tenera (Hustedt) Liu, Kociolek & Wan	242	2	0.03	8	0.126	FB	M	Motile solitary	Motile	None	None	Low	11	190	154	1.23	2.2
Pseudopodosira westii (W.Smith) Sheshukova-Poretzskaya & Glezer	153	3	0.03	-	-	FB	M	Floating colony	Floating	Chain	None	Medium	10	340	442	0.77	1.0
Pseudostaurosira brevistriata (Grunow) Williams & Round	479	74	1.86	34	4.445	FB	L	Attached colony	Attached	Ribbon	Pad	Low	12	192	154	1.25	3.0
Pseudostaurosira brevistriata var. inflata (Pantocsek) Hartley	480	-	-	28	3.353	FB	M	Attached colony	Attached	Ribbon	Pad	Low	14	213	157	1.36	3.5
Pseudostaurosira elliptica (Schumann) Edlund, Morales & Spaulding	491	121	96.03	61	15.487	BM	XL	Attached colony	Attached	Ribbon	Pad	Low	4	61	41	1.48	1.2
Pseudostaurosira perminuta (Grunow) Sabbe & Vijverman	481	-	-	34	5.261	MB	M	Attached colony	Attached	Ribbon	Pad	Low	11	129	64	2.00	3.7
Pseudostaurosira robusta (Fusey) D.M.Williams & Round	-	-	-	17	2.000	B	M	Attached colony	Attached	Ribbon	Pad	Low	7	112	90	1.25	1.8
Pseudostaurosira subsalina (Hustedt) Morales	-	-	-	12	4.555	FB	XL	Attached colony	Attached	Ribbon	Pad	Low	4	61	41	1.48	1.2

Pseudostaurosira zeilleri (Héribaud-Joseph) Williams & Round	482	116	29.40	66	16.790	FB	L	Attached colony	Attached	Ribbon	Pad	Low	7	112	90	1.25	1.8
Pteroncola inane (Giffen) Round	80	-	-	7	3.639	FB	M	Attached colony	Attached	Zigzag	Pad	High	9	160	129	1.24	1.7
Reimeria sinuata (Gregory) Kociolek & Stoermer	388	2	0.02	2	0.025	MB	L	Motile solitary	Motile	None	None	Low	17	241	180	1.34	4.1
Rhoicosphenia abbreviata (C.A. Agardh) Lange-Bertalot	81	121	47.02	116	53.622	MB	M	Attached solitary	Attached	None	Stalk	Medium	24	545	698	0.78	3.9
Rhopalodia acuminata Krammer	288	-	-	1	0.017	FB	L	Motile solitary	Motile	None	None	Low	49	1833	3717	0.49	2.9
Rhopalodia brebissonii Krammer	483	-	-	5	0.134	FB	XL	Motile solitary	Motile	None	None	Low	25	764	1109	0.69	1.9
Rhopalodia gibba (Ehrenberg) Otto Müller	82	41	1.07	13	0.286	B	M	Motile solitary	Motile	None	None	Low	60	2618	6216	0.42	3.0
Rhopalodia gibba var. parallela (Grunow) H. Peragallo & M. Peragallo	-	5	0.08	-	-	FB	M	Motile solitary	Motile	None	None	Low	150	8813	24609	0.36	6.0
Sellaphora pupula Kützing	289	3	0.07	1	0.017	FB	M	Motile solitary	Motile	None	None	Low	22	537	694	0.77	3.0
Sellaphora stroemii (Hustedt) Kobayasi	-	-	-	1	0.017	MB	M	Motile solitary	Motile	None	None	Low	22	537	694	0.77	3.0
Skeletonema costatum (Greville) Cleve	83	32	1.02	4	0.151	FBM	XXL	Floating colony	Floating	Chain	None	Medium	10	333	459	0.73	1.4
Skeletonema subsalsum (A. Cleve) Bethge	290	-	-	1	0.008	FB	L	Floating colony	Floating	Chain	None	Medium	17	333	353	0.94	3.0
Stauroneis legumen (Ehrenberg) Kützing	-	-	-	2	0.025	FB	M	Motile solitary	Motile	None	None	Low	28	568	720	0.79	4.0
Stauroneis phoenicenteron (Nitzsch) Ehrenberg	395	-	-	1	0.017	B	L	Motile solitary	Motile	None	None	Low	122	11392	50275	0.23	4.2
Stauroneis simulans (Donkin) Ross	187	1	0.02	11	0.748	FB	XXL	Motile solitary	Motile	None	None	Low	31	1209	2096	0.58	2.4
Stauroneis smithii var. karelica Wislouch & Kolbe	-	-	-	1	0.008	FB	M	Motile solitary	Motile	None	None	Low	23	450	550	0.82	3.4
Staurophora anuschkae Witkowski	-	-	-	4	0.185	FB	M	Motile solitary	Motile	None	None	Low	31	1209	2096	0.58	2.4
Staurophora wislouchii (Poretzky & Anisimova) D.G. Mann	487	-	-	1	0.008	FB	XL	Motile solitary	Motile	None	None	Low	40	1813	4637	0.39	2.9
Staurosira punctiformis Witkowski, Metzeltin & Lange-Bertalot	-	-	-	41	13.218	FB	L	Attached colony	Attached	Ribbon	Pad	Low	4	61	41	1.48	1.2
Staurosira venter (Ehrenberg) Kobayasi	490	-	-	10	1.479	FB	L	Attached colony	Attached	Ribbon	Pad	Low	8	144	120	1.20	1.5
Staurosirella lapponica (Grunow) Williams & Round	-	-	-	1	0.008	FB	M	Attached colony	Attached	Ribbon	Pad	Low	7	112	90	1.25	1.8
Staurosirella leptostauron (Ehrenberg) Williams & Round	-	-	-	1	0.008	FB	M	Attached colony	Attached	Ribbon	Pad	Low	15	312	403	0.77	1.9
Stephanodiscus hantzschii Grunow	84	2	0.02	4	0.067	BM	XL	Floating solitary	Floating	None	None	Low	11	346	454	0.76	1.0
Stephanodiscus minutulus (Kützing) Cleve & Möller	190	1	0.01	-	-	FB	M	Floating solitary	Floating	None	None	Low	8	181	172	1.05	1.0
Stephanodiscus parvus Stoermer & Håkansson	191	-	-	3	0.050	BM	L	Floating solitary	Floating	None	None	Low	7	137	113	1.21	1.0
Surirella angustata Hustedt	-	-	-	2	0.017	FB	M	Motile solitary	Motile	None	None	Low	50	1740	3780	0.46	4.2
Surirella brebissonii Krammer & Lange-Bertalot	86	56	1.39	69	2.454	BF	XL	Motile solitary	Motile	None	None	Low	28	977	1451	0.67	1.7
Surirella brightwellii var. baltica (Schumann) Krammer	493	-	-	9	0.109	BF	XXL	Motile solitary	Motile	None	None	Low	34	1421	2393	0.59	1.8
Surirella minuta Brébisson	87	-	-	1	0.008	FB	M	Motile solitary	Motile	None	None	Low	47	871	758	1.15	5.7
Surirella ovalis Brébisson	-	-	-	1	0.042	FB	M	Motile solitary	Motile	None	None	Low	47	871	758	1.15	5.7
Synedra acus Kützing	294	2	0.02	9	0.261	FB	M	Attached colony	Attached	Bush	Pad	Medium	150	1617	910	1.78	42.9
Synedra ulna (Nitzsch) Ehrenberg	295	-	-	6	0.202	FB	M	Attached colony	Attached	Bush	Pad	Medium	218	4021	4133	0.97	41.1
Tabellaria fenestrata (Lyngbye) Kützing	192	2	0.02	1	0.017	B	XXL	Attached colony	Attached	Bush	Pad	Medium	59	1912	2390	0.80	6.6
Tabellaria flocculosa (Roth) Kützing	193	3	0.03	6	0.067	MB	XXL	Attached colony	Attached	Bush	Pad	Medium	36	2902	5054	0.57	1.4
Tabularia fasciculata (C.A. Agardh) Williams & Round	89	120	24.43	87	55.613	FB	M	Attached colony	Attached	Bush	Pad	Medium	61	1093	1104	0.99	11.6
Tabularia ktenoeides Kuylenstierna	91	-	-	3	0.084	MB	XXL	Attached colony	Attached	Bush	Pad	Medium	21	415	459	0.91	3.7
Tabularia laevis Kützing	-	-	-	34	1.782	MB	M	Attached colony	Attached	Bush	Pad	Medium	61	873	696	1.25	13.6
Tabularia tabulata (C.A. Agardh) Snoeijs	92	70	5.61	44	0.908	MB	XL	Attached colony	Attached	Bush	Pad	Medium	273	7586	11967	0.63	35.0
Tabularia waernii Snoeijs	93	1	0.01	51	8.966	BM	XL	Attached colony	Attached	Bush	Pad	Medium	26	241	129	1.87	8.8
Tetracyclus glans (Ehrenberg) Mills	-	-	-	1	0.008	FB	M	Attached colony	Attached	Zigzag	Pad	High	50	2767	9610	0.29	2.3

Thalassiosira baltica (Grunow) Ostenfeld	95	49	0.72	25	0.345	BF	XL	Floating colony	Floating	Chain	None	Medium	55	9637	66737	0.14	1.0
Thalassiosira hyperborea var. lacunosa (Berg) Hasle	195	-	-	9	0.092	MB	M	Floating colony	Floating	Chain	None	Medium	25	1994	6281	0.32	1.0
Thalassiosira levanderi Van Goor	98	35	0.65	49	1.824	B	M	Floating colony	Floating	Chain	None	Medium	10	327	417	0.78	1.0
Thalassiosira proschkinae Makarova	99	32	1.54	5	0.109	B	XXL	Floating colony	Floating	Chain	None	Medium	5	92	62	1.48	1.0
Thalassiosira pseudonana Hasle & Heimdal	100	45	0.74	3	0.042	MB	XXL	Floating colony	Floating	Chain	None	Medium	7	145	123	1.18	1.0
Thalassiosira punctigera (Castracane) Hasle	495	-	-	1	0.017	MB	XL	Floating colony	Floating	Chain	None	Medium	50	7850	49063	0.16	1.0
Tropidoneis dannfeltii Cleve-Euler	-	1	0.02	-	-	B	M	Motile solitary	Motile	None	None	Low	159	23596	158936	0.15	3.8
Tryblionella angustata W. Smith	-	1	0.02	5	0.067	FB	M	Motile solitary	Motile	None	None	Low	63	1178	1386	0.85	9.6
Tryblionella apiculata Gregory	196	26	0.45	13	0.235	B	XL	Motile solitary	Motile	None	None	Low	38	858	993	0.86	5.8
Tryblionella ardua (Cholnoky) D.G. Mann	461	7	0.12	3	0.076	B	L	Motile solitary	Motile	None	None	Low	23	315	243	1.29	5.5
Tryblionella hungarica (Grunow) Frenguelli	298	1	0.01	2	0.092	FB	XL	Motile solitary	Motile	None	None	Low	43	856	885	0.97	7.8
Tryblionella levidensis W. Smith	198	9	0.15	2	0.017	B	L	Motile solitary	Motile	None	None	Low	46	2793	8015	0.35	2.8
Tryblionella littoralis var. tergestina (Grunow) Snoeijs	497	-	-	1	0.017	B	M	Motile solitary	Motile	None	None	Low	68	5358	18496	0.29	3.4
Tryblionella salinarum (Grunow) Pelletan	299	-	-	9	0.168	BF	XL	Motile solitary	Motile	None	None	Low	36	1145	1743	0.66	4.4
Tryblionella scalaris (Ehrenberg) Siver & Hamilton	270	-	-	1	0.008	FB	M	Motile solitary	Motile	None	None	Low	653	62895	343482	0.18	24.8
