# Supplementary material for: Diatom Cell Size, Coloniality and Motility: Trade-Offs between Temperature, Salinity and Nutrient Supply with Climate Change
Source: PLoS One. 2014 Oct 3;9(10):e109993. doi: 10.1371/journal.pone.0109993 (PMC4184900; doi:10.1371/journal.pone.0109993)
Supplement: Table S7 — Result from the autocorrelation analyses in the temperature and salinity gradients, showing the P-values from the RDA analysis using eigenvectors describing positive spatial and temporal autocorrelation. (PDF) [file pone.0109993.s007.pdf]

Table S7. Result from the autocorrelation analyses in the temperature and salinity gradients, showing the P-values from the RDA analysis using eigenvectors describing positive spatial and temporal autocorrelation.

Spatial autocorrelation within dates<br><br>Temperature gradient		Temporal autocorrelation within sites<br><br>Temperature gradient		Spatial autocorrelation within areas<br><br>Salinity gradient	
Sampling date	P-value	Sampling site	P-value	Sampling area	P-value
840502	0.42	A	0.015	Hal	0.32
840523	0.71	B	0.075	Kal	0.29
840612	0.29	C	0.010	Kvä	0.82
840703	0.33	D	0.005	Him	0.13
840724	0.41	E	0.005	Grä	0.38
840814	0.94	F	0.047	Gäv	0.67
840906	0.60	G	0.005	Hor	0.69
840926	0.68	H	0.105	Omn	0.13
841016	0.64	I	0.005	Hol	0.15
841106	0.61	J	0.015	Ske	0.22
841127	0.37	K	0.005	Rån	0.02
