# Supplementary material for: Diatom Cell Size, Coloniality and Motility: Trade-Offs between Temperature, Salinity and Nutrient Supply with Climate Change
Source: PLoS One. 2014 Oct 3;9(10):e109993. doi: 10.1371/journal.pone.0109993 (PMC4184900; doi:10.1371/journal.pone.0109993)
Supplement: Table S8 — Correlations between cell size and shape traits in the temperature gradient. The lower triangle shows Spearman rank correlation coefficients, the upper triangle shows the adjusted P-values using Holm's method. (PDF) [file pone.0109993.s008.pdf]

Table S8. Correlations between cell size and shape traits in the temperature gradient. The lower triangle shows Spearman rank correlation coefficients, the upper triangle shows the adjusted P-values using Holm’s method.

	Length	Surface	Volume	S:V	Shape
Length		<0.0001	<0.0001	<0.0001	<0.0001
Surface	0.90		<0.0001	<0.0001	0.054
Volume	0.83	0.98		<0.0001	0.726
S:V	-0.62	-0.87	-0.94		0.002
Shape	0.51	0.15	0.02	0.23	
