# Supplementary material for: Diatom Cell Size, Coloniality and Motility: Trade-Offs between Temperature, Salinity and Nutrient Supply with Climate Change
Source: PLoS One. 2014 Oct 3;9(10):e109993. doi: 10.1371/journal.pone.0109993 (PMC4184900; doi:10.1371/journal.pone.0109993)
Supplement: Table S9 — Correlations between cell size and shape traits in the salinity gradient. The lower triangle shows Spearman rank correlation coefficients, the upper triangle shows the adjusted P-values using Holm's method. (PDF) [file pone.0109993.s009.pdf]

Table S9. Correlations between cell size and shape traits in the salinity gradient. The lower triangle shows Spearman rank correlation coefficients, the upper triangle shows the adjusted P-values using Holm’s method.

	Length	Surface	Volume	S:V	Shape
Length		<0.0001	<0.0001	<0.0001	<0.0001
Surface	0.91		<0.0001	<0.0001	0.003
Volume	0.85	0.99		<0.0001	0.232
S:V	-0.66	-0.89	-0.95		0.003
Shape	0.51	0.17	0.06	0.17	
