# Supplementary material for: Diatom Cell Size, Coloniality and Motility: Trade-Offs between Temperature, Salinity and Nutrient Supply with Climate Change
Source: PLoS One. 2014 Oct 3;9(10):e109993. doi: 10.1371/journal.pone.0109993 (PMC4184900; doi:10.1371/journal.pone.0109993)
Supplement: Table S10 — Correlations between the environmental and biotic variables in the temperature gradient. The lower triangle shows Spearman rank correlation coefficients, the upper triangle shows the adjusted P-values using Holm's method. Abbreviations cf. Figure 5. (PDF) [file pone.0109993.s010.pdf]

Table S10. Correlations between the environmental and biotic variables in the temperature gradient. The lower triangle shows Spearman rank correlation coefficients, the upper triangle shows the adjusted P-values using Holm's method. Abbreviations cf. Figure 5.

	Temp	Seastemp	Spatanom	PAR	DIN	DIP	DSi	N:P	FLOW	Richness	MPB	Maccov	Fauna
Temp		<0.0001	<0.0001	<0.0001	0.035	0.190	1.000	0.082	0.211	0.110	1.000	1.000	0.036
Seastemp	0.74		1.000	<0.0001	0.005	0.302	1.000	0.0026	1.000	0.007	1.000	1.000	1.000
Spatanom	0.56	0.00		1.000	1.000	1.000	1.000	1.000	<0.0001	1.000	1.000	1.000	1.000
PAR	0.43	0.55	0.00		<0.0001	<0.0001	<0.0001	<0.0001	1.000	1.000	1.000	1.000	1.000
DIN	-0.31	-0.35	0.00	-0.82		<0.0001	<0.0001	<0.0001	1.000	1.000	1.000	1.000	1.000
DIP	-0.26	-0.25	0.00	-0.77	0.80		<0.0001	<0.0001	1.000	1.000	1.000	1.000	1.000
DSi	-0.21	-0.14	0.00	-0.77	0.83	0.80		<0.0001	1.000	1.000	1.000	1.000	1.000
N:P	-0.29	-0.36	0.00	-0.71	0.95	0.57	0.71		1.000	1.000	1.000	1.000	1.000
FLOW	0.26	0.00	0.45	0.00	0.00	0.00	0.00	0.00		0.668	1.000	1.000	1.000
Richness	0.28	0.34	0.05	0.09	-0.03	0.00	0.09	-0.04	0.22		1.000	0.223	1.000
MPB	0.00	-0.17	0.06	-0.19	0.12	0.02	0.03	0.12	0.17	-0.01		0.003	0.110
Maccov	0.15	-0.05	0.15	-0.16	0.19	0.13	0.17	0.20	0.13	-0.26	0.36		<0.0001
Fauna	0.31	0.16	0.15	0.10	-0.06	-0.11	-0.09	-0.01	0.02	-0.09	0.28	0.65	
