# Supplementary material for: Diatom Cell Size, Coloniality and Motility: Trade-Offs between Temperature, Salinity and Nutrient Supply with Climate Change
Source: PLoS One. 2014 Oct 3;9(10):e109993. doi: 10.1371/journal.pone.0109993 (PMC4184900; doi:10.1371/journal.pone.0109993)
Supplement: Table S11 — Correlations between the environmental and biotic variables in the salinity gradient. The lower triangle shows Spearman rank correlation coefficients, the upper triangle shows the adjusted P-values using Holm's method. Abbreviations cf. Figure 6. (PDF) [file pone.0109993.s011.pdf]

Table S11. Correlations between the environmental and biotic variables in the salinity gradient. The lower triangle shows Spearman rank correlation coefficients, the upper triangle shows the adjusted P-values using Holm’s method. Abbreviations cf. Figure 6.

	Salinity	Temp	DIN	DIP	DSi	N:P	EXP	SAND	BEACH	Richness	ADW	ADW%
Salinity		<0.0001	<0.0001	0.009	<0.0001	<0.0001	1.000	0.158	0.029	0.002	<0.0001	<0.0001
Temp	0.59		<0.0001	1.000	<0.0001	1.000	<0.0001	0.0002	<0.0001	1.000	0.001	1.000
DIN	-0.73	-0.48		1.000	<0.0001	<0.0001	1.000	0.127	1.000	0.094	<0.0001	<0.0001
DIP	0.33	-0.08	-0.14		1.000	<0.0001	1.000	1.000	1.000	1.000	1.000	1.000
DSi	-0.89	-0.65	0.72	-0.14		<0.0001	1.000	0.024	0.019	0.004	<0.0001	<0.0001
N:P	-0.61	-0.17	0.63	-0.83	0.48		1.000	1.000	1.000	0.049	0.015	0.014
EXP	-0.14	-0.50	0.06	-0.14	0.15	0.13		0.049	<0.0001	0.005	1.000	0.152
SAND	0.25	0.41	-0.26	0.05	-0.31	-0.16	-0.29		<0.0001	0.163	<0.0001	0.0003
BEACH	-0.30	-0.44	0.18	-0.08	0.31	0.16	0.53	-0.46		1.000	1.000	0.557
Richness	-0.36	0.11	0.27	-0.16	0.35	0.29	-0.34	0.25	-0.09		1.000	<0.0001
ADW	0.46	0.38	-0.46	0.12	-0.47	-0.32	-0.06	0.44	-0.17	0.03		1.000
ADW%	0.49	0.07	-0.42	0.12	-0.45	-0.32	0.25	-0.40	0.21	-0.60	0.13	
